# Supplementary material for: Improving the mental health and mental health support available to adolescents in out-of-home care via Adolescent-Focused Low-Intensity Life Story Work: a realist review
Source: BMJ Open. 2023 Oct 9;13(10):e075093. doi: 10.1136/bmjopen-2023-075093 (PMC10565277; doi:10.1136/bmjopen-2023-075093)
Supplement: Supplementary data [file bmjopen-2023-075093supp002.pdf]

Improving the mental health and mental health support available to adolescents in out-of-home care via Adolescent-Focused Low-Intensity Life Story Work: A realist review

Supplementary File 2: Detailed summary of the included documents

Authors	Year	Country	Title	Publication type/ Study Design/ Methods	Sample/ Setting	Objectives
ARCBOX [93]		United Kingdom (UK)	Digital Life Story	Webpage	Adoption	To describe a digital platform for adopted young people to collect memories throughout their life from the past to the present day.
Atwell [97]	2016	UK	Working with children with a disability.	Book Chapter	Across all settings	To discuss Life Story Work (LSW) with children and young people with special educational needs or disabilities (SEND).
Atwool [43]	2017	New Zealand	Life Story Work Optional Extra or Fundamental Entitlement.	Journal, Commentary/ practice guidance	Across all settings	To discuss LSW practice in New Zealand and the reasons why LSW is fundamental to young people with care experience.
Aust [87]	1981	United States of America (USA)	Using the Life Story Book in Treatment of Children in Placement	Journal, Case Study	Foster Placement	To describe the use of life story books in foster placements using a case study example.
Aventin, Houston, & Macdonald [66]	2014	UK	Utilising a computer game as a therapeutic intervention for youth in residential care Some preliminary findings	Journal, Semi-structured interviews	Residential placement (N = 16 consisting of 8 young person and	To explore the accessibility and therapeutic impact of a computer-based intervention delivered to young people in residential placements by their key worker.

Improving the mental health and mental health support available to adolescents in out-of-home care via Adolescent-Focused Low-Intensity Life Story Work: A realist review

			on use and acceptability.		worker pairs)	
BASW [73]	2020	UK	Recording in children's social work	Commentary/ practice guidance	Across all settings	To discuss recording in social work providing ten recommendations for practice.
Baynes [18]	2008	UK	Untold stories A discussion of life story work.	Journal, Commentary/ practice guidance	Across all settings	To discuss LSW practice by social care professionals and the issues surrounding practice.
Bazalgette, Rahilly &Trevelyan [91]	2015	UK	Achieving-emotional-wellbeing-for-looked-after children a whole system approach	Report, Commentary/ practice guidance	Across all settings	To discuss recommendations based on research for a whole system approach to improving the emotional wellbeing of young people with care experience by delivering preventative as opposed to crisis driven interventions, including LSW.
Beste & Richardson [92]	1981	USA	Developing a Life Story Book Program for Foster Children	Journal, Commentary/ practice guidance	Across all settings	To describe a training programme for foster carers to support young people to create a Life Story Book.
Bolton Council [58]	2022	UK	Life Story Work 5 minute facts	Webpage, Commentary/ practice guidance	Foster placement	To provide guidance for carrying out LSW within foster placements.
Braiden [62]	2016	UK	Evaluation of a short training programme for foster carers	Journal, Case study	Foster placement	To discuss a training package for foster carers that provides skills around art and creative therapy they can engage in with the young person in their care.
Brookfield, Brown, & Reavey [60]	2008	UK	Vicarious and Post-memory Practices in Adopting Families: The Re-production of the Past through	Journal, Focus Groups	Adoption (n=12 adoptive parents)	To explore adoptive parents experience of supporting adoptive children with life story work.

Improving the mental health and mental health support available to adolescents in out-of-home care via Adolescent-Focused Low-Intensity Life Story Work: A realist review

			Photography and Narrative			
Buchanan [36]	2014	UK	The experience of life story work reflections of young people leaving care.	Thesis, semi-structured interviews	Care leavers	To explore care leavers experiences of LSW, highlighting the importance of taking a person-centred approach, trusted relationships and involving birth relatives for successful LSW.
Connor, Sclare, Dunbar and Elliffe [79]	1985	UK	Making a life story book	Journal, Case Study	Residential Placement	To discuss making a life story book with a young person in a residential placement, highlights topics of planning, relationship building and access to supervision.
Cook-Cottone & Beck [19]	2007	USA	A Model for Life-Story Work Facilitating the Construction of Personal Narrative for Foster Children	Journal, Commentary/ practice guidance	Foster placement	To discuss life story work and theoretical frameworks around LSW. Introduces a model for life story work for children in foster care.
Davies & Hodges [76]	2017	UK	Relationship renaissance the use of attachment-based narrative and metaphor in life story work	Journal, Case Study	Foster placement (n=3)	To describe an attachment based narrative approach to LSW presenting two case studies of the approach in practice.
Devenney [90]	2017	UK	Pathway planning with unaccompanied young people leaving care Biographical narratives of past, present, and future	Journal, Interviews	unaccompanied care leavers (n=18) Social work professionals (n=12)	To explore how unaccompanied young people create biographical narratives of their past, present and future.

Improving the mental health and mental health support available to adolescents in out-of-home care via Adolescent-Focused Low-Intensity Life Story Work: A realist review

Eenshuistra, Harder, & Knorth [103]	2019	Netherlands	One size does not fit all A systematic review of training outcomes on residential youth care professionals' skills	Journal, Systematic Review	Residential placement	To review training programmes for residential placement workers.
Eldridge [65]	2018	UK	Confiding in Others: A Qualitative Study Exploring the Experiences of Young people who have been in the Care System	Thesis, semi-structured interviews	Across all settings	To explore factors associated with confiding relationships and what elements are influential on an individual confiding in others.
Ferrier [54]	2011	UK	Life story work in the context of attachment led care planning	Journal, Commentary/ practice guidance	Residential placement	To discuss a model of LSW that uses an attachment-based theoretical framework for carrying out LSW in residential placements.
Finlay [70]	2022	UK	Meaningful natural mentoring relationship characteristics and informal therapeutic life space interactions for youth in care	Thesis, systematic review and interviews	Therapeutic care workers (n=8)	To explore factors that contribute to 'natural mentoring relationships' and trusting relationships with young people with care experience by looking at the perspectives of workers within a residential setting in UK.
Fitzhardinge [81]	2008	Australia	Adoption, resilience and the importance of stories The making of a film about teenage adoptees.	Journal, Commentary/ practice guidance	Adoption (n=6)	To discuss a group project where adopted young people work as a group to make a film about being adopted.

Improving the mental health and mental health support available to adolescents in out-of-home care via Adolescent-Focused Low-Intensity Life Story Work: A realist review

Fostering Sunderland [46]	2021	UK	Foster Carer Handbook	Webpage, Practice guidance	Foster Placement	To provide guidance to foster carers.
Furnivall & Grant [75]	2014	UK	Trauma sensitive practice with children in care	Report		To discuss the importance of social care practitioners being trauma informed to improve their practice when working with young people with care experience.
Gallagher & Green [26]	2012	UK	In, out and after care Young adults' views on their lives, as children, in a therapeutic residential establishment.	Journal, Semi-structured interviews	Residential placement (n=16)	To explore young people with care experience views of their experience in residential care including their experience of LSW.
Gray, Hahn, Cater, Watson, Meineck, & Metcalfe [99]	2019	UK	Trove A Digitally Enhanced Memory Box for Looked after and adopted children	Conference paper, report	Across all settings	To discuss Trove, a digital memory box used with in LSW, it's development, intended purpose and uses.
Gustavsson & MacEachron [52]	2008	USA	Creating Foster Care Youth Biographies A Role for the Internet.	Journal, Commentary/ practice guidance	Across all settings	To describe a digital LSW approach where digital records are made for young people with care experience.
Gutsche [67]	2013	South Africa	Perceptions of social workers regarding life story work with children in child youth centres.	Thesis, semi-structured interviews and focus groups	Residential placement Social workers (n=6)	To explore social workers views in South Africa of LSW with young people living in South Africa's equivalent of residential placements.

Improving the mental health and mental health support available to adolescents in out-of-home care via Adolescent-Focused Low-Intensity Life Story Work: A realist review

Haight, Black, & Sheridan [68]	2010	USA	A Mental Health Intervention for Rural, Foster Children from Methamphetamine-involved Families Experimental Assessment with Qualitative Elaboration	Journal, Randomised	Foster placement (n=23)	To evaluate the efficacy of a LSW intervention delivered by individuals from the community to young people living in foster placements.
Hamilton [64]	2020	UK	Life story approaches and relationships within residential child care A practice reflection. E. Hamilton	Journal, Case Study	Residential placement	To explore the importance of everyday interactions to support life stories for young people within residential care using a case study as an example.
Hammond [37]	2012	UK	Exploring a role for digital technologies in life story work with adolescents in residential care a discourse analysis	Thesis, ethnographic observations, interviews and focus groups	Residential placement (10 young people, 35 carer)	To explore the use of novel digital technologies as tools to facilitate LSW with adolescents in residential placements.
Hammond & Cooper [47]	2013	UK	Digital Life Story Work using technology to help young people make sense of their experience	Book	Across all settings	To discuss digital LSW approaches that can be used in practice with young people with care experience.
Hammond [48]	2016	UK	Digital Life Story Work in Action.	Book chapter	Across all settings	To discuss digital LSW approaches that can be used in practice with young people with care experience.

Improving the mental health and mental health support available to adolescents in out-of-home care via Adolescent-Focused Low-Intensity Life Story Work: A realist review

Hammond, Cooper & Jordan [49]	2021	UK	Mental health, identity and informal education opportunities for adolescents with experience of living in state care: a role for digital storytelling,	Journal, Commentary/ practice guidance	Residential placement (10 young people, 35 carer)	To discuss digital storytelling, how both formal and informal educators can play a role in supporting young people with care experiences use of digital technologies within LSW.
Hanna [77]	2007	USA	Preparing School Age Children for Adoption	Journal, Interviews	Adoption - social care professional s (n=26) and adoptive parents (n=55)	To explore how older children are prepared for adoption, an included topic discussed is life story work/books.
Happer, McCreadie, & Aldgate [86]	2006	UK	Celebrating Success: What Helps Looked After Children Succeed	Book	Across all settings	To explore young people with care experiences views on their successes and the factors that promoted success for them while being looked after by the local authority.
Hertfordshire Children's Specialist and Safeguarding Services [38]	2022	UK	Direct Work and Life Story Work with Children and young people	Webpage, Commentary/ practice guidance	Adoptive and foster placements	To provide guidance on how LSW should be carried out in practice making a distinction between adoptive and foster placements and how these different contexts impact LSW.
Hills [57]	2022	UK	Ensuring life stories are at the centre of direct work	Podcast	Across all settings	To discuss a model of practice that ensures LSW is woven into all interactions with young people in contact with social care services.
Holody & Mäher [39]	1996	USA	Using lifebooks with children in family foster care a here	Journal, Commentary/ practice guidance	Foster placement	To discuss the 'here and now' model for supporting young people in foster placements with life story books using case examples.

Improving the mental health and mental health support available to adolescents in out-of-home care via Adolescent-Focused Low-Intensity Life Story Work: A realist review

			and now process model			
Hooley [55]	2015	UK	Identifying perspectives on life story work with looked after and adopted children	Thesis, Q-methodology	Clinical psychologists and therapists, social work professionals, foster carers, adoptive parents, care leavers (N= 29)	To explore how different aspects of LSW that are important to different groups of people using Q-Methodology.
Hooley, Stokes & Combes [27]	2016	UK	Life story work with looked after and adopted children how professional training and experience determine perceptions of its value	Journal, mixed methods	Clinical psychologists and therapists, social work professionals, foster carers, adoptive parents, care leavers (N= 29)	To investigate which aspects of LSW are important to different groups of people using Q-Methodology.
Hoyle, Shepherd, Lomas, & Flinn [44]	2020	UK	Recordkeeping and the life long memory and identity needs of care experienced children and young people	Journal, Interviews	N=21 care leavers	To explores record keeping practices in social care settings.

Improving the mental health and mental health support available to adolescents in out-of-home care via Adolescent-Focused Low-Intensity Life Story Work: A realist review

Hughes [72]	2013	UK	Life-story work a journey into a child's world Seen and Heard	Commentary/ practice guidance	Across all settings	To discuss different LSW models using case examples to describe their uses
Humphreys, & Kertesz [102]	2014	Australia	Making Records Meaningful Creating an Identity Resource for young people in care	Journal, Survey and focus groups	Survey n=37 young people with care experience by foster care and residential placement. Focus group n=5 social care workers, n=2 young people current care experience, n=4 care leavers.	To explore record keeping practices in Australia to support identity from key stakeholders.
Känkänen & Bardy [80]	2014	Norway	Life stories and arts in child welfare enriching communication	Commentary/ practice guidance	Residential placement	To discuss the authors experiences of using participatory arts within LSW in residential placements.
Lucas, Matthews, L. Brady, Breguet, & Parson [53]	2020	Australia	Therapeutic-Life-Story-Work-Barwon-Pilot-Evaluation	Interim report	Across all settings	To evaluate the implementation of a therapeutic LSW model in Australia.

Improving the mental health and mental health support available to adolescents in out-of-home care via Adolescent-Focused Low-Intensity Life Story Work: A realist review

Malik [63]	2005	USA	Application of attachment theory for training foster parents a model program	Thesis, semi-structured interviews	Foster carers	To discuss the development of a training programme for foster carers.
Monson [94]	2020	Australia	Promoting mental health in out of home care in Australia	Journal, focus group and interviews	N=14 young people with care experience	To explore the experience of young people with care experience in Australia on what promotes positive mental health.
Neil & Beek [59]	2020	UK	Respecting Children's Relationships and Identities in Adoption.	Book Chapter	Adoption	To discuss how adopted young people can be supported to maintain significant relationships while outlining key principles for supporting transitions for adoptive families, drawing on the “Secure Base Model”.
National Institute for Health and Care Excellence (NICE) [7]	2021	UK	Looked after children and young people NICE guideline 2021	Quality standard	Across all settings	To provide the NICE guidelines for LSW with young people with care experience, including recommendations for how LSW should be delivered and what should be included.
Nicholls [101]	2003	UK	Model answer - Community Care	Commentary/ practice guidance	Across all settings	To discuss a model of LSW that focuses on a collaborative approach to securing memories for young people with care experience.
National Society for the Prevention of Cruelty to Children (NSPCC) [83]	2022	UK	Life Story Work _ NSPCC Learning 2022	Commentary/ practice guidance	Across all settings	To discuss the NSPCC's strengths-based model of LSW that is delivered to young people with care experience.
Nuffield Family	2021	UK	Modernising post adoption contact	Report	Adoption n=80	To discuss findings of a consultation with organisations, birth families, adoptive families,

Improving the mental health and mental health support available to adolescents in out-of-home care via Adolescent-Focused Low-Intensity Life Story Work: A realist review

Justice Observatory [96]			findings from recent consultation			young people, regional and voluntary adoption agencies on post-adoption contact with birth relatives
Pakrosnis, & Cˇepukiene [85]	2011	Lithuani a	Outcomes of Solution-Focused Brief Therapy for Adolescents in Foster Care and Health Care Settings.	Book chapter, Matched pairs design	Foster and health care placement N=139	To evaluate the efficacy of solution focused brief therapy for adolescents in Foster Care and Health Care Settings.
Peake [50]	2009	UK	Life story work A resource for foster carers, residential social workers, adoptive parents, and kinship carers, to support this work.	Booklet	Foster care	To provide guidance for foster carers and social care professionals to carry out LSW with young people with care experience.
Sanders [89]	2020	UK	Care experienced children and young people's mental health	Literature review	Across all settings	To review the factors that impact the mental health of young people with care experience.
Shepard [100]	2022	UK	Good practice in record-keeping in children's social care	Commentary/ practice guidance	Across all settings	To discuss record keeping practices in children's social care including the development of a digital tool for care experienced young people to keep a diary to preserve their memories.
Shotton [51]	2012	UK	"Remember when…" Exploring the experiences of looked after children and their carers in engaging in	Thesis, Semi-structured interviews	Foster placement (N=5 carers, N=4 young people)	To explore the experience of foster carers and looked after children using the memory store approach.

Improving the mental health and mental health support available to adolescents in out-of-home care via Adolescent-Focused Low-Intensity Life Story Work: A realist review

			collaborative reminiscence			
Shotton [40]	2013	UK	Remember when...' exploring the experiences of looked after children and their carers in engaging in collaborative reminiscence	Journal, Semi-structured interviews	Foster placement (N=5 carers, N=4 young people)	To explore carers and young people's experiences of using the memory store approach to LSW using semi-structured interviews and a board game approach using Interpretative Phenomenological Analysis.
Shotton [71]	2010	UK	Telling different stories The experience of foster adoptive carers in carrying out collaborative memory work with children.	Journal, Semi-structured interviews	Adoptive parents and foster carers (n=5)	To explore adoptive and foster carers experience using the memory story approach to LSW.
Steenbakkers, van der Steen, & Grietens [82]	2016	Netherlands	‘To talk or not to talk?’: Foster youth's experiences of sharing stories about their past and being in foster care	Journal, Interviews	Foster placement (n=13)	To explore young people’s experiences of foster care in Netherlands.
Teodorczuk, Guse, & du Plessis [88]	2018	South Africa	The effect of positive psychology interventions on hope and well-being of adolescents living in a child and youth care centre.	Journal, Matched pairs design	Adolescents (n=29)	To investigate the efficacy of a positive psychology intervention on hope and wellbeing of young people with care experience in South Africa.

Improving the mental health and mental health support available to adolescents in out-of-home care via Adolescent-Focused Low-Intensity Life Story Work: A realist review

Together for Children Sunderland [45]	2019	UK	Foster Carer Handbook 2019 together for children Sunderland	Commentary/ practice guidance	Foster care	To give guidance to foster carers.
Walker, & Ryan [97]	2016	UK	Working with black and minority ethnic children	Book chapter	Across all settings	To discuss LSW with black and minority ethnic children.
Walker, & Ryan [98]	2016	UK	Why do life story work?	Book chapter	Across all settings	To discuss why LSW is carried out with young people with care experience.
Ward [78]	2002	UK	Opportunity led work maximising the possibilities for therapeutic communication in everyday interactions.	Commentary/ practice guidance	Across all settings	To discuss the concept of “opportunity led working’, a framework to view everyday interactions as having therapeutic potential.
Watson, Hahn, & Staines [41]	2020	UK	Storying special objects Material culture, narrative identity and life story work for children in care.	Journal, Semi-structured interviews	Social care professionals (n=9)	To explore social care professional’s perspectives of the importance of material objects in LSW to support young people with care experience identity construction.
Watson, Latter, & Bellew [22]	2015	UK	Adopted children and young people's views on their life storybooks The role of narrative in the formation of identities.	Journal, Semi-structured interviews	Adopted young people (n=20)	To explore adopted children and young people’s views on their life story books.

Improving the mental health and mental health support available to adolescents in out-of-home care via Adolescent-Focused Low-Intensity Life Story Work: A realist review

Watson, Meineck & Lancaster [42]	2018	UK	Adopted children's co-production and use of 'trove' (a digitally enhanced memory box) to better understand their care histories through precious objects.	Journal, Semi-structured interviews	Adopted young people (n=10)	To explore adopted young people's experience of trialling the prototype Trove, a digital approach to facilitating LSW.
Watson, Staples, & Riches [61]	2021	UK	'We need to understand what's going on because it's our life' using sandboxing to understand children and young people's everyday conversations about care	Journal, Semi-structured interviews	Care experienced children and young people N = 11 (5 adopted, 6 care leavers).	To explore children and young people's hopes and fears for their everyday conversations with carers about their care experience.
Watson, Latter & Bellew [21]	2015	UK	Adopters' views on their children's life story books.	Journal, Focus groups and interviews	Adopters (n=40)	To explore adopters' views on their children's life story books.
Watts [56]	2021	UK	Relationships, reviews and recording Developing practice for children in care.	Journal, Focus Groups and interviews	Social care professional s (n=42), foster carers (n=10) and young people with care	To explore stakeholders views on reviews and recording practices within social care including The Me and My World model introduced to Brighton and Hove City Council.

Improving the mental health and mental health support available to adolescents in out-of-home care via Adolescent-Focused Low-Intensity Life Story Work: A realist review

					experience (n=4).	
Willis, & Holland [20]	2009	UK	Life story work Reflections on the experience by looked after young people	Journal, Semi-structured interviews	Young people with care experience (n=12)	To explore young people with care experiences views on LSW.
Wood [104]	2019	UK	West Sussex Children's improvement board. Performance report against the practice improvement plan improving quality of life story work.	Report	Across all settings	To report findings from an audit of West Sussex children's services LSW practice.
Wood, & Selwyn [74]	2017	UK	Looked after children and young people’s views on what matters to their subjective well-being.	Journal, Focus groups	Young people with care experience (n=140)	To explore and identify the factors that children and young people identify as being indicative of subjective wellbeing.
