# Supplementary material for: Improving the mental health and mental health support available to adolescents in out-of-home care via Adolescent-Focused Low-Intensity Life Story Work: a realist review
Source: BMJ Open. 2023 Oct 9;13(10):e075093. doi: 10.1136/bmjopen-2023-075093 (PMC10565277; doi:10.1136/bmjopen-2023-075093)
Supplement: Supplementary data [file bmjopen-2023-075093supp003.pdf]

Improving the mental health and mental health support available to adolescents in out-of-home care via Adolescent-Focused Low-Intensity Life Story Work: A realist review

Supplementary File 3: Initial practice guidelines

Initial practice guidance recommendation
**1. Adolescent-Focused Low-Intensity Life Story Work should be flexible and person-centred** to ensure how, why, when, by whom and to what extent Adolescent-Focused Low-Intensity LSW is delivered, fits the needs of the individual adolescent and their circumstances.
**2. There is no 'bad time' to start Adolescent-Focused Low-Intensity Life Story Work so this should start early and make use of everyday opportunities.** Everyday opportunities should be used to engage the adolescent in preserving and reflecting on their life experiences.
**3. Adolescent-Focused Low-Intensity Life Story Work should begin in the present day as this grows trust in caring adult(s) and relationships.** Sharing everyday life experiences helps to establish trusting relationships with caring adult(s).
**4. Adolescent-Focused Low-Intensity Life Story Work should involve co-construction of narratives because this improves coherence and authenticity.** Adolescents should be supported to construct a narrative that makes sense and feels authentic to them.
**5. Adolescents should be supported to control how their lives are recorded and preserved in Adolescent-Focused Low-Intensity Life Story Work whenever and wherever possible.** Adolescents should control the processes involved so they feel ownership and power over their own story and how and what is captured.
**5.1 Adolescents should be supported to control the processes for recording and preserving**
**5.2 Adolescents should be supported to control Adolescent-Focused Low-Intensity Life Story Work products (e.g., artefacts)**
**6. Adolescent-Focused Low-Intensity Life Story Work should provide adolescents with somewhere to begin future telling,** supporting adolescents' communication about their lived experiences by providing storytelling prompts, helping adolescents through transitions.
**7. Adolescent-Focused Low-Intensity Life Story Work should record positive aspects of everyday experiences**, there should be **a** focus on the positives in adolescents lives to promote the development of positive identity and positive future expectations.
**8 Adolescent-Focused Low-Intensity Life Story Work should include support for adolescents, caring adults and others involved in supporting adolescents** with life story work should be adequately prepared and/or trained and supported themselves.

Hammond SP, *et al. BMJ Open* 2023; 13:e075093. doi: 10.1136/bmjopen-2023-075093
