# Supplementary material for: Improving the mental health and mental health support available to adolescents in out-of-home care via Adolescent-Focused Low-Intensity Life Story Work: a realist review
Source: BMJ Open. 2023 Oct 9;13(10):e075093. doi: 10.1136/bmjopen-2023-075093 (PMC10565277; doi:10.1136/bmjopen-2023-075093)
Supplement: Supplementary data [file bmjopen-2023-075093supp004.pdf]

Improving the mental health and mental health support available to adolescents in out-of-home care via Adolescent-Focused Low-Intensity Life Story Work: A realist review

Supplementary File 4: Detailed summary of the CMOCs developed and underpinning data

CMOC Label	CMOC	Supporting evidence	Example extracts
**CMOC1**	When low-intensity LSW is flexible and person-centred (C) better practice is achieved (O) because adaptions have been made relating to who, when, where, in what circumstances and how low intensity LSW is delivered to the adolescent (M).	(Buchanan, 2014) (Hammond, 2012) (HCC, 2022) (Holody & Mäher, 1996)<br>(Hooley et al., 2016) (NICE, 2021) (Shotton, 2013) (Watson, Hahn, et al., 2020) (Watson et al., 2018) (Willis & Holland, 2009)	'Take a flexible approach to life story work, and tailor it to the developmental age and needs of the looked-after child or young person' (NICE, 2021)<br><br>'The content of the LSW reported as being most helpful incorporated photos, drawings, speech bubbles, sentimental objects and opportunities for the young people to comment on their feelings and to be creative in the content. This type of child-led approach offered choice with respect to both content and format' (Buchanan, 2014)<br><br>'The findings suggest that it may not be important what form the work takes, as long as it is adapted to suit the child's interests and needs.' (Willis & Holland, 2009)
**CMOC2**	When everyday life experiences are preserved (C) it improves young people's ability to construct a coherent identity (O) because they have autobiographical memory cues available to re-visit (M).	(Atwool, 2017) (Buchanan, 2014) (Cook-Cottone & Beck, 2007) (Hammond, 2016) (Hammond et al., 2021) (Shotton, 2013) (Watson, Hahn, et al., 2020) (Peake, 2009) (Shotton, 2012) (Willis & Holland, 2009) (Gustavsson & MacEachron, 2008) (Hooley, 2015)	'A tangible and visual record of the child's time in care maintained from the outset through the use of memory boxes and life story books is essential to ensure access to a coherent narrative.' (Watson, Hahn, et al., 2020)

Hammond SP, *et al. BMJ Open* 2023; 13:e075093. doi: 10.1136/bmjopen-2023-075093

Improving the mental health and mental health support available to adolescents in out-of-home care via Adolescent-Focused Low-Intensity Life Story Work: A realist review

			'Entries assist in helping the youth preserve memories. These entries can consist of a description of a birthday party, or a school event in which the youth played a role such as a play or sporting event. These stories may someday help the youth establish a stronger sense of self based on positive memories of their past.' (Gustavsson & MacEachron, 2008)
**CMOC3**	When significant events are preserved through recording and made available to an adolescent when they have contact with social care (C) this helps an adolescent construct a coherent understanding of their experiences (O) because memories are available to revisit and reflect upon (M).	(Hills, 2022) (Watson et al., 2015b) (Hoyle et al., 2020) (Together for Children Sunderland, 2019) (Sunderland, 2021) (Hammond, 2012) (Hammond & Cooper, 2013)	'Life Story Work is very important to the child and will help them in childhood and in later life gain some understanding of their identity and background and help them understand and recall their life in care. As the child's carer it is essential that you keep items for the child that can later be used for life story work, such as mementos of special occasions, photographs (including school photos) special clothes or toys.' (Together for Children Sunderland, 2019)
**CMOC4**	When everyday life experiences are preserved with narrative accounts (C) the ability to construct a coherent identity improves (O) because of the availability of contextual information (M).	(Watson et al., 2015a) (Atwool, 2017; Lucas et al., 2020)	'For many of the children the absence of a "story" was a source of criticism about their own book and we were regularly told about books that contained photographs but with little account of who was in the photos or how the photos contributed to the child's story.' (Watson et al., 2015a)<br><br>'The children appeared overall to value their books even though they could see the flaws

Improving the mental health and mental health support available to adolescents in out-of-home care via Adolescent-Focused Low-Intensity Life Story Work: A realist review

			and were critical of these. A lack of narrative and the failure to capture multiple perspectives was a common theme.' (Atwool, 2017)
**CMOC5**	If adolescents are not ready to engage in low-intensity LSW, caring adults in the adolescent's support network should begin to collect and preserve everyday life experiences (C) this ensures adolescents have access to information about their care journey (O) because information is not lost or forgotten (M).	(HCC, 2022) (Ferrier, 2011) (Willis & Holland, 2009) (Cook-Cottone & Beck, 2007) (Atwool, 2017) (Hooley et al., 2016) (Watts, 2021) (Peake, 2009) (NICE, 2021) (Shotton, 2012) (Shotton, 2013)	'The gaps in records that several young people had, including from some foster placements, reinforce the importance of gathering photographs and other mementos at each stage of a child's life, even if they do not want them immediately.' (Willis & Holland, 2009)<br><br>Start life story work as soon as possible after the looked-after child or young person enters care, to support care placement and emotional stability, rather than as an intervention to deliver once placements are stable.' (NICE, 2021)<br><br>'Some children may not feel motivated to engage with the approach, though even if they show little or no interest the carer should continue to collect memories of the child's time with them so that memories of that placement are safeguarded… Knowing that those memories have been safeguarded communicates to the child that their time is important and that they matter.' (Shotton, 2012)

Improving the mental health and mental health support available to adolescents in out-of-home care via Adolescent-Focused Low-Intensity Life Story Work: A realist review

**CMOC6**	When all caring adults within the adolescent's support network are actively involved in preserving memories and artefacts of everyday life experiences (C) it reduces the risk of there being gaps in the adolescent's autobiographical memory (O) because information is not lost (M).	(HCC, 2022) (Hills, 2022) (Atwool, 2017) (Bolton, 2022) (Watson, Hahn, et al., 2020) (Gustavsson & MacEachron, 2008) (Hammond, 2012) (Hammond et al., 2021) (Neil & Beek, 2020) (Brookfield et al., 2008)	'Birth families, all workers involved in the child's journey should take responsibility and have a role in collecting memorabilia of all significant events/achievements for the child. This information should be recorded for the child, and any memorabilia given to the child (or held in safekeeping by the carers according to the child's age and understanding).' (HCC, 2022)<br><br>'All caregivers (including those offering short-term care) need to be equipped to support children in keeping an ongoing record of their time in care through the use of memory boxes, life story books and digital records.' (Atwool, 2017)
**CMOC7**	When adolescents do not have access to information about their everyday life experiences and care journey (C) they can become frustrated and angry (O) because they feel a lack of control over information about their life and their experiences (M).	(Hoyle et al., 2020) (Buchanan, 2014) (Watson, Staples, et al., 2020)	'Their wanting information and clarity about their life was often exacerbated by their judgement that others knew more about their lives and families than they did. This was a source of frustration and anger for many.' (Buchanan, 2014)<br><br>'The common belief that others knew more than they did was often associated with a general feeling that the information others held was deliberately kept from them. A sense of information being kept secret, hidden or confused was evident, which understandably led to many feeling frustrated,

Improving the mental health and mental health support available to adolescents in out-of-home care via Adolescent-Focused Low-Intensity Life Story Work: A realist review

			angry and mistrustful of others' (Buchanan, 2014)
**CMOC8**	When caring adults and adolescents share, preserve, and reflect on everyday life experiences (C) connection and rapport between them increases (O) because they spend quality one to one time together (M).	(Hammond et al., 2021) (Braiden, 2016) (Shotton, 2012) (Holody & Mäher, 1996) (Hooley, 2015) (NICE, 2021) (Shotton, 2012) (Malik, 2005) (Buchanan, 2014) (Watson, Staples, et al., 2020) (Hamilton, 2020) (Hooley et al., 2016) (Eldridge, 2018) (Shotton, 2013) (Aventin et al., 2014)	'All the carers talked positively about how the approach had been good for helping them to spend quality time with the child. The carers felt closer to the child through using it, and thought that the child felt closer to them as well.' (Shotton, 2012)<br><br>'Life story work has the potential for building relationships (for example, by sharing joint activities).' (NICE, 2021)<br><br>'While it may be time consuming, it can also be very rewarding such that in the process of creating this narrative and recording these memories together, a special relationship develops whereby both the child and foster parent learn more about the foster child.' (Malik, 2005)
**CMOC9**	When caring adults and adolescents share, preserve and reflect on everyday life experiences (C) adolescent's self-worth increases (O) because the caring adult shows they want to spend time with them, and their experiences are valuable (M).	(Malik, 2005) (Shotton, 2012) (Shotton, 2013) (Watts, 2021) (Peake, 2009) (Hooley, 2015)	'By taking the time to complete this project, the caregiver conveys to the child that he or she is important and worth the time to get to know.' (Malik, 2005)<br><br>'Evidence within the store of children's particular preferences helps them to see that their opinions matter and are worth recording and talking about, thus contributing to their

Improving the mental health and mental health support available to adolescents in out-of-home care via Adolescent-Focused Low-Intensity Life Story Work: A realist review

			sense of identity and self-worth' (Shotton, 2013)
**CMOC10**	When caring adults and adolescents share, preserve, and reflect on everyday life experiences (C) it helps to establish a safe relationship for the exploration of thoughts and feelings (O) because they establish a point of connection, rapport, and trust (M).	(Aventin et al., 2014) (Gutsche, 2013) (Hammond et al., 2021) (Haight et al., 2010) (Watson, Staples, et al., 2020) (Shotton, 2012) (Finlay, 2022) (Hamilton, 2020) (Holody & Mäher, 1996) (Shotton, 2013) (Hooley, 2015) (Hamilton, 2020) (Willis & Holland, 2009) (Buchanan, 2014)	'The participants claimed that often the children preferred drawing or doing arts and crafts to talking, for example, and this would sometimes prompt them to talk more as they built their confidence in their therapeutic relationship with the social worker.' (Gutsche, 2013)<br><br>'As in conventional LSW with younger children with care-experience, the trusted adult relationship context and engagement with reflective activities created opportunities for a therapeutic alliance between adolescent participants and the first author. In this space, adolescent participants were able to express and begin to reflect on difficult events in their lives' (Hammond et al., 2021)
**CMOC11**	When adolescents' everyday life experiences are recorded (C) they can share it with others in the future (O) because prompts from events have been saved (M).	(Shotton, 2012) (Shotton, 2013) (Watson, Hahn, et al., 2020) (Hammond, 2012) (Atwool, 2017) (Hanna, 2007)	'In looking through the store both carers and children were able to flesh out each memory with details of when it occurred or who else was there. It was clear that the pictorial information was very powerful in taking the participants back to the time and place when it occurred.' (Shotton, 2013)<br><br>'Importantly, children's 'stuff' can be used to help the child and new family to understand their journey' (Watson, Hahn, et al., 2020)

Hammond SP, *et al. BMJ Open* 2023; 13:e075093. doi: 10.1136/bmjopen-2023-075093

Improving the mental health and mental health support available to adolescents in out-of-home care via Adolescent-Focused Low-Intensity Life Story Work: A realist review

**CMOC12**	Sharing, preserving, and reflecting on everyday life experiences with a caring adult (C) increases an adolescent's sense of connectedness and understanding of themselves and others (O) because it provides opportunities to talk and be listened to (M)	(Shotton, 2010) (Watts, 2021) (Eldridge, 2018) (Shotton, 2012) (Watson, Staples, et al., 2020) (Hamilton, 2020) (Shotton, 2013) (Känkänen & Bardy, 2014) (Malik, 2005) (Hughes, 2013) (Gutsche, 2013) (BASW, 2020)	It often opened up lines of communication, sometimes leading to conversations, where other positive memories were triggered or where the child felt able to talk about sensitive issues.' (Shotton, 2010)<br><br>'Where adopted CYP were able to discuss their life stories with their parents in nonthreatening, everyday ways, feelings of relief and connection with parents were expressed.' (Watson, Staples, et al., 2020)
**CMOC13**	When caring adults are prepared to share personal information with the adolescent they are caring for (C) the adolescent is more willing to confide with their caring adult (O) because trust is established (M).	(Eldridge, 2018) (Watson, Staples, et al., 2020)	'Furthermore, in the current study, limited reciprocal confiding and self-disclosure appeared to help equalise power dynamics and facilitate genuine confiding across personal and professional relationships.' (Eldridge, 2018)
**CMOC14**	When adolescents have consistent and repeated positive engagements with caring adults who support them to preserve and reflect on everyday life experiences (C) then they may start to build trust in others (O) because it challenges their previously held negative relational beliefs (M).	(Finlay, 2022) (Eldridge, 2018) (Wood & Selwyn, 2017) (Atwool, 2017) (Hamilton, 2020) (Hooley et al., 2016) (Shotton, 2012) (Furnivall & Grant, 2014)	'Reciprocity, understanding, acceptance and perseverance all appeared to be important for the emergence of sufficient trust to confide. For some young people, trust developed in one relationship then appeared to generalise into other relationships' (Eldridge, 2018)<br><br>'Such work also enables the child to share their story, has the potential to increase self-esteem and, by building a sense of trust with the social worker and caregivers, may facilitate the development of secure

Improving the mental health and mental health support available to adolescents in out-of-home care via Adolescent-Focused Low-Intensity Life Story Work: A realist review

			attachment in new situations.' (Atwool, 2017)
**CMOC15**	When a caring adult provides consistent support to an adolescent to discuss their thoughts and feelings in response to everyday life experiences (C) the adolescent comes to understand their experiences and themselves better, including their emotional and behavioural responses to experiences (O) because they develop reflective and reminiscence skills through shared story telling (M).	(Ward, 2002) (Hamilton, 2020) (Hooley et al., 2016) (Hooley, 2015) (Fitzhardinge, 2008) (Steenbakkers et al., 2016) (Watson et al., 2018) (Haight et al., 2010) (Holody & Mäher, 1996) (Watson et al., 2015b) (Malik, 2005) (Shotton, 2013) (Ferrier, 2011)	'Reflective function can be encouraged by bringing into consciousness a range of possible interpretations or ways of experiencing similar stories. It has been argued that both reflective function and neural integration develop only in a context of something akin to a secure attachment relationship.' (Fitzhardinge, 2008)<br><br>"This life book focuses on the here and now and makes use of the relationship between the worker and the child. While not specifically therapeutic in its goals, it seeks to help the child make connections between his or her present experiences, emotional needs, and past events." (Holody & Mäher, 1996)<br><br>'In this model, the key aims of the LSW are to integrate a child's internal and external experiences by collaboratively constructing a narrative.' (Hooley et al., 2016)
**CMOC16**	When a caring adult provides consistent support to an adolescent to discuss their thoughts and feelings in response to everyday life experiences (C) they experience better mental	(NSPCC, 2022) (Hooley et al., 2016) (Willis & Holland, 2009) (Känkänen & Bardy, 2014) (Steenbakkers et al., 2016) (Hills, 2022) (Furnivall & Grant, 2014) (Aventin et al., 2014) (Holody & Mäher, 1996) (Eldridge, 2018)	'A universal viewpoint of exploring and managing emotions emerged, clearly suggesting the importance of helping children to identify, express and regulate emotions during their involvement' (Hooley et al., 2016)<br><br>'respondents indicated that good life story

Improving the mental health and mental health support available to adolescents in out-of-home care via Adolescent-Focused Low-Intensity Life Story Work: A realist review

	health (O) because they learn emotion regulation skills (M).		work should involve helping a child to express and manage emotions that arise during the work.' (Hooley et al., 2016)
**CMOC17**	When caring adults consistently support an adolescent to preserve and reflect on everyday life experiences (C) it strengthens the relationship between them (O) because the caring adult becomes better attuned to understanding the adolescent and their support needs (M).	(Davies & Hodges, 2017) (Malik, 2005) (Shotton, 2012) (Holody & Mäher, 1996) (Atwool, 2017) (Hooley, 2015) (Hanna, 2007)	'But by this stage, the carer was more attuned to Katie's particular experiences and was able to demonstrate the necessary acceptance, understanding, commitment and unconditional love for Katie that soothed and ameliorated her anxieties'. (Davies & Hodges, 2017)<br><br>'it gives the worker information critical to understanding and linking the child's present functioning and coping patterns with previous events in the child's life.' (Holody & Mäher, 1996)<br><br>'The here-and-now approach allowed Helen's mother to accept her child's thoughts and feelings as the girl's own perception and understanding of her life.' (Holody & Mäher, 1996)
**CMOC18**	When caring adults view everyday interactions with an adolescent as providing reflective opportunities (C) their relationship is strengthened (O) because adolescents are more comfortable	(Ward, 2002) (Finlay, 2022) (Hammond, 2016) (Connor et al., 1985) (Hamilton, 2020) (Aventin et al., 2014) (Känkänen & Bardy, 2014)	'The keyworker also had to find time for spontaneous 'out of hours' sessions, such as helping Ben write short stories about his temper tantrums after they happened.' (Connor et al., 1985)<br><br>'You can use your very being to embrace the

Improving the mental health and mental health support available to adolescents in out-of-home care via Adolescent-Focused Low-Intensity Life Story Work: A realist review

	talking and reflecting on everyday events (M).		relationships and create key opportunities for exploring each life story. 'Don't wait for the perfect moment, take the moment and make it perfect' (Anonymous).' (Hamilton, 2020)
**CMOC19**	When low-intensity LSW takes place in emotionally meaningful places (C) richer narratives can be constructed (O) because of the availability of contextual cues (M).	(Hills, 2022) (Haight et al., 2010) (Hammond, 2012) (Hamilton, 2020) (Watson, Staples, et al., 2020)	'Providing an intervention to children within a context familiar and emotionally meaningful to them has a variety of other benefits as well. When the clinician travels to see them individually and on their own turf, children can feel valued and cared for, and relationship building may be enhanced. In addition, the community context is rich in cues for children to spontaneously initiate personal narratives.' (Haight et al., 2010)
**CMOC20**	When everyday life experiences are preserved using temporal anchor points and reflected on at regular intervals (C) it aids the ease of storytelling (O) because it is easier to follow (M).	(Hammond et al., 2021) (Hammond, 2012) (Gustavsson & MacEachron, 2008) (NICE, 2021)	'My regular weekly visits also provided young people with a frame of reference from which to begin to construct and reflect upon recent events in a temporal fashion.' (Hammond, 2012)<br><br>'Schedule regular, dedicated times for life story work to help the looked-after child or young person make sense of their journey through the care system and beyond, their significant relationships and their identity.' (NICE, 2021)

Improving the mental health and mental health support available to adolescents in out-of-home care via Adolescent-Focused Low-Intensity Life Story Work: A realist review

**CMOC21**	When everyday life experiences are preserved and reflected on at regular intervals (C) adolescents become more comfortable engaging in the process (O) because the approach becomes familiar and predictable (M).	(Holody & Mäher, 1996) (Hamilton, 2020) (Shotton, 2012)	'The here-and-now lifebook is more user-friendly than the traditional lifebook, not only for the child but for the worker as well. If it is used in every contact between worker and child, it is transformed from something extra into the very forum for interaction.'(Holody & Mäher, 1996)
**CMOC22**	When a caring adult regularly demonstrates how to respond to challenging situations in constructive ways (C) the adolescent learns how to respond to challenging situations (O) because of role modelling (M).	(Furnivall & Grant, 2014) (Hammond & Cooper, 2013) (Hammond, 2016) (Finlay, 2022) (Steenbakkers et al., 2016)	Developing the capacity to self-regulate and become accountable requires safe, positive relationships with adults who can scaffold children's' learning (Furnivall & Grant, 2014)<br><br>'This person also needs to be a role model, particularly when handling the inherent frustrations of working with digital media in innovative ways and helping to mediate the risks that digital media may bring (discussed later in this chapter).' (Hammond, 2016)<br><br>'In addition, negative experiences with (previous) attachment figures make sharing distressing memories more difficult for adolescents in family foster care, for example when these attachment figures insufficiently regulated the emotional aspects of distressing events for them, or insufficiently scaffolded them in constructing these memories' (Steenbakkers et al., 2016)

Hammond SP, et al. BMJ Open 2023; 13:e075093. doi: 10.1136/bmjopen-2023-075093

Improving the mental health and mental health support available to adolescents in out-of-home care via Adolescent-Focused Low-Intensity Life Story Work: A realist review

**CMOC23**	When a caring adult consistently and sensitively helps an adolescent curate, develop, and reflect upon narratives about their lived experiences (C) the narratives become authentic accounts that resonate with the adolescent's experience (O) because they were actively involved in the construction of accounts (M)	(Atwool, 2017) (Baynes, 2008) (Watson, Hahn, et al., 2020) (Watson, Staples, et al., 2020)	'This will help to ensure that the written story reflects the child's experiences, helping to create a coherent narrative of events, feelings and memories. Without this, we risk creating a life story book that has little meaning to the child,' (Baynes, 2008)
**CMOC24**	When adolescents are not actively involved in the construction of narratives about their lived experience (C) they can struggle to accept the narratives as their own story (O) because they do not resonate with the young persons lived experience (M).	(Baynes, 2008) (Watson, Hahn, et al., 2020) (Gallagher & Green, 2012) (Atwool, 2017)	'Children may feel that their life story book lacks a coherent narrative or presents a narrative with which they disagree (Watson et al., 2015a). This can lead to dissonance between their identity and the identity presented in the book, leading children to believe that including multiple perspectives would be beneficial (Watson et al., 2015a).' (Watson, Hahn, et al., 2020)<br><br>'All proponents of life story work emphasise the importance of working with the child and allowing them to give voice to their subjective experience. Kagan (2014) warns of the dangers of practitioners writing life stories for children, arguing that their validity is likely to be questioned because they do not fully capture the child's experience or perspective and traumatic events may be glossed over.'

Improving the mental health and mental health support available to adolescents in out-of-home care via Adolescent-Focused Low-Intensity Life Story Work: A realist review

			(Atwool, 2017)
**CMOC25**	When caring adults do not consistently and sensitively help adolescents curate, develop, and reflect upon narratives about their lived experiences (C) identity development can be negatively impacted (O) because the adolescent constructs unnecessarily negative narrative accounts (M).	(Watson, Hahn, et al., 2020) (Shotton, 2012) (Atwool, 2017) (Watson, Staples, et al., 2020)	'Granting children control over narratives aids identity formation but left unchallenged their perspective of events may also become one-sided and inaccurate' (Watson, Hahn, et al., 2020)
**CMOC26**	When a caring adult supports an adolescent to construct narrative accounts of their experience using alternative information or interpretations (C), less negative narratives may result (O), because they have more perspectives to draw on (M)	(Fitzhardinge, 2008) (Shotton, 2012) (Davies & Hodges, 2017) (Pakrosnis & Cˇepukiene, 2011) (Hooley et al., 2016) (Baynes, 2008) (Shotton, 2013) (Gallagher and Green, 2012) (Shotton, 2010) (Willis and Holland, 2009)	'Narratives were enriched by bringing into consciousness some alternative interpretations and accommodating in a deeper way the perspectives of different people within the story.' (Fitzhardinge, 2008)<br><br>'From a narrative perspective it can help the child to thicken positive counter-narratives impacting on the dominant stories the child has and is able to tell about themselves, particularly thickening stories around their strengths, worth and belonging' (Shotton, 2012)<br><br>Participants also highlighted the importance of finding out what the events mean to the child and offering alternative narratives:' (Hooley et al., 2016)
**CMOC27**	When a caring adult consistently and sensitively helps an	(Connor et al., 1985) (Hammond & Cooper, 2013) (Finlay, 2022)	The therapeutic process involves creating opportunities to open up conversational

Improving the mental health and mental health support available to adolescents in out-of-home care via Adolescent-Focused Low-Intensity Life Story Work: A realist review

	adolescent curate, develop, and reflect upon narratives about their lived experiences (C) positive narratives including liberating narratives can be constructed (O) because caring adults can help adolescents to re-script negative narrative accounts (M).	(Hammond, 2016) (Hammond, 2012) (Fitzhardinge, 2008) (Gutsche, 2013) (Happer et al., 2006) (Hooley, 2015) (Känkänen & Bardy, 2014) (Cook-Cottone & Beck, 2007) (Watson et al., 2015b) (Holody & Mäher, 1996) (Shotton, 2013) (Hooley et al., 2016)	spaces. These spaces provide young people with opportunities to share accounts of their own lives, in their own words. These accounts are listened to by a consistently available adult who can sensitively support the young person's reflections on the stories they choose to share, and potentially, provide re-scripting or re-editing advice.' (Hammond, 2016).<br><br>'Importantly, these reflections must eradicate self-blame and encourage young people to look forward positively towards their futures. This phase is about encouraging young people to recognise their own resilience, effectively turning 'if only this hadn't happened' into 'despite all these things that have happened to me'. (Hammond & Cooper, 2013)
**CMOC28**	When significant individuals provide alternative positively framed narratives to the adolescent (C) it helps to increase their self-esteem and ability to develop positive future expectations (O) because they become increasingly aware of their positive characteristics and abilities (M).	(Shotton, 2012) (Aust, 1981) (Hooley et al., 2016) (Teodorczuk et al., 2018) (Eldridge, 2018) (Happer et al., 2006) (Sanders, 2020) (Devenney, 2017)	'From a narrative perspective it can help the child to thicken positive counter-narratives impacting on the dominant stories the child has and is able to tell about themselves, particularly thickening stories around their strengths, worth and belonging.' (Shotton, 2012)<br><br>'The co-construction of stories around their achievements and certificates contributes to

Improving the mental health and mental health support available to adolescents in out-of-home care via Adolescent-Focused Low-Intensity Life Story Work: A realist review

			their perception of themselves as someone who can achieve. As they reflect with their carer and look together at their writing and spelling they are able to co-construct stories about their progress over time and see themselves as someone who can make progress, who can grow and change in positive ways.' (Shotton, 2012)
**CMOC29**	When significant individuals are given the opportunity to offer their narrative account of adolescent's life experiences (C) adolescents become aware of alternative interpretations of life events (O) because they have access to different narrative accounts (M).	(Bazalgette, 2015) (HCC, 2022) (Hills, 2022) (Beste & Richardson, 1981) (Baynes, 2008) (Peake, 2009) (Ferrier, 2011) (BASW, 2020) (ARCBOX, 2022) (Fitzhardinge, 2008) (Watson, Hahn, et al., 2020) (Hoyle et al., 2020) (Watson et al., 2015a) (Watts, 2021)	'Fourth, Life Story Books that include contributions from more than one source, such as foster parents, social worker, and parents, will allow children to have a broader understanding of their life events. It will also increase the child's awareness that different people see the same situation differently.' (Beste & Richardson, 1981)<br><br>'Children were consistently clear that the narrative presented in their book should be of their biography, representing multiple actor viewpoints including those of their birth family.' (Watson et al., 2015a)<br><br>'In addition, a life story book that contains many voices is likely to be seen by the child as having more credibility in the future'' (Baynes, 2008)
**CMOC30**	When significant individuals including birth relatives are	(Cook-Cottone & Beck, 2007) (Shotton, 2012) (Buchanan, 2014)	''The life-story work can also be used as a way to structure visits with biological parents

Improving the mental health and mental health support available to adolescents in out-of-home care via Adolescent-Focused Low-Intensity Life Story Work: A realist review

	positively involved in Low intensity LSW (C) stronger relationships can be established between those involved (O) because of the interactions facilitated through the activity (M).	(Watson, Staples, et al., 2020) (Hooley, 2015) (Shotton, 2012)	and siblings. The visit time can be used to collect artifacts (take photos or share old photos) and record related information (e.g. write letters, create a memory page for photos, tell stories about the photos).' (Cook-Cottone & Beck, 2007)<br><br>'facilitating interaction with other family members, bringing the family closer together, helping other family members feel involved in the care of the child' (Shotton, 2012)
**CMOC31**	When significant individuals including birth relatives are involved in low intensity LSW (C) identity development can be supported (O) because significant individuals can share information about the adolescent's family heritage (M).	(Monson et al., 2020) (Neil & Beek, 2020) (Buchanan, 2014) (Hoyle et al., 2020) (Sanders, 2020)	'For young people, carers and workers could also be gatekeepers for connectedness with cultural heritage and identity. One young person described what he believed was a turning point in a relationship with a carer, when the carer took him to a restaurant serving the cuisine of the young person's country of origin' (Monson et al., 2020)<br><br>'Staying in touch with parents, siblings and other important people helps children develop a sense of identity and belonging and promotes healthy and stable relationships,'(Sanders, 2020)
**CMOC32**	When adolescents exert a choice over how low intensity LSW everyday life experiences are preserved and reflected on (C) they experience ownership over the process and resulting	(Hooley, 2015) (Watson et al., 2018) (Buchanan, 2014) (Hammond & Cooper, 2013) (Watson, Hahn, et al., 2020) (Holody & Mäher, 1996) (NICE, 2021) (HCC, 2022) (Hammond, 2012) (Peake, 2009)	'Children can have ownership of their story work via choosing which objects to story/not story and by dictating the pace at which the work progresses:<br>I think it's a great concept and would be a good idea for children to have ownership of

Improving the mental health and mental health support available to adolescents in out-of-home care via Adolescent-Focused Low-Intensity Life Story Work: A realist review

	products (O) because they feel valued their perspectives and opinions are valued.	(Gutsche, 2013) (Buchanan, 2014) (Lucas et al., 2020) (Baynes, 2008)	their stories and memories. (FSW, Ben)' (Watson, Hahn, et al., 2020)<br><br>'A feeling of ownership appeared to be fostered through the use of a person-centred and flexible approach to LSW.' (Buchanan, 2014)
**CMOC33**	When adolescents are given agency to choose the mediums (digital, paints, puppets, physical/digital 'visits' music etc) used to collect and conserve everyday memories (C) it is engaging to them (O) because they have a sense of control, ownership and power over the process, (M).	(Watson, Hahn, et al., 2020) (Hammond, 2012) (Hammond, 2016) (Watson et al., 2015a) (Aventin et al., 2014) (Hills, 2022) (Buchanan, 2014) (Holody & Mäher, 1996) (Hammond & Cooper, 2013) (Nuffield Family Justice Observatory, 2021) (Baynes, 2008) (Hooley et al., 2016) (Hammond et al., 2021) (Lucas et al., 2020) (Peake, 2009) (Willis & Holland, 2009) (Känkänen & Bardy, 2014) (Gutsche, 2013) (Hammond & Cooper, 2013)	'trove aims to support children to keep their own record of their life, through interactive child-driven technology, to give them some control of their life story.' (Watson, Hahn, et al., 2020)<br><br>'…how we can support that so it feels like their story rather than produced in a format that somebody has given them. We want to make sure that for young people, their stories are owned by them, and that means having them in a format that they would choose.' (Hills, 2022)
**CMOC34**	When caring adults are aware of the adolescent's special educational needs and disabilities (SEND) (C) the adolescent can engage with preserving and reflecting on their experiences (O) because appropriate adaptations	(Hamilton, 2020) (Hammond, 2012) (HCC, 2022) (Atwell, 2016) (NICE, 2021)	'Where the child has some level of learning disability, careful thought will need to be given to the implications this will have on undertaking life story work, considering in particular the most effective methods of communication.' (HCC, 2022)

Improving the mental health and mental health support available to adolescents in out-of-home care via Adolescent-Focused Low-Intensity Life Story Work: A realist review

	can be made to make the activity accessible (M).		'For children with a disability who perhaps have difficulty in communicating by speech, or who may lack the use of one or more of the senses, it is possible to develop life story work that does not depend only on visual methods but involves stimulation of other senses.' (Atwell, 2016)<br><br>'Take a flexible approach to life story work, and tailor it to the developmental age and needs of the looked-after child or young person.' (NICE, 2021)
**CMOC35**	When caring adults from the adolescent's support network have training materials available to them (C) adolescents with SEND can engage with preserving and reflecting on their lived experiences (O) because the caring adults in their support network are equipped with the knowledge and skill to make appropriate adaptations (M).	(Atwell, 2016)	'In deciding how to tackle life story work with disabled children, it is also important to consider who would be best able to do this work. Since being able to understand and communicate with the child is paramount, this should be undertaken by the person who communicates best with the child, rather than assume it is always done by the child's social worker.' (Atwell, 2016)
**CMOC36**	When the creation of a product is prioritised over a meaningful process for securing everyday memories (C) the value of narratives contained will be	(Känkänen & Bardy, 2014)<br>(Hammond & Cooper, 2013)<br>(Hooley et al., 2016)<br>(Baynes, 2008) (Hammond, 2012)	'Baynes (2008) continues by suggesting that life story work has been pushed to the periphery of practice within a more quantitatively driven target-focused profession. She fears that this may lead some

Improving the mental health and mental health support available to adolescents in out-of-home care via Adolescent-Focused Low-Intensity Life Story Work: A realist review

	reduced (O) because the product does not represent adolescents lived experience of everyday events (M).		to conceptualise life story work as a product, which could detract from the potentially therapeutic nature of the underlying process.' (Hammond, 2012)<br><br>'They also note that although the process is of primary importance, the material record was also significant.' (Atwool, 2017)
**CMOC37**	When the process of curating, developing, and reflecting upon narratives about lived experiences is prioritised over the recording (C) a bank of future reflective starting points is lost (O) because everyday memories and artefacts are not secured (M).	(Aust, 1981) (Walker & Ryan, 2016a) (Hoyle et al., 2020) (Hooley, 2015) (Watson et al., 2015b) (Hills, 2022) (Watts, 2021) (Buchanan, 2014) (Känkänen & Bardy, 2014) (Atwool, 2017) (Willis & Holland, 2009) (Beste & Richardson, 1981) (Aust, 1981) (Hammond, 2016)	'We never regard the work as finished as life goes on, but some record of the process is important as it provides a reference point, particularly as it can be updated until adulthood.' (Walker & Ryan, 2016a)<br><br>'Material items and the records produced during the work were important to the young people. Many described treasuring them, returning to them often and planning to continue to add to them.' (Buchanan, 2014)<br><br>'Concrete artefacts – such as poems, photographs, paintings, cartoons and so on – cannot be ignored, which also means that unlike an unrecorded spoken narrative, they persist after the initial act of telling and we can return to them again and again.' (Känkänen & Bardy, 2014)
**CMOC38**	When adolescents feel empowered to have editorial	(Baynes, 2008) (Watson, Hahn, et al., 2020) (Watson et al., 2018)	'Children can have ownership of their story work via choosing which objects to story/not

Improving the mental health and mental health support available to adolescents in out-of-home care via Adolescent-Focused Low-Intensity Life Story Work: A realist review

	control over memory product(s) (C) they feel more positive about the product(s) and experience greater ownership of their story/narrative (O) because they have control over how, and which, events are recorded or not (M).	(Hoyle et al., 2020; Watson et al., 2015a) (Buchanan, 2014) (Gallagher & Green, 2012) (Gray et al., 2019)	story and by dictating the pace at which the work progresses' (Watson, Hahn, et al., 2020)<br><br>'In some cases children were dismissive about their book, as the focus did not seem to be on them specifically. For a few children, photos included were stark reminders of differential treatment of siblings by birth parents and contributed to negative feelings.' (Watson et al., 2015a)<br><br>'have stuck a piece of paper over a section about me behaving inappropriately around Rhian and John, my adoptive family that broke down. The thing in the life story book spoilt my memory of Rhian and John's …. It seemed like it was written for somebody younger than me. (Caitlyn, 19)' (Gallagher & Green, 2012)
**CMOC39**	When the medium used for collecting the product is easily editable (e.g., digitally) (C) it helps the contents of low intensity LSW hold meaning over time (O) because it can be updated and edited when needed (M)	(Beste & Richardson, 1981) (Hammond, 2012) (Hammond & Cooper, 2013) (Watson et al., 2015a) (Watson, Latter and Below, 2015) (Atwool, 2017) (Hammond & Cooper, 2013) (Hooley et al., 2016) (Nuffield Family Justice Observatory, 2021)	'Digital tools offer the ability to help young people to express thoughts and feelings which can be continually updated, edited and reflected upon. Using interactive computer-based mediums in this way grants the user flexibility to make changes frequently and easily.' (Hammond & Cooper, 2013)<br><br>'Concerns about the scrapbook style focused on the inability to remove information or to update as the child grew older. ' (Watson,

Improving the mental health and mental health support available to adolescents in out-of-home care via Adolescent-Focused Low-Intensity Life Story Work: A realist review

			Latter and Below, 2015)
**CMOC40**	When digital artefacts/products are backed-up and have access controls (e.g., encrypted and password protected) (C) it helps preserve and keep the contents safe (O) because it is secured and protected from unauthorised access (M).	(Hammond & Cooper, 2013) (Shepard, 2022) (Ferrier, 2011) (Watson et al., 2015a) (Atwool, 2017) (Willis & Holland, 2009) (Gustavsson & MacEachron, 2008) (Beste & Richardson, 1981) (Hammond & Cooper, 2013) (Hammond, 2016) (Ferrier, 2011) (Gray et al., 2019)	'Security mechanisms were posited from both groups, including remotely programmable passcodes or a scannable key.' (Gray et al., 2019)<br><br>'Provision of back-up storage in case records are lost or destroyed is also needed.' (Atwool, 2017)<br><br>'Digital life story work products can be copied easily and, with permission, back-up copies can be stored with young people's files for posterity.' (Hammond, 2016)
**CMOC41**	When a medium for capturing a product enables narratives to be stored in different formats (e.g. voices, videos, pictures, objects) (C), they provide a richer starting point for future retelling (O), because they connect adolescents to their memories through the engaging and tangible nature of the artefacts (M).	(Hammond & Cooper, 2013) (Peake, 2009) (Hills, 2022) (Watson et al., 2015a) (Gray et al., 2019) (Watson et al., 2018) (Shotton, 2012) (Shotton, 2013) (Hammond, 2016) (Buchanan, 2014) (Atwell, 2016) (Hoyle et al., 2020) (Watson, Hahn, et al., 2020)	'The background sounds to everyday life often go unnoticed. Sounds from the environment can help to connect to memories and hearing a specific sound can prompt a memory.' (Hammond & Cooper, 2013)<br><br>'For looked after and adopted children, physical objects are often the only remaining link to their past; a portal to stories of birth families, former homes, and significant people.' (Gray et al., 2019)<br><br>'In looking through the store both carers and children were able to flesh out each memory with details of when it occurred or who else

Improving the mental health and mental health support available to adolescents in out-of-home care via Adolescent-Focused Low-Intensity Life Story Work: A realist review

			was there. It was clear that the pictorial information was very powerful in taking the participants back to the time and place when it occurred.' (Shotton, 2013)
**CMOC42**	When adolescent's communication needs are considered when creating low-intensity LSW products (C) it ensures that memories are accessible for the adolescent to engage with (O) because they can understand the contents (M).	(Watson, Latter & Bellew, 2015) (Hammond, 2012) (Atwell, 2016)	"A lack of appropriate methods of communication in life story work may result in engagement opportunities with adolescents becoming lost and/or overlooked." (Hammond, 2012)<br><br>"…the care system and social work practice itself needs to converse using culturally appropriate methods of communication… ' (Hammond, 2012)<br><br>'For children with a disability who perhaps have difficulty in communicating by speech, or who may lack the use of one or more of the senses, it is possible to develop life story work that does not depend only on visual methods but involves stimulation of other senses.' (Atwell, 2016)
**CMOC43**	When adolescents use LI-LSW products as storytelling prompts to share stories with chosen caring adults (C) it helps to strengthen relationships between adolescents	(Buchanan, 2014) (Shotton, 2012) (Hammond, 2012) (Hamilton, 2020)	'Sharing a moment, an experience together helps to create social connections and helps us understand one another and our thinking.' (Hamilton, 2020)

Improving the mental health and mental health support available to adolescents in out-of-home care via Adolescent-Focused Low-Intensity Life Story Work: A realist review

	and caring adults (O) because caring adults understand adolescents and their experiences (M)		
**CMOC44**	When current everyday life experiences are preserved and reflected on (C) positive self-narratives can become more accessible for the adolescent to make sense of their experiences, construct their identity and develop positive future expectations (O) because the focus is not on past negative experiences (M)	(Shotton, 2013) (Shotton, 2012) (Nicholls, 2003) (Teodorczuk et al., 2018) (Ferrier, 2011) (Haight et al., 2010) (Watson, Staples, et al., 2020) (Steenbakkers et al., 2016) (NSPCC, 2022) (Hammond et al., 2021) (Teodorczuk et al., 2018)	‘Why Life Story Work is Failing…It has the potential to deny the promotion of a child’s identity by focusing on his or her experience as a looked-after child.’ (Nicholls, 2003)<br><br>‘From a narrative perspective it can help the child to thicken positive counter-narratives impacting positively on the dominant stories the child has and is able to tell about themselves, particularly thickening stories around their strengths, worth and belonging.’ (Shotton, 2012)<br><br>‘A key part of Life Story Work is celebrating the child or young person’s achievements. We recognise the resilience they’ve shown by overcoming adversity and help them to see themselves in a positive light.’ (NSPCC, 2022)
**CMOC45**	When everyday life experiences are reflected on between an adolescent and caring adult (C) difficulties and challenges can be positively re-framed (O) because	(Walker & Ryan, 2016a) (Holody & Mäher, 1996) (Buchanan, 2014) (Teodorczuk et al., 2018) (Hooley et al., 2016)	‘Life story books are thought to sometimes silence the difficulties experienced by adoptees by not allowing them enough space for exploring troublesome feelings and fantasies.’ (Walker & Ryan, 2016a)

Improving the mental health and mental health support available to adolescents in out-of-home care via Adolescent-Focused Low-Intensity Life Story Work: A realist review

	opportunities to reflect on positive interpretations are taken (M).		'The statements relating to this all emphasised 'feelings to be shown, managed and normalised'. Participants in all groups agreed with the suggestion that work should not be stopped if difficult feelings came up and that upsetting or traumatic experiences should be explored. They indicated that a balance needed to be achieved that included happy as well as difficult memories.' (Hooley et al., 2016)
**CMOC46**	When low-intensity LSW containing a bank of positive memories and personal achievements is re-visited by an adolescent needing emotional support (C) their wellbeing may improve (O), because they find the contents comforting (M)	(Watts, 2021) (Shotton, 2010) (Buchanan, 2014) (Cook-Cottone & Beck, 2007; Gallagher & Green, 2012) (Shotton, 2012) (Shotton, 2013) (Humphreys & Kertesz, 2014) (Willis & Holland, 2009) (Watts, 2021) (Holody & Mäher, 1996)	'The value of returning to the book was described by the other participants. Some chose to simply look through it to see 'familiar faces' or to remind them of coming through difficult times in the past when faced with new transitions. (Buchanan, 2014)<br><br>'As many of the memories were of happy/positive times, the carers felt that reflecting on the store often had a positive effect on mood for both themselves and the children.' (Shotton, 2012)<br><br>'The carers perceived that using the memory store approach often had a calming effect on the children and that it was also an activity that they seemed to find comforting. (Shotton, 2012)

Hammond SP, *et al. BMJ Open* 2023; 13:e075093. doi: 10.1136/bmjopen-2023-075093

Improving the mental health and mental health support available to adolescents in out-of-home care via Adolescent-Focused Low-Intensity Life Story Work: A realist review

**CMOC47**	When caring adults have an awareness of the adolescent's cultural background (C) it promotes appropriate preservation and reflection of everyday life experiences (O) because caring adults have an increased awareness of the adolescent's individuality and cultural heritage (M).	(Monson et al., 2020) (HCC, 2022) (Walker & Ryan, 2016b) (NICE, 2021)	'It is important to a child that the worker doing life story work has a good grasp of the child's world, both the inner world and external realities. This means the worker must familiarise him or herself with aspects of family life of the ethnic community of the child in the context of this society. This involves getting information from a variety of sources, for example, people from a culture similar to that of the child, and of course the internet.' (Walker & Ryan, 2016b)<br><br>'Ensure that life story work for looked-after children and young people captures and embraces ethnicity, cultural and religious identity, as well as other personal aspects of identity, for example, sexual identity or disabilities.' (NICE, 2021)
**CMOC48**	When institutions consistently communicate and take measures to support the importance of collecting everyday life experiences for adolescents (C) significant individuals are more likely to prioritise this activity (O) because they know what is expected of them (M).	(Finlay, 2022) (Wood, 2019) (Atwool, 2017) (Holody & Mäher, 1996) (Shotton, 2013) (Watts, 2021) (Hooley et al., 2016) (Connor et al., 1985) (Brookfield et al., 2008)	'Recognition of children's entitlement to a coherent narrative needs to be embedded in practice at the micro level. This can only occur if appropriate supports are in place at the organisational level, facilitated by macro-level priority being accorded to provision of resources needed to ensure the best possible outcomes for children and young people in care.' (Atwool, 2017)<br><br>''Policy needs to be developed which specifies that training for foster carers in

Improving the mental health and mental health support available to adolescents in out-of-home care via Adolescent-Focused Low-Intensity Life Story Work: A realist review

			using the approach, or similar, should be part of their induction to fostering. This would raise their awareness and help them feel equipped to use the approach from the start of a child's placement with them.' (Shotton, 2013)
**CMOC49**	When caring adults are provided with relevant ongoing training that highlights the importance of preserving and reflecting on everyday life experiences (C), they are more likely to carry these out (O) because they have the confidence and skills to do so (M).	(Finlay, 2022) (Braiden, 2016) (Walker & Ryan, 2016a) (Eenshuistra et al., 2019) (Hills, 2022) (Haight et al., 2010) (Wood, 2019) (Watson, Staples, et al., 2020) (NICE, 2021) (Shotton, 2012) (Buchanan, 2014) (Holody & Mäher, 1996) (Shotton, 2013) (Atwool, 2017) (Sanders, 2020)	'To embark on this journey with the child, above all you need time, sensitivity, empathy for the child and a commitment to the work. An ability to listen to the child and understand them is paramount; the skills and particular techniques can be learnt' (Walker & Ryan, 2016a)<br><br>'Ensure that the experience and skillset of the practitioner or carer delivering life story work for looked-after children and young people is sufficient to deliver good quality work, particularly in complex situations.' (NICE, 2021)
**CMOC50**	When peers with similar lived experiences introduce low-intensity LSW to an adolescent (C) their engagement with low-intensity LSW increases (O) because they find advice from peers more credible and authentic (M).	(Holody & Mäher, 1996) (Hughes, 2013; Lucas et al., 2020) (Fitzhardinge, 2008)	'James was able to show his lifebook to another foster child who was resistant to beginning this work.' (Holody & Mäher, 1996)<br><br>'The use of published story books which describe similar scenarios of children who are in similar circumstances - such as where the child in the story is also blaming themselves about their parents' separation - can be very helpful.' (Hughes, 2013)

Improving the mental health and mental health support available to adolescents in out-of-home care via Adolescent-Focused Low-Intensity Life Story Work: A realist review

**CMOC51**	Caring adults who are supporting low-intensity LSW should have access to regular supervision (C) this helps the caring adult feel prepared and confident to support low-intensity LSW and improves their practice (O) because they have support and guidance available to reinforce training and a self-reflective space to process emotions (M).	(Walker & Ryan, 2016a) (Braiden, 2016) (Hooley et al., 2016) (Eenshuistra et al., 2019) (Haight et al., 2010) (Furnivall & Grant, 2014) (Buchanan, 2014) (Connor et al., 1985) (Watson, Hahn, et al., 2020) (Holody & Mäher, 1996) (Peake, 2009)	‘Unless training is reinforced by regular supervision and consultancy and embedded within a trauma sensitive organisational system, it will be unable to change children’s experience significantly.’ (Furnivall & Grant, 2014)<br><br>‘Clear areas for support and consultation that could be provided by clinical psychologists and social care professionals have been highlighted together with areas for future research.’ (Hooley et al., 2016)<br><br>‘Any worker undertaking life story work requires not only a reasonable knowledge of child development but also access to other workers in this field who can be called upon to give advice and support in the way that we used the clinical psychologist.’ (Connor et al., 1985)

Improving the mental health and mental health support available to adolescents in out-of-home care via Adolescent-Focused Low-Intensity Life Story Work: A realist review

References

ARCBOX. (2022). *Digital Life Story ARCBOX* ARC Adoption North East. https://www.arcadoptionne.org.uk/life-story

Atwell, A. (2016). Working with children with a disability. In R. Walker & T. Ryan (Eds.), *Life Story Work why, what, how and when* (pp. 97-102). CoramBAAF Adoption and Fostering Academy.

Atwool, N. (2017). Life story work: Optional extra or fundamental entitlement? *Child Care in Practice*, *23*(1), 64-76. https://www.tandfonline.com/doi/full/10.1080/13575279.2015.1126228

Aust, P. H. (1981). Using the Life Story Book in Treatment of Children in Placement. *Child Welfare*, *60*(8), 535-536,553-560. https://www.jstor.org/stable/45393827

Aventin, A., Houston, S., & Macdonald, G. (2014). Utilising a computer game as a therapeutic intervention for youth in residential care: Some preliminary findings on use and acceptability. *Children and Youth Services Review*, *47*, 362-369. http://ovidsp.ovid.com/ovidweb.cgi?T=JS&PAGE=reference&D=psyc11&NEWS=N&AN=2014-55927-021

BASW. (2020). *Recording in childrens social work*. https://www.basw.co.uk/system/files/resources/basw_recording_in_childrens_social_work_aug_2020.pdf

Baynes, P. (2008). Untold stories: A discussion of life story work. *Adoption & Fostering*, *32*(2), 43-49. https://doi.org/10.1177%2F030857590803200206

Bazalgette, T. R., G. Trevelyan. (2015). Achieving emotional wellbeing for looked after children a whole system approach. .

Beste, H. M., & Richardson, R. G. (1981). Developing a Life Story Book Program for Foster Children. *Child Welfare*, *60*(8), 529-534. https://www.jstor.org/stable/45393826

Bolton, C. (2022). *Life Story Work 5 minute facts* https://www.bolton.gov.uk/downloads/file/2299/life-story-work

Braiden, S. (2016). Evaluation of a short training programme for foster carers. *Scottish Journal of Residential Child Care*, *15*(2). https://www.celcis.org/application/files/2016/2308/5288/004._2016_Vol_15_2_Braiden_Evaluation_for_foster_carers.pdf

Brookfield, H., Brown, S. D., & Reavey, P. (2008). Vicarious and post-memory practices in adopting families: The re-production of the past through photography and narrative. *Journal of Community & Applied Social Psychology*, *18*(5), 474-491. https://onlinelibrary.wiley.com/doi/abs/10.1002/casp.960

Buchanan, A. (2014). *The experience of life story work: reflections of young people leaving care* Cardiff University]. EThOS. https://orca.cardiff.ac.uk/64531/

Connor, T., Sclare, I., Dunbar, D., & Elliffe, J. (1985). Making a life story book. *Adoption & Fostering*, *9*(2), 32-46. https://dx.doi.org/10.1177/030857598500900210

Cook-Cottone, & Beck. (2007). A Model for Life-Story Work: Facilitating the Construction of Personal Narrative for Foster Children. *Child Adolesc Ment Health*, *12*(4), 193-195. https://doi.org/10.1111/j.1475-3588.2007.00446.x

Improving the mental health and mental health support available to adolescents in out-of-home care via Adolescent-Focused Low-Intensity Life Story Work: A realist review

Davies, M., & Hodges, J. (2017). Relationship renaissance: the use of attachment-based narrative and metaphor in life story work. *Adoption & Fostering*, *41*(2), 131-141. https://doi.org/10.1177%2F0308575917702831

Devenney, K. (2017). Pathway planning with unaccompanied young people leaving care: Biographical narratives of past, present, and future. *Child & Family Social Work*, *22*(3), 1313-1321. https://onlinelibrary.wiley.com/doi/full/10.1111/cfs.12348

Eenshuistra, A., Harder, A. T., & Knorth, E. J. (2019). One size does not fit all: A systematic review of training outcomes on residential youth care professionals' skills. *Children and Youth Services Review*, *103*, 135-147. https://doi.org/10.1016/j.childyouth.2019.05.010

Eldridge, J. (2018). *Confiding in Others: A Qualitative Study Exploring the Experiences of Young people who have been in the Care System* University of Surrey Guildford].

Ferrier, M. (2011). Life story work in the context of attachment-led care planning. *Scottish Journal of Residential Child Care*, *10*(1). https://www.celcis.org/application/files/4516/2316/8859/2011_Vol_10_1_Ferrier_Life_story_work.pdf

Finlay, J. (2022). *Meaningful Natural Mentoring Relationship Characteristics and informal therapeutic life spcae interactions in youth care* Canterbury Christ Church University].

Fitzhardinge, H. (2008). Adoption, Resilience and the Importance of Stories: The Making of a Film about Teenage Adoptees. *Adoption & Fostering*, *32*(1), 58-68. https://doi.org/10.1177/030857590803200108

Furnivall, J., & Grant, E. (2014). *Trauma sensitive practice with children in care* (Insights: evidence summaries to support social services in Scotland, Issue. https://www.iriss.org.uk/sites/default/files/iriss-insight-27.pdf

Gallagher, B., & Green, A. (2012). In, out and after care: Young adults' views on their lives, as children, in a therapeutic residential establishment. *Children and Youth Services Review*, *34*(2), 437-450. https://dx.doi.org/10.1016/j.childyouth.2011.11.014

Gray, S., Hahn, R., Cater, K., Watson, D., Meineck, C., & Metcalfe, T. (2019). *Trove: A Digitally Enhanced Memory Box for Looked after and Adopted Children* Proceedings of the 18th ACM International Conference on Interaction Design and Children, https://www.researchgate.net/publication/333628573_Trove_A_Digitally_Enhanced_Memory_Box_for_Looked_after_and_Adopted_Children/link/5d31cbc4299bf1995b382ab3/download

Gustavsson, N., & MacEachron, A. (2008). Creating Foster Care Youth Biographies: A Role for the Internet. *Journal of Technology in Human Services*, *26*(1), 45-55. https://www.tandfonline.com/doi/abs/10.1300/J017v26n01_03

Gutsche, K. H. (2013). *Perceptions of social workers regarding life story work with children in child youth centres* North-West University].

Haight, W., Black, J., & Sheridan, K. (2010). A Mental Health Intervention for Rural, Foster Children from Methamphetamine-involved Families: Experimental Assessment with Qualitative Elaboration. *Child Youth Serv Rev*, *32*(10), 1146-1457. https://doi.org/10.1016/j.childyouth.2010.06.024

Hamilton, E. (2020). Life story approaches and relationships within residential child care: A practice reflection. *Relational Child and Youth Care Practice*, *33*(1),

87. https://www.celcis.org/application/files/8316/2307/7669/2019_Vol_18_No__2_Hamilton_E_A_practice_reflection.pdf

Hammond, S. (2012). *Exploring a role for digital technology in life story work with adolescents in residential care: a discourse analysis* University of East Anglia]. EThOS. https://ueaeprints.uea.ac.uk/id/eprint/39133/

Hammond, S. (2016). Digital Life Story Work in Action. In R. Walker & T. Ryan (Eds.), *Life story work Why, what, how and when* (pp. pp.106-119). CoramBAAF Adoption and Fostering Academy.

Hammond, S., & Cooper, N. (2013). *Digital Life Story Work: Using technology to help young people make sense of their experience.* (1 ed.). British Association of Adoption and Fostering.

Hammond, S. P., Cooper, N. J., & Jordan, P. (2021). Mental health, identity and informal education opportunities for adolescents with experience of living in state care: a role for digital storytelling. *Cambridge Journal of Education*, *51*(6), 713-732. https://doi.org/10.1080/0305764x.2021.1919057

Hanna, M. D. (2007). Preparing school age children for adoption: perspectives of successful adoptive parents and caseworkers. *Adoption Quarterly*, *10*(2), 1-32. https://www.tandfonline.com/doi/abs/10.1300/J145v10n02_01

Happer, H., McCreadie, J., & Aldgate, J. (2006). *Celebrating Success: What Helps Looked After Children Succeed*. Social Work Inspection Agency.

HCC. (2022). *Direct Work and Life Story Work with Children and Young People*. Hertfordshire County Council. Retrieved 12.08.22 from https://hertschildcare.proceduresonline.com/p_direct_life_story.html

Hills, L. (2022). In *Ensuring children's life stories are at the centre of direct work.*

Holody, R., & Mäher, S. (1996). Using lifebooks with children in family foster care a here and now process model. *Child Welfare*, *75*(4), 321-335.

Hooley, K., Stokes, L., & Combes, H. (2016). Life story work with looked after and adopted children: how professional training and experience determine perceptions of its value. *Adoption & Fostering*, *40*(3), 219-233. https://doi.org/10.1177%2F0308575916661129

Hooley, K. C. (2015). *Identifying perspectives on life story work with looked-after and adopted children* Staffordshire University]. EThOS. http://eprints.staffs.ac.uk/2244/

Hoyle, V., Shepherd, E., Lomas, E., & Flinn, A. (2020). Recordkeeping and the life-long memory and identity needs of care-experienced children and young people. *Child & Family Social Work*, *25*(4), 935-945. https://doi.org/https://doi.org/10.1111/cfs.12778

Hughes, J. I. (2013). Life-story work: a journey into a child's world. *Seen and Heard*, *23*(1), 23-30.

Humphreys, C., & Kertesz, M. (2014). Making Records Meaningful: Creating an Identity Resource for Young People in Care. *Australian Social Work*, *68*(4), 497-514. https://doi.org/10.1080/0312407x.2014.960434

Känkänen, P., & Bardy, M. (2014). Life stories and arts in child welfare: enriching communication. *Nordic Social Work Research*, *4*(1), 37-51. https://www.tandfonline.com/doi/full/10.1080/2156857X.2013.781536

Lucas, J., Matthews, L., Brady, K., Breguet, R., & Parson, J. (2020). *Therapeutic Life Story Work Barwon Pilot Program: Interim Report 2020* [Research report].

Malik, S. (2005). *Application of attachment theory for training foster parents: A model program* https://www.proquest.com/dissertations-theses/application-attachment-theory-training-foster/docview/305340219/

Monson, K., Moeller-Saxone, K., Humphreys, C., Harvey, C., & Herrman, H. (2020). Promoting mental health in out of home care in Australia. *Health Promot Int*, *35*(5), 1026-1036. https://doi.org/10.1093/heapro/daz090

Neil, E., & Beek, B. (2020). Resepecting Children's Relationships and Identities in Adoption. In *The Routledge Handbook of Adoption* (1st ed., pp. 77-89). Routledge.

NICE. (2021). *Looked after children and young people NICE guideline*. www.nice.org.uk/guidance/ng205

Nicholls, E. (2003). Model answer. *Community Care*(1479), 32-34. https://www.communitycare.co.uk/2003/07/03/model-answer/

NSPCC. (2022). *Life Story Work NSPCC Learning*. https://learning.nspcc.org.uk/services-children-families/life-story-work#heading-top

Nuffield Family Justice Observatory. (2021). *Modernising post-adoption contact: findings from a recent consultation*. https://www.nuffieldfjo.org.uk/wp-content/uploads/2021/08/nfjo_report_adoption_connections_20210913v2.pdf

Pakrosnis, R., & Cˇepukiene, V. (2011). Outcomes of Solution-Focused Brief Therapy for Adolescents in Foster Care and Health Care Settings. In C. Franklin, T. S. Trepper, E. E. McCollum, & W. J. Gingerich (Eds.), *Solution-Focused Brief Therapy: A Handbook of Evidence-Based Practice*. Oxford Scholarship Online.

Peake, A. (2009). Life Story Work. A resource for foster carers, residential social workers, adoptive parents, and kinship carers, to support this work. . In.

Sanders, R. (2020). *ESSS Outline: Care experienced children and young people's mental health*. https://doi.org/10.31583/esss.20201012

Shepard, E. (2022). *Good practice in record-keeping in children's social care*. Research in Practice. https://www.researchinpractice.org.uk/children/news-views/2022/february/good-practice-in-record-keeping-in-children-s-social-care/

Shotton, G. (2010). Telling different stories The experience of foster/adoptive carers in carrying out collaborative memory work with children. *Adoption & Fostering*, *34*(4), 61-68. https://doi.org/10.1177%2F030857591003400407

Shotton, G. (2012). *"Remember when...": exploring the experiences of looked after children and their carers in engaging in collaborative reminiscence* University of Newcastle upon Tyne]. EThOS. http://theses.ncl.ac.uk/jspui/handle/10443/1685

Shotton, G. (2013). 'Remember when...': exploring the experiences of looked after children and their carers in engaging in collaborative reminiscence. *Adoption & Fostering*, *37*(4), 352-367. https://doi.org/10.1177%2F0308575913508721

Steenbakkers, A., van der Steen, S., & Grietens, H. (2016). 'To talk or not to talk?': Foster youth's experiences of sharing stories about their past and being in foster care. *Children and Youth Services Review*, *71*, 2-9. https://doi.org/10.1016/j.childyouth.2016.10.008

Sunderland, F. (2021). Foster Carers Handbook. In.

Teodorczuk, K., Guse, T., & du Plessis, G. A. (2018). The effect of positive psychology interventions on hope and well-being of adolescents living in a

child and youth care centre. *British Journal of Guidance & Counselling*, *47*(2), 234-245. https://doi.org/10.1080/03069885.2018.1504880

Together for Children Sunderland. (2019). *Foster Carers Handbook April 2019*. https://www.togetherforchildren.org.uk/sites/default/files/2019-12/Foster%20Carer%20HandbookFinalDraft16052019%20-%20Updated%20April%202019.pdf

Walker, R., & Ryan, T. (2016a). Why do life story work? In R. Walker & T. Ryan (Eds.), *Life story work Why, what, how and when* (pp. 4-17). CoramBAAF Adoption and Fostering Academy.

Walker, R., & Ryan, T. (2016b). Working with black and minority ethnic children. In R. Walker & T. Ryan (Eds.), *Life story work Why, what, how and when* (pp. 77-84). CoramBAAF Adoption and Fostering Academy.

Ward, A. (2002). Opportunity led work maximising the possibilities for therapeutic communication in everyday interactions. *Therapeutic Communities*, *23*(2), 111-124.

Watson, Hahn, & Staines. (2020). Storying special objects: Material culture, narrative identity and life story work for children in care. *Qualitative Social Work: Research and Practice*, *19*(4), 701-718. https://doi.org/10.1177%2F1473325019850616

Watson, Latter, & Bellew. (2015a). Adopted children and young people's views on their life storybooks: The role of narrative in the formation of identities. *Children and Youth Services Review*, *58*, 90-98. https://dx.doi.org/10.1016/j.childyouth.2015.09.010

Watson, Latter, & Bellew. (2015b). Adopters' views on their children's life story books. *Adoption & Fostering*, *39*(2), 119. https://doi.org/10.1177%2F0308575915588723

Watson, Staples, & Riches. (2020). 'We need to understand what's going on because it's our life': Using sandboxing to understand children and young people's everyday conversations about care. *Children & Society*, *35*(5), 663-679. https://doi.org/10.1111/chso.12432

Watson, D., Meineck, C., & Lancaster, B. (2018). Adopted children's co-production and use of 'trove' (a digitally enhanced memory box) to better understand their care histories through precious objects. *Clinical child psychology and psychiatry*, *23*(4), 614-628. https://dx.doi.org/10.1177/1359104518776359

Watts, R. (2021). Relationships, reviews and recording: Developing practice for children in care. *Practice: Social Work in Action*, *33*(3), 207-222. https://www.tandfonline.com/doi/full/10.1080/09503153.2021.1889488

Willis, R., & Holland, S. (2009). Life story work: Reflections on the experience by looked after young people. *Adoption & Fostering*, *33*(4), 44-52. https://doi.org/10.1177%2F030857590903300406

Wood, J. (2019). West Sussex Children's imporvement board. Performance Report Against the Practice Improvement Plan Improving Quality of Life Story Work.

Wood, M., & Selwyn, J. (2017). Looked after children and young people's views on what matters to their subjective well-being. *Adoption & Fostering*, *41*(1), 20-34. https://doi.org/10.1177/0308575916686034
