# Supplementary material for: Improving the mental health and mental health support available to adolescents in out-of-home care via Adolescent-Focused Low-Intensity Life Story Work: a realist review
Source: BMJ Open. 2023 Oct 9;13(10):e075093. doi: 10.1136/bmjopen-2023-075093 (PMC10565277; doi:10.1136/bmjopen-2023-075093)
Supplement: Author's manuscript [file bmjopen-2023-075093.draft_revisions.pdf]

BMJ Open is committed to open peer review. As part of this commitment we make the peer review history of every article we publish publicly available.

When an article is published we post the peer reviewers' comments and the authors' responses online. We also post the versions of the paper that were used during peer review. These are the versions that the peer review comments apply to.

The versions of the paper that follow are the versions that were submitted during the peer review process. They are not the versions of record or the final published versions. They should not be cited or distributed as the published version of this manuscript.

BMJ Open is an open access journal and the full, final, typeset and author-corrected version of record of the manuscript is available on our site with no access controls, subscription charges or pay-per-view fees (http://bmjopen.bmj.com).

If you have any questions on BMJ Open's open peer review process please email info.bmjopen@bmj.com

# Improving the mental health and mental health support available to adolescents looked-after by social care via adolescent-focused low-intensity life story work: A realist review


| | |
|---|---|
| Journal: | *BMJ Open* |
| Manuscript ID | bmjopen-2023-075093 |
| Article Type: | Original research |
| Date Submitted by the Author: | 26-Apr-2023 |


| | |
|---|---|
| Complete List of Authors: | Hammond, Simon; University of East Anglia, School of Education and Lifelong Learning<br>Mickleburgh, Ella; Norfolk and Suffolk NHS Foundation Trust, Research<br>Duddy, Claire; University of Oxford, Nuffield Department of Primary Care Health Sciences<br>Hiller, Rachel; University College London, Division of Psychology and Language Sciences<br>Neil, Elsbeth; University of East Anglia, School of Social Work<br>Blackett, Rosie; National House Project<br>Williams, Kevin; The Fostering Network<br>Wilson, Jon; Norfolk and Suffolk NHS Foundation Trust<br>Wong, Geoff; Oxford University, Nuffield Department of Primary Care Health Sciences |


| | |
|---|---|
| Keywords: | Adolescent, MENTAL HEALTH, Systematic Review |
| | |


SCHOLARONE™
Manuscripts

*I, the Submitting Author has the right to grant and does grant on behalf of all authors of the Work (as defined in the below author licence), an exclusive licence and/or a non-exclusive licence for contributions from authors who are: i) UK Crown employees; ii) where BMJ has agreed a CC-BY licence shall apply, and/or iii) in accordance with the terms applicable for US Federal Government officers or employees acting as part of their official duties; on a worldwide, perpetual, irrevocable, royalty-free basis to BMJ Publishing Group Ltd ("BMJ") its licensees and where the relevant Journal is co-owned by BMJ to the co-owners of the Journal, to publish the Work in this journal and any other BMJ products and to exploit all rights, as set out in our licence.*

*The Submitting Author accepts and understands that any supply made under these terms is made by BMJ to the Submitting Author unless you are acting as an employee on behalf of your employer or a postgraduate student of an affiliated institution which is paying any applicable article publishing charge ("APC") for Open Access articles. Where the Submitting Author wishes to make the Work available on an Open Access basis (and intends to pay the relevant APC), the terms of reuse of such Open Access shall be governed by a Creative Commons licence – details of these licences and which Creative Commons licence will apply to this Work are set out in our licence referred to above.*

*Other than as permitted in any relevant BMJ Author's Self Archiving Policies, I confirm this Work has not been accepted for publication elsewhere, is not being considered for publication elsewhere and does not duplicate material already published. I confirm all authors consent to publication of this Work and authorise the granting of this licence.*

For peer review only

# Improving the mental health and mental health support available to adolescents looked-after by social care via adolescent-focused low-intensity life story work: A realist review

Simon P Hammond[ab]*, Ella Mickleburgh[b], Claire Duddy[c], Rachel Hiller[de], Elsbeth Neil[f], Rosie Blackett[g], Kevin Williams[h], Jon Wilson[b], Geoff Wong[c].

[a]*School of Education and Lifelong Learning, University of East Anglia (UEA), Norwich, United Kingdom (UK);* [b]*Norfolk and Suffolk NHS Foundation Trust, Norwich, UK,* [c]*Nuffield Department of Primary Care Health Sciences, University of Oxford, UK;* [d]Division of Psychology and Language Sciences, University College London, UK; [e]Anna Freud National Centre for Children and Families, London; [f]*Centre for Research on the Child and Family, School of Social Work, UEA, Norwich, UK;* [g]*The National House Project, UK;* [h]*The Fostering Network, UK.*

**School of Education and Lifelong Learning, Faculty of Social Sciences, University of East Anglia. Norwich. NR4 7TJ. United Kingdom. Tel: (+44) (0)1603 591460. Email: s.hammond@uea.ac.uk*

*Keywords*

Adolescent, life story work, realist review, mental health, mental health support

Word count: 4,925 (excluding references and table)



For peer review only - http://bmjopen.bmj.com/site/about/guidelines.xhtml

# Improving the mental health and mental health support available to adolescents looked-after by social care via adolescent-focused low-intensity life story work: A realist review


## Abstract

**Objectives:** Life Story Work (LSW) is an intervention used to promote the mental health and wellbeing of children and adolescents 'looked-after' by social care. LSW should be offered to all but is traditionally delivered in a high-intensity way. Low-intensity approaches are used but there is significant variation and little guidance for those using these to support adolescents. This project aimed to improve the guidance available to users of low intensity approaches to LSW.

**Design:** Realist review

**Data sources:** MEDLINE, Embase, PsycINFO, Sociology Collection (Proquest), CINAHL, CDAS, Web of Science (SCIE, SSCI), Social Care Online and grey literature sources.

**Eligibility criteria:** Documents reporting on children and adolescents looked-after, LSW and/or low-intensity interventions to improve mental health were included. Documents focusing on parenting style and contact with birth family members were excluded.

**Analysis:** Documents were analysed using a realist logic of analysis. In consultation with Content Expert Groups (comprising professionals and care leavers), we developed an initial programme theory. Data relating to and challenging the initial programme theory were extracted and context-mechanism-outcome configurations developed, critiqued, and refined in an iterative fashion. Inferences were drawn from context-mechanism-outcome-configurations to refine the Programme Theory.

**Results:** 75 documents contributed to the analysis. Generally, studies were small-scale and lacked in-depth description of methods and evaluation processes utilised. The analysis found important factors contributed to the development of high-quality Adolescent-Focused Low-Intensity LSW (AF-LI-LSW). AF-LI-LSW should be flexible and person-centred, begin in the present day, involve co-construction, be adolescent-led, record everyday positive life events, and be supported by a trained caring adult(s). Context-mechanism-outcome-configurations relating to these themes are reported.

**Conclusions:** To develop the AF-LI-LSW evidence-base and address gaps in our knowledge about the impact of AF-LI-LSW and how it works, further primary research is needed to strengthen understandings of how the components of AF-LI-LSW function in different contexts.

**PROSPERO registration number:** CRD42021279816.




For peer review only - http://bmjopen.bmj.com/site/about/guidelines.xhtml

# Strengths and limitations of the study

- This is the first realist review of Adolescent-Focused Low-Intensity Life Story Work (AF-LI-LSW) and it improves our understanding of how this intervention may work adolescents with social care experience.
- The review benefited from ongoing consultation with stakeholders in two separate Content Expert Groups, featuring young adults with care-experience and professionals, as recipients and deliverers of AF-LI-LSW.
- Content Expert Groups identified gaps in the evidence base, meaning there were limitations in how these aspects could be incorporated into the developing Programme Theory.
- Future primary research is invaluable and needed: (i) to further consolidate AF-LI-LSW's core components (elements that cannot be changed) and its adaptable periphery (elements that can be changed), (ii) to identify important outcomes for adolescents in care and those who support them (iii) and deliver a comprehensive evaluation to enable the therapeutic potential of AF-LI-LSW to be examined.

For peer review only

# Improving the mental health and mental health support available to adolescents looked-after by social care via adolescent-focused low-intensity life story work: A realist review

## Introduction and background

There are currently over 105,000 children and adolescents 'looked-after' by social care in the United Kingdom (UK) (1-4). Within this population, adolescents are the largest group currently in, and fastest growing age group entering care in England (1). They are amongst the most vulnerable members of society (5). They are six times more likely than the general population to experience mental illness (6). Despite this, their mental health needs are "underreported and undertreated" (7), leading the National Institute for Health and Care Excellence (NICE) to describe the need to increase the evidence-base for effective mental health interventions for adolescents in care as an "urgent research priority" (8).

Life Story Work (LSW) is an existing transdiagnostic intervention thought to improve the well-being and mental health of those living in care (9). It aims to improve individuals' sense of identity, ethnic heritage, placement stability, and mental health, by addressing gaps in self-knowledge, reframing past events, and increasing positive future expectations (8-14). Typical LSW components include a therapeutic alliance (relationship with caring adults), procedures (prompts to action), products (materials/artefacts/life story books), and therapeutic activities (rescripting/reframing).

NICE states LSW should be offered to all those living in care (8). LSW is often delivered in a high-intensity way, involving specialist professional (e.g., Social Worker, Educational Psychologist, Clinical Psychologist, Psychotherapist) input over many sessions and months. This is potentially expensive and impractical, limiting its availability. LSW also tends to be delivered to younger children (aged 0-11 years) meaning older children (i.e., adolescents) in care (12-18 years old) often miss out (13, 15). LSW can be delivered in a lower-intensity way, for example, by foster carers. This increases its accessibility and is sometimes called 'low-intensity' LSW.

The intensity of LSW vary along several continua including whether their delivery involves support from a specialist professional or not, delivery mode (face-to-face and/or digital), duration (prescribed sessions or ongoing) and intensity of services provided (e.g., past and/or trauma-focused or child led) (16, 17). Low-Intensity Life Story Work (LI-LSW) usually involves a carer (therapeutic alliance) recording (prompts to action) potentially valuable parts of a child's every day present circumstances (product creation). The carer and child discuss these, helping the child to process and consolidate their experiences (therapeutic activities). This in turn serves to generate positive future expectations and helps a therapeutic alliance develop between child and carer (14, 18). There is currently no accepted standard for LI-LSW delivery or high-quality evidence-based guidance and yet evidence has repeatedly highlighted the harms of poorly undertaken LSW (19-26). What little guidance there is focuses mainly on younger children in care (21, 27-29).

The current lack of adolescent-focused LI-LSW guidance means adolescents in care experience ongoing mental health inequalities and inconsistent practices leaving them and their carers feeling unsupported (13, 15, 19, 21-23, 30, 31). To address this evidence and practice gap, we aimed to generate in-depth knowledge to understand if LSW can be delivered to adolescents in care as a low-intensity intervention by asking:



For peer review only - http://bmjopen.bmj.com/site/about/guidelines.xhtml

*How, why, to what extent, for whom and in what circumstances can low-intensity LSW interventions, or elements of LSW interventions, be delivered to improve important and relevant outcomes for adolescents with care experience with mental health and wellbeing needs?*

The end goal of the project was to develop a set of initial guidelines for optimising Adolescent-Focused LI-LSW (AF-LI-LSW) to improve the mental health and mental health support offered to adolescents in care.

## Methods

To address this gap, we conducted a realist review to synthesise evidence to help us understand the importance of context in the delivery of AF-LI-LSW, and the mechanisms by which outcomes, both intended and unintended, are produced. A realist review was selected because AF-LI-LSW is complex and how well it works (or not) depends on context, who delivers it, and how. This is vital as previous research has consistently illustrated that LSW is a complex intervention with context-sensitive outcomes (9, 15).

### Realist review

Realist approaches are grounded in the assumption that the same intervention will not work everywhere and for everyone (32). A realist review is a theory-driven interpretive type of literature review. They are particularly useful for generating knowledge about how complex interventions produce mixed outcomes by generating better understandings of how and why different outcomes occur (33-35). Central to realist reviews is the creation and iterative refinement of a Programme Theory (PrT).

A PrT describes how an intervention is expected to lead to its outcomes and in which contexts these should occur (33-35). Realist reviews begin with an initial PrT, developed from previous research, lived-experiences, and assumptions about how an intervention works. Literature searching is then undertaken, data extracted and synthesised using a realist logic of analysis to develop context–mechanism–outcome configurations (CMOCs). CMOCs are causal explanations describing how and why particular outcomes are generated in particular contexts (33-35). CMOCs are key components that inform any PrT as they underpin any causal claims made.

Realist reviews can draw on a breadth of data sources to inform the PrT. This was important due to the “practice-based-wisdom” of much LSW literature [ref], and the aim of the current review to understand the various contexts within which AF-LI-LSW may occur. Hence, it was important to not limit included documents to solely published academic literature. Conducting a realist review enabled the inherent complexity of the research question to be addressed by using any relevant data that accounted for the different settings and services adolescents in care may experience. Through its focus on commonly occurring causal processes that underlie LSW, the realist approach also potentially provides transferable explanations for how and why other low-intensity mental health intervention strategies ‘work’ (and do not work) for adolescents with care-experience. This enabled the study to begin to address knowledge gaps highlighted in previous work relating to improving implementation guidance (9, 15).

In this paper, we report on a realist review study conducted between September 2021 and January 2023. A five-step process for conducting the realist review was undertaken following published standards (34, 35). These steps are summarised in Table 1 below with full details about our methods available in our open-access protocol paper (10).



For peer review only - http://bmjopen.bmj.com/site/about/guidelines.xhtml

INSERT TABLE 1

## Patient and Public Involvement – working with experts via experience

The idea for the project from which this paper report was driven by the lead authors time in practice and shaped through working with a variety of experts via experience from this conception. Once funded, this review was informed by the involvement of two expert via experience groups. These groups consisted of a Care-experienced Content Expert Group (CCEG) which involved care leavers (young adults with recent experiences of living in social care), and a professional expert panel (known as the Content Expert Group (CEG)) which comprised of multidisciplinary professionals with divergent topic area knowledge and/or experiences (i.e., Foster Carers, Social Workers, Commissioners, Educational and Clinical Psychologists).

We met with the CCEG and CEG three times during the project. We asked these groups to review information, bring their own experiences to bear on this, and help us to critique and refine the developing PrT. These discussions helped clarify important areas that needed explanation (e.g., the need for AF-LI-LSW to be flexible) and where the existing literature was lacking (e.g., peer-support for adolescents engaging with AF-LI-LSW), thus shaping our analysis. In our final meetings, we asked each group to help us to develop and refine an initial set of guidelines. Both groups also helped develop dissemination strategies, ensuring project outputs were tailored to different audiences.

# Results

## Document characteristics

In total, 75 documents were included in the review. Documents were published between 1981-2022 and were from 8 countries (56 from the UK). Included documents were research articles (36%), commentaries or practice guidance (32%), web sources (3%), reports (5%), conference paper (1%), quality standards (1%), books or book chapters (11%) and theses (11%). Of the research articles, 78% were qualitative studies, 4% used mixed methods, 7% were literature reviews, 7% were matched pairs and 4% were randomised trials. Judgements were made concerning the relevance of data within documents and whether data could be used to improve our understanding of how AF-LI-LSW could be implemented. The main reasons for exclusion were documents being about the wrong population or not containing information that contributed relevant data to the context, mechanism, or outcome configurations for AF-LI-LSW. The processes of identifying, screening, and selecting documents are illustrated in Figure 1: An adapted version of the PRISMA flow diagram. A detailed summary of included documents are available include in Supplementary File 2.

INSERT FIGURE 1 – PRISMA

## Review findings

INSERT FIGURE 2 – INITIAL PROGRAMME THEORY

The initial PrT presented a linear explanation of AF-LI-LSW (see Figure 2). What was clear within the literature and from consultations with our CCEG and CEG was the current low-quality of the evidence-base, alongside the need for AF-LI-LSW to be both continual and flexible. Data synthesis resulted in a consolidated PrT from which a set of initial guidelines for optimising AF-LI-LSW to improve the mental health and mental health support offered to adolescents in care was created (see Figure 3). Eight initial guidelines are outlined below. The



For peer review only - http://bmjopen.bmj.com/site/about/guidelines.xhtml

narrative summary accompanying each guideline is based on our realist analysis that developed 51 CMOCs, underpinned by the data included in the review. To enhance transparency, we provide a detailed summary of the CMOCs developed and the data underpinning each in Supplemental File 3. We also provide references to illustrate CMOCs to support the report of the results of the review that follows.

INSERT FIGURE 3 – CONSOLIDATED PROGRAMME THEORY

**1. AF-LI-LSW should be flexible and person-centred (CMOC 1)**

Ten documents contributed to the development of this CMOC (8, 21, 28, 36-42). The review's core finding is the importance of AF-LI-LSW being flexible and person-centred. Adapting to the needs, interests and wishes of the adolescent is the key initial guideline that underpins all that follow.

> *'Take a flexible approach to life story work, and tailor it to the developmental age and needs of the looked-after child or young person' (8)*

**2. There is no 'bad time' to start AF-LI-LSW so this should start early and make use of everyday opportunities (CMOCs 2-7).**

Thirty-one documents contributed to the development of these CMOCs (8, 20-23, 28, 36-38, 40, 41, 43-61). The review identified two factors associated with when to begin, and how to initiate AF-LI-LSW. It should start immediately and take advantage of everyday opportunities. Beginning early helps preserve information and avoid narrative gaps. This ensures that adolescents have memory prompts available to begin to construct a coherent identity, with these prompts containing everyday events.

> *"Entries assist in helping the youth preserve memories…. These stories may someday help the youth establish a stronger sense of self based on positive memories of their past.' (52).*

A caveat within conventional, 'high-intensity' LSW literature is that it should begin at the 'right time'. This 'right time' is often described as when a child is in a stable placement and has trusting relationships to support them as this work involves processing their past, potentially traumatic experiences (28, 36). This is often a barrier to any LSW being implemented. However, an AF-LI-LSW approach focuses on an adolescent's everyday life and future, avoiding an over-emphasis on past experiences and freeing carers to begin this work immediately. This relationship may then serve as a vehicle to explore more sensitive previous experiences. However, as we were interested in increasing access and the standard of AF-LI-LSW, higher intensity LSW approaches are not the focus of this review and represent a distinct area of need.

**3. AF-LI-LSW should begin in the present day as this grows trust in caring adult(s) and relationships (CMOCs 8-10, 12-18, 30)**

Forty-three documents contributed to the development of these CMOCs (8, 20-22, 28, 36, 39, 40, 42, 43, 48-51, 54-57, 61-83). Difficulties in establishing and maintaining trusting relationships was a main theme. AF-LI-LSW should provide a platform to engage in positive activities between caring adults and adolescents, encouraging one-to-one time together and increasing communication (8, 20, 28, 36, 39-41, 49, 62-66). These shared activities should encourage increased communication between the adolescent and caring adults, strengthening relationships, and helping to grow trust and a therapeutic alliance between the



For peer review only - http://bmjopen.bmj.com/site/about/guidelines.xhtml

adolescent and caring adult(s). This helps the adolescent to understand themselves better as well as helping the caring adult become better attuned to the adolescent's needs, improving their ability to care for them effectively.

> *'Young people seemed to trust relationships where others would sensitively listen to their confidences, whilst remaining unconditionally accepting of them as a person…'(65).*

As well as contributing to interpersonal outcomes such as increased connection, communication, sense of belonging, enhanced ability to understand others behaviour and placement stability, the literature indicated the potential value an AF-LI-LSW approach/model has on intra-personal outcomes such as increased self-worth and emotion regulation. Adolescents can develop their emotional regulation skills through discussing thoughts and feelings and having a caring adult modelling adaptive behaviours to challenging experiences.

> *'If adults respond to children's distress in a calm but engaged way they demonstrate an alternative way of managing stress..' (84).*

**4. AF-LI-LSW should involve co-construction of narratives because this improves coherence and authenticity (CMOC 23-29 and 31)**

Forty-four documents contributed to the development of these CMOCs (19-23, 27, 28, 36-41, 43, 44, 47, 48, 50, 51, 54-57, 59, 61, 65, 67, 70, 71, 73, 76, 79-81, 85-94). The review shows narratives most valued by adolescents were constructed collaboratively with caring adult support. The therapeutic alliance enabled adolescents to feel supported to see a range of perspectives when interpreting events. When adolescents are actively involved in the co-construction of narratives, these narratives become more coherent and feel authentic because they reflect adolescents' lived experiences.

> *'The therapeutic process involves creating opportunities to open up conversational spaces. These spaces provide young people with opportunities to share accounts of their own lives, in their own words.'* (48).

Literature indicates that adolescents in care may construct self-limiting accounts of their lived experiences due to their chronic and/or traumatic nature (41, 43, 51, 95). Evidence indicates that poorly executed LSW has negative impacts (19-23). Having caring adult support can help adolescents reconstruct narratives, reducing self-blame and self-limiting interpretations of events and circumstances. When alternatively framed narratives are available, adolescents can become increasingly aware of their positive attributes. This can increase self-esteem and the availability of positive future expectations.

> *'There is something powerful in how people come to see their stories and make sense of their experiences. This is the heart of resilience – the ability of a person to understand their story in such a way that it creates opportunity rather than limits it.' (81).*

The inclusion of multiple voices and perspectives increases adolescent's awareness that there are different ways to interpret situations and circumstances. Relevant literature was limited in this area, perhaps pointing towards its inherent complexity and clashes between adolescent agency and professionalised notions of 'accuracy'.



For peer review only - http://bmjopen.bmj.com/site/about/guidelines.xhtml

*'Granting children control over narratives aids identity formation but left unchallenged their perspective of events may also become one-sided and inaccurate' (41).*

However, both of our stakeholder groups indicated the value of including significant individuals, including birth relatives and siblings in AF-LI-LSW. Literature indicated that when/if significant individuals are positively involved in AF-LI-LSW activities, this can facilitate the establishment of stronger relationships because of the interactions that result from the activity. The involvement of significant individuals may increase adolescents' sense of identity because they are able to access and process information about their heritage.

**5. Adolescents should be supported to control how their lives are recorded and preserved in AF-LI-LSW whenever and wherever possible (CMOC 32-42)**

Adolescents should control how their everyday life experiences are reflected on and preserved including the processes used for recording and preserving lived experiences as well as the resulting products.

**5.1 Processes for recording and preserving (CMOC 32-26)**

Twenty-six documents contributed to the development of these CMOCs (8, 19, 21, 23, 28, 36-39, 41, 42, 47-50, 53, 55, 57, 64, 66, 67, 80, 96, 97). The review indicates that being able to exert control over the process increases adolescents' sense of ownership. This helps adolescents engage better and feel their opinions are valued. This is invaluable for adolescents with care experience as often due to their circumstances their sense of power can be reduced.

'C*hildren can have ownership of their story work via choosing which objects to story/not story and by dictating the pace at which the work progresses… (FSW, Ben)' (41)*

There was limited literature surrounding how, when and who should be responsible for making LSW accessible for adolescents with special educational needs. The available literature and our stakeholder groups indicated the importance of the adolescents' support network feeling equipped with the knowledge and skill to make adaptions where necessary. Caring adult(s) will benefit from, for example, training materials developed by speech and language therapists. This may include written and visual resources that increase their understanding of how to make adaptations to improve adolescent's abilities to engage with AF-LI-LSW in a meaningful way. Though research was sparse in this area, our CCEG and CEG stated this was important to ensure that AF-LI-LSW is flexible and person-centred.

**5.2 Products (e.g., artefacts) (CMOCs 37 – 42)**

Thirty-one documents contributed to the development of these CMOCs (19, 21-23, 27, 28, 36, 37, 40-44, 47, 48, 50-52, 54-57, 80, 87, 92, 96-100). The literature highlighted that when an adolescent has editorial power to control what is recorded, they can feel more positive about the product(s). This is because what is recorded aligns more closely with their lived experiences. The risk of LSW products being lost or damaged was a theme within the literature. Literature and our CCEG and CEG repeatedly illustrated the benefits of digital tools/platforms to create and store editable products. These products were cited as holding meaning over time because they held identity information about adolescents, their relationships, and spaces at a point in time and could be updated and edited when needed.



For peer review only - http://bmjopen.bmj.com/site/about/guidelines.xhtml

*'Digital tools offer the ability to help young people to express thoughts and feelings which can be continually updated, edited and reflected upon. Using interactive computer-based mediums in this way grants the user flexibility to make changes frequently and easily.' (47)*

The importance of both physical, audio, and visual artefacts was emphasised. Physical objects can increase a sense of connection to others and/or past experiences via the tangible nature of the artefact that was present during life experiences. Audio and visual formats, including capturing the actual voice or video recording of significant individuals can be powerful, helping adolescents to situate memories and feel connected to their experiences and significant individuals.

*'For looked after and adopted children, physical objects are often the only remaining link to their past; a portal to stories of birth families, former homes, and significant people.' (41)*

**6. AF-LI-LSW should provide adolescents with somewhere to begin future telling (CMOC 11 and 43)**

Eight documents contributed to the development of CMOCs (36, 37, 40, 41, 43, 51, 64, 77). Our stakeholder groups repeatedly emphasised the life course nature of care-experience and enduring impacts that missing information has on identity and self-esteem throughout one's life. During adolescents' time in care, they can use their collection of preserved memories as storytelling prompts (e.g., a photo with a date, time, location, names of people and activity relating to) with caring adults, which helps others to understand them, strengthening relationships. This may include transitions within and beyond care. When preserved, life experiences can become a bank of narrative starting points (artefacts from which to begin reminiscing) for adolescents to share stories about themselves with future audiences.

*'The book acted as an aide to him telling me about his life, the things and people that are important to him.' (51)*

**7. AF-LI-LSW should record positive aspects of everyday experiences (CMOC 44-46)**

Twenty-one documents contributed to the development of these CMOCs (20, 21, 27, 28, 36, 39, 40, 49, 51, 54, 56, 61, 68, 71, 82, 83, 88, 98, 101, 102). The review highlighted the value of using AF-LI-LSW to capture adolescents' 'everyday magic' (i.e., an individual's mannerisms, characteristics, and their idiosyncrasies, humour and histories of relationships shared with those around them and everyday successes) helping adolescents to feel more positive about themselves and their relationships (40, 41, 49, 51, 54, 68, 82, 83, 88, 101). When achievements are preserved and reflected adolescent's ability to develop positive identities and future expectations are supported because the availability of positive self-narratives for interpreting their experiences are more readily available. They can become better at identifying future achievements themselves.

*'…it can help the child to thicken positive counter-narratives impacting positively on the dominant stories the child has and is able to tell about themselves, particularly thickening stories around their strengths, worth and belonging.' (51).*



For peer review only - http://bmjopen.bmj.com/site/about/guidelines.xhtml

**8 AF-LI-LSW should include support for adolescents, caring adults and others involved (48-51)**

Twenty-five documents contributed to the development of these CMOCs (8, 28, 36, 39-41, 43, 50, 51, 53, 56, 57, 60-62, 68, 70, 72, 74, 79, 81, 84, 89, 98, 103). As mentioned above, AF-LI-LSW should be flexible and person-centred. The literature highlights the essential role confidence has on the ability of caring adults to support AF-LI-LSW. It is important that caring adult(s) understand the adolescents' individuality and cultural heritage to develop their awareness of the potential significance of everyday lived experiences to promote appropriate preservation and reflection. Caring adults' need to be supported via training and ongoing supervision to improve their confidence and skills to support and facilitate AF-LI-LSW.

> *''Training to ensure a consistent approach to life story work could be incorporated into existing training.' (8)*

The literature indicated that training is most impactful when accompanied by supervision to consolidate learning. Supervision for caring adults ensures advice and guidance is readily available as well as providing a self-reflective space to process emotions and improve practice.

> *''Recognition of children's entitlement to a coherent narrative needs to be embedded in practice at the micro level. This can only occur if appropriate supports are in place at the organisational level, facilitated by macro-level priority….' (43)*

Alongside providing training and supervision for caring adults, the potential role of peer-support, for adolescents and caring adults and when implementing AF-LI-LSW was repeatedly mentioned repeatedly by our expert groups. Adolescents may benefit from peers supporting their understanding of AF-LI-LSW as during adolescence it is widely acknowledged that peers become increasingly influential (104), meaning there is the potential for peer support to be impactful. Peer-support for caring adults also hinted at ways to develop communities of practice. Literature was extremely limited on the role of peer-support both groups. However, other models of practice have employed peer support models with these populations with promising outcomes (e.g., The Mockingbird programme). This is an important direction for future research.

# Discussion

## Summary

Adolescent-Focused Low-Intensity Life Story Work (AF-LI-LSW) involves an adolescent and/or caring adult(s) recording potentially valuable parts of an adolescent's everyday life (e.g., diary entry or picture). The caring adult(s) and adolescent discuss these, helping the adolescent to process and consolidate their experiences. This in turn serves to generate positive future expectations and helps a therapeutic alliance develop between adolescent and caring adult(s) (8, 28, 39, 40, 49, 51, 63-65, 86-88, 90).

Our realist review, the first of its kind in this area, created a PrT for AF-LI-LSW and used this to produce preliminary guidance for how current practice should be improved. Our review is unique in describing considerations that support adolescents in care to experience ownership over how, when, why, and in what contexts their everyday lived experiences are narrated, recorded, and reflected upon during their time living in social care. A flexible and person-centred approach is key, both in generating feelings of investment and empowerment but also



For peer review only - http://bmjopen.bmj.com/site/about/guidelines.xhtml

in implementing AF-LI-LSW. An important task for caring adults is identifying and empathising with adolescents, particularly in relation to their desired level of involvement within AF-LI-LSW, which may fluctuate over time. Nevertheless, this review illustrates that AF-LI-LSW has core components (elements that cannot be changed) and is likely to have an ‘adaptable periphery’ (elements that can be changed) capable of adjusting to contextual factors (105).

## Comparison with existing literature

Systematic review evidence examining LSW interventions for children and adolescents in care concluded that LSW had potentially far-reaching benefits but an “immature” evidence-base (106). A recent attempt to improve this evidence-base was inconclusive (9), with a rare example of a Randomised Controlled Trial in this area ultimately lacking statistical power. However, the existing evidence does indicate that the lack of “robust implementation guidance, efficacy or cost-effectiveness studies” (15) is problematic and that “greater implementation knowledge” is needed to examine therapeutic outcomes (13). The current review adds to this by improving our understanding of how, why, what and whom should be involved in the delivery of AF-LI-LSW to produce potentially therapeutic outcomes. Further research is required to strengthen our understanding of how AF-LI-LSW delivery is impacted by individual circumstances experienced adolescents living within different placement contexts (e.g., foster, residential, kinship), what therapeutic outcomes are important for adolescents, caring adults, and social care more broadly before any future evaluations may be undertaken.

## Limitations

This review has several limitations. The literature that informed the CMOCs were supported by relevant and best currently available evidence. However, when framed by the hierarchy of evidence for therapeutic studies (107, 108) most of the available evidence is of a ‘low quality’ (9, 29, 109-111). Existing literature was mainly qualitative in nature, tended to focus on LSW undertaken with younger children (0-11 years old) and higher intensity delivery models. Whilst the available evidence with an adolescent focus was invaluable for illustrating ‘good’ and ‘bad’ practices, it did not provide enough detailed information about implementation or how different circumstances, including different placement contexts impact on outcomes or how AF-LI-LSW operated over time. Given the complexity of AF-LI-LSW, it is perhaps unsurprising that many studies reported variation in intervention and implementation. However, there were also issues within many studies where the description of both were either absent or underdeveloped. This can be remedied by researchers using tools such as the TIDierR or StaRI checklists to enable authors to efficiently outline interventions and report their implementation in detail (112, 113).

To develop CMOCs or some aspects of CMOCs which were only supported by limited evidence, we drew on expert opinion. These CMOCs were discussed with our expert groups who felt they were important to include. Gaps in these areas, (e.g., use of digital media, peer-to-peer support, engaging with adolescents in care with Special Educational Needs, and involving significant individuals (including birth relatives), effectiveness and implementation of training and supervision) also represent important future research directions.

## Implications for practice

AF-LI-LSW is not a silver bullet. However, considering NICE states LSW should be offered to all those living in care (8), that it is endorsed by legislation underpinning its usage (114-117) and widely valued by those living and working within social care (11, 19-23, 28, 36, 83), we hope our initial practice guidance (summarised in Supplementary File 4) can begin to improve



For peer review only - http://bmjopen.bmj.com/site/about/guidelines.xhtml

existing practices. In implementing this guidance, there is a need to consider where, how, and in what format AF-LI-LSW products are stored, who has ownership of this content, and how it can be transferred when those living in care move within and beyond the care system. Our Care-experienced Content Expert Group and Content Expert Group both repeatedly highlighted the potential of digital platforms/devices. They felt that a digital platform had the potential to be effectively used within AF-LI-LSW to record and collate everyday life experiences. This is something that members of the team have written about before (30, 37, 47, 48). However, existing research was sparse in this area and research to understand the value of digital platforms to support LSW, such as CaringLife, is ongoing currently.

### Implications for research

Future primary research is needed: (i) to further consolidate AF-LI-LSW's core components (elements that cannot be changed) and its adaptable periphery (elements that can be changed) to enable transferability, accessibility, and implementation, (ii) to identify important and relevant outcomes for adolescents in care and those who support them (iii) use this knowledge to inform the design of a comprehensive, appropriately powered evaluation, including economic and process elements of AF-LI-LSW. When considering AF-LI-LSW as an intervention, the evaluation of outcomes may be challenging. As a result, evaluations measuring the impact of AF-LI-LSW may need to assess success in relation to intermediate outcomes and longer-term outcomes over an extended follow up time.

## Conclusion

To our knowledge, this is the first realist review exploring how, why, to what extent and for whom AF-LI-LSW may work (or not) for adolescents in care. Our PrT and initial guidelines provide important ways to improve the nature of good practice when delivering AF-LI-LSW to adolescents in care and produce benefits for them, their carers and health/social-care professionals.

However, to make this approach accessible to all adolescents in care a programme of research is needed to clarify how contexts impact on outcomes to enable greater transferability and more successful implementation. Additionally, to improve the current low quality evidence base, a fully powered evaluation with embedded economic and process evaluations and long-term follow-up is required to generate knowledge regarding the therapeutic potential of this highly valued yet inconsistently implemented mental health intervention. Importantly, if not done properly AF-LI-LSW, like other interventions could do harm (118). Hence, any future evaluation is not just about optimising delivery but also minimising harm. Given that research consistently demonstrates that adolescents in care are amongst the most vulnerable in society, this difficult but not impossible challenge needs urgent action.



For peer review only - http://bmjopen.bmj.com/site/about/guidelines.xhtml

# References


1. Gov.UK. Children looked after in England including adoptions. 2022 [cited 2023 20.02.2023]. Available from: https://explore-education-statistics.service.gov.uk/find-statistics/children-looked-after-in-england-including-adoptions/2022
2. Gov.Scot. Children's Social Work Statistics, Scotland 2020-21 2022 [cited 2023 20.02.2023]. Available from: https://www.gov.scot/publications/childrens-social-work-statistics-scotland-2020-21/pages/4/.
3. Gov.Wales. Children looked after by local authorities: April 2021 to March 2022. 2022 [cited 2023 20.02.2023]. Available from: https://gov.wales/children-looked-after-local-authorities-april-2021-march-2022-html#section-109870.
4. NI.Gov. Looked after children, adoption and fostering statistics for Northern Ireland. 2022 [cited 2023 20.02.2023]. Available from: https://www.health-ni.gov.uk/publications/childrens-social-care-statistics-northern-ireland-202021.
5. Tarren-Sweeney M. The mental health of children in out-of-home care. Current Opinion in Psychiatry. 2008;21(4):345-9.
6. Meltzer H, Gatward, R., Corbin, T., Goodman, R., Ford, T. The Mental Health of Young People Looked After by Local Authorities in England London: TSO; 2003.
7. Commission CQ. The state of mental health services 2014 - 2017: Findings from CQCs programme of comprehensive inspections of specialist mental health services. 2018 [cited 2023 20.02.2023]. Available from: https://www.cqc.org.uk/sites/default/files/20170720_stateofmh_report.pdf.
8. NICE. Looked-after children and young people: NICE; 2021 [Available from: https://www.nice.org.uk/guidance/ng205/chapter/recommendations#life-story-work-for-identity-and-wellbeing.
9. Taylor S, Lawrence H, Blackshaw E, Stern D, Gilbert L, Mollidor C, et al. Evaluation of Creative Life Story Work. 2022 December 2022.
10. Hammond SP, Duddy C, Mickleburgh E, Hiller R, Neil E, Williams K, et al. Improving the mental health and mental health support available to adolescents with social care-experience via low-intensity life story work: a realist review protocol. BMJ Open. 2022;12(3):e058424.
11. Ryan T, Walker R. Life Story Work. London: CoramBAAF; 2016.
12. Herwig K. What Does Current Literature Tell Us About the Mental Health of Looked After Children? BJPsych Open. 2022;8(S1):S54-S.
13. Kontomichalos-Eyre SA, Lake AJ, McGillivray JA. Life story work for children and youth in out of home care: A systematic review and synthesis of qualitative studies. Children and Youth Services Review. 2023;144:106697.
14. Prince DM, Epstein M, Nurius PS, Gorman-Smith D, Henry DB. Reciprocal Effects of Positive Future Expectations, Threats to Safety, and Risk Behavior Across Adolescence. Journal of Clinical Child & Adolescent Psychology. 2019;48(1):54-67.
15. Hammond SP, Young J, Duddy C. Life story work for children and young people with care experience: A scoping review. Developmental Child Welfare. 2020;2(4):293-315.
16. NICE. Common mental health problems: identification and pathways to care: Clinical guideline [CG123] 2011 [cited 2023 07.03.23]. Available from: https://www.nice.org.uk/guidance/cg123
17. Kazdin AE, Rabbitt SM. Novel Models for Delivering Mental Health Services and Reducing the Burdens of Mental Illness. Clinical Psychological Science, 2013;1(2):170-91.
18. Askelund AD, Schweizer S, Goodyer, IM, van Harmelen, AL. Positive memory specificity is associated with reduced vulnerability to depression. Nat Hum Behav. 2019;3(3):265-73.
19. Baynes P. Untold Stories: A Discussion of Life Story Work. Adoption & Fostering. 2008;32(2):43-9.
20. Cook-Cottone C, Beck M. A Model for Life-Story Work: Facilitating the Construction of Personal Narrative for Foster Children. Child and Adolescent Mental Health. 2007;12(4):193-5.




For peer review only - http://bmjopen.bmj.com/site/about/guidelines.xhtml

21. Willis R, Holland S. Life Story Work: Reflections on the Experience by Looked after Young People. Adoption & Fostering. 2009;33(4):44-52.
22. Watson DL, Latter S, Bellew R. Adopters' views on their children's life story books. Adoption & Fostering. 2015;39(2):119-34.
23. Watson DL, Latter S, Bellew R. Adopted children and young people's views on their life storybooks: The role of narrative in the formation of identities. Children and Youth Services Review. 2015;58:90-8.
24. Staines J, Selwyn J. "I wish someone would explain why I am in care": The impact of children and young people's lack of understanding of why they are in out-of-home care on their well-being and felt security. . Child & Family Social Work. 2020.
25. Neil E. Making sense of adoption: Integration and differentiation from the perspective of adopted children in middle childhood. Children and Youth Services Review. 2012;34(2):409-16.
26. Selwyn J. Children and Young People's Views om Being in Care. Coram Voice:London, UK 2015.
27. Gallagher B, Green A. In, out and after care: Young adults' views on their lives, as children, in a therapeutic residential establishment. Children and Youth Services Review. 2012;34(2):437-50.
28. Hooley K, Stokes L, Combes H. Life story work with looked after and adopted children: how professional training and experience determine perceptions of its value. Adoption & Fostering. 2016;40(3):219-33.
29. Luke N, Sinclair I, Woolgar M, Sebba J. What works in preventing and treating poor mental health in looked after children? London, UK: Rees Centre; 2014.
30. Hammond SP, Cooper N. Digital life story work: Using technology to help young people make sense of their experiences. London: British Association of Adoption and Fostering; 2013.
31. Hiller RM, Halligan SL, Meiser-Stedman R, Elliott E, Rutter-Eley E. Supporting the emotional needs of young people in care: a qualitative study of foster carer perspectives. BMJ Open. 2020;10(3):e033317.
32. Duddy C. Wong G. Grand rounds in methodology: when are realist reviews useful, and what does a 'good' realist review look like? BMJ Quality & Safety 2023;32:173-80.
33. Pawson RT, Tilley N. Realist evaluation. 2004.
34. Pawson R, Greenhalgh, T., Harvey, G, Walshe, K. Realist review--a new method of systematic review designed for complex policy interventions. Journal Health Services Research Policy. 2005;10(1):21-34.
35. Wong G, Greenhalgh, T., Westhorp, G., & Pawson, R. Development of methodological guidance, publication standards and training materials for realist and meta-narrative reviews: the RAMESES (Realist And Meta-narrative Evidence Syntheses - Evolving Standards) project. . Health Services Delivery Research. 2014;2(30).
36. Buchanan A. The experience of Life Story Work: Reflections of Young People Leaving Care. Cardiff, UK: Cardiff University; 2014.
37. Hammond SP. Exploring a role for digital technologies in life story work with adolescents in residential care a discourse analysis Norwich: University of East Anglia; 2012.
38. Hertfordshire County Council. Direct Work and Life Story Work with Children and Young People. Hertfordshire County Council 2022 [Available from: https://hertschildcare.proceduresonline.com/p_direct_life_story.html.
39. Holody R, Mäher S. Using lifebooks with children in family foster care a here and now process model. Child Welfare,. 1996;75(4):321-35.
40. Shotton G. 'Remember when...': exploring the experiences of looked after children and their carers in engaging in collaborative reminiscence. Adoption & Fostering 2013;37(4):352-67.
41. Watson D, Hahn R, Staines J. Storying special objects: Material culture, narrative identity and life story work for children in care. Qualitative Social Work: Research and Practice. 2020;19(4):701-18.



For peer review only - http://bmjopen.bmj.com/site/about/guidelines.xhtml

42. Watson D, Meineck C, Lancaster B. Adopted children's co-production and use of 'trove' (a digitally enhanced memory box) to better understand their care histories through precious objects. Clinical child psychology and psychiatry,
. 2018;23(4):614-28.
43. Atwool N. Life story work: Optional extra or fundamental entitlement? Child Care in Practice. 2017;23(1):64-76.
44. Hoyle V, Shepherd E, Lomas E, Flinn A. Recordkeeping and the life-long memory and identity needs of care-experienced children and young people. Child & Family Social Work. 2020;25(4):935-45.
45. Together for Children Sunderland. Foster Carers Handbook April 2019. Sunderland City Council,; 2019.
46. Together for Children Sunderland. Foster Carers Handbook 2021 [cited 2022 13.11.22]. Available from: https://www.togetherforchildren.org.uk/media/25536/Foster-Carer-Handbook/pdf/Foster_Carer_Handbook.pdf?m=637983250338700000.
47. Hammond SP, Cooper N. Digital Life Story Work: Using technology to help young people make sense of their experience. 1 ed. London: British Association of Adoption and Fostering; 2013.
48. Hammond SP. Digital Life Story Work in Action. In: Ryan T, Walker R. editor. Life story work Why, what, how and when. UK: CoramBAAF Adoption and Fostering Academy.; 2016. p. 106-19.
49. Hammond SP, Cooper, N. J., & Jordan, P. Mental health, identity and informal education opportunities for adolescents with experience of living in state care: a role for digital storytelling. Cambridge Journal of Education. 2021;51(6):713-32.
50. Peake A. Life Story Work. A resource for foster carers, residential social workers, adoptive parents, and kinship carers, to support this work. Oxfordshire Foster Carers/Adopters; 2009.
51. Shotton G. "Remember when...": exploring the experiences of looked after children and their carers in engaging in collaborative reminiscence: University of Newcastle upon Tyne; 2012.
52. Gustavsson N, MacEachron A. Creating Foster Care Youth Biographies: A Role for the Internet. Journal of Technology in Human Services. 2008;26(1):45-55.
53. Lucas J, Matthews L, Brady K, Parson J, Breguet R. Therapeutic Life Story Work Barwon Pilot Program: Interim Report 2020. Geelong, Australia: School of Health and Social Development, Deakin University; 2020.
54. Ferrier M. Life story work in the context of attachment-led care planning. Scottish Journal of Residential Child Care. 2011;10(1).
55. Hooley KC. Identifying Perspectives on Life Story Work with Looked-after and Adopted Children. Staffordshire: Staffordshire University; 2015.
56. Watts R. Relationships, reviews and recording: Developing practice for children in care. Practice: Social Work in Action. 2021;33(3):207-22.
57. Hills L. Ensuring children's life stories are at the centre of direct work. [Internet]: Research in practice; 2022. Podcast. Available from: https://www.researchinpractice.org.uk/children/content-pages/podcasts/ensuring-childrens-life-stories-are-at-the-centre-of-direct-work/
58. Bolton Council B. Life Story Work. 5 Minute Facts. Available from: life-story-work (bolton.gov.uk)
59. Neil E, Beek B. Resepecting Children's Relationships and Identities in Adoption The Routledge Handbook of Adoption. 1st ed. UK: Routledge; 2020. p. 77-89.
60. Brookfield H, Brown, SD, Reavey P. Vicarious and post-memory practices in adopting families: The re-production of the past through photography and narrative. Journal of Community & Applied Social Psychology. 2008;18(5):474-91.
61. Watson DL, Staples E, Riches, K. 'We need to understand what's going on because it's our life': Using sandboxing to understand children and young people's everyday conversations about care. Children & Society
2020;35(5):663-79.
62. Braiden S. Evaluation of a short training programme for foster carers. Scottish Journal of Residential Child Care. 2016;15(2).



For peer review only - http://bmjopen.bmj.com/site/about/guidelines.xhtml

63. Malik S. Application of attachment theory for training foster parents: A model program. US: San Francisco Bay Campus; 2005.
64. Hamilton E. Life story approaches and relationships within residential child care: A practice reflection. Relational Child and Youth Care Practice. 2020;33(1):1-11.
65. Eldridge J. Confiding in Others: A Qualitative Study Exploring the Experiences of Young people who have been in the Care System. UK: University of Surrey Guildford; 2018.
66. Aventin A, Houston S, Macdonald G. Utilising a computer game as a therapeutic intervention for youth in residential care: Some preliminary findings on use and acceptability. Children and Youth Services Review. 2014;47:362-9.
67. Gutsche K. Perceptions of social workers regarding life story work with children in child and youth care centres: North-West University; 2013.
68. Haight W, Black, J, Sheridan K. A Mental Health Intervention for Rural, Foster Children from Methamphetamine-involved Families: Experimental Assessment with Qualitative Elaboration. Child Youth Serv Rev. 2010;32(10):1146-7.
69. Agencies TCoVA. CVAA submission to the Review's Call for Ideas. 2021.
70. Finlay J. Meaningful Natural Mentoring Relationship Characteristics and informal therapeutic life spcae interactions in youth care: Canterbury Christ Church University; 2022.
71. Shotton G. Telling different stories The experience of foster/adoptive carers in carrying out collaborative memory work with children. Adoption & Fostering. 2010;34(4):61-8.
72. Hughes JI. Life-story work: a journey into a child's world. Seen and Heard. 2013;23(1):23-30.
73. BASW England. Recording in Children's Social Work. 10 Top Tips.; 2020.
74. Wood M, Selwyn J. Looked after children and young people’s views on what matters to their subjective well-being. Adoption & Fostering. 2017;41(1):20-34.
75. Furnivall J, Grant E. Trauma sensitive practice with children in care. Iriss; 2014.
76. Davies M, Hodges J. Relationship renaissance: the use of attachment-based narrative and metaphor in life story work Adoption & Fostering. 2017;41(2):131-41.
77. Hanna MD. Preparing school age children for adoption: perspectives of successful adoptive parents and caseworkers. Adoption Quarterly. 2007;10(2):1-32.
78. Ward A. Opportunity led work maximising the possibilities for therapeutic communication in everyday interactions. Therapeutic Communities. 2002;23(2):111-24.
79. Connor T, Sclare, I., Dunbar, D, Elliffe, J. Making a life story book. Adoption & Fostering. 1985;9(2):32-46.
80. Känkänen P, Bardy, M. Life stories and arts in child welfare: enriching communication. Nordic Social Work Research 2014;4(1):37-51.
81. Fitzhardinge H. Adoption, Resilience and the Importance of Stories: The Making of a Film about Teenage Adoptees Adoption & Fostering. 2008;32(1):58-68.
82. Steenbakkers A, van der Steen S, Grietens H. ‘To talk or not to talk?’: Foster youth's experiences of sharing stories about their past and being in foster care. Children and Youth Services Review. 2016;71:2-9.
83. NSPCC. Life Story Work [Available from: https://learning.nspcc.org.uk/services-children-families/life-story-work/.
84. Furnivall J, Grant E. Trauma sensitive practice with children in care (Insights: evidence summaries to support social services in Scotland 2014 [Available from: https://www.iriss.org.uk/sites/default/files/iriss-insight-27.pdf.
85. Pakrosnis R, Cˇepukiene V. (2011). Outcomes of Solution-Focused Brief Therapy for Adolescents in Foster Care and Health Care Settings. Solution-Focused Brief Therapy: A Handbook of Evidence-Based Practice. In: C. Franklin TST, E. E. McCollum, & W. J. Gingerich (Eds.), editor. Oxford Scholarship Online2011.
86. Happer H, McCreadie J, Aldgate J. Celebrating Success: What Helps Looked After Children Succeed 2006 [cited 2023 12.02.23]. Available from: https://www.wecanandmustdobetter.org/files/8314/2781/0566/Celebrating_success.pdf.



For peer review only - http://bmjopen.bmj.com/site/about/guidelines.xhtml

87. Aust PH. Using the Life Story Book in Treatment of Children in Placement. Child Welfare. 1981;60(8):535-60.
88. Teodorczuk K, Guse, T, du Plessis GA. The effect of positive psychology interventions on hope and well-being of adolescents living in a child and youth care centre. British Journal of Guidance & Counselling. 2018;47(2):234-45.
89. Sanders R. ESSS Outline: Care experienced children and young people's mental health 2020 [cited 2023 20.02.23]. Available from: https://doi.org/10.31583/esss.20201012.
90. Devenney K. Pathway planning with unaccompanied young people leaving care: Biographical narratives of past, present, and future. Child & Family Social Work. 2017;22(3):1313-21.
91. Bazalgette L, Rahilly T, Trevelyan G. Achieving emotional wellbeing for looked after children. NSPCC; 2015.
92. Beste HM, Richardson RG. Developing a Life Story Book Program for Foster Children. Child Welfare. 1981;60(8):529-34.
93. ARCBOX. Digital Life Story ARCBOX ARC Adoption North East 2022 [cited 2023 16.02.23]. Available from: https://www.arcadoptionne.org.uk/life-story.
94. Monson K, Moeller-Saxone, K, Humphreys C, Harvey C, Herrman H. Promoting mental health in out of home care in Australia. Health Promot Int. 2020;35(5):1026-36.
95. Watson D, Hahn R, Staines J. Storying special objects: Material culture, narrative identity and life story work for children in care. Qualitative Social Work. 2019;19(4):701-18.
96. Nuffield Family Justice Observatory. Modernising post-adoption contact: findings from a recent consultation 2021 [cited 2023 12.02.23]. Available from: https://www.nuffieldfjo.org.uk/wp-content/uploads/2021/08/nfjo_report_adoption_connections_20210913v2.pdf.
97. Atwell A. Working with children with a disability. In: Ryan T, Walker, R. editor. Life Story Work why, what, how and when. UK: CoramBAAF Adoption and Fostering Academy. ; 2016. p. 97-102.
98. Walker R, Ryan, T. Why do life story work? In: Ryan T, Walker, R. editor. Life story work Why, what, how and when UK: CoramBAAF Adoption and Fostering Academy; 2016. p. 4-17.
99. Gray S, Hahn R, Cater K, Watson D, Meineck C, Metcalfe T. Trove: A Digitally Enhanced Memory Box for Looked after and Adopted Children. Proceedings of the 18th ACM International Conference on Interaction Design and Children2019. p. 458-63.
100. Shepherd E. Good practice in record-keeping in children's social care: Research in Practice; 2022 [Available from: https://www.researchinpractice.org.uk/children/news-views/2022/february/good-practice-in-record-keeping-in-children-s-social-care/.
101. Nicholls E. Model answer. Community Care. 2003;1479:32-4.
102. Humphreys C, Kertesz, M. Making Records Meaningful: Creating an Identity Resource for Young People in Care. Australian Social Work. 2014;68(4):497-514.
103. Eenshuistra A, Harder, AT, Knorth EJ. One size does not fit all: A systematic review of training outcomes on residential youth care professionals' skills. Children and Youth Services Review 2019;103:135-47.
104. Steinberg L, Monahan KC. Age differences in resistance to peer influence. Developmental Psychology. 2007;43(6):1531-43.
105. Saluja S, Silverstein A, Mukhopadhyay, S. et al., Using the Consolidated Framework for Implementation Research to implement and evaluate national surgical planning. BMJ Glob Health. 2017;2(2):e000269.
106. McKeown J, A. Clarke, and J. Repper, Life story work in health and social care: systematic literature review. Life story work in health and social care: systematic literature review. Journal of Advanced Nursing,
. 2006; 55:237-47.
107. CEBM. Oxford Centre for Evidence-based Medicine - Levels of Evidence (March 2009) 2009 [Available from: https://www.cebm.net/2009/06/oxford-centre-evidence-based-medicine-levels-evidence-march-2009/.



For peer review only - http://bmjopen.bmj.com/site/about/guidelines.xhtml

108. Evans D. Hierarchy of evidence: a framework for ranking evidence evaluating healthcare interventions. Journal of Clinical Nursing. 2003;12(1):77-84.
109. Department of Heath & Department of Education. Transforming children and young people's mental health provision: Green paper 2017 [Available from: https://www.gov.uk/government/consultations/transforming-children-and-young-peoples-mental-health-provision-a-green-paper.
110. House of Commons Education Committee. Mental health and well-being of looked-after children. London, UK: House of Commons; 2016.
111. NICE. Child abuse and neglect: NICE guideline [NG76] 2017 [Available from: https://www.nice.org.uk/guidance/ng76.
112. Hoffmann TC, Glasziou, PP, Boutron I, et al., Better reporting of interventions: template for intervention description and replication (TIDieR) checklist and guide. BMJ. 2014;348:G1687.
113. Pinnock H, Barwick, M., Carpenter, C.R., Eldridge, S., Grandes, G., Griffiths, C.J., Rycroft-Malone, J., Meissner, P., Murray, E., Patel A., Sheikh, A., Taylor, S.J.C. Standards for Reporting Implementation Studies (StaRI) Statement. BMJ. 2017;356:i6795.
114. Adoption and Children Act 2002, (2002).
115. Children Act 1989, (1989).
116. Guide to the Children's Homes Regulations including the quailty standards, (2015).
117. The Fostering Services (England) Regulations 2011, (2011).
118. Lilienfield SO. Psychological Treatments That Cause Harm. Perspectives on Psychological Science. 2007;2(1):53-70.

For peer review only



For peer review only - http://bmjopen.bmj.com/site/about/guidelines.xhtml

Author’s contributions

Contributors SH conceived the study with input from GW, RH, KW, EN, CD and JW. SH and EM and wrote the first draft of this paper, and GW, CD, RH, EN, RB, JW and KW provided criticism and refinement. All the authors have read and approved the final manuscript.

Funding

This project is funded by the National Institute for Health Research (NIHR) Research for Patient Benefit Programme (Grant Reference Number NIHR 201963).

Disclaimer

The views expressed in this publication are those of the author(s) and not necessarily those of the NIHR or the Department of Health and Social Care.

Competing interests

SH offers consultancy services in social media and Digital Life Story Work via www.digitallifestorywork.co.uk. KW is the CEO of The Fostering Network. The remaining authors declare that they have no known competing financial interests or personal relationships that could have appeared to influence the work reported in this paper.

For peer review only

# Table 1: Summary of methods

| Step | Aim | Approach |
|---|---|---|
| Step 1: Develop an Initial Programme Theory (iPrT) | To identify existing relevant theories that provide explanations of why and how Adolescent-Focused Low-Intensity Life Story Work (AF-LI-LSW) approaches work (or are thought to work), in what contexts they work, to what extent and for whom. | Exploratory searches (CD)<br><br>Project team knowledge and consultation with stakeholders (All) |
| Step 2: Evidence search | To perform a comprehensive literature search to identify data to develop and refine the iPrT. | Electronic database searches across multiple databases (see supplementary file 1)<br><br>Targeted Google searches to retrieve grey literature produced by local authorities (CD)<br><br>Citation tracking (EM)<br><br>Project team knowledge (All)<br><br>Email alert to capture new literature (CD) |
| Step 3: Document selection, data organisation and extraction | To select eligible documents that could include relevant data to contribute to the development and refinement of the Programme Theory (PrT). To describe selected documents and to extract and code relevant data. The full set of extracted data are available on request. | Screen title and abstract and then full text against the inclusion and exclusion criteria (See Table 2: Inclusion and exclusion criteria (EM & SH)<br><br>Included documents selected based on the rigour and relevance of documents.<br><br>10% random sample of documents screened in duplicate with discrepancies resolved through group discussion (EM & SH)<br><br>Key characteristics of selected documents were extracted into Excel (EM)<br><br>Full texts uploaded to NVivo and relevant data to the research question and review focus were coded (EM) |



For peer review only - http://bmjopen.bmj.com/site/about/guidelines.xhtml

| | | |
|---|---|---|
| Step 4: Evidence synthesis | To apply a realist logic of analysis to extracted data to develop Context, Mechanism, Outcome Configurations (CMOCs) about how AF-LI-LSW improves the mental health support available to adolescents looked-after by social care. CMOCs are central to realist analysis, offering an explanation of the relationships between the conditions an intervention is delivered, the mechanisms by which the intervention works and the outcomes that are produced when delivered within those particular contexts. | The coded data was closely inspected, and interpretations were made about whether evidence was functioning as a context, mechanism, or outcome and which CMOC the evidence belonged to (EM & SH)<br><br>Cross document comparisons were made to build CMOCs, as documents did not always contain evidence that supported the development of CMOCs as evidence for each element of the configuration could not always be found in one document (EM & SH)<br><br>As the CMOCs were created and refined, judgements were made on how they related to each other in consultation with the project team and where relevant stakeholders (All) |
| Step 5: Development of consolidated PrT and drawing conclusions | To develop a consolidated programme theory containing the CMOCs | The programme theory was refined iteratively through the development of CMOCs and consultation with stakeholders and project team discussions (All)<br><br>Judgements were made on the plausibility and coherence of the developing PrT (All)<br><br>The consolidated PrT was written and shared with stakeholders and final amendments were made based on their consultation (All) |



For peer review only - http://bmjopen.bmj.com/site/about/guidelines.xhtml

Improving the mental health and mental health support available to adolescents looked-after by social care via adolescent-focused low-intensity life story work: A realist review

# Figure 1: PRISMA

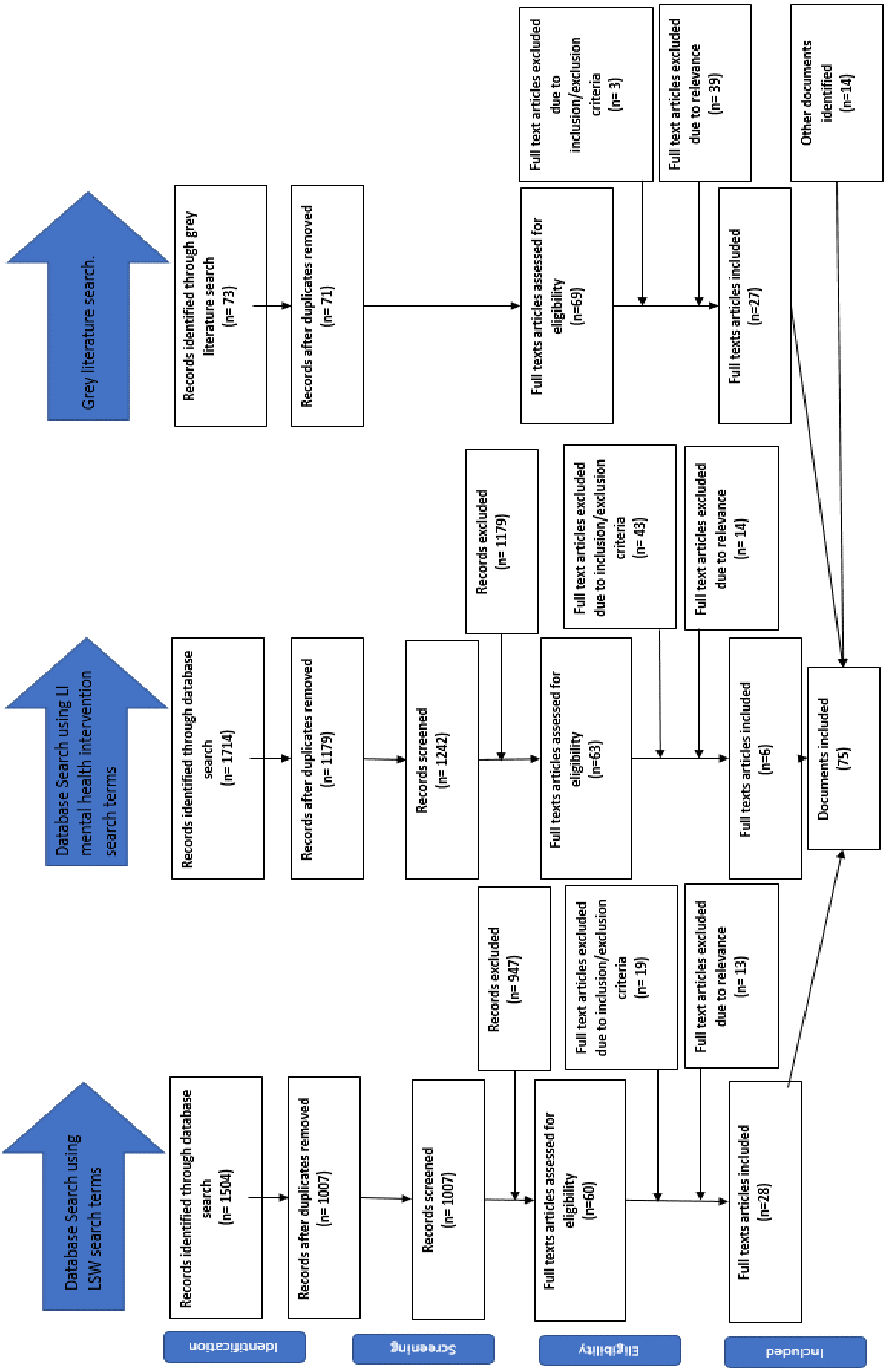

Improving the mental health and mental health support available to adolescents looked-after by social care via adolescent-focused low-intensity life story work: A realist review

# Figure 2: initial Programme Theory

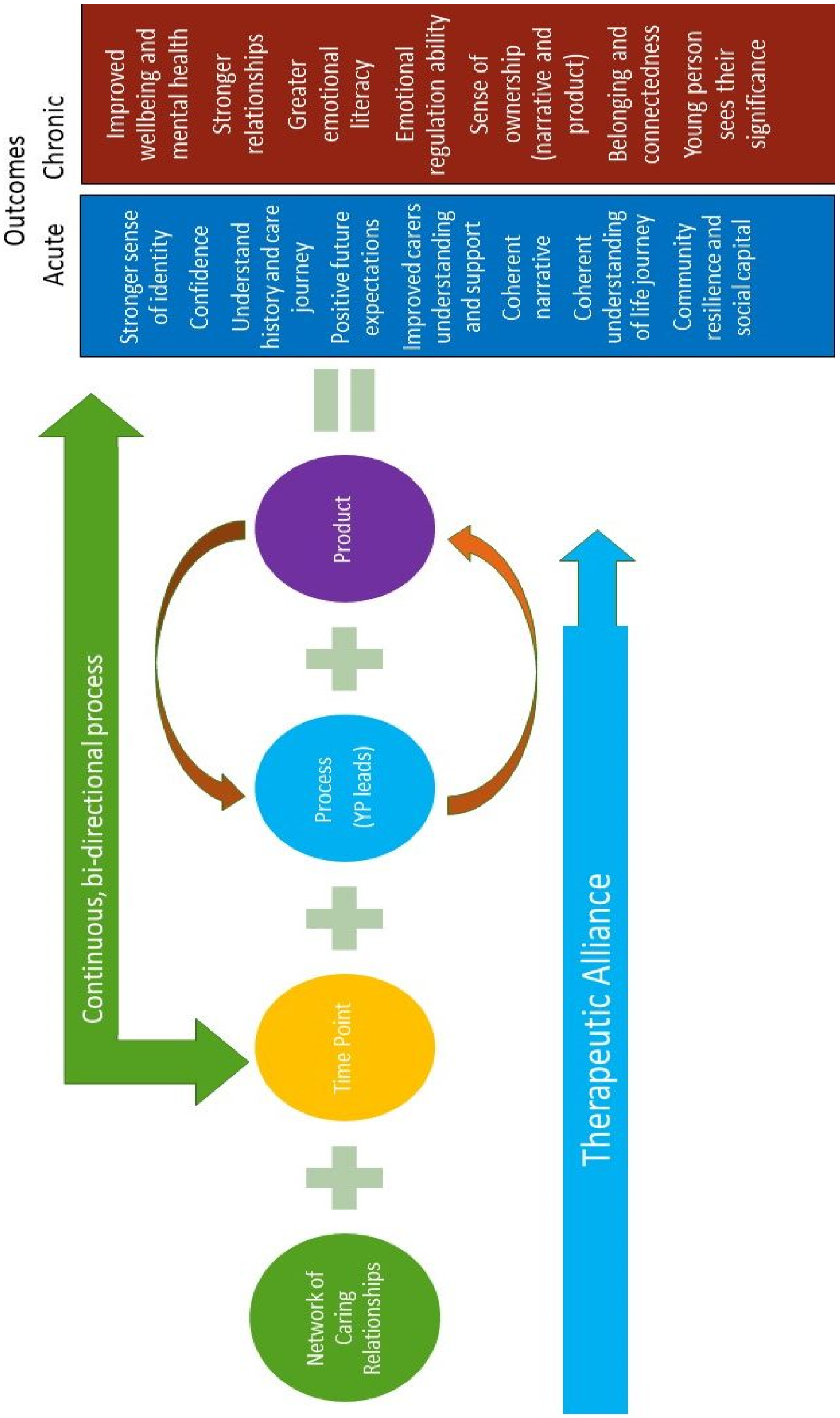

Improving the mental health and mental health support available to adolescents looked-after by social care via adolescent-focused low-intensity life story work: A realist review

# Figure 3: Consolidated Programme Theory

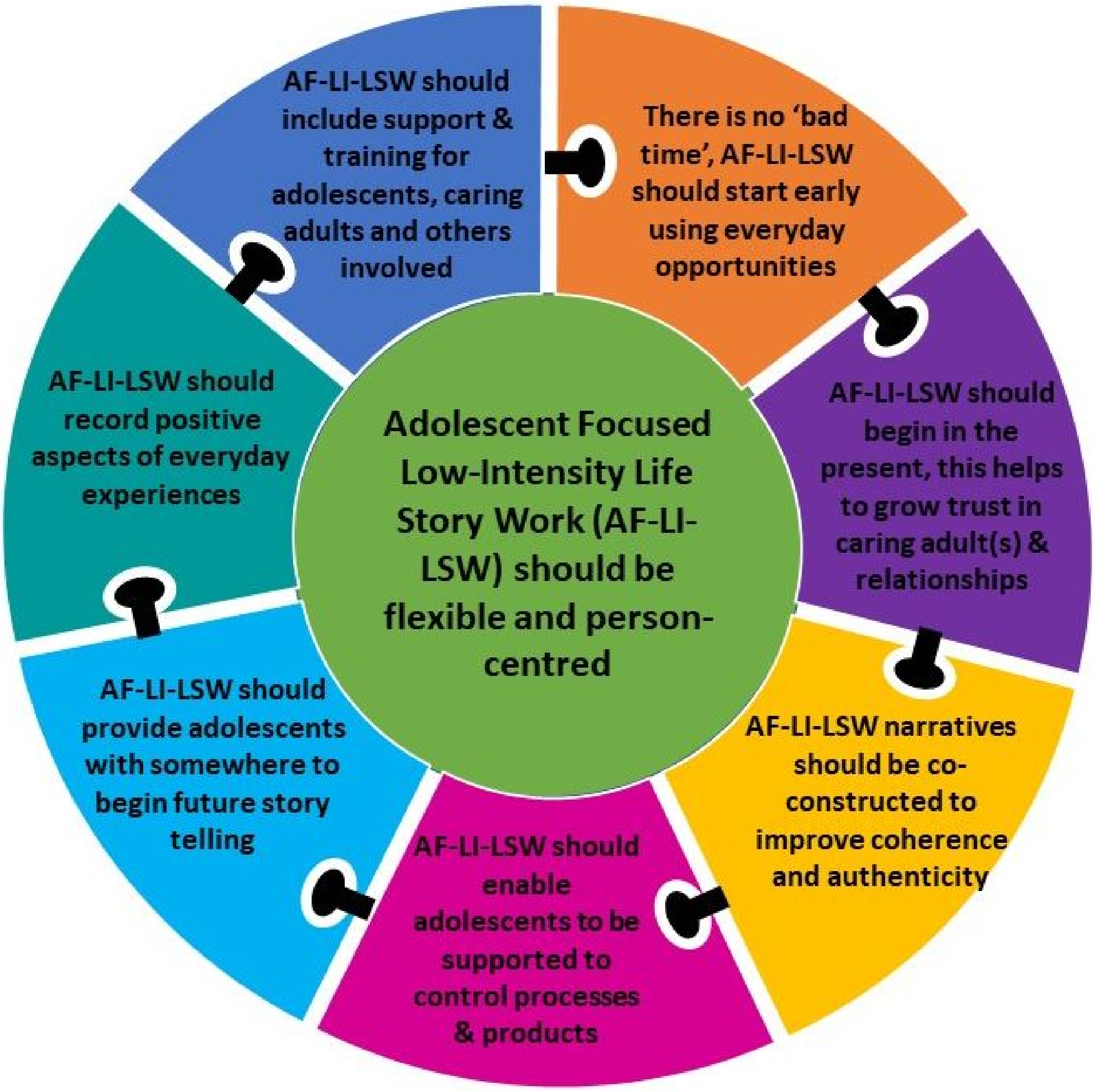

Improving the mental health and mental health support available to adolescents looked-after by social care via adolescent-focused low-intensity life story work: A realist review

# Supplementary File 1

**Data sources searched**

Electronic database searches were performed between December 2021 and March 2022. Eight databases were searched: MEDLINE, Embase, PsychINFO, Sociology Collection (Proquest), CINAHL, CDAS, Web of Science and Social Care Online. To retrieve relevant PhD theses, we also searched EThoS (British Library) and Proquest Dissertations and Theses. Targeted Google searches were performed to retrieve grey literature produced by local authorities and further documents were obtained through citation tracking and team members knowledge.

For peer review only

Improving the mental health and mental health support available to adolescents looked-after by social care via adolescent-focused low-intensity life story work: A realist review

# Supplementary File 3: Detailed summary of the included documents

| Authors | Year | Country | Title | Study Design/Methods | Sample/Setting | Objectives |
|---|---|---|---|---|---|---|
| ARCBOX | | UK | Digital Life Story | | Adoption | To describe a digital platform for adopted young people to collect memories throughout their life from the past to the present day. |
| Atwell | 2016 | UK | Working with children with a disability. | Book Chapter | | To discuss LSW with children and young people with special educational needs or disabilities (SEND). |
| Atwool | 2017 | New Zealand | Life Story Work Optional Extra or Fundamental Entitlement. | Commentary/ practice guidance | | To discuss LSW practice in New Zealand and the reasons why LSW is fundamental to young people with care experience. |
| Aust | 1981 | USA | Using the Life Story Book in Treatment of Children in Placement | Case Study | Foster Placement | To describe the use of life story books in foster placements using a case study example. |
| Aventin, Houston, & Macdonald | 2014 | UK | Utilising a computer game as a therapeutic intervention for youth in residential care Some preliminary findings on use and acceptability. | semi-structured interviews | Residential placement (N = 16 consisting of 8 young person and worker pairs) | To explore the accessibility and therapeutic impact of a computer-based intervention delivered to young people in residential placements by their key worker. |



For peer review only - http://bmjopen.bmj.com/site/about/guidelines.xhtml

Improving the mental health and mental health support available to adolescents looked-after by social care via adolescent-focused low-intensity life story work: A realist review

| | | | | | | |
|---|---|---|---|---|---|---|
| BASW | 2020 | UK | Recording in children's social work | Commentary/ practice guidance | | To discuss recording in social work providing ten recommendations for practice. |
| Baynes | 2008 | UK | Untold stories A discussion of life story work. | Commentary/ practice guidance | | To discuss LSW practice by social care professionals and the issues surrounding practice. |
| Bazalgette, Rahilly &Trevelyan | 2015 | UK | Achieving-emotional-wellbeing-for-looked-after children a whole system approach | Commentary/ practice guidance | | To discuss recommendations based on research for a whole system approach to improving the emotional wellbeing of young people with care experience by delivering preventative as opposed to crisis driven interventions, including LSW. |
| Beste & Richardson | 1981 | USA | Developing a Life Story Book Program for Foster Children | Commentary/ practice guidance | | To describe a training programme for foster carers to support young people to create a Life Story Book. |
| Bolton Council | | UK | Life Story Work 5 minute facts | Commentary/ practice guidance | Foster placement | To provide guidance for carrying out LSW within foster placements. |
| Braiden | 2016 | UK | Evaluation of a short training programme for foster carers | Case study | Foster placement | To discuss a training package for foster carers that provides skills around art and creative therapy they can engage in with the young person in their care. |
| Brookfield, Brown, & Reavey, | 2008 | UK | Vicarious and Post-memory Practices in Adopting Families: The Re-production of the Past through Photography and Narrative | Focus Groups | Adoption (n=12 adoptive parents) | To explore adoptive parents experience of supporting adoptive children with life story work. |
| Buchanan | 2014 | UK | The experience of life story work reflections of young | Thesis | Care leavers | To explore care leavers experiences of LSW, highlighting the importance of taking a person- |



For peer review only - http://bmjopen.bmj.com/site/about/guidelines.xhtml

Improving the mental health and mental health support available to adolescents looked-after by social care via adolescent-focused low-intensity life story work: A realist review

| | | | | | | |
|---|---|---|---|---|---|---|
| | | | people leaving care. A. Buchanan 2014 | | | centred approach, trusted relationships and involving birth relatives for successful LSW. |
| Connor, Sclare, Dunbar and Elliffe | 1985 | UK | Making a life story book | Case Study | Residential Placement | To discuss making a life story book with a young person in a residential placement, highlights topics of planning, relationship building and access to supervision. |
| Cook-Cottone & Beck | 2007 | USA | A Model for Life-Story Work Facilitating the Construction of Personal Narrative for Foster Children | Commentary/ practice guidance | Foster placement | To discuss life story work and theoretical frameworks around LSW. Introduces a model for life story work for children in foster care. |
| Davies & Hodges | 2017 | UK | Relationship renaissance the use of attachment-based narrative and metaphor in life story work | Case Study | Foster placement (n=3) | To describe an attachment based narrative approach to LSW presenting two case studies of the approach in practice. |
| Devenney | 2017 | UK | Pathway planning with unaccompanied young people leaving care Biographical narratives of past, present, and future | Interviews | unaccompanied care leavers (n=18) Social work professionals (n=12) | To explore how unaccompanied young people create biographical narratives of their past, present and future. |
| Eenshuistra, Harder, & Knorth | 2019 | Netherlands | One size does not fit all A systematic review of training outcomes on residential youth | Systematic Review | Residential placement | To review training programmes for residential placement workers. |



For peer review only - http://bmjopen.bmj.com/site/about/guidelines.xhtml

Improving the mental health and mental health support available to adolescents looked-after by social care via adolescent-focused low-intensity life story work: A realist review

| | | | | | | |
|---|---|---|---|---|---|---|
| | | | care professionals' skills | | | |
| Eldridge | 2018 | UK | Confiding in Others: A Qualitative Study Exploring the Experiences of Young people who have been in the Care System | Thesis | | To explore factors associated with confiding relationships and what elements are influential on an individual confiding in others. |
| Ferrier | 2011 | UK | Life story work in the context of attachment led care planning | Commentary/ practice guidance | Residential placement | To discuss a model of LSW that uses an attachment-based theoretical framework for carrying out LSW in residential placements. |
| Finlay | 2022 | UK | Meaningful natural mentoring relationship characteristics and informal therapeutic life space interactions for youth in care | Thesis – systematic review and interviews | Therapeutic care workers (n=8) | To explore factors that contribute to 'natural mentoring relationships' and trusting relationships with young people with care experience by looking at the perspectives of workers within a residential setting in UK. |
| Fitzharding e | 2008 | Australia | Adoption, resilience and the importance of stories The making of a film about teenage adoptees. | Commentary/ practice guidance | Adoption (n=6) | To discuss a group project where adopted young people work as a group to make a film about being adopted. |
| Fostering Sunderland | 2021 | UK | Foster Carer Handbook | Practice guidance | Foster Placement | To provide guidance to foster carers. |
| Furnivall & Grant | 2014 | UK | Trauma sensitive practice with children in care | Report | | To discuss the importance of social care practitioners being trauma informed to improve their practice when working with young people with care experience. |

Improving the mental health and mental health support available to adolescents looked-after by social care via adolescent-focused low-intensity life story work: A realist review

| | | | | | | |
|---|---|---|---|---|---|---|
| Gallagher & Green | 2012 | UK | In, out and after care Young adults' views on their lives, as children, in a therapeutic residential establishment. | Semi-structured interviews | Residential placement (n=16) | To explore young people with care experience views of their experience in residential care including their experience of LSW. |
| Gray, Hahn, Cater, Watson, Meineck, & Metcalfe. | 2019 | UK | Trove A Digitally Enhanced Memory Box for Looked after and adopted children | Conference paper | | To discuss Trove, a digital memory box used with in LSW, it's development, intended purpose and uses. |
| Gustavsson & MacEachron | 2008 | USA | Creating Foster Care Youth Biographies A Role for the Internet. | Commentary/ practice guidance | | To describe a digital LSW approach where digital records are made for young people with care experience. |
| Gutsche | 2013 | South Africa | Perceptions of social workers regarding life story work with children in child youth centres. | Thesis | Residential placement Social workers (n=6) | To explore social workers views in South Africa of LSW with young people living in South Africa's equivalent of residential placements. |
| Haight, Black, & Sheridan | 2010 | USA | A Mental Health Intervention for Rural, Foster Children from Methamphetamine-involved Families Experimental Assessment with | Randomised | Foster placement (n=23) | To evaluate the efficacy of a LSW intervention delivered by individuals from the community to young people living in foster placements. |



For peer review only - http://bmjopen.bmj.com/site/about/guidelines.xhtml

Improving the mental health and mental health support available to adolescents looked-after by social care via adolescent-focused low-intensity life story work: A realist review

| | | | | | | |
|---|---|---|---|---|---|---|
| | | | Qualitative Elaboration | | | |
| Hamilton | 2020 | UK | Life story approaches and relationships within residential child care A practice reflection. E. Hamilton | Case Study | Residential placement | To explore the importance of everyday interactions to support life stories for young people within residential care using a case study as an example. |
| Hammond | 2012 | UK | Exploring a role for digital technologies in life story work with adolescents in residential care a discourse analysis | Thesis | Residential placement (n=10) | To explore the use of novel digital technologies as tools to facilitate LSW with adolescents in residential placements. |
| Hammond & Cooper | 2013 | UK | Digital Life Story Work using technology to help young people make sense of their experience | Book | | To discuss digital LSW approaches that can be used in practice with young people with care experience. |
| Hammond | 2016 | UK | Digital Life Story Work in Action. | Book chapter | | To discuss digital LSW approaches that can be used in practice with young people with care experience. |
| Hammond, Cooper & Jordan | 2021 | UK | Mental health, identity and informal education opportunities for adolescents with experience of living in state care: a role for digital storytelling, | Commentary/ practice guidance | Residential placement | To discuss digital storytelling, how both formal and informal educators can play a role in supporting young people with care experiences use of digital technologies within LSW. |



For peer review only - http://bmjopen.bmj.com/site/about/guidelines.xhtml

Improving the mental health and mental health support available to adolescents looked-after by social care via adolescent-focused low-intensity life story work: A realist review

| | | | | | | |
|---|---|---|---|---|---|---|
| Hanna | 2007 | USA | Preparing School Age Children for Adoption | Interviews | Adoption - social care professionals (n=26) and adoptive parents (n=55) | To explore how older children are prepared for adoption, an included topic discussed is life story work/books. |
| Happer, McCreadie, & Aldgate | 2006 | UK | Celebrating Success: What Helps Looked After Children Succeed | Book | | To explore young people with care experiences views on their successes and the factors that promoted success for them while being looked after by the local authority. |
| Hertfordshire Children's Specialist and Safeguarding Services | 2022 | UK | Direct Work and Life Story Work with Children and young people | Commentary/ practice guidance | | To provide guidance on how LSW should be carried out in practice making a distinction between adoptive and foster placements and how these different contexts impact LSW. |
| Hills | 2022 | UK | Ensuring life stories are at the centre of direct work | Podcast | | To discuss a model of practice that ensures LSW is woven into all interactions with young people in contact with social care services. |
| Holody & Mäher | 1996 | USA | Using lifebooks with children in family foster care a here and now process model | Commentary/ practice guidance | Foster placement | To discuss the 'here and now' model for supporting young people in foster placements with life story books using case examples. |
| Hooley | 2015 | UK | Identifying perspectives on life story work with looked after and adopted children | Thesis | Clinical psychologists and therapists, social work professionals, foster carers, | To explore how different aspects of LSW that are important to different groups of people using Q-Methodology. |



For peer review only - http://bmjopen.bmj.com/site/about/guidelines.xhtml

Improving the mental health and mental health support available to adolescents looked-after by social care via adolescent-focused low-intensity life story work: A realist review

| | | | | | | |
|---|---|---|---|---|---|---|
| | | | | | adoptive parents, care leavers (N= 29) | |
| Hooley,Stokes & Combes | 2016 | UK | Life story work with looked after and adopted children how professional training and experience determine perceptions of its value | mixed methods | Clinical psychologists and therapists, social work professionals, foster carers, adoptive parents, care leavers (N= 29) | To investigate which aspects of LSW are important to different groups of people using Q-Methodology. |
| Hoyle, Shepherd, Lomas, & Flinn | 2020 | UK | Recordkeeping and the life long memory and identity needs of care experienced children and young people | Interviews | N=21 care leavers | To explores record keeping practices in social care settings. |
| Hughes | 2013 | UK | Life-story work a journey into a child's world Seen and Heard | Commentary/ practice guidance | | To discuss different LSW models using case examples to describe their uses |
| Humphreys, & Kertesz | 2014 | Australia | Making Records Meaningful Creating an Identity Resource for young people in care | Survey and focus groups | Survey n=37 young people with care experience by foster care and | To explore record keeping practices in Australia to support identity from key stakeholders. |



For peer review only - http://bmjopen.bmj.com/site/about/guidelines.xhtml

Improving the mental health and mental health support available to adolescents looked-after by social care via adolescent-focused low-intensity life story work: A realist review

| | | | | | | |
|---|---|---|---|---|---|---|
| | | | | | residential placement. Focus group n=5 social care workers, n=2 young people current care experience, n=4 care leavers. | |
| Känkänen & Bardy | 2014 | Norway | Life stories and arts in child welfare enriching communication | Commentary/ practice guidance | Residential placement | To discuss the authors experiences of using participatory arts within LSW in residential placements. |
| Lucas, Matthews, L. Brady, Breguet, & Parson | 2020 | Australia | Therapeutic-Life-Story-Work-Barwon-Pilot-Evaluation | Interim report | | To evaluate the implementation of a therapeutic LSW model in Australia. |
| Malik | 2005 | USA | Application of attachment theory for training foster parents a model program | Thesis | Foster carers | To discuss the development of a training programme for foster carers. |
| Monson | 2020 | Australia | Promoting mental health in out of home care in Australia | focus group and interviews | N=14 young people with care experience | To explore the experience of young people with care experience in Australia on what promotes positive mental health. |
| Neil, & Beek | 2020 | UK | Respecting Children's Relationships and | Book Chapter | Adoption | To discuss how adopted young people can be supported to maintain significant relationships while outlining key principles for supporting transitions for |



For peer review only - http://bmjopen.bmj.com/site/about/guidelines.xhtml

Improving the mental health and mental health support available to adolescents looked-after by social care via adolescent-focused low-intensity life story work: A realist review

| | | | | | | |
|---|---|---|---|---|---|---|
| | | | Identities in Adoption. | | | adoptive families, drawing on the “Secure Base Model”. |
| NICE | 2021 | UK | Looked after children and young people NICE guideline 2021 | Quality standard | | To provide the NICE guidelines for LSW with young people with care experience, including recommendations for how LSW should be delivered and what should be included. |
| Nicholls | 2003 | UK | Model answer - Community Care | Commentary/ practice guidance | | To discuss a model of LSW that focuses on a collaborative approach to securing memories for young people with care experience. |
| NSPCC | 2022 | UK | Life Story Work _ NSPCC Learning 2022 | Commentary/ practice guidance | | To discuss the NSPCC's strengths-based model of LSW that is delivered to young people with care experience. |
| Nuffield Family Justice Observator y | 2021 | UK | Modernising post adoption contact findings from recent consultation | Report | Adoption n=80 | To discuss findings of a consultation with organisations, birth families, adoptive families, young people, regional and voluntary adoption agencies on post-adoption contact with birth relatives |
| Pakrosnis, & Cˇepukiene | 2011 | Lithuani a | Outcomes of Solution-Focused Brief Therapy for Adolescents in Foster Care and Health Care Settings. | Matched pairs design | Foster and health care placement N=139 | To evaluate the efficacy of solution focused brief therapy for adolescents in Foster Care and Health Care Settings. |
| Peake | 2009 | UK | Life story work A resource for foster carers, residential social workers, adoptive parents, and kinship carers, to support this work. | Booklet | | To provide guidance for foster carers and social care professionals to carry out LSW with young people with care experience. |



For peer review only - http://bmjopen.bmj.com/site/about/guidelines.xhtml

Improving the mental health and mental health support available to adolescents looked-after by social care via adolescent-focused low-intensity life story work: A realist review

| | | | | | | |
|---|---|---|---|---|---|---|
| Sanders | 2020 | UK | Care experienced children and young people's mental health | Literature review | | To review the factors that impact the mental health of young people with care experience. |
| Shepard | 2022 | UK | Good practice in record-keeping in children's social care | Commentary/ practice guidance | | To discuss record keeping practices in children's social care including the development of a digital tool for care experienced young people to keep a diary to preserve their memories. |
| Shotton | 2012 | UK | "Remember when…" Exploring the experiences of looked after children and their carers in engaging in collaborative reminiscence | Thesis | | To explore the experience of foster carers and looked after children using the memory store approach. |
| Shotton | 2013 | UK | Remember when...' exploring the experiences of looked after children and their carers in engaging in collaborative reminiscence | semi-structured interviews | Foster placement (N=5 carers, N=4 young people) | To explore carers and young people's experiences of using the memory store approach to LSW using semi-structured interviews and a board game approach using Interpretative Phenomenological Analysis. |
| Shotton | 2010 | UK | Telling different stories The experience of foster adoptive carers in carrying out collaborative memory work with children. | Interviews | Adoptive parents and foster carers (n=5) | To explore adoptive and foster carers experience using the memory story approach to LSW. |



For peer review only - http://bmjopen.bmj.com/site/about/guidelines.xhtml

Improving the mental health and mental health support available to adolescents looked-after by social care via adolescent-focused low-intensity life story work: A realist review

| | | | | | | |
|---|---|---|---|---|---|---|
| Steenbakkers, van der Steen, & Grietens, | 2016 | Netherlands | 'To talk or not to talk?': Foster youth's experiences of sharing stories about their past and being in foster care | Interviews | Foster placement (n=13) | To explore young people's experiences of foster care in Netherlands. |
| Teodorczuk, Guse, & du Plessis, | 2018 | South Africa | The effect of positive psychology interventions on hope and well-being of adolescents living in a child and youth care centre. | Matched pairs design | Adolescents (n=29) | To investigate the efficacy of a positive psychology intervention on hope and wellbeing of young people with care experience in South Africa. |
| Together for Children Sunderland | 2019 | UK | Foster Carer Handbook 2019 together for children Sunderland | Commentary/ practice guidance | Foster placement | To give guidance to foster carers. |
| Walker, & Ryan | 2016 | UK | Working with black and minority ethnic children | Book chapter | | To discuss LSW with black and minority ethnic children. |
| Walker, & Ryan | 2016 | UK | Why do life story work? | Book chapter | | To discuss why LSW is carried out with young people with care experience. |
| Ward | 2002 | UK | Opportunity led work maximising the possibilities for therapeutic communication in everyday interactions. | Commentary/ practice guidance | | To discuss the concept of "opportunity led working', a framework to view everyday interactions as having therapeutic potential. |



For peer review only - http://bmjopen.bmj.com/site/about/guidelines.xhtml

Improving the mental health and mental health support available to adolescents looked-after by social care via adolescent-focused low-intensity life story work: A realist review

| | | | | | | |
|---|---|---|---|---|---|---|
| Watson , Hahn, & Staines | 2020 | UK | Storying special objects Material culture, narrative identity and life story work for children in care. | Interviews | Social care professional s (n=9) | To explore social care professional's perspectives of the importance of material objects in LSW to support young people with care experience identity construction. |
| Watson, Latter, & Bellew | 2015 | UK | Adopted children and young people's views on their life storybooks The role of narrative in the formation of identities. | Interviews | Adopted young people (n=20) | To explore adopted children and young people's views on their life story books. |
| Watson, Meineck & Lancaster | 2018 | UK | Adopted children's co-production and use of 'trove' (a digitally enhanced memory box) to better understand their care histories through precious objects. | Interviews | Adopted young people (n=10) | To explore adopted young people's experience of trialling the prototype Trove, a digital approach to facilitating LSW. |
| Watson, Staples, & Riches | 2021 | UK | 'We need to understand what's going on because it's our life' using sandboxing to understand children and young people's everyday conversations about care | Interviews | Care experienced children and young people N = 11 (5 adopted, 6 care leavers). | To explore children and young people's hopes and fears for their everyday conversations with carers about their care experience. |



For peer review only - http://bmjopen.bmj.com/site/about/guidelines.xhtml

Improving the mental health and mental health support available to adolescents looked-after by social care via adolescent-focused low-intensity life story work: A realist review

| | | | | | | |
|---|---|---|---|---|---|---|
| Watson, Latter & Bellew | 2015 | UK | Adopters' views on their children's life story books. | Focus groups and interviews | Adopters (n=40) | To explore adopters' views on their children's life story books. |
| Watts | 2021 | UK | Relationships, reviews and recording Developing practice for children in care. | Focus Groups and interviews | Social care professionals (n=42), foster carers (n=10) and young people with care experience (n=4). | To explore stakeholders views on reviews and recording practices within social care including The Me and My World model introduced to Brighton and Hove City Council. |
| Willis, & Holland | 2009 | UK | Life story work Reflections on the experience by looked after young people | semi-structured interviews | Young people with care experience (n=12) | To explore young people with care experiences views on LSW. |
| Wood | 2019 | UK | West Sussex Children's improvement board. Performance report against the practice improvement plan improving quality of life story work. | Report | | To report findings from an audit of West Sussex children's services LSW practice. |
| Wood, & Selwyn | 2017 | UK | Looked after children and young people's views on what matters to their subjective well-being. | Focus groups | Young people with care experience (n=140) | To explore and identify the factors that children and young people identify as being indicative of subjective wellbeing. |



For peer review only - http://bmjopen.bmj.com/site/about/guidelines.xhtml

Improving the mental health and mental health support available to adolescents looked-after by social care via adolescent-focused low-intensity life story work: A realist review

References

ARCBOX. (2022). *Digital Life Story ARCBOX* ARC Adoption North East. https://www.arcadoptionne.org.uk/life-story

Atwell, A. (2016). Working with children with a disability. In R. Walker & T. Ryan (Eds.), *Life Story Work why, what, how and when* (pp. 97-102). CoramBAAF Adoption and Fostering Academy.

Atwool, N. (2017). Life story work: Optional extra or fundamental entitlement? *Child Care in Practice*, *23*(1), 64-76. https://www.tandfonline.com/doi/full/10.1080/13575279.2015.1126228

Aust, P. H. (1981). Using the Life Story Book in Treatment of Children in Placement. *Child Welfare*, *60*(8), 535-536,553-560. https://www.jstor.org/stable/45393827

Aventin, A., Houston, S., & Macdonald, G. (2014). Utilising a computer game as a therapeutic intervention for youth in residential care: Some preliminary findings on use and acceptability. *Children and Youth Services Review*, *47*, 362-369. http://ovidsp.ovid.com/ovidweb.cgi?T=JS&PAGE=reference&D=psyc11&NEWS=N&AN=2014-55927-021

BASW. (2020). *Recording in childrens social work*. https://www.basw.co.uk/system/files/resources/basw_recording_in_childrens_social_work_aug_2020.pdf

Baynes, P. (2008). Untold stories: A discussion of life story work. *Adoption & Fostering*, *32*(2), 43-49. https://doi.org/10.1177%2F030857590803200206

Bazalgette, T. R., G. Trevelyan. (2015). Achieving emotional wellbeing for looked after children a whole system approach. .

Beste, H. M., & Richardson, R. G. (1981). Developing a Life Story Book Program for Foster Children. *Child Welfare*, *60*(8), 529-534. https://www.jstor.org/stable/45393826

Bolton, C. (2022). *Life Story Work 5 minute facts* https://www.bolton.gov.uk/downloads/file/2299/life-story-work

Braiden, S. (2016). Evaluation of a short training programme for foster carers. *Scottish Journal of Residential Child Care*, *15*(2). https://www.celcis.org/application/files/2016/2308/5288/004._2016_Vol_15_2_Braiden_Evaluation_for_foster_carers.pdf

Brookfield, H., Brown, S. D., & Reavey, P. (2008). Vicarious and post-memory practices in adopting families: The re-production of the past through photography and narrative. *Journal of Community & Applied Social Psychology*, *18*(5), 474-491. https://onlinelibrary.wiley.com/doi/abs/10.1002/casp.960

Buchanan, A. (2014). *The experience of life story work: reflections of young people leaving care* Cardiff University]. EThOS. https://orca.cardiff.ac.uk/64531/

Connor, T., Sclare, I., Dunbar, D., & Elliffe, J. (1985). Making a life story book. *Adoption & Fostering*, *9*(2), 32-46. https://dx.doi.org/10.1177/030857598500900210

Cook-Cottone, & Beck. (2007). A Model for Life-Story Work: Facilitating the Construction of Personal Narrative for Foster Children. *Child Adolesc Ment Health*, *12*(4), 193-195. https://doi.org/10.1111/j.1475-3588.2007.00446.x



For peer review only - http://bmjopen.bmj.com/site/about/guidelines.xhtml

Improving the mental health and mental health support available to adolescents looked-after by social care via adolescent-focused low-intensity life story work: A realist review

Davies, M., & Hodges, J. (2017). Relationship renaissance: the use of attachment-based narrative and metaphor in life story work. *Adoption & Fostering*, *41*(2), 131-141. https://doi.org/10.1177%2F0308575917702831

Devenney, K. (2017). Pathway planning with unaccompanied young people leaving care: Biographical narratives of past, present, and future. *Child & Family Social Work*, *22*(3), 1313-1321. https://onlinelibrary.wiley.com/doi/full/10.1111/cfs.12348

Eenshuistra, A., Harder, A. T., & Knorth, E. J. (2019). One size does not fit all: A systematic review of training outcomes on residential youth care professionals' skills. *Children and Youth Services Review*, *103*, 135-147. https://doi.org/10.1016/j.childyouth.2019.05.010

Eldridge, J. (2018). *Confiding in Others: A Qualitative Study Exploring the Experiences of Young people who have been in the Care System* University of Surrey Guildford].

Ferrier, M. (2011). Life story work in the context of attachment-led care planning. *Scottish Journal of Residential Child Care*, *10*(1). https://www.celcis.org/application/files/4516/2316/8859/2011_Vol_10_1_Ferrier_Life_story_work.pdf

Finlay, J. (2022). *Meaningful Natural Mentoring Relationship Characteristics and informal therapeutic life spcae interactions in youth care* Canterbury Christ Church University].

Fitzhardinge, H. (2008). Adoption, Resilience and the Importance of Stories: The Making of a Film about Teenage Adoptees. *Adoption & Fostering*, *32*(1), 58-68. https://doi.org/10.1177/030857590803200108

Furnivall, J., & Grant, E. (2014). *Trauma sensitive practice with children in care* (Insights: evidence summaries to support social services in Scotland, Issue. https://www.iriss.org.uk/sites/default/files/iriss-insight-27.pdf

Gallagher, B., & Green, A. (2012). In, out and after care: Young adults' views on their lives, as children, in a therapeutic residential establishment. *Children and Youth Services Review*, *34*(2), 437-450. https://dx.doi.org/10.1016/j.childyouth.2011.11.014

Gray, S., Hahn, R., Cater, K., Watson, D., Meineck, C., & Metcalfe, T. (2019). *Trove: A Digitally Enhanced Memory Box for Looked after and Adopted Children* Proceedings of the 18th ACM International Conference on Interaction Design and Children, https://www.researchgate.net/publication/333628573_Trove_A_Digitally_Enhanced_Memory_Box_for_Looked_after_and_Adopted_Children/link/5d31cbc4299bf1995b382ab3/download

Gustavsson, N., & MacEachron, A. (2008). Creating Foster Care Youth Biographies: A Role for the Internet. *Journal of Technology in Human Services*, *26*(1), 45-55. https://www.tandfonline.com/doi/abs/10.1300/J017v26n01_03

Gutsche, K. H. (2013). *Perceptions of social workers regarding life story work with children in child youth centres* North-West University].

Haight, W., Black, J., & Sheridan, K. (2010). A Mental Health Intervention for Rural, Foster Children from Methamphetamine-involved Families: Experimental Assessment with Qualitative Elaboration. *Child Youth Serv Rev*, *32*(10), 1146-1457. https://doi.org/10.1016/j.childyouth.2010.06.024

Hamilton, E. (2020). Life story approaches and relationships within residential child care: A practice reflection. *Relational Child and Youth Care Practice*, *33*(1),



For peer review only - http://bmjopen.bmj.com/site/about/guidelines.xhtml

Improving the mental health and mental health support available to adolescents looked-after by social care via adolescent-focused low-intensity life story work: A realist review

87. https://www.celcis.org/application/files/8316/2307/7669/2019_Vol_18_No__2_Hamilton_E_A_practice_reflection.pdf

Hammond, S. (2012). *Exploring a role for digital technology in life story work with adolescents in residential care: a discourse analysis* University of East Anglia]. EThOS. https://ueaeprints.uea.ac.uk/id/eprint/39133/

Hammond, S. (2016). Digital Life Story Work in Action. In R. Walker & T. Ryan (Eds.), *Life story work Why, what, how and when* (pp. pp.106-119). CoramBAAF Adoption and Fostering Academy.

Hammond, S., & Cooper, N. (2013). *Digital Life Story Work: Using technology to help young people make sense of their experience.* (1 ed.). British Association of Adoption and Fostering.

Hammond, S. P., Cooper, N. J., & Jordan, P. (2021). Mental health, identity and informal education opportunities for adolescents with experience of living in state care: a role for digital storytelling. *Cambridge Journal of Education*, *51*(6), 713-732. https://doi.org/10.1080/0305764x.2021.1919057

Hanna, M. D. (2007). Preparing school age children for adoption: perspectives of successful adoptive parents and caseworkers. *Adoption Quarterly*, *10*(2), 1-32. https://www.tandfonline.com/doi/abs/10.1300/J145v10n02_01

Happer, H., McCreadie, J., & Aldgate, J. (2006). *Celebrating Success: What Helps Looked After Children Succeed*. Social Work Inspection Agency.

HCC. (2022). *Direct Work and Life Story Work with Children and Young People*. Hertfordshire County Council. Retrieved 12.08.22 from https://hertschildcare.proceduresonline.com/p_direct_life_story.html

Hills, L. (2022). In *Ensuring children's life stories are at the centre of direct work.*

Holody, R., & Mäher, S. (1996). Using lifebooks with children in family foster care a here and now process model. *Child Welfare*, *75*(4), 321-335.

Hooley, K., Stokes, L., & Combes, H. (2016). Life story work with looked after and adopted children: how professional training and experience determine perceptions of its value. *Adoption & Fostering*, *40*(3), 219-233. https://doi.org/10.1177%2F0308575916661129

Hooley, K. C. (2015). *Identifying perspectives on life story work with looked-after and adopted children* Staffordshire University]. EThOS. http://eprints.staffs.ac.uk/2244/

Hoyle, V., Shepherd, E., Lomas, E., & Flinn, A. (2020). Recordkeeping and the life-long memory and identity needs of care-experienced children and young people. *Child & Family Social Work*, *25*(4), 935-945. https://doi.org/https://doi.org/10.1111/cfs.12778

Hughes, J. I. (2013). Life-story work: a journey into a child's world. *Seen and Heard*, *23*(1), 23-30.

Humphreys, C., & Kertesz, M. (2014). Making Records Meaningful: Creating an Identity Resource for Young People in Care. *Australian Social Work*, *68*(4), 497-514. https://doi.org/10.1080/0312407x.2014.960434

Känkänen, P., & Bardy, M. (2014). Life stories and arts in child welfare: enriching communication. *Nordic Social Work Research*, *4*(1), 37-51. https://www.tandfonline.com/doi/full/10.1080/2156857X.2013.781536

Lucas, J., Matthews, L., Brady, K., Breguet, R., & Parson, J. (2020). *Therapeutic Life Story Work Barwon Pilot Program: Interim Report 2020* [Research report].



For peer review only - http://bmjopen.bmj.com/site/about/guidelines.xhtml

Improving the mental health and mental health support available to adolescents looked-after by social care via adolescent-focused low-intensity life story work: A realist review


Malik, S. (2005). *Application of attachment theory for training foster parents: A model program* https://www.proquest.com/dissertations-theses/application-attachment-theory-training-foster/docview/305340219/

Monson, K., Moeller-Saxone, K., Humphreys, C., Harvey, C., & Herrman, H. (2020). Promoting mental health in out of home care in Australia. *Health Promot Int*, *35*(5), 1026-1036. https://doi.org/10.1093/heapro/daz090

Neil, E., & Beek, B. (2020). Resepecting Children's Relationships and Identities in Adoption. In *The Routledge Handbook of Adoption* (1st ed., pp. 77-89). Routledge.

NICE. (2021). *Looked after children and young people NICE guideline*. www.nice.org.uk/guidance/ng205

Nicholls, E. (2003). Model answer. *Community Care*(1479), 32-34. https://www.communitycare.co.uk/2003/07/03/model-answer/

NSPCC. (2022). *Life Story Work NSPCC Learning*. https://learning.nspcc.org.uk/services-children-families/life-story-work#heading-top

Nuffield Family Justice Observatory. (2021). *Modernising post-adoption contact: findings from a recent consultation*. https://www.nuffieldfjo.org.uk/wp-content/uploads/2021/08/nfjo_report_adoption_connections_20210913v2.pdf

Pakrosnis, R., & Cˇepukiene, V. (2011). Outcomes of Solution-Focused Brief Therapy for Adolescents in Foster Care and Health Care Settings. In C. Franklin, T. S. Trepper, E. E. McCollum, & W. J. Gingerich (Eds.), *Solution-Focused Brief Therapy: A Handbook of Evidence-Based Practice*. Oxford Scholarship Online.

Peake, A. (2009). Life Story Work. A resource for foster carers, residential social workers, adoptive parents, and kinship carers, to support this work. . In.

Sanders, R. (2020). *ESSS Outline: Care experienced children and young people's mental health*. https://doi.org/10.31583/esss.20201012

Shepard, E. (2022). *Good practice in record-keeping in children's social care*. Research in Practice. https://www.researchinpractice.org.uk/children/news-views/2022/february/good-practice-in-record-keeping-in-children-s-social-care/

Shotton, G. (2010). Telling different stories The experience of foster/adoptive carers in carrying out collaborative memory work with children. *Adoption & Fostering*, *34*(4), 61-68. https://doi.org/10.1177%2F030857591003400407

Shotton, G. (2012). *"Remember when...": exploring the experiences of looked after children and their carers in engaging in collaborative reminiscence* University of Newcastle upon Tyne]. EThOS. http://theses.ncl.ac.uk/jspui/handle/10443/1685

Shotton, G. (2013). 'Remember when...': exploring the experiences of looked after children and their carers in engaging in collaborative reminiscence. *Adoption & Fostering*, *37*(4), 352-367. https://doi.org/10.1177%2F0308575913508721

Steenbakkers, A., van der Steen, S., & Grietens, H. (2016). 'To talk or not to talk?': Foster youth's experiences of sharing stories about their past and being in foster care. *Children and Youth Services Review*, *71*, 2-9. https://doi.org/10.1016/j.childyouth.2016.10.008

Sunderland, F. (2021). Foster Carers Handbook. In.

Teodorczuk, K., Guse, T., & du Plessis, G. A. (2018). The effect of positive psychology interventions on hope and well-being of adolescents living in a




For peer review only - http://bmjopen.bmj.com/site/about/guidelines.xhtml

child and youth care centre. *British Journal of Guidance & Counselling*, *47*(2), 234-245. https://doi.org/10.1080/03069885.2018.1504880

Together for Children Sunderland. (2019). *Foster Carers Handbook April 2019*. https://www.togetherforchildren.org.uk/sites/default/files/2019-12/Foster%20Carer%20HandbookFinalDraft16052019%20-%20Updated%20April%202019.pdf

Walker, R., & Ryan, T. (2016a). Why do life story work? In R. Walker & T. Ryan (Eds.), *Life story work Why, what, how and when* (pp. 4-17). CoramBAAF Adoption and Fostering Academy.

Walker, R., & Ryan, T. (2016b). Working with black and minority ethnic children. In R. Walker & T. Ryan (Eds.), *Life story work Why, what, how and when* (pp. 77-84). CoramBAAF Adoption and Fostering Academy.

Ward, A. (2002). Opportunity led work maximising the possibilities for therapeutic communication in everyday interactions. *Therapeutic Communities*, *23*(2), 111-124.

Watson, Hahn, & Staines. (2020). Storying special objects: Material culture, narrative identity and life story work for children in care. *Qualitative Social Work: Research and Practice*, *19*(4), 701-718. https://doi.org/10.1177%2F1473325019850616

Watson, Latter, & Bellew. (2015a). Adopted children and young people's views on their life storybooks: The role of narrative in the formation of identities. *Children and Youth Services Review*, *58*, 90-98. https://dx.doi.org/10.1016/j.childyouth.2015.09.010

Watson, Latter, & Bellew. (2015b). Adopters' views on their children's life story books. *Adoption & Fostering*, *39*(2), 119. https://doi.org/10.1177%2F0308575915588723

Watson, Staples, & Riches. (2020). 'We need to understand what's going on because it's our life': Using sandboxing to understand children and young people's everyday conversations about care. *Children & Society*, *35*(5), 663-679. https://doi.org/10.1111/chso.12432

Watson, D., Meineck, C., & Lancaster, B. (2018). Adopted children's co-production and use of 'trove' (a digitally enhanced memory box) to better understand their care histories through precious objects. *Clinical child psychology and psychiatry*, *23*(4), 614-628. https://dx.doi.org/10.1177/1359104518776359

Watts, R. (2021). Relationships, reviews and recording: Developing practice for children in care. *Practice: Social Work in Action*, *33*(3), 207-222. https://www.tandfonline.com/doi/full/10.1080/09503153.2021.1889488

Willis, R., & Holland, S. (2009). Life story work: Reflections on the experience by looked after young people. *Adoption & Fostering*, *33*(4), 44-52. https://doi.org/10.1177%2F030857590903300406

Wood, J. (2019). West Sussex Children's imporvement board. Performance Report Against the Practice Improvement Plan Improving Quality of Life Story Work.

Wood, M., & Selwyn, J. (2017). Looked after children and young people's views on what matters to their subjective well-being. *Adoption & Fostering*, *41*(1), 20-34. https://doi.org/10.1177/0308575916686034



For peer review only - http://bmjopen.bmj.com/site/about/guidelines.xhtml

Improving the mental health and mental health support available to adolescents looked-after by social care via adolescent-focused low-intensity life story work: A realist review

# Supplementary File 2: Detailed summary of the CMOCs developed and underpinning data

| CMOC Label | CMOC | Supporting evidence | Example extracts |
|---|---|---|---|
| **CMOC1** | When low-intensity LSW is flexible and person-centred (C) better practice is achieved (O) because adaptions have been made relating to who, when, where, in what circumstances and how low intensity LSW is delivered to the adolescent (M). | (Buchanan, 2014) (Hammond, 2012) (HCC, 2022) (Holody & Mäher, 1996) (Hooley et al., 2016) (NICE, 2021) (Shotton, 2013) (Watson, Hahn, et al., 2020) (Watson et al., 2018) (Willis & Holland, 2009) | 'Take a flexible approach to life story work, and tailor it to the developmental age and needs of the looked-after child or young person' (NICE, 2021)<br><br>'The content of the LSW reported as being most helpful incorporated photos, drawings, speech bubbles, sentimental objects and opportunities for the young people to comment on their feelings and to be creative in the content. This type of child-led approach offered choice with respect to both content and format' (Buchanan, 2014)<br><br>'The findings suggest that it may not be important what form the work takes, as long as it is adapted to suit the child's interests and needs.' (Willis & Holland, 2009) |
| **CMOC2** | When everyday life experiences are preserved (C) it improves young people's ability to construct a coherent identity (O) because they have autobiographical memory cues available to re-visit (M). | (Atwool, 2017) (Buchanan, 2014) (Cook-Cottone & Beck, 2007) (Hammond, 2016) (Hammond et al., 2021) (Shotton, 2013) (Watson, Hahn, et al., 2020) (Peake, 2009) (Shotton, 2012) (Willis & Holland, 2009) (Gustavsson & MacEachron, | 'A tangible and visual record of the child's time in care maintained from the outset through the use of memory boxes and life story books is essential to ensure access to a coherent narrative.' (Watson, Hahn, et al., 2020) |



For peer review only - http://bmjopen.bmj.com/site/about/guidelines.xhtml

Improving the mental health and mental health support available to adolescents looked-after by social care via adolescent-focused low-intensity life story work: A realist review

| | | | |
|---|---|---|---|
| | | 2008) (Hooley, 2015) | 'Entries assist in helping the youth preserve memories. These entries can consist of a description of a birthday party, or a school event in which the youth played a role such as a play or sporting event. These stories may someday help the youth establish a stronger sense of self based on positive memories of their past.' (Gustavsson & MacEachron, 2008) |
| **CMOC3** | When significant events are preserved through recording and made available to an adolescent when they have contact with social care (C) this helps an adolescent construct a coherent understanding of their experiences (O) because memories are available to revisit and reflect upon (M). | (Hills, 2022) (Watson et al., 2015b) (Hoyle et al., 2020) (Together for Children Sunderland, 2019) (Sunderland, 2021) (Hammond, 2012) (Hammond & Cooper, 2013) | 'Life Story Work is very important to the child and will help them in childhood and in later life gain some understanding of their identity and background and help them understand and recall their life in care. As the child's carer it is essential that you keep items for the child that can later be used for life story work, such as mementos of special occasions, photographs (including school photos) special clothes or toys.' (Together for Children Sunderland, 2019) |
| **CMOC4** | When everyday life experiences are preserved with narrative accounts (C) the ability to construct a coherent identity improves (O) because of the availability of contextual information (M). | (Watson et al., 2015a) (Atwool, 2017; Lucas et al., 2020) | 'For many of the children the absence of a "story" was a source of criticism about their own book and we were regularly told about books that contained photographs but with little account of who was in the photos or how the photos contributed to the child's story.' (Watson et al., 2015a)<br><br>'The children appeared overall to value their books even though they could see the flaws |



For peer review only - http://bmjopen.bmj.com/site/about/guidelines.xhtml

Improving the mental health and mental health support available to adolescents looked-after by social care via adolescent-focused low-intensity life story work: A realist review

| | | | |
|---|---|---|---|
| | | | and were critical of these. A lack of narrative and the failure to capture multiple perspectives was a common theme.’ (Atwool, 2017) |
| **CMOC5** | If adolescents are not ready to engage in low-intensity LSW, caring adults in the adolescent’s support network should begin to collect and preserve everyday life experiences (C) this ensures adolescents have access to information about their care journey (O) because information is not lost or forgotten (M). | (HCC, 2022) (Ferrier, 2011) (Willis & Holland, 2009) (Cook-Cottone & Beck, 2007) (Atwool, 2017) (Hooley et al., 2016) (Watts, 2021) (Peake, 2009) (NICE, 2021) (Shotton, 2012) (Shotton, 2013) | ‘The gaps in records that several young people had, including from some foster placements, reinforce the importance of gathering photographs and other mementos at each stage of a child’s life, even if they do not want them immediately.’ (Willis & Holland, 2009)<br><br>Start life story work as soon as possible after the looked-after child or young person enters care, to support care placement and emotional stability, rather than as an intervention to deliver once placements are stable.’ (NICE, 2021)<br><br>‘Some children may not feel motivated to engage with the approach, though even if they show little or no interest the carer should continue to collect memories of the child’s time with them so that memories of that placement are safeguarded… Knowing that those memories have been safeguarded communicates to the child that their time is important and that they matter.’ (Shotton, 2012) |



For peer review only - http://bmjopen.bmj.com/site/about/guidelines.xhtml

Improving the mental health and mental health support available to adolescents looked-after by social care via adolescent-focused low-intensity life story work: A realist review

| | | | |
|---|---|---|---|
| **CMOC6** | When all caring adults within the adolescent's support network are actively involved in preserving memories and artefacts of everyday life experiences (C) it reduces the risk of there being gaps in the adolescent's autobiographical memory (O) because information is not lost (M). | (HCC, 2022) (Hills, 2022) (Atwool, 2017) (Bolton, 2022) (Watson, Hahn, et al., 2020) (Gustavsson & MacEachron, 2008) (Hammond, 2012) (Hammond et al., 2021) (Neil & Beek, 2020) (Brookfield et al., 2008) | 'Birth families, all workers involved in the child's journey should take responsibility and have a role in collecting memorabilia of all significant events/achievements for the child. This information should be recorded for the child, and any memorabilia given to the child (or held in safekeeping by the carers according to the child's age and understanding).' (HCC, 2022)<br><br>'All caregivers (including those offering short-term care) need to be equipped to support children in keeping an ongoing record of their time in care through the use of memory boxes, life story books and digital records.' (Atwool, 2017) |
| **CMOC7** | When adolescents do not have access to information about their everyday life experiences and care journey (C) they can become frustrated and angry (O) because they feel a lack of control over information about their life and their experiences (M). | (Hoyle et al., 2020) (Buchanan, 2014) (Watson, Staples, et al., 2020) | 'Their wanting information and clarity about their life was often exacerbated by their judgement that others knew more about their lives and families than they did. This was a source of frustration and anger for many.' (Buchanan, 2014)<br><br>'The common belief that others knew more than they did was often associated with a general feeling that the information others held was deliberately kept from them. A sense of information being kept secret, hidden or confused was evident, which understandably led to many feeling frustrated, |



For peer review only - http://bmjopen.bmj.com/site/about/guidelines.xhtml

Improving the mental health and mental health support available to adolescents looked-after by social care via adolescent-focused low-intensity life story work: A realist review

| | | | |
|---|---|---|---|
| | | | angry and mistrustful of others' (Buchanan, 2014) |
| **CMOC8** | When caring adults and adolescents share, preserve, and reflect on everyday life experiences (C) connection and rapport between them increases (O) because they spend quality one to one time together (M). | (Hammond et al., 2021) (Braiden, 2016) (Shotton, 2012) (Holody & Mäher, 1996) (Hooley, 2015) (NICE, 2021) (Shotton, 2012) (Malik, 2005) (Buchanan, 2014) (Watson, Staples, et al., 2020) (Hamilton, 2020) (Hooley et al., 2016) (Eldridge, 2018) (Shotton, 2013) (Aventin et al., 2014) | 'All the carers talked positively about how the approach had been good for helping them to spend quality time with the child. The carers felt closer to the child through using it, and thought that the child felt closer to them as well.' (Shotton, 2012)<br><br>'Life story work has the potential for building relationships (for example, by sharing joint activities).' (NICE, 2021)<br><br>'While it may be time consuming, it can also be very rewarding such that in the process of creating this narrative and recording these memories together, a special relationship develops whereby both the child and foster parent learn more about the foster child.' (Malik, 2005) |
| **CMOC9** | When caring adults and adolescents share, preserve and reflect on everyday life experiences (C) adolescent's self-worth increases (O) because the caring adult shows they want to spend time with them, and their experiences are valuable (M). | (Malik, 2005) (Shotton, 2012) (Shotton, 2013) (Watts, 2021) (Peake, 2009) (Hooley, 2015) | 'By taking the time to complete this project, the caregiver conveys to the child that he or she is important and worth the time to get to know.' (Malik, 2005)<br><br>'Evidence within the store of children's particular preferences helps them to see that their opinions matter and are worth recording and talking about, thus contributing to their |



For peer review only - http://bmjopen.bmj.com/site/about/guidelines.xhtml

Improving the mental health and mental health support available to adolescents looked-after by social care via adolescent-focused low-intensity life story work: A realist review

| | | | |
|---|---|---|---|
| | | | sense of identity and self-worth' (Shotton, 2013) |
| **CMOC10** | When caring adults and adolescents share, preserve, and reflect on everyday life experiences (C) it helps to establish a safe relationship for the exploration of thoughts and feelings (O) because they establish a point of connection, rapport, and trust (M). | (Aventin et al., 2014) (Gutsche, 2013) (Hammond et al., 2021) (Haight et al., 2010) (Watson, Staples, et al., 2020) (Shotton, 2012) (Finlay, 2022) (Hamilton, 2020) (Holody & Mäher, 1996) (Shotton, 2013) (Hooley, 2015) (Hamilton, 2020) (Willis & Holland, 2009) (Buchanan, 2014) | 'The participants claimed that often the children preferred drawing or doing arts and crafts to talking, for example, and this would sometimes prompt them to talk more as they built their confidence in their therapeutic relationship with the social worker.' (Gutsche, 2013)<br><br>'As in conventional LSW with younger children with care-experience, the trusted adult relationship context and engagement with reflective activities created opportunities for a therapeutic alliance between adolescent participants and the first author. In this space, adolescent participants were able to express and begin to reflect on difficult events in their lives' (Hammond et al., 2021) |
| **CMOC11** | When adolescents' everyday life experiences are recorded (C) they can share it with others in the future (O) because prompts from events have been saved (M). | (Shotton, 2012) (Shotton, 2013) (Watson, Hahn, et al., 2020) (Hammond, 2012) (Atwool, 2017) (Hanna, 2007) | 'In looking through the store both carers and children were able to flesh out each memory with details of when it occurred or who else was there. It was clear that the pictorial information was very powerful in taking the participants back to the time and place when it occurred.' (Shotton, 2013)<br><br>'Importantly, children's 'stuff' can be used to help the child and new family to understand their journey' (Watson, Hahn, et al., 2020) |



For peer review only - http://bmjopen.bmj.com/site/about/guidelines.xhtml

Improving the mental health and mental health support available to adolescents looked-after by social care via adolescent-focused low-intensity life story work: A realist review

| | | | |
|---|---|---|---|
| **CMOC12** | Sharing, preserving, and reflecting on everyday life experiences with a caring adult (C) increases an adolescent's sense of connectedness and understanding of themselves and others (O) because it provides opportunities to talk and be listened to (M) | (Shotton, 2010) (Watts, 2021) (Eldridge, 2018) (Shotton, 2012) (Watson, Staples, et al., 2020) (Hamilton, 2020) (Shotton, 2013) (Känkänen & Bardy, 2014) (Malik, 2005) (Hughes, 2013) (Gutsche, 2013) (BASW, 2020) | It often opened up lines of communication, sometimes leading to conversations, where other positive memories were triggered or where the child felt able to talk about sensitive issues.' (Shotton, 2010)<br><br>'Where adopted CYP were able to discuss their life stories with their parents in nonthreatening, everyday ways, feelings of relief and connection with parents were expressed.' (Watson, Staples, et al., 2020) |
| **CMOC13** | When caring adults are prepared to share personal information with the adolescent they are caring for (C) the adolescent is more willing to confide with their caring adult (O) because trust is established (M). | (Eldridge, 2018) (Watson, Staples, et al., 2020) | 'Furthermore, in the current study, limited reciprocal confiding and self-disclosure appeared to help equalise power dynamics and facilitate genuine confiding across personal and professional relationships.' (Eldridge, 2018) |
| **CMOC14** | When adolescents have consistent and repeated positive engagements with caring adults who support them to preserve and reflect on everyday life experiences (C) then they may start to build trust in others (O) because it challenges their previously held negative relational beliefs (M). | (Finlay, 2022) (Eldridge, 2018) (Wood & Selwyn, 2017) (Atwool, 2017) (Hamilton, 2020) (Hooley et al., 2016) (Shotton, 2012) (Furnivall & Grant, 2014) | 'Reciprocity, understanding, acceptance and perseverance all appeared to be important for the emergence of sufficient trust to confide. For some young people, trust developed in one relationship then appeared to generalise into other relationships' (Eldridge, 2018)<br><br>'Such work also enables the child to share their story, has the potential to increase self-esteem and, by building a sense of trust with the social worker and caregivers, may facilitate the development of secure |



For peer review only - http://bmjopen.bmj.com/site/about/guidelines.xhtml

Improving the mental health and mental health support available to adolescents looked-after by social care via adolescent-focused low-intensity life story work: A realist review

| | | | |
|---|---|---|---|
| | | | attachment in new situations.' (Atwool, 2017) |
| **CMOC15** | When a caring adult provides consistent support to an adolescent to discuss their thoughts and feelings in response to everyday life experiences (C) the adolescent comes to understand their experiences and themselves better, including their emotional and behavioural responses to experiences (O) because they develop reflective and reminiscence skills through shared story telling (M). | (Ward, 2002) (Hamilton, 2020) (Hooley et al., 2016) (Hooley, 2015) (Fitzhardinge, 2008) (Steenbakkers et al., 2016) (Watson et al., 2018) (Haight et al., 2010) (Holody & Mäher, 1996) (Watson et al., 2015b) (Malik, 2005) (Shotton, 2013) (Ferrier, 2011) | 'Reflective function can be encouraged by bringing into consciousness a range of possible interpretations or ways of experiencing similar stories. It has been argued that both reflective function and neural integration develop only in a context of something akin to a secure attachment relationship.' (Fitzhardinge, 2008)<br><br>"This life book focuses on the here and now and makes use of the relationship between the worker and the child. While not specifically therapeutic in its goals, it seeks to help the child make connections between his or her present experiences, emotional needs, and past events." (Holody & Mäher, 1996)<br><br>'In this model, the key aims of the LSW are to integrate a child's internal and external experiences by collaboratively constructing a narrative.' (Hooley et al., 2016) |
| **CMOC16** | When a caring adult provides consistent support to an adolescent to discuss their thoughts and feelings in response to everyday life experiences (C) they experience better mental | (NSPCC, 2022) (Hooley et al., 2016) (Willis & Holland, 2009) (Känkänen & Bardy, 2014) (Steenbakkers et al., 2016) (Hills, 2022) (Furnivall & Grant, 2014) (Aventin et al., 2014) (Holody & Mäher, 1996) (Eldridge, 2018) | 'A universal viewpoint of exploring and managing emotions emerged, clearly suggesting the importance of helping children to identify, express and regulate emotions during their involvement' (Hooley et al., 2016)<br><br>'respondents indicated that good life story |



For peer review only - http://bmjopen.bmj.com/site/about/guidelines.xhtml

Improving the mental health and mental health support available to adolescents looked-after by social care via adolescent-focused low-intensity life story work: A realist review

| | | | |
|---|---|---|---|
| | health (O) because they learn emotion regulation skills (M). | | work should involve helping a child to express and manage emotions that arise during the work.' (Hooley et al., 2016) |
| **CMOC17** | When caring adults consistently support an adolescent to preserve and reflect on everyday life experiences (C) it strengthens the relationship between them (O) because the caring adult becomes better attuned to understanding the adolescent and their support needs (M). | (Davies & Hodges, 2017) (Malik, 2005) (Shotton, 2012) (Holody & Mäher, 1996) (Atwool, 2017) (Hooley, 2015) (Hanna, 2007) | 'But by this stage, the carer was more attuned to Katie's particular experiences and was able to demonstrate the necessary acceptance, understanding, commitment and unconditional love for Katie that soothed and ameliorated her anxieties'. (Davies & Hodges, 2017)<br><br>'it gives the worker information critical to understanding and linking the child's present functioning and coping patterns with previous events in the child's life.' (Holody & Mäher, 1996)<br><br>'The here-and-now approach allowed Helen's mother to accept her child's thoughts and feelings as the girl's own perception and understanding of her life.' (Holody & Mäher, 1996) |
| **CMOC18** | When caring adults view everyday interactions with an adolescent as providing reflective opportunities (C) their relationship is strengthened (O) because adolescents are more comfortable | (Ward, 2002) (Finlay, 2022) (Hammond, 2016) (Connor et al., 1985) (Hamilton, 2020) (Aventin et al., 2014) (Känkänen & Bardy, 2014) | 'The keyworker also had to find time for spontaneous 'out of hours' sessions, such as helping Ben write short stories about his temper tantrums after they happened.' (Connor et al., 1985)<br><br>'You can use your very being to embrace the |



For peer review only

| | | | |
|---|---|---|---|
| | talking and reflecting on everyday events (M). | | relationships and create key opportunities for exploring each life story. 'Don't wait for the perfect moment, take the moment and make it perfect' (Anonymous).' (Hamilton, 2020) |
| **CMOC19** | When low-intensity LSW takes place in emotionally meaningful places (C) richer narratives can be constructed (O) because of the availability of contextual cues (M). | (Hills, 2022) (Haight et al., 2010) (Hammond, 2012) (Hamilton, 2020) (Watson, Staples, et al., 2020) | 'Providing an intervention to children within a context familiar and emotionally meaningful to them has a variety of other benefits as well. When the clinician travels to see them individually and on their own turf, children can feel valued and cared for, and relationship building may be enhanced. In addition, the community context is rich in cues for children to spontaneously initiate personal narratives.' (Haight et al., 2010) |
| **CMOC20** | When everyday life experiences are preserved using temporal anchor points and reflected on at regular intervals (C) it aids the ease of storytelling (O) because it is easier to follow (M). | (Hammond et al., 2021) (Hammond, 2012) (Gustavsson & MacEachron, 2008) (NICE, 2021) | 'My regular weekly visits also provided young people with a frame of reference from which to begin to construct and reflect upon recent events in a temporal fashion.' (Hammond, 2012)<br><br>'Schedule regular, dedicated times for life story work to help the looked-after child or young person make sense of their journey through the care system and beyond, their significant relationships and their identity.' (NICE, 2021) |



For peer review only - http://bmjopen.bmj.com/site/about/guidelines.xhtml

Improving the mental health and mental health support available to adolescents looked-after by social care via adolescent-focused low-intensity life story work: A realist review

| | | | |
|---|---|---|---|
| **CMOC21** | When everyday life experiences are preserved and reflected on at regular intervals (C) adolescents become more comfortable engaging in the process (O) because the approach becomes familiar and predictable (M). | (Holody & Mäher, 1996) (Hamilton, 2020) (Shotton, 2012) | 'The here-and-now lifebook is more user-friendly than the traditional lifebook, not only for the child but for the worker as well. If it is used in every contact between worker and child, it is transformed from something extra into the very forum for interaction.'(Holody & Mäher, 1996) |
| **CMOC22** | When a caring adult regularly demonstrates how to respond to challenging situations in constructive ways (C) the adolescent learns how to respond to challenging situations (O) because of role modelling (M). | (Furnivall & Grant, 2014) (Hammond & Cooper, 2013) (Hammond, 2016) (Finlay, 2022) (Steenbakkers et al., 2016) | Developing the capacity to self-regulate and become accountable requires safe, positive relationships with adults who can scaffold children's' learning (Furnivall & Grant, 2014)<br><br>'This person also needs to be a role model, particularly when handling the inherent frustrations of working with digital media in innovative ways and helping to mediate the risks that digital media may bring (discussed later in this chapter).' (Hammond, 2016)<br><br>'In addition, negative experiences with (previous) attachment figures make sharing distressing memories more difficult for adolescents in family foster care, for example when these attachment figures insufficiently regulated the emotional aspects of distressing events for them, or insufficiently scaffolded them in constructing these memories' (Steenbakkers et al., 2016) |



For peer review only - http://bmjopen.bmj.com/site/about/guidelines.xhtml

Improving the mental health and mental health support available to adolescents looked-after by social care via adolescent-focused low-intensity life story work: A realist review

| | | | |
|---|---|---|---|
| **CMOC23** | When a caring adult consistently and sensitively helps an adolescent curate, develop, and reflect upon narratives about their lived experiences (C) the narratives become authentic accounts that resonate with the adolescent's experience (O) because they were actively involved in the construction of accounts (M) | (Atwool, 2017) (Baynes, 2008) (Watson, Hahn, et al., 2020) (Watson, Staples, et al., 2020) | 'This will help to ensure that the written story reflects the child's experiences, helping to create a coherent narrative of events, feelings and memories. Without this, we risk creating a life story book that has little meaning to the child,' (Baynes, 2008) |
| **CMOC24** | When adolescents are not actively involved in the construction of narratives about their lived experience (C) they can struggle to accept the narratives as their own story (O) because they do not resonate with the young persons lived experience (M). | (Baynes, 2008) (Watson, Hahn, et al., 2020) (Gallagher & Green, 2012) (Atwool, 2017) | 'Children may feel that their life story book lacks a coherent narrative or presents a narrative with which they disagree (Watson et al., 2015a). This can lead to dissonance between their identity and the identity presented in the book, leading children to believe that including multiple perspectives would be beneficial (Watson et al., 2015a).' (Watson, Hahn, et al., 2020)<br><br>'All proponents of life story work emphasise the importance of working with the child and allowing them to give voice to their subjective experience. Kagan (2014) warns of the dangers of practitioners writing life stories for children, arguing that their validity is likely to be questioned because they do not fully capture the child's experience or perspective and traumatic events may be glossed over.' |



For peer review only - http://bmjopen.bmj.com/site/about/guidelines.xhtml

Improving the mental health and mental health support available to adolescents looked-after by social care via adolescent-focused low-intensity life story work: A realist review

| | | | |
|---|---|---|---|
| | | | (Atwool, 2017) |
| **CMOC25** | When caring adults do not consistently and sensitively help adolescents curate, develop, and reflect upon narratives about their lived experiences (C) identity development can be negatively impacted (O) because the adolescent constructs unnecessarily negative narrative accounts (M). | (Watson, Hahn, et al., 2020) (Shotton, 2012) (Atwool, 2017) (Watson, Staples, et al., 2020) | 'Granting children control over narratives aids identity formation but left unchallenged their perspective of events may also become one-sided and inaccurate' (Watson, Hahn, et al., 2020) |
| **CMOC26** | When a caring adult supports an adolescent to construct narrative accounts of their experience using alternative information or interpretations (C), less negative narratives may result (O), because they have more perspectives to draw on (M) | (Fitzhardinge, 2008) (Shotton, 2012) (Davies & Hodges, 2017) (Pakrosnis & Cˇepukiene, 2011) (Hooley et al., 2016) (Baynes, 2008) (Shotton, 2013) (Gallagher and Green, 2012) (Shotton, 2010) (Willis and Holland, 2009) | 'Narratives were enriched by bringing into consciousness some alternative interpretations and accommodating in a deeper way the perspectives of different people within the story.' (Fitzhardinge, 2008)<br><br>'From a narrative perspective it can help the child to thicken positive counter-narratives impacting on the dominant stories the child has and is able to tell about themselves, particularly thickening stories around their strengths, worth and belonging' (Shotton, 2012)<br><br>Participants also highlighted the importance of finding out what the events mean to the child and offering alternative narratives:' (Hooley et al., 2016) |
| **CMOC27** | When a caring adult consistently and sensitively helps an | (Connor et al., 1985) (Hammond & Cooper, 2013) (Finlay, 2022) | The therapeutic process involves creating opportunities to open up conversational |



For peer review only - http://bmjopen.bmj.com/site/about/guidelines.xhtml

Improving the mental health and mental health support available to adolescents looked-after by social care via adolescent-focused low-intensity life story work: A realist review

| | | | |
|---|---|---|---|
| | adolescent curate, develop, and reflect upon narratives about their lived experiences (C) positive narratives including liberating narratives can be constructed (O) because caring adults can help adolescents to re-script negative narrative accounts (M). | (Hammond, 2016) (Hammond, 2012) (Fitzhardinge, 2008) (Gutsche, 2013) (Happer et al., 2006) (Hooley, 2015) (Känkänen & Bardy, 2014) (Cook-Cottone & Beck, 2007) (Watson et al., 2015b) (Holody & Mäher, 1996) (Shotton, 2013) (Hooley et al., 2016) | spaces. These spaces provide young people with opportunities to share accounts of their own lives, in their own words. These accounts are listened to by a consistently available adult who can sensitively support the young person's reflections on the stories they choose to share, and potentially, provide re-scripting or re-editing advice.' (Hammond, 2016).<br><br>'Importantly, these reflections must eradicate self-blame and encourage young people to look forward positively towards their futures. This phase is about encouraging young people to recognise their own resilience, effectively turning 'if only this hadn't happened' into 'despite all these things that have happened to me'. (Hammond & Cooper, 2013) |
| **CMOC28** | When significant individuals provide alternative positively framed narratives to the adolescent (C) it helps to increase their self-esteem and ability to develop positive future expectations (O) because they become increasingly aware of their positive characteristics and abilities (M). | (Shotton, 2012) (Aust, 1981) (Hooley et al., 2016) (Teodorczuk et al., 2018) (Eldridge, 2018) (Happer et al., 2006) (Sanders, 2020) (Devenney, 2017) | 'From a narrative perspective it can help the child to thicken positive counter-narratives impacting on the dominant stories the child has and is able to tell about themselves, particularly thickening stories around their strengths, worth and belonging.' (Shotton, 2012)<br><br>'The co-construction of stories around their achievements and certificates contributes to |



For peer review only - http://bmjopen.bmj.com/site/about/guidelines.xhtml

Improving the mental health and mental health support available to adolescents looked-after by social care via adolescent-focused low-intensity life story work: A realist review

| | | | |
|---|---|---|---|
| | | | their perception of themselves as someone who can achieve. As they reflect with their carer and look together at their writing and spelling they are able to co-construct stories about their progress over time and see themselves as someone who can make progress, who can grow and change in positive ways.' (Shotton, 2012) |
| **CMOC29** | When significant individuals are given the opportunity to offer their narrative account of adolescent's life experiences (C) adolescents become aware of alternative interpretations of life events (O) because they have access to different narrative accounts (M). | (Bazalgette, 2015) (HCC, 2022) (Hills, 2022) (Beste & Richardson, 1981) (Baynes, 2008) (Peake, 2009) (Ferrier, 2011) (BASW, 2020) (ARCBOX, 2022) (Fitzhardinge, 2008) (Watson, Hahn, et al., 2020) (Hoyle et al., 2020) (Watson et al., 2015a) (Watts, 2021) | 'Fourth, Life Story Books that include contributions from more than one source, such as foster parents, social worker, and parents, will allow children to have a broader understanding of their life events. It will also increase the child's awareness that different people see the same situation differently.' (Beste & Richardson, 1981)<br><br>'Children were consistently clear that the narrative presented in their book should be of their biography, representing multiple actor viewpoints including those of their birth family.' (Watson et al., 2015a)<br><br>'In addition, a life story book that contains many voices is likely to be seen by the child as having more credibility in the future'' (Baynes, 2008) |
| **CMOC30** | When significant individuals including birth relatives are | (Cook-Cottone & Beck, 2007) (Shotton, 2012) (Buchanan, 2014) | ''The life-story work can also be used as a way to structure visits with biological parents |



For peer review only - http://bmjopen.bmj.com/site/about/guidelines.xhtml

Improving the mental health and mental health support available to adolescents looked-after by social care via adolescent-focused low-intensity life story work: A realist review

| | | | |
|---|---|---|---|
| | positively involved in Low intensity LSW (C) stronger relationships can be established between those involved (O) because of the interactions facilitated through the activity (M). | (Watson, Staples, et al., 2020) (Hooley, 2015) (Shotton, 2012) | and siblings. The visit time can be used to collect artifacts (take photos or share old photos) and record related information (e.g. write letters, create a memory page for photos, tell stories about the photos).' (Cook-Cottone & Beck, 2007)<br><br>'facilitating interaction with other family members, bringing the family closer together, helping other family members feel involved in the care of the child' (Shotton, 2012) |
| **CMOC31** | When significant individuals including birth relatives are involved in low intensity LSW (C) identity development can be supported (O) because significant individuals can share information about the adolescent's family heritage (M). | (Monson et al., 2020) (Neil & Beek, 2020) (Buchanan, 2014) (Hoyle et al., 2020) (Sanders, 2020) | 'For young people, carers and workers could also be gatekeepers for connectedness with cultural heritage and identity. One young person described what he believed was a turning point in a relationship with a carer, when the carer took him to a restaurant serving the cuisine of the young person's country of origin' (Monson et al., 2020)<br><br>'Staying in touch with parents, siblings and other important people helps children develop a sense of identity and belonging and promotes healthy and stable relationships,'(Sanders, 2020) |
| **CMOC32** | When adolescents exert a choice over how low intensity LSW everyday life experiences are preserved and reflected on (C) they experience ownership over the process and resulting | (Hooley, 2015) (Watson et al., 2018) (Buchanan, 2014) (Hammond & Cooper, 2013) (Watson, Hahn, et al., 2020) (Holody & Mäher, 1996) (NICE, 2021) (HCC, 2022) (Hammond, 2012) (Peake, 2009) | 'Children can have ownership of their story work via choosing which objects to story/not story and by dictating the pace at which the work progresses:<br>I think it's a great concept and would be a good idea for children to have ownership of |

For peer review only

Improving the mental health and mental health support available to adolescents looked-after by social care via adolescent-focused low-intensity life story work: A realist review

| | | | |
|---|---|---|---|
| | products (O) because they feel valued their perspectives and opinions are valued. | (Gutsche, 2013) (Buchanan, 2014) (Lucas et al., 2020) (Baynes, 2008) | their stories and memories. (FSW, Ben)' (Watson, Hahn, et al., 2020)<br><br>'A feeling of ownership appeared to be fostered through the use of a person-centred and flexible approach to LSW.' (Buchanan, 2014) |
| **CMOC33** | When adolescents are given agency to choose the mediums (digital, paints, puppets, physical/digital 'visits' music etc) used to collect and conserve everyday memories (C) it is engaging to them (O) because they have a sense of control, ownership and power over the process, (M). | (Watson, Hahn, et al., 2020) (Hammond, 2012) (Hammond, 2016) (Watson et al., 2015a) (Aventin et al., 2014) (Hills, 2022) (Buchanan, 2014) (Holody & Mäher, 1996) (Hammond & Cooper, 2013) (Nuffield Family Justice Observatory, 2021) (Baynes, 2008) (Hooley et al., 2016) (Hammond et al., 2021) (Lucas et al., 2020) (Peake, 2009) (Willis & Holland, 2009) (Känkänen & Bardy, 2014) (Gutsche, 2013) (Hammond & Cooper, 2013) | 'trove aims to support children to keep their own record of their life, through interactive child-driven technology, to give them some control of their life story.' (Watson, Hahn, et al., 2020)<br><br>'…how we can support that so it feels like their story rather than produced in a format that somebody has given them. We want to make sure that for young people, their stories are owned by them, and that means having them in a format that they would choose.' (Hills, 2022) |
| **CMOC34** | When caring adults are aware of the adolescent's special educational needs and disabilities (SEND) (C) the adolescent can engage with preserving and reflecting on their experiences (O) because appropriate adaptations | (Hamilton, 2020) (Hammond, 2012) (HCC, 2022) (Atwell, 2016) (NICE, 2021) | 'Where the child has some level of learning disability, careful thought will need to be given to the implications this will have on undertaking life story work, considering in particular the most effective methods of communication.' (HCC, 2022) |



For peer review only - http://bmjopen.bmj.com/site/about/guidelines.xhtml

Improving the mental health and mental health support available to adolescents looked-after by social care via adolescent-focused low-intensity life story work: A realist review

| | | | |
|---|---|---|---|
| | can be made to make the activity accessible (M). | | 'For children with a disability who perhaps have difficulty in communicating by speech, or who may lack the use of one or more of the senses, it is possible to develop life story work that does not depend only on visual methods but involves stimulation of other senses.' (Atwell, 2016)<br><br>'Take a flexible approach to life story work, and tailor it to the developmental age and needs of the looked-after child or young person.' (NICE, 2021) |
| **CMOC35** | When caring adults from the adolescent's support network have training materials available to them (C) adolescents with SEND can engage with preserving and reflecting on their lived experiences (O) because the caring adults in their support network are equipped with the knowledge and skill to make appropriate adaptations (M). | (Atwell, 2016) | 'In deciding how to tackle life story work with disabled children, it is also important to consider who would be best able to do this work. Since being able to understand and communicate with the child is paramount, this should be undertaken by the person who communicates best with the child, rather than assume it is always done by the child's social worker.' (Atwell, 2016) |
| **CMOC36** | When the creation of a product is prioritised over a meaningful process for securing everyday memories (C) the value of narratives contained will be | (Känkänen & Bardy, 2014) (Hammond & Cooper, 2013) (Hooley et al., 2016) (Baynes, 2008) (Hammond, 2012) | 'Baynes (2008) continues by suggesting that life story work has been pushed to the periphery of practice within a more quantitatively driven target-focused profession. She fears that this may lead some |



For peer review only - http://bmjopen.bmj.com/site/about/guidelines.xhtml

Improving the mental health and mental health support available to adolescents looked-after by social care via adolescent-focused low-intensity life story work: A realist review

| | | | |
|---|---|---|---|
| | reduced (O) because the product does not represent adolescents lived experience of everyday events (M). | | to conceptualise life story work as a product, which could detract from the potentially therapeutic nature of the underlying process.' (Hammond, 2012)<br><br>'They also note that although the process is of primary importance, the material record was also significant.' (Atwool, 2017) |
| **CMOC37** | When the process of curating, developing, and reflecting upon narratives about lived experiences is prioritised over the recording (C) a bank of future reflective starting points is lost (O) because everyday memories and artefacts are not secured (M). | (Aust, 1981) (Walker & Ryan, 2016a) (Hoyle et al., 2020) (Hooley, 2015) (Watson et al., 2015b) (Hills, 2022) (Watts, 2021) (Buchanan, 2014) (Känkänen & Bardy, 2014) (Atwool, 2017) (Willis & Holland, 2009) (Beste & Richardson, 1981) (Aust, 1981) (Hammond, 2016) | 'We never regard the work as finished as life goes on, but some record of the process is important as it provides a reference point, particularly as it can be updated until adulthood.' (Walker & Ryan, 2016a)<br><br>'Material items and the records produced during the work were important to the young people. Many described treasuring them, returning to them often and planning to continue to add to them.' (Buchanan, 2014)<br><br>'Concrete artefacts – such as poems, photographs, paintings, cartoons and so on – cannot be ignored, which also means that unlike an unrecorded spoken narrative, they persist after the initial act of telling and we can return to them again and again.' (Känkänen & Bardy, 2014) |
| **CMOC38** | When adolescents feel empowered to have editorial | (Baynes, 2008) (Watson, Hahn, et al., 2020) (Watson et al., 2018) | 'Children can have ownership of their story work via choosing which objects to story/not |



For peer review only - http://bmjopen.bmj.com/site/about/guidelines.xhtml

Improving the mental health and mental health support available to adolescents looked-after by social care via adolescent-focused low-intensity life story work: A realist review

| | | | |
|---|---|---|---|
| | control over memory product(s) (C) they feel more positive about the product(s) and experience greater ownership of their story/narrative (O) because they have control over how, and which, events are recorded or not (M). | (Hoyle et al., 2020; Watson et al., 2015a) (Buchanan, 2014) (Gallagher & Green, 2012) (Gray et al., 2019) | story and by dictating the pace at which the work progresses' (Watson, Hahn, et al., 2020)<br><br>'In some cases children were dismissive about their book, as the focus did not seem to be on them specifically. For a few children, photos included were stark reminders of differential treatment of siblings by birth parents and contributed to negative feelings.' (Watson et al., 2015a)<br><br>'have stuck a piece of paper over a section about me behaving inappropriately around Rhian and John, my adoptive family that broke down. The thing in the life story book spoilt my memory of Rhian and John's …. It seemed like it was written for somebody younger than me. (Caitlyn, 19)' (Gallagher & Green, 2012) |
| **CMOC39** | When the medium used for collecting the product is easily editable (e.g., digitally) (C) it helps the contents of low intensity LSW hold meaning over time (O) because it can be updated and edited when needed (M) | (Beste & Richardson, 1981) (Hammond, 2012) (Hammond & Cooper, 2013) (Watson et al., 2015a) (Watson, Latter and Below, 2015) (Atwool, 2017) (Hammond & Cooper, 2013) (Hooley et al., 2016) (Nuffield Family Justice Observatory, 2021) | 'Digital tools offer the ability to help young people to express thoughts and feelings which can be continually updated, edited and reflected upon. Using interactive computer-based mediums in this way grants the user flexibility to make changes frequently and easily.' (Hammond & Cooper, 2013)<br><br>'Concerns about the scrapbook style focused on the inability to remove information or to update as the child grew older. ' (Watson, |



For peer review only - http://bmjopen.bmj.com/site/about/guidelines.xhtml

Improving the mental health and mental health support available to adolescents looked-after by social care via adolescent-focused low-intensity life story work: A realist review

| | | | |
|---|---|---|---|
| | | | Latter and Below, 2015) |
| **CMOC40** | When digital artefacts/products are backed-up and have access controls (e.g., encrypted and password protected) (C) it helps preserve and keep the contents safe (O) because it is secured and protected from unauthorised access (M). | (Hammond & Cooper, 2013) (Shepard, 2022) (Ferrier, 2011) (Watson et al., 2015a) (Atwool, 2017) (Willis & Holland, 2009) (Gustavsson & MacEachron, 2008) (Beste & Richardson, 1981) (Hammond & Cooper, 2013) (Hammond, 2016) (Ferrier, 2011) (Gray et al., 2019) | 'Security mechanisms were posited from both groups, including remotely programmable passcodes or a scannable key.' (Gray et al., 2019)<br><br>'Provision of back-up storage in case records are lost or destroyed is also needed.' (Atwool, 2017)<br><br>'Digital life story work products can be copied easily and, with permission, back-up copies can be stored with young people's files for posterity.' (Hammond, 2016) |
| **CMOC41** | When a medium for capturing a product enables narratives to be stored in different formats (e.g. voices, videos, pictures, objects) (C), they provide a richer starting point for future retelling (O), because they connect adolescents to their memories through the engaging and tangible nature of the artefacts (M). | (Hammond & Cooper, 2013) (Peake, 2009) (Hills, 2022) (Watson et al., 2015a) (Gray et al., 2019) (Watson et al., 2018) (Shotton, 2012) (Shotton, 2013) (Hammond, 2016) (Buchanan, 2014) (Atwell, 2016) (Hoyle et al., 2020) (Watson, Hahn, et al., 2020) | 'The background sounds to everyday life often go unnoticed. Sounds from the environment can help to connect to memories and hearing a specific sound can prompt a memory.' (Hammond & Cooper, 2013)<br><br>'For looked after and adopted children, physical objects are often the only remaining link to their past; a portal to stories of birth families, former homes, and significant people.' (Gray et al., 2019)<br><br>'In looking through the store both carers and children were able to flesh out each memory with details of when it occurred or who else |



For peer review only - http://bmjopen.bmj.com/site/about/guidelines.xhtml

Improving the mental health and mental health support available to adolescents looked-after by social care via adolescent-focused low-intensity life story work: A realist review

| | | | |
|---|---|---|---|
| | | | was there. It was clear that the pictorial information was very powerful in taking the participants back to the time and place when it occurred.' (Shotton, 2013) |
| **CMOC42** | When adolescent's communication needs are considered when creating low-intensity LSW products (C) it ensures that memories are accessible for the adolescent to engage with (O) because they can understand the contents (M). | (Watson, Latter & Bellew, 2015) (Hammond, 2012) (Atwell, 2016) | "A lack of appropriate methods of communication in life story work may result in engagement opportunities with adolescents becoming lost and/or overlooked." (Hammond, 2012)<br><br>"…the care system and social work practice itself needs to converse using culturally appropriate methods of communication… ' (Hammond, 2012)<br><br>'For children with a disability who perhaps have difficulty in communicating by speech, or who may lack the use of one or more of the senses, it is possible to develop life story work that does not depend only on visual methods but involves stimulation of other senses.' (Atwell, 2016) |
| **CMOC43** | When adolescents use LI-LSW products as storytelling prompts to share stories with chosen caring adults (C) it helps to strengthen relationships between adolescents | (Buchanan, 2014) (Shotton, 2012) (Hammond, 2012) (Hamilton, 2020) | 'Sharing a moment, an experience together helps to create social connections and helps us understand one another and our thinking.' (Hamilton, 2020) |



For peer review only - http://bmjopen.bmj.com/site/about/guidelines.xhtml

Improving the mental health and mental health support available to adolescents looked-after by social care via adolescent-focused low-intensity life story work: A realist review

| | | | |
|---|---|---|---|
| | and caring adults (O) (O) because caring adults understand adolescents and their experiences | | |
| **CMOC44** | When current everyday life experiences are preserved and reflected on (C) positive self-narratives can become more accessible for the adolescent to make sense of their experiences, construct their identity and develop positive future expectations (O) because the focus is not on past negative experiences. | (Shotton, 2013) (Shotton, 2012) (Nicholls, 2003) (Teodorczuk et al., 2018) (Ferrier, 2011) (Haight et al., 2010) (Watson, Staples, et al., 2020) (Steenbakkers et al., 2016) (NSPCC, 2022) (Hammond et al., 2021) (Teodorczuk et al., 2018) | 'Why Life Story Work is Failing…It has the potential to deny the promotion of a child's identity by focusing on his or her experience as a looked-after child.' (Nicholls, 2003)<br><br>'From a narrative perspective it can help the child to thicken positive counter-narratives impacting positively on the dominant stories the child has and is able to tell about themselves, particularly thickening stories around their strengths, worth and belonging.' (Shotton, 2012)<br><br>'A key part of Life Story Work is celebrating the child or young person's achievements. We recognise the resilience they've shown by overcoming adversity and help them to see themselves in a positive light.' (NSPCC, 2022) |
| **CMOC45** | When everyday life experiences are reflected on between an adolescent and caring adult (C) difficulties and challenges can be positively re-framed (O) because | (Walker & Ryan, 2016a) (Holody & Mäher, 1996) (Buchanan, 2014) (Teodorczuk et al., 2018) (Hooley et al., 2016) | 'Life story books are thought to sometimes silence the difficulties experienced by adoptees by not allowing them enough space for exploring troublesome feelings and fantasies.' (Walker & Ryan, 2016a) |



For peer review only - http://bmjopen.bmj.com/site/about/guidelines.xhtml

Improving the mental health and mental health support available to adolescents looked-after by social care via adolescent-focused low-intensity life story work: A realist review

| | | | |
|---|---|---|---|
| | opportunities to reflect on positive interpretations are taken (M). | | 'The statements relating to this all emphasised 'feelings to be shown, managed and normalised'. Participants in all groups agreed with the suggestion that work should not be stopped if difficult feelings came up and that upsetting or traumatic experiences should be explored. They indicated that a balance needed to be achieved that included happy as well as difficult memories.' (Hooley et al., 2016) |
| **CMOC46** | When low-intensity LSW containing a bank of positive memories and personal achievements is re-visited by an adolescent needing emotional support (C) their wellbeing may improve (O), because they find the contents comforting (M) | (Watts, 2021) (Shotton, 2010) (Buchanan, 2014) (Cook-Cottone & Beck, 2007; Gallagher & Green, 2012) (Shotton, 2012) (Shotton, 2013) (Humphreys & Kertesz, 2014) (Willis & Holland, 2009) (Watts, 2021) (Holody & Mäher, 1996) | 'The value of returning to the book was described by the other participants. Some chose to simply look through it to see 'familiar faces' or to remind them of coming through difficult times in the past when faced with new transitions. (Buchanan, 2014)<br><br>'As many of the memories were of happy/positive times, the carers felt that reflecting on the store often had a positive effect on mood for both themselves and the children.' (Shotton, 2012)<br><br>'The carers perceived that using the memory store approach often had a calming effect on the children and that it was also an activity that they seemed to find comforting. (Shotton, 2012) |



For peer review only - http://bmjopen.bmj.com/site/about/guidelines.xhtml

Improving the mental health and mental health support available to adolescents looked-after by social care via adolescent-focused low-intensity life story work: A realist review

| | | | |
|---|---|---|---|
| **CMOC47** | When caring adults have an awareness of the adolescent's cultural background (C) it promotes appropriate preservation and reflection of everyday life experiences (O) because caring adults have an increased awareness of the adolescent's individuality and cultural heritage (M). | (Monson et al., 2020) (HCC, 2022) (Walker & Ryan, 2016b) (NICE, 2021) | 'It is important to a child that the worker doing life story work has a good grasp of the child's world, both the inner world and external realities. This means the worker must familiarise him or herself with aspects of family life of the ethnic community of the child in the context of this society. This involves getting information from a variety of sources, for example, people from a culture similar to that of the child, and of course the internet.' (Walker & Ryan, 2016b)<br><br>'Ensure that life story work for looked-after children and young people captures and embraces ethnicity, cultural and religious identity, as well as other personal aspects of identity, for example, sexual identity or disabilities.' (NICE, 2021) |
| **CMOC48** | When institutions consistently communicate and take measures to support the importance of collecting everyday life experiences for adolescents (C) significant individuals are more likely to prioritise this activity (O) because they know what is expected of them (M). | (Finlay, 2022) (Wood, 2019) (Atwool, 2017) (Holody & Mäher, 1996) (Shotton, 2013) (Watts, 2021) (Hooley et al., 2016) (Connor et al., 1985) (Brookfield et al., 2008) | 'Recognition of children's entitlement to a coherent narrative needs to be embedded in practice at the micro level. This can only occur if appropriate supports are in place at the organisational level, facilitated by macro-level priority being accorded to provision of resources needed to ensure the best possible outcomes for children and young people in care.' (Atwool, 2017)<br><br>''Policy needs to be developed which specifies that training for foster carers in |



For peer review only - http://bmjopen.bmj.com/site/about/guidelines.xhtml

Improving the mental health and mental health support available to adolescents looked-after by social care via adolescent-focused low-intensity life story work: A realist review

| | | | |
|---|---|---|---|
| | | | using the approach, or similar, should be part of their induction to fostering. This would raise their awareness and help them feel equipped to use the approach from the start of a child's placement with them.' (Shotton, 2013) |
| **CMOC49** | When caring adults are provided with relevant ongoing training that highlights the importance of preserving and reflecting on everyday life experiences (C), they are more likely to carry these out (O) because they have the confidence and skills to do so (M). | (Finlay, 2022) (Braiden, 2016) (Walker & Ryan, 2016a) (Eenshuistra et al., 2019) (Hills, 2022) (Haight et al., 2010) (Wood, 2019) (Watson, Staples, et al., 2020) (NICE, 2021) (Shotton, 2012) (Buchanan, 2014) (Holody & Mäher, 1996) (Shotton, 2013) (Atwool, 2017) (Sanders, 2020) | 'To embark on this journey with the child, above all you need time, sensitivity, empathy for the child and a commitment to the work. An ability to listen to the child and understand them is paramount; the skills and particular techniques can be learnt' (Walker & Ryan, 2016a)<br><br>'Ensure that the experience and skillset of the practitioner or carer delivering life story work for looked-after children and young people is sufficient to deliver good quality work, particularly in complex situations.' (NICE, 2021) |
| **CMOC50** | When peers with similar lived experiences introduce low-intensity LSW to an adolescent (C) their engagement with low-intensity LSW increases (O) because they find advice from peers more credible and authentic (M). | (Holody & Mäher, 1996) (Hughes, 2013; Lucas et al., 2020) (Fitzhardinge, 2008) | 'James was able to show his lifebook to another foster child who was resistant to beginning this work.' (Holody & Mäher, 1996)<br><br>'The use of published story books which describe similar scenarios of children who are in similar circumstances - such as where the child in the story is also blaming themselves about their parents' separation - can be very helpful.' (Hughes, 2013) |



For peer review only - http://bmjopen.bmj.com/site/about/guidelines.xhtml

Improving the mental health and mental health support available to adolescents looked-after by social care via adolescent-focused low-intensity life story work: A realist review

| | | | |
|---|---|---|---|
| **CMOC51** | Caring adults who are supporting low-intensity LSW should have access to regular supervision (C) this helps the caring adult feel prepared and confident to support low-intensity LSW and improves their practice (O) because they have support and guidance available to reinforce training and a self-reflective space to process emotions (M). | (Walker & Ryan, 2016a) (Braiden, 2016) (Hooley et al., 2016) (Eenshuistra et al., 2019) (Haight et al., 2010) (Furnivall & Grant, 2014) (Buchanan, 2014) (Connor et al., 1985) (Watson, Hahn, et al., 2020) (Holody & Mäher, 1996) (Peake, 2009) | 'Unless training is reinforced by regular supervision and consultancy and embedded within a trauma sensitive organisational system, it will be unable to change children's experience significantly.' (Furnivall & Grant, 2014)<br><br>'Clear areas for support and consultation that could be provided by clinical psychologists and social care professionals have been highlighted together with areas for future research.' (Hooley et al., 2016)<br><br>'Any worker undertaking life story work requires not only a reasonable knowledge of child development but also access to other workers in this field who can be called upon to give advice and support in the way that we used the clinical psychologist.' (Connor et al., 1985) |

For peer review only

Improving the mental health and mental health support available to adolescents looked-after by social care via adolescent-focused low-intensity life story work: A realist review

References

ARCBOX. (2022). *Digital Life Story ARCBOX* ARC Adoption North East. https://www.arcadoptionne.org.uk/life-story

Atwell, A. (2016). Working with children with a disability. In R. Walker & T. Ryan (Eds.), *Life Story Work why, what, how and when* (pp. 97-102). CoramBAAF Adoption and Fostering Academy.

Atwool, N. (2017). Life story work: Optional extra or fundamental entitlement? *Child Care in Practice*, *23*(1), 64-76. https://www.tandfonline.com/doi/full/10.1080/13575279.2015.1126228

Aust, P. H. (1981). Using the Life Story Book in Treatment of Children in Placement. *Child Welfare*, *60*(8), 535-536,553-560. https://www.jstor.org/stable/45393827

Aventin, A., Houston, S., & Macdonald, G. (2014). Utilising a computer game as a therapeutic intervention for youth in residential care: Some preliminary findings on use and acceptability. *Children and Youth Services Review*, *47*, 362-369. http://ovidsp.ovid.com/ovidweb.cgi?T=JS&PAGE=reference&D=psyc11&NEWS=N&AN=2014-55927-021

BASW. (2020). *Recording in childrens social work*. https://www.basw.co.uk/system/files/resources/basw_recording_in_childrens_social_work_aug_2020.pdf

Baynes, P. (2008). Untold stories: A discussion of life story work. *Adoption & Fostering*, *32*(2), 43-49. https://doi.org/10.1177%2F030857590803200206

Bazalgette, T. R., G. Trevelyan. (2015). Achieving emotional wellbeing for looked after children a whole system approach. .

Beste, H. M., & Richardson, R. G. (1981). Developing a Life Story Book Program for Foster Children. *Child Welfare*, *60*(8), 529-534. https://www.jstor.org/stable/45393826

Bolton, C. (2022). *Life Story Work 5 minute facts* https://www.bolton.gov.uk/downloads/file/2299/life-story-work

Braiden, S. (2016). Evaluation of a short training programme for foster carers. *Scottish Journal of Residential Child Care*, *15*(2). https://www.celcis.org/application/files/2016/2308/5288/004._2016_Vol_15_2_Braiden_Evaluation_for_foster_carers.pdf

Brookfield, H., Brown, S. D., & Reavey, P. (2008). Vicarious and post-memory practices in adopting families: The re-production of the past through photography and narrative. *Journal of Community & Applied Social Psychology*, *18*(5), 474-491. https://onlinelibrary.wiley.com/doi/abs/10.1002/casp.960

Buchanan, A. (2014). *The experience of life story work: reflections of young people leaving care* Cardiff University]. EThOS. https://orca.cardiff.ac.uk/64531/

Connor, T., Sclare, I., Dunbar, D., & Elliffe, J. (1985). Making a life story book. *Adoption & Fostering*, *9*(2), 32-46. https://dx.doi.org/10.1177/030857598500900210

Cook-Cottone, & Beck. (2007). A Model for Life-Story Work: Facilitating the Construction of Personal Narrative for Foster Children. *Child Adolesc Ment Health*, *12*(4), 193-195. https://doi.org/10.1111/j.1475-3588.2007.00446.x



For peer review only - http://bmjopen.bmj.com/site/about/guidelines.xhtml

Improving the mental health and mental health support available to adolescents looked-after by social care via adolescent-focused low-intensity life story work: A realist review

Davies, M., & Hodges, J. (2017). Relationship renaissance: the use of attachment-based narrative and metaphor in life story work. *Adoption & Fostering*, *41*(2), 131-141. https://doi.org/10.1177%2F0308575917702831

Devenney, K. (2017). Pathway planning with unaccompanied young people leaving care: Biographical narratives of past, present, and future. *Child & Family Social Work*, *22*(3), 1313-1321. https://onlinelibrary.wiley.com/doi/full/10.1111/cfs.12348

Eenshuistra, A., Harder, A. T., & Knorth, E. J. (2019). One size does not fit all: A systematic review of training outcomes on residential youth care professionals' skills. *Children and Youth Services Review*, *103*, 135-147. https://doi.org/10.1016/j.childyouth.2019.05.010

Eldridge, J. (2018). *Confiding in Others: A Qualitative Study Exploring the Experiences of Young people who have been in the Care System* University of Surrey Guildford].

Ferrier, M. (2011). Life story work in the context of attachment-led care planning. *Scottish Journal of Residential Child Care*, *10*(1). https://www.celcis.org/application/files/4516/2316/8859/2011_Vol_10_1_Ferrier_Life_story_work.pdf

Finlay, J. (2022). *Meaningful Natural Mentoring Relationship Characteristics and informal therapeutic life spcae interactions in youth care* Canterbury Christ Church University].

Fitzhardinge, H. (2008). Adoption, Resilience and the Importance of Stories: The Making of a Film about Teenage Adoptees. *Adoption & Fostering*, *32*(1), 58-68. https://doi.org/10.1177/030857590803200108

Furnivall, J., & Grant, E. (2014). *Trauma sensitive practice with children in care* (Insights: evidence summaries to support social services in Scotland, Issue. https://www.iriss.org.uk/sites/default/files/iriss-insight-27.pdf

Gallagher, B., & Green, A. (2012). In, out and after care: Young adults' views on their lives, as children, in a therapeutic residential establishment. *Children and Youth Services Review*, *34*(2), 437-450. https://dx.doi.org/10.1016/j.childyouth.2011.11.014

Gray, S., Hahn, R., Cater, K., Watson, D., Meineck, C., & Metcalfe, T. (2019). *Trove: A Digitally Enhanced Memory Box for Looked after and Adopted Children* Proceedings of the 18th ACM International Conference on Interaction Design and Children, https://www.researchgate.net/publication/333628573_Trove_A_Digitally_Enhanced_Memory_Box_for_Looked_after_and_Adopted_Children/link/5d31cbc4299bf1995b382ab3/download

Gustavsson, N., & MacEachron, A. (2008). Creating Foster Care Youth Biographies: A Role for the Internet. *Journal of Technology in Human Services*, *26*(1), 45-55. https://www.tandfonline.com/doi/abs/10.1300/J017v26n01_03

Gutsche, K. H. (2013). *Perceptions of social workers regarding life story work with children in child youth centres* North-West University].

Haight, W., Black, J., & Sheridan, K. (2010). A Mental Health Intervention for Rural, Foster Children from Methamphetamine-involved Families: Experimental Assessment with Qualitative Elaboration. *Child Youth Serv Rev*, *32*(10), 1146-1457. https://doi.org/10.1016/j.childyouth.2010.06.024

Hamilton, E. (2020). Life story approaches and relationships within residential child care: A practice reflection. *Relational Child and Youth Care Practice*, *33*(1),



For peer review only - http://bmjopen.bmj.com/site/about/guidelines.xhtml

87. https://www.celcis.org/application/files/8316/2307/7669/2019_Vol_18_No__2_Hamilton_E_A_practice_reflection.pdf

Hammond, S. (2012). *Exploring a role for digital technology in life story work with adolescents in residential care: a discourse analysis* University of East Anglia]. EThOS. https://ueaeprints.uea.ac.uk/id/eprint/39133/

Hammond, S. (2016). Digital Life Story Work in Action. In R. Walker & T. Ryan (Eds.), *Life story work Why, what, how and when* (pp. pp.106-119). CoramBAAF Adoption and Fostering Academy.

Hammond, S., & Cooper, N. (2013). *Digital Life Story Work: Using technology to help young people make sense of their experience.* (1 ed.). British Association of Adoption and Fostering.

Hammond, S. P., Cooper, N. J., & Jordan, P. (2021). Mental health, identity and informal education opportunities for adolescents with experience of living in state care: a role for digital storytelling. *Cambridge Journal of Education*, *51*(6), 713-732. https://doi.org/10.1080/0305764x.2021.1919057

Hanna, M. D. (2007). Preparing school age children for adoption: perspectives of successful adoptive parents and caseworkers. *Adoption Quarterly*, *10*(2), 1-32. https://www.tandfonline.com/doi/abs/10.1300/J145v10n02_01

Happer, H., McCreadie, J., & Aldgate, J. (2006). *Celebrating Success: What Helps Looked After Children Succeed*. Social Work Inspection Agency.

HCC. (2022). *Direct Work and Life Story Work with Children and Young People*. Hertfordshire County Council. Retrieved 12.08.22 from https://hertschildcare.proceduresonline.com/p_direct_life_story.html

Hills, L. (2022). In *Ensuring children's life stories are at the centre of direct work.*

Holody, R., & Mäher, S. (1996). Using lifebooks with children in family foster care a here and now process model. *Child Welfare*, *75*(4), 321-335.

Hooley, K., Stokes, L., & Combes, H. (2016). Life story work with looked after and adopted children: how professional training and experience determine perceptions of its value. *Adoption & Fostering*, *40*(3), 219-233. https://doi.org/10.1177%2F0308575916661129

Hooley, K. C. (2015). *Identifying perspectives on life story work with looked-after and adopted children* Staffordshire University]. EThOS. http://eprints.staffs.ac.uk/2244/

Hoyle, V., Shepherd, E., Lomas, E., & Flinn, A. (2020). Recordkeeping and the life-long memory and identity needs of care-experienced children and young people. *Child & Family Social Work*, *25*(4), 935-945. https://doi.org/https://doi.org/10.1111/cfs.12778

Hughes, J. I. (2013). Life-story work: a journey into a child's world. *Seen and Heard*, *23*(1), 23-30.

Humphreys, C., & Kertesz, M. (2014). Making Records Meaningful: Creating an Identity Resource for Young People in Care. *Australian Social Work*, *68*(4), 497-514. https://doi.org/10.1080/0312407x.2014.960434

Känkänen, P., & Bardy, M. (2014). Life stories and arts in child welfare: enriching communication. *Nordic Social Work Research*, *4*(1), 37-51. https://www.tandfonline.com/doi/full/10.1080/2156857X.2013.781536

Lucas, J., Matthews, L., Brady, K., Breguet, R., & Parson, J. (2020). *Therapeutic Life Story Work Barwon Pilot Program: Interim Report 2020* [Research report].



For peer review only - http://bmjopen.bmj.com/site/about/guidelines.xhtml

Improving the mental health and mental health support available to adolescents looked-after by social care via adolescent-focused low-intensity life story work: A realist review

Malik, S. (2005). *Application of attachment theory for training foster parents: A model program* https://www.proquest.com/dissertations-theses/application-attachment-theory-training-foster/docview/305340219/

Monson, K., Moeller-Saxone, K., Humphreys, C., Harvey, C., & Herrman, H. (2020). Promoting mental health in out of home care in Australia. *Health Promot Int*, *35*(5), 1026-1036. https://doi.org/10.1093/heapro/daz090

Neil, E., & Beek, B. (2020). Resepecting Children's Relationships and Identities in Adoption. In *The Routledge Handbook of Adoption* (1st ed., pp. 77-89). Routledge.

NICE. (2021). *Looked after children and young people NICE guideline*. www.nice.org.uk/guidance/ng205

Nicholls, E. (2003). Model answer. *Community Care*(1479), 32-34. https://www.communitycare.co.uk/2003/07/03/model-answer/

NSPCC. (2022). *Life Story Work NSPCC Learning*. https://learning.nspcc.org.uk/services-children-families/life-story-work#heading-top

Nuffield Family Justice Observatory. (2021). *Modernising post-adoption contact: findings from a recent consultation*. https://www.nuffieldfjo.org.uk/wp-content/uploads/2021/08/nfjo_report_adoption_connections_20210913v2.pdf

Pakrosnis, R., & Cˇepukiene, V. (2011). Outcomes of Solution-Focused Brief Therapy for Adolescents in Foster Care and Health Care Settings. In C. Franklin, T. S. Trepper, E. E. McCollum, & W. J. Gingerich (Eds.), *Solution-Focused Brief Therapy: A Handbook of Evidence-Based Practice*. Oxford Scholarship Online.

Peake, A. (2009). Life Story Work. A resource for foster carers, residential social workers, adoptive parents, and kinship carers, to support this work. . In.

Sanders, R. (2020). *ESSS Outline: Care experienced children and young people's mental health*. https://doi.org/10.31583/esss.20201012

Shepard, E. (2022). *Good practice in record-keeping in children's social care*. Research in Practice. https://www.researchinpractice.org.uk/children/news-views/2022/february/good-practice-in-record-keeping-in-children-s-social-care/

Shotton, G. (2010). Telling different stories The experience of foster/adoptive carers in carrying out collaborative memory work with children. *Adoption & Fostering*, *34*(4), 61-68. https://doi.org/10.1177%2F030857591003400407

Shotton, G. (2012). *"Remember when...": exploring the experiences of looked after children and their carers in engaging in collaborative reminiscence* University of Newcastle upon Tyne]. EThOS. http://theses.ncl.ac.uk/jspui/handle/10443/1685

Shotton, G. (2013). 'Remember when...': exploring the experiences of looked after children and their carers in engaging in collaborative reminiscence. *Adoption & Fostering*, *37*(4), 352-367. https://doi.org/10.1177%2F0308575913508721

Steenbakkers, A., van der Steen, S., & Grietens, H. (2016). 'To talk or not to talk?': Foster youth's experiences of sharing stories about their past and being in foster care. *Children and Youth Services Review*, *71*, 2-9. https://doi.org/10.1016/j.childyouth.2016.10.008

Sunderland, F. (2021). Foster Carers Handbook. In.

Teodorczuk, K., Guse, T., & du Plessis, G. A. (2018). The effect of positive psychology interventions on hope and well-being of adolescents living in a



For peer review only - http://bmjopen.bmj.com/site/about/guidelines.xhtml

child and youth care centre. *British Journal of Guidance & Counselling*, *47*(2), 234-245. https://doi.org/10.1080/03069885.2018.1504880

Together for Children Sunderland. (2019). *Foster Carers Handbook April 2019*. https://www.togetherforchildren.org.uk/sites/default/files/2019-12/Foster%20Carer%20HandbookFinalDraft16052019%20-%20Updated%20April%202019.pdf

Walker, R., & Ryan, T. (2016a). Why do life story work? In R. Walker & T. Ryan (Eds.), *Life story work Why, what, how and when* (pp. 4-17). CoramBAAF Adoption and Fostering Academy.

Walker, R., & Ryan, T. (2016b). Working with black and minority ethnic children. In R. Walker & T. Ryan (Eds.), *Life story work Why, what, how and when* (pp. 77-84). CoramBAAF Adoption and Fostering Academy.

Ward, A. (2002). Opportunity led work maximising the possibilities for therapeutic communication in everyday interactions. *Therapeutic Communities*, *23*(2), 111-124.

Watson, Hahn, & Staines. (2020). Storying special objects: Material culture, narrative identity and life story work for children in care. *Qualitative Social Work: Research and Practice*, *19*(4), 701-718. https://doi.org/10.1177%2F1473325019850616

Watson, Latter, & Bellew. (2015a). Adopted children and young people's views on their life storybooks: The role of narrative in the formation of identities. *Children and Youth Services Review*, *58*, 90-98. https://dx.doi.org/10.1016/j.childyouth.2015.09.010

Watson, Latter, & Bellew. (2015b). Adopters' views on their children's life story books. *Adoption & Fostering*, *39*(2), 119. https://doi.org/10.1177%2F0308575915588723

Watson, Staples, & Riches. (2020). 'We need to understand what's going on because it's our life': Using sandboxing to understand children and young people's everyday conversations about care. *Children & Society*, *35*(5), 663-679. https://doi.org/10.1111/chso.12432

Watson, D., Meineck, C., & Lancaster, B. (2018). Adopted children's co-production and use of 'trove' (a digitally enhanced memory box) to better understand their care histories through precious objects. *Clinical child psychology and psychiatry*, *23*(4), 614-628. https://dx.doi.org/10.1177/1359104518776359

Watts, R. (2021). Relationships, reviews and recording: Developing practice for children in care. *Practice: Social Work in Action*, *33*(3), 207-222. https://www.tandfonline.com/doi/full/10.1080/09503153.2021.1889488

Willis, R., & Holland, S. (2009). Life story work: Reflections on the experience by looked after young people. *Adoption & Fostering*, *33*(4), 44-52. https://doi.org/10.1177%2F030857590903300406

Wood, J. (2019). West Sussex Children's imporvement board. Performance Report Against the Practice Improvement Plan Improving Quality of Life Story Work.

Wood, M., & Selwyn, J. (2017). Looked after children and young people's views on what matters to their subjective well-being. *Adoption & Fostering*, *41*(1), 20-34. https://doi.org/10.1177/0308575916686034



For peer review only - http://bmjopen.bmj.com/site/about/guidelines.xhtml

Improving the mental health and mental health support available to adolescents looked-after by social care via adolescent-focused low-intensity life story work: A realist review

# Supplementary File 4: Initial practice guidelines

| Initial practice guidance recommendation |
| --- |
| **1. AF-LI-LSW should be flexible and person-centred** to ensure how, why, when, by whom and to what extent AF-LI-LSW is delivered, fits the needs of the individual adolescent and their circumstances. |
| **2. There is no 'bad time' to start AF-LI-LSW so this should start early and make use of everyday opportunities.** Everyday opportunities should be used to engage the adolescent in preserving and reflecting on their life experiences. |
| **3. AF-LI-LSW should begin in the present day as this grows trust in caring adult(s) and relationships.** Sharing everyday life experiences helps to establish trusting relationships with caring adult(s). |
| **4. AF-LI-LSW should involve co-construction of narratives because this improves coherence and authenticity.** Adolescents should be supported to construct a narrative that makes sense and feels authentic to them. |
| **5. Adolescents should be supported to control how their lives are recorded and preserved in AF-LI-LSW whenever and wherever possible. A**dolescents should control the processes involved so they feel ownership and power over their own story and how and what is captured. |
| **5.1 Adolescents should be supported to control the processes for recording and preserving** |
| **5.2 Adolescents should be supported to control AF-LI-LSW products (e.g., artefacts)** |
| **6. AF-LI-LSW should provide adolescents with somewhere to begin future telling,** supporting adolescents' communication about their lived experiences by providing storytelling prompts, helping adolescents through transitions. |
| **7. AF-LI-LSW should record positive aspects of everyday experiences, there should be a** focus on the positives in adolescents lives to promote the development of positive identity and positive future expectations. |
| **8 AF-LI-LSW should include support for adolescents, caring adults and others involved** adolescents, caring adults and others supporting adolescents with life story work should be adequately prepared and/or trained and supported themselves. |



For peer review only - http://bmjopen.bmj.com/site/about/guidelines.xhtml

BMJ Open

# Improving the mental health and mental health support available to adolescents in out-of-home care via adolescent-focused low-intensity life story work: A realist review

| | |
|---|---|
| Journal: | *BMJ Open* |
| Manuscript ID | bmjopen-2023-075093.R1 |
| Article Type: | Original research |
| Date Submitted by the Author: | 21-Jul-2023 |
| Complete List of Authors: | Hammond, Simon; University of East Anglia, School of Education and Lifelong Learning<br>Mickleburgh, Ella; Norfolk and Suffolk NHS Foundation Trust, Research<br>Duddy, Claire; University of Oxford, Nuffield Department of Primary Care Health Sciences<br>Hiller, Rachel; University College London, Division of Psychology and Language Sciences<br>Neil, Elsbeth; University of East Anglia, School of Social Work<br>Blackett, Rosie; National House Project<br>Williams, Kevin; The Fostering Network<br>Wilson, Jon; Norfolk and Suffolk NHS Foundation Trust<br>Wong, Geoff; Oxford University, Nuffield Department of Primary Care Health Sciences |
| <b>Primary Subject Heading</b>: | Mental health |
| Secondary Subject Heading: | Evidence based practice, Health services research, Paediatrics |
| Keywords: | Adolescent, MENTAL HEALTH, Systematic Review |
| | |

SCHOLARONE™
Manuscripts

*I, the Submitting Author has the right to grant and does grant on behalf of all authors of the Work (as defined in the below author licence), an exclusive licence and/or a non-exclusive licence for contributions from authors who are: i) UK Crown employees; ii) where BMJ has agreed a CC-BY licence shall apply, and/or iii) in accordance with the terms applicable for US Federal Government officers or employees acting as part of their official duties; on a worldwide, perpetual, irrevocable, royalty-free basis to BMJ Publishing Group Ltd ("BMJ") its licensees and where the relevant Journal is co-owned by BMJ to the co-owners of the Journal, to publish the Work in this journal and any other BMJ products and to exploit all rights, as set out in our licence.*

*The Submitting Author accepts and understands that any supply made under these terms is made by BMJ to the Submitting Author unless you are acting as an employee on behalf of your employer or a postgraduate student of an affiliated institution which is paying any applicable article publishing charge ("APC") for Open Access articles. Where the Submitting Author wishes to make the Work available on an Open Access basis (and intends to pay the relevant APC), the terms of reuse of such Open Access shall be governed by a Creative Commons licence – details of these licences and which Creative Commons licence will apply to this Work are set out in our licence referred to above.*

*Other than as permitted in any relevant BMJ Author's Self Archiving Policies, I confirm this Work has not been accepted for publication elsewhere, is not being considered for publication elsewhere and does not duplicate material already published. I confirm all authors consent to publication of this Work and authorise the granting of this licence.*

For peer review only

# Improving the mental health and mental health support available to adolescents in out-of-home care via Adolescent-Focused Low-Intensity Life Story Work: A realist review

Simon P Hammond[ab]*, Ella Mickleburgh[b], Claire Duddy[c], Rachel Hiller[de], Elsbeth Neil[f], Rosie Blackett[g], Kevin Williams[h], Jon Wilson[b], Geoff Wong[c].

*[a]School of Education and Lifelong Learning, University of East Anglia (UEA), Norwich, United Kingdom (UK); [b]Norfolk and Suffolk NHS Foundation Trust, Norwich, UK, [c]Nuffield Department of Primary Care Health Sciences, University of Oxford, UK;* [d]Division of Psychology and Language Sciences, University College London, UK; [e]Anna Freud National Centre for Children and Families, London; *[f]Centre for Research on the Child and Family, School of Social Work, UEA, Norwich, UK; [g]The National House Project, UK; [h]The Fostering Network, UK.*

**Corresponding Author.*

*School of Education and Lifelong Learning, Faculty of Social Sciences, University of East Anglia. Norwich. NR4 7TJ. United Kingdom. Tel: (+44) (0)1603 591460. Email: s.hammond@uea.ac.uk*

*Keywords*

Adolescent, life story work, realist review, mental health, mental health support

Word count: 5,748 (excluding references and table)



For peer review only - http://bmjopen.bmj.com/site/about/guidelines.xhtml

# Abstract

**Objectives:** Life Story Work (LSW) is used to promote the mental health of children and adolescents living in out-of-home care. LSW should be offered to all but is conventionally delivered in high-intensity ways. Low-intensity approaches are more accessible but there is significant variation and little guidance for supporting adolescents. We aimed to create guidance for Adolescent-Focused Low-Intensity LSW.

**Design:** Realist review

**Data sources:** MEDLINE, Embase, PsycINFO, Sociology Collection (Proquest), CINAHL, CDAS, Web of Science (SCIE, SSCI), Social Care Online and grey literature sources. Searches were performed between December 2021-March 2022

**Eligibility criteria:** Documents on children and adolescents in care, LSW and/or low-intensity interventions to improve mental health were included. Documents focusing on parenting style and contact with birth family were excluded.

**Analysis:** Documents were analysed using a realist logic of analysis. In consultation with Content Expert Groups (comprising professionals and care leavers), we developed an initial programme theory. Data relating to and challenging the initial programme theory were extracted and Context-Mechanism-Outcome-Configurations developed, critiqued, and refined in an iterative fashion. Interpretations were drawn from Context-Mechanism-Outcome-Configurations to enhance the programme theory.

**Results:** 75 documents contributed to the analysis. Generally, studies were small-scale and lacked in-depth methods and evaluation descriptions. Findings indicated important factors contribute to the development of high-quality Adolescent-Focused Low-Intensity LSW. Adolescent-Focused Low-Intensity LSW should be person-centred, begin in the now, involve co-construction, record everyday positive life events, and be supported by trained carer(s). Context-mechanism-outcome-configurations relating to these themes are reported.

**Conclusions:** Using this knowledge we developed of initial practice guidance to support social care to deliver better quality Adolescent-Focused Low-Intensity LSW more consistently. To address gaps in our knowledge about the impact of Adolescent-Focused Low-Intensity LSW, further primary research is needed to strengthen understandings of how this intervention works (or not) in different contexts.

**PROSPERO registration number:** CRD42021279816.




For peer review only - http://bmjopen.bmj.com/site/about/guidelines.xhtml

## Strengths and limitations of the study

- This is the first realist review of Adolescent-Focused Low-Intensity Life Story Work and it improves our understanding of how this intervention may work for adolescents in out-of-home care.
- The review benefited from ongoing consultations with stakeholders in two separate Content Expert Groups, featuring young adults with care-experience and professionals, as recipients and deliverers of Adolescent-Focused Low-Intensity Life Story Work.
- Content Expert Groups identified gaps in the evidence base, meaning there were limitations in how these aspects could be incorporated into the developing programme theory.
- Future primary research is needed: (i) to further consolidate Adolescent-Focused Low-Intensity Life Story Work components (elements that cannot be changed) and its adaptable periphery (elements that can be changed), (ii) build on initial practice guidance offered by this paper (iii) to identify important outcomes for adolescents in care and those who support them (iv) and deliver a comprehensive evaluation to enable the therapeutic potential of Adolescent-Focused Low-Intensity LSW to be examined.

For peer review only

# Background

There are currently over 105,000 children and adolescents removed from their homes and looked-after in out-of-home care in for example, foster or residential placements, in the United Kingdom (UK) [1-4]. Within this population, adolescents living in out-of-home care (henceforth adolescents in care) are the largest group currently in, and fastest growing age group entering care in England [1]. They are amongst the most vulnerable members of society and are six times more likely than the general population to experience mental illness [5, 6]. The National Institute for Health and Care Excellence (NICE) describe the need to increase the evidence-base for effective mental health interventions for adolescents in care as an "urgent research priority" [7].

Life Story Work (LSW) is an existing transdiagnostic intervention thought to improve the well-being and mental health of those living in care [8]. It aims to improve individuals' sense of identity, relationships with important adults, ethnic heritage, placement stability, and mental health, by addressing gaps in self-knowledge, reframing past events, and increasing positive future expectations [7-13]. Typical LSW components include a therapeutic alliance (relationship with caring adults), procedures (prompts to action), products (materials/artefacts/life story books), and therapeutic activities (rescripting/reframing).

The intensity of LSW varies along several continua including whether delivery involves support from a specialist professional or not, delivery mode (face-to-face and/or digital), duration (prescribed sessions or ongoing) and intensity of services provided (e.g., past and/or trauma-focused or child led) [14, 15]. LSW is often delivered in a high-intensity way, involving specialist professionals (e.g., Social Workers, Educational Psychologists, Clinical Psychologists, Psychotherapists) input over many sessions and months. This is potentially expensive and impractical, limiting its availability. LSW also tends to be delivered to younger children (aged 0-11 years) meaning adolescents in care (12-18 years old) often miss out [16].

LSW can be delivered in a lower-intensity way, for example, by foster carers. This increases its accessibility and is sometimes called 'low-intensity' LSW. Low-Intensity Life Story Work (LI-LSW) usually involves a carer (therapeutic alliance) recording (prompts to action) potentially valuable parts of a child's every day present circumstances (product creation). The carer and child discuss these, helping the child to process and consolidate their experiences (therapeutic activities). This in turn serves to generate positive future expectations and helps a therapeutic alliance develop between child and carer [13, 17].

NICE states LSW should be offered to all those living in care [7]. There is currently no accepted standard for LI-LSW delivery or high-quality evidence-based guidance and yet evidence has repeatedly highlighted the harms of poorly undertaken LSW [18-25]. What little guidance there is focuses mainly on younger children in care [20, 26-28] The current lack of 'Adolescent-Focused' LI-LSW guidance means adolescents in care experience ongoing inconsistent practices and mental health inequalities leaving them and their carers feeling unsupported [28]. Adolescence is an important period where concepts of self and identity are developing as well as social and emotional practices that are influential to well-being [29]. Therefore a whole-child approach is necessary to ensure adolescents gain the potential benefits of LSW, particularly when delivered taking a low-intensity approach.

To address this evidence and practice gap, we aimed to generate in-depth knowledge to understand if LSW can be delivered to adolescents in care as a low-intensity intervention by asking:

*How, why, to what extent, for whom and in what circumstances can low-intensity LSW interventions, or elements of LSW interventions, be delivered to improve important and*



For peer review only - http://bmjopen.bmj.com/site/about/guidelines.xhtml

*relevant outcomes for adolescents with care experience with mental health and wellbeing needs?*

The end goal of the project was to develop a set of initial guidelines for optimising Adolescent-Focused Low-Intensity LSW to improve the mental health and mental health support offered to adolescents in care.

# Methods

We conducted a realist review to synthesise evidence to help us understand the importance of context in the delivery of Adolescent-Focused Low-Intensity LSW, and the mechanisms by which outcomes, both intended and unintended, are produced. Understanding what works, for whom, in what respects, to what extent, in what contexts is vitally important as adolescents in care are an extremely heterogeneous and complex group interacting with a wide variety of complex services [28]. A realist review was selected because Adolescent-Focused Low-Intensity LSW is a complex intervention and how well it works (or not) depends on context, who delivers it, and how, with context-sensitive outcomes particularly likely [8, 16]. Producing knowledge to understand this complexity will enable commissioners and implementors of Adolescent-Focused Low-Intensity LSW to make more informed decisions about if, how, when and where it may be useful.

## Realist review

Realist approaches are grounded in the assumption that the same intervention will not work everywhere and for everyone [30]. A realist review is a theory-driven interpretive type of literature review. Realist reviews are particularly useful for generating knowledge about how complex interventions produce mixed outcomes by generating better understandings of how and why different outcomes occur [31-33]. Central to realist reviews is the creation and iterative refinement of a programme theory. A programme theory describes how an intervention is expected to lead to its outcomes and in which contexts these should occur [31-33].

Realist reviews begin with an initial programme theory, developed from previous research, lived-experiences, and assumptions about how an intervention works. Literature searching is then undertaken, data extracted and synthesised using a realist logic of analysis to develop context–mechanism–outcome configurations (CMOCs). CMOCs are causal explanations describing how and why particular outcomes are generated in particular contexts [31-33]. CMOCs are key components that inform any programme theory as they underpin any causal claims made.

Realist reviews can draw on a breadth of data sources to inform the programme theory. This was important due to the low-quality of the majority LSW literature and the aim of the current review to understand the various contexts within which Adolescent-Focused Low-Intensity LSW may occur [16]. Hence, it was important to not limit included documents to solely published academic literature. Conducting a realist review enabled the inherent complexity of the research question to be addressed by using any relevant data that accounted for the different settings and services adolescents in care may experience. Through its focus on commonly occurring causal processes that underlie LSW, the realist approach also potentially provides transferable explanations for how and why other low-intensity mental health intervention strategies 'work' (and do not work) for adolescents in care. This enabled the study to begin to address knowledge gaps highlighted in previous work relating to improving implementation guidance [8, 16].



For peer review only - http://bmjopen.bmj.com/site/about/guidelines.xhtml

In this paper, we report on a realist review study conducted between September 2021 and January 2023. A five-step process for conducting the realist review was undertaken following published standards [32, 33]. These steps are summarised in Table 1 below, with Table 2 showing eligibility criteria, with full details about our methods available in our open-access protocol paper [9].

## Data sources searched

As per our protocol paper[9], electronic database searches were performed between December 2021 and March 2022. Eight databases were searched: MEDLINE, Embase, PsychINFO, Sociology Collection (Proquest), CINAHL, CDAS, Web of Science and Social Care Online. To retrieve relevant PhD theses, we also searched EThoS (British Library) and Proquest Dissertations and Theses. Targeted Google searches were performed to retrieve grey literature produced by local authorities and further documents were obtained through citation tracking and team members knowledge. The precise full search strategy is shown in Supplementary File 1.

INSERT TABLES 1 & 2

## Patient and Public Involvement – working with experts via experience

The idea for the project from which this paper reports was driven by the lead authors time in practice and shaped through working with a variety of experts via experience from this conception. This review was informed by the involvement of two expert via experience groups. These groups consisted of a Care-experienced Content Expert Group which involved care leavers (young adults with recent experiences of living in social care), and a professional expert panel (known as the Content Expert Group) which comprised of multidisciplinary professionals with divergent topic area knowledge and/or experiences (i.e., Foster Carers, Social Workers, Commissioners, Educational and Clinical Psychologists).

We met with the Care-experienced Content Expert Group and Content Expert Group three times during the project. We asked these groups to review information, bring their own experiences to bear on this, and help us to critique and refine the developing programme theory . These discussions helped clarify important areas that needed explanation (e.g., the need for adolescent-focused low-intensity LSW to be flexible) and where the existing literature was lacking (e.g., peer-support for adolescents engaging with adolescent-focused low-intensity LSW ), thus shaping our analysis. In our final meetings, we asked each group to help us to develop and refine an initial set of guidelines. Both groups also helped develop dissemination strategies, ensuring project outputs were tailored to different audiences.

# Results

## Document characteristics

In total, 75 documents were included in the review. Documents were published between 1981-2022 and were from 8 countries (56 from the UK). Included documents were research articles (36%), commentaries or practice guidance (32%), web sources (3%), reports (5%), conference paper (1%), quality standards (1%), books or book chapters (11%) and theses (11%). Of the research articles, 78% were qualitative studies, 4% used mixed methods, 7% were literature reviews, 7% were matched pairs and 4% were randomised trials. Judgements were made concerning the relevance of data within documents and whether data could be used to improve our understanding of how Adolescent-Focused Low-Intensity LSW could be implemented. The main reasons for exclusion were documents being about the wrong



For peer review only - http://bmjopen.bmj.com/site/about/guidelines.xhtml

population or not containing information that contributed relevant data to the context, mechanism, or outcome configurations for Adolescent-Focused Low-Intensity LSW. The processes of identifying, screening, and selecting documents are illustrated in Figure 1: An adapted version of the PRISMA flow diagram. A detailed summary of included documents are available in Supplementary File 2.

INSERT FIGURE 1 – PRISMA

## Review findings

INSERT FIGURE 2 – INITIAL PROGRAMME THEORY

The initial programme theory presented a linear explanation of Adolescent-Focused Low-Intensity LSW (see Figure 2). What was clear within the literature and from consultations with our Care-experienced Content Expert Group and Content Expert Group was the current low-quality of the evidence-base, alongside the need for Adolescent-Focused Low-Intensity LSW to be both continual and flexible. Data synthesis resulted in a consolidated programme theory from which a set of initial guidelines for optimising Adolescent-Focused Low-Intensity LSW to improve the mental health and mental health support offered to adolescents in care was created (see Figure 3). Eight initial guidelines are outlined below and also concisely summarised in Supplementary File 3). The narrative summary accompanying each guideline is based on our realist analysis that developed 51 CMOCs, underpinned by the data included in the review. To enhance transparency, we provide a detailed summary of the CMOCs developed and the data underpinning each in Supplementary File 4. We also provide references to illustrate CMOCs to support the report of the results of the review that follows.

INSERT FIGURE 3 – CONSOLIDATED PROGRAMME THEORY

**1. Adolescent-Focused Low-Intensity LSW should be flexible and person-centred (CMOC 1)**

Ten documents contributed to the development of this CMOC [7, 20, 27, 34-40]. The review's core finding is the importance of adolescent-focused low-intensity LSW being flexible and person-centred. Adapting to the needs, interests and wishes of the adolescent is the key initial guideline that underpins all that follow.

> *'Take a flexible approach to life story work, and tailor it to the developmental age and needs of the looked-after child or young person' [7]*

**2. There is no 'bad time' to start Adolescent-Focused Low-Intensity LSW so this should start early and make use of everyday opportunities (CMOCs 2-7).**

Thirty-one documents contributed to the development of these CMOCs [7, 19-22, 27, 34-36, 38, 39, 41-59]. The review identified two factors associated with when to begin, and how to initiate Adolescent-Focused Low-Intensity LSW. It should start immediately and take advantage of everyday opportunities. Beginning early helps preserve information and avoid narrative gaps. This ensures that adolescents have memory prompts available to begin to construct a coherent identity, with these prompts containing everyday events.

> *"Entries assist in helping the youth preserve memories…. These stories may someday help the youth establish a stronger sense of self based on positive memories of their past.' [50].*



For peer review only - http://bmjopen.bmj.com/site/about/guidelines.xhtml

A caveat within conventional, 'high-intensity' LSW literature is that it should begin at the 'right time'. This 'right time' is often described as when a child is in a stable placement and has trusting relationships to support them as this work involves processing their past, potentially traumatic experiences [27, 34]. This is often a barrier to any LSW being implemented. However, an Adolescent-Focused Low-Intensity LSW approach focuses on an adolescent's everyday life and future, avoiding an over-emphasis on past experiences and freeing carers to begin this work immediately. This relationship may then serve as a vehicle to explore more sensitive previous experiences. However, higher intensity LSW approaches are not the focus of this review and represent a distinct area of need.

**3. Adolescent-Focused Low-Intensity LSW should begin in the present day as this grows trust in caring adult(s) and relationships (CMOCs 8-10, 12-18, 30)**

Forty-three documents contributed to the development of these CMOCs [7, 19-21, 27, 34, 37, 38, 40, 41, 46-49, 52-55, 59-81]. Difficulties in establishing and maintaining trusting relationships was a main theme. Adolescent-Focused Low-Intensity LSW should provide a platform to engage in positive activities between caring adults and adolescents, encouraging one-to-one time together and increasing communication [7, 19, 27, 34, 37-39, 47, 60-64]. These shared activities should encourage increased communication between the adolescent and caring adults, strengthening relationships, and helping to grow trust and a therapeutic alliance between the adolescent and caring adult(s). This helps the adolescent to understand themselves better as well as helping the caring adult become better attuned to the adolescent's needs, improving their ability to care for them effectively.

> *'Young people seemed to trust relationships where others would sensitively listen to their confidences, whilst remaining unconditionally accepting of them as a person…'[63].*

As well as contributing to interpersonal outcomes such as increased connection, communication, sense of belonging, enhanced ability to understand others behaviour and placement stability, the literature indicated the potential value of Adolescent-Focused Low-Intensity LSW has on intra-personal outcomes such as increased self-worth and emotion regulation. Adolescents can develop their emotional regulation skills through discussing thoughts and feelings and having a caring adult modelling adaptive behaviours to challenging experiences.

> *'If adults respond to children's distress in a calm but engaged way they demonstrate an alternative way of managing stress..' [82].*

**4. Adolescent-Focused Low-Intensity LSW should involve co-construction of narratives because this improves coherence and authenticity (CMOC 23-29 and 31)**

Forty-four documents contributed to the development of these CMOCs [18-22, 26, 27, 34-39, 41, 42, 45, 46, 48, 49, 52-55, 57, 59, 63, 65, 68, 69, 71, 74, 77-79, 83-92]. The review shows narratives most valued by adolescents were constructed collaboratively with caring adult support. The therapeutic alliance enabled adolescents to feel supported to see a range of perspectives when interpreting events. When adolescents are actively involved in the co-construction of narratives, these narratives become more coherent and feel authentic because they reflect adolescents' lived experiences.



For peer review only - http://bmjopen.bmj.com/site/about/guidelines.xhtml

*'The therapeutic process involves creating opportunities to open up conversational spaces. These spaces provide young people with opportunities to share accounts of their own lives, in their own words.'* (48).

Literature indicates that adolescents in care may construct self-limiting accounts of their lived experiences due to their chronic and/or traumatic nature [39, 41, 49, 93]. Evidence indicates that poorly executed LSW has negative impacts [18-22]. Having caring adult support can help adolescents reconstruct narratives, reducing self-blame and self-limiting interpretations of events and circumstances. When alternatively framed narratives are available, adolescents can become increasingly aware of their positive attributes. This can increase self-esteem and the availability of positive future expectations.

*'There is something powerful in how people come to see their stories and make sense of their experiences. This is the heart of resilience – the ability of a person to understand their story in such a way that it creates opportunity rather than limits it.' [79].*

The inclusion of multiple voices and perspectives increases adolescent's awareness that there are different ways to interpret situations and circumstances. Relevant literature was limited in this area, perhaps pointing towards its inherent complexity and clashes between adolescent agency and professionalised notions of 'accuracy'.

*'Granting children control over narratives aids identity formation but left unchallenged their perspective of events may also become one-sided and inaccurate' [39].*

However, both of our stakeholder groups indicated the value of including significant individuals, including birth relatives and siblings in Adolescent-Focused Low-Intensity LSW. Literature indicated that when/if significant individuals are positively involved in Adolescent-Focused Low-Intensity LSW activities, this can facilitate the establishment of stronger relationships because of the interactions that result from the activity. The involvement of significant individuals may also increase adolescents' sense of identity because they are able to access and process information about their heritage.

**5. Adolescents should be supported to control how their lives are recorded and preserved in Adolescent-Focused Low-Intensity LSW whenever and wherever possible (CMOC 32-42)**

Adolescents should control how their everyday life experiences are reflected on and preserved including the processes used for recording and preserving lived experiences as well as the resulting products.

**5.1 Processes for recording and preserving (CMOC 32-36)**

Twenty-six documents contributed to the development of these CMOCs [7, 18, 20, 22, 27, 34-37, 39, 40, 45-48, 51, 53, 55, 62, 64, 65, 78, 94, 95]. The review indicates that being able to exert control over the process increases adolescents' sense of ownership. This helps adolescents engage better and feel their opinions are valued. This is invaluable for adolescents in care as often due to their circumstances their sense of power can be reduced.

*'Children can have ownership of their story work via choosing which objects to story/not story and by dictating the pace at which the work progresses… (FSW, Ben)' [39]*



For peer review only - http://bmjopen.bmj.com/site/about/guidelines.xhtml

There was limited literature surrounding how, when and who should be responsible for making LSW accessible for adolescents with special educational needs. The available literature and our stakeholder groups indicated the importance of the adolescents' support network feeling equipped with the knowledge and skill to make adaptions where necessary. Caring adult(s) will benefit from, for example, training materials developed by speech and language therapists. This may include written and visual resources that increase their understanding of how to make adaptations to improve adolescent's abilities to engage with Adolescent-Focused Low-Intensity LSW in a meaningful way. Though research was sparse in this area, our Care-experienced Content Expert Group and Content Expert Group stated this was important to ensure that adolescent-focused low-intensity LSW is flexible and person-centred.

**5.2 Products for recording and preserving (e.g., artefacts) (CMOCs 37 – 42)**

Thirty-one documents contributed to the development of these CMOCs [18, 20-22, 26, 27, 34, 35, 38-42, 45, 46, 48-50, 52-55, 78, 85, 90, 94-98]. The literature highlighted that when an adolescent has editorial power to control what is recorded, they can feel more positive about the product(s). This is because what is recorded aligns more closely with their lived experiences. The risk of LSW products being lost or damaged was a theme within the literature. Literature and our CCEG and CEG repeatedly illustrated the benefits of digital tools/platforms to create and store editable products. These products were cited as holding meaning over time because they held identity information about adolescents, their relationships, and spaces at a point in time and could be updated and edited when needed.

> *'Digital tools offer the ability to help young people to express thoughts and feelings which can be continually updated, edited and reflected upon. Using interactive computer-based mediums in this way grants the user flexibility to make changes frequently and easily.' [45]*

The importance of both physical, audio, and visual artefacts was emphasised. Physical objects can increase a sense of connection to others and/or past experiences via the tangible nature of the artefact that was present during life experiences. Audio and visual formats, including capturing the actual voice or video recording of significant individuals can be powerful, helping adolescents to situate memories and feel connected to their experiences and significant individuals.

> *'For looked after and adopted children, physical objects are often the only remaining link to their past; a portal to stories of birth families, former homes, and significant people.' [39]*

**6. Adolescent-Focused Low-Intensity LSW should provide adolescents with somewhere to begin future telling (CMOC 11 and 43)**

Eight documents contributed to the development of CMOCs [34, 35, 38, 39, 41, 49, 62, 75]. Our stakeholder groups repeatedly emphasised the life course nature of care-experience and enduring impacts that missing information has on identity and self-esteem throughout one's life. During adolescents' time in care, they can use their collection of preserved memories as storytelling prompts (e.g., a photo with a date, time, location, names of people and activity relating to) with caring adults, which helps others to understand them, strengthening relationships. This may include transitions within and beyond care. When preserved, life



For peer review only - http://bmjopen.bmj.com/site/about/guidelines.xhtml

experiences can become a bank of narrative starting points (artefacts from which to begin reminiscing) for adolescents to share stories about themselves with future audiences.

> '*The book acted as an aide to him telling me about his life, the things and people that are important to him.' [49]*

**7. Adolescent-Focused Low-Intensity LSW should record positive aspects of everyday experiences (CMOC 44-46)**

Twenty-one documents contributed to the development of these CMOCs [19, 20, 26, 27, 34, 37, 38, 47, 49, 52, 54, 59, 66, 69, 80, 81, 86, 96, 99, 100]. The review highlighted the value of using Adolescent-Focused Low-Intensity LSW to capture adolescents' 'everyday magic' (i.e., an individual's mannerisms, characteristics, and their idiosyncrasies, humour and histories of relationships shared with those around them and everyday successes) helping adolescents to feel more positive about themselves and their relationships [38, 39, 47, 49, 52, 66, 80, 81, 86, 99]. When achievements are preserved and reflected adolescent's ability to develop positive identities and future expectations are supported because the availability of positive self-narratives for interpreting their experiences are more readily available. They can become better at identifying future achievements themselves.

> *'…it can help the child to thicken positive counter-narratives impacting positively on the dominant stories the child has and is able to tell about themselves, particularly thickening stories around their strengths, worth and belonging.' [49].*

**8. Adolescent-Focused Low-Intensity LSW should include support for adolescents, caring adults and others involved (48-51)**

Twenty-five documents contributed to the development of these CMOCs [7, 27, 34, 37-39, 41, 48, 49, 51, 54, 55, 58-60, 66, 68, 70, 72, 77, 79, 82, 87, 96, 101]. As mentioned above, Adolescent-Focused Low-Intensity LSW should be flexible and person-centred. The literature highlights the essential role confidence has on the ability of caring adults to support Adolescent-Focused Low-Intensity LSW. It is important that caring adult(s) understand the adolescents' individuality and cultural heritage to develop their awareness of the potential significance of everyday lived experiences to promote appropriate preservation and reflection. Caring adults' need to be supported via training and ongoing supervision to improve their confidence and skills to support and facilitate Adolescent-Focused Low-Intensity LSW.

> *''Training to ensure a consistent approach to life story work could be incorporated into existing training.' [7]*

The literature indicated that training is most impactful when accompanied by supervision to consolidate learning. Supervision for caring adults ensures advice and guidance is readily available as well as providing a self-reflective space to process emotions and improve practice.

> *''Recognition of children's entitlement to a coherent narrative needs to be embedded in practice at the micro level. This can only occur if appropriate supports are in place at the organisational level, facilitated by macro-level priority….' [41]*

Alongside providing training and supervision for caring adults, the potential role of peer-support, for adolescents and caring adults and when implementing Adolescent-Focused Low-



For peer review only - http://bmjopen.bmj.com/site/about/guidelines.xhtml

Intensity LSW was repeatedly mentioned by our expert groups. Adolescents may benefit from peers supporting their understanding of Adolescent-Focused Low-Intensity LSW as during adolescence it is widely acknowledged that peers become increasingly influential [102], meaning there is the potential for peer support to be impactful. Peer-support for caring adults also hinted at ways to develop communities of practice. Literature was extremely limited on the role of peer-support for both groups. However, other models of practice have employed peer support models with these populations with promising outcomes (e.g., The Mockingbird programme). This is an important direction for future research.

# Discussion

## Summary

Adolescent-Focused Low-Intensity LSW involves an adolescent and/or caring adult(s) recording potentially valuable parts of an adolescent's everyday life (e.g., diary entry or picture). The caring adult(s) and adolescent discuss these, helping the adolescent to process and consolidate their experiences. This in turn serves to generate positive future expectations and helps a therapeutic alliance develop between adolescent and caring adult(s) [7, 27, 37, 38, 47, 49, 61-63, 84-86, 88].

## Comparison with existing literature

In a recent systematic review targeting the mental health and wellbeing of care-experienced children and young people [103], only one paper related to LSW was included [66]. The authors of the systematic review described the paper as providing a limited description of intervention implementation and acceptability [103]. This dearth of good quality evidence on LSW as a whole is contrary to repeated high-quality evidence illustrating that key components that make up LSW are effective. For example, firstly we know that being able to express emotions, change negative cognitions, recall positive life experiences, create positive future expectations and therapeutic alliances are key targets to promote mental health, especially for vulnerable adolescents [13, 17, 28, 104-106]. Secondly, we know that trusting relationships with key adult(s) and peer(s) are frequently indicated as important in recovering from traumatic life experiences beyond childhood [107, 108]. Thirdly, we know that positive psychological and reminiscence interventions are impactful [11]. Finally, we know that better access to high-quality mental health support can build self-esteem and belonging, themes frequently cited in reports about the mental health needs of adolescents in care [28, 103, 104, 109]

Systematic review evidence examining LSW interventions for children and adolescents in care concluded that LSW had potentially far-reaching benefits but an "immature" evidence-base [12, 110]. However, the existing evidence does indicate that the lack of "robust implementation guidance, efficacy or cost-effectiveness studies" [16] is problematic and that "greater implementation knowledge" is needed to examine therapeutic outcomes [12]. A recent attempt to improve this evidence-base was inconclusive [8], with this rare example of a Randomised Controlled Trial in this area ultimately lacking statistical power. The authors reflect on a number of design issues including 71% of the young people in the active arm not receiving the intervention, a lack of carer engagement due to restrictive intervention processes and issues with the extraction of the selected primary outcome measure from social care records [8]. However, the process evaluation did indicate widespread positive qualitative feedback with young people reporting feeling more connected to carers. Echoing earlier systematic review findings, authors concluded that any future trial must be sufficiently powered and sensitive to how context shapes intervention delivery [8].



For peer review only - http://bmjopen.bmj.com/site/about/guidelines.xhtml

## Limitations

This review has several limitations. The literature that informed the CMOCs were supported by relevant and best currently available evidence. However, when framed by the hierarchy of evidence for therapeutic studies [111, 112] most of the available evidence is 'low-quality' [8, 28, 113-115]. Existing literature was mainly qualitative in nature, tended to focus on LSW undertaken with younger children (0-11 years old) and higher intensity delivery models. Whilst the available evidence with an adolescent focus was invaluable for illustrating 'good' and 'bad' practices, it did not provide enough detailed information about implementation or how different circumstances, including different placement contexts impact on outcomes or how Adolescent-Focused Low-Intensity LSW operated over time. This is problematic from a realist perspective since the same body of qualitative evidence is used to inform the Contexts, the Mechanism and the Outcomes meaning the review teeters on the edge of working in one data medium method rather than being multi-method [116]. This means that we were unable to effectively test the programme theory and, in particular, the potential of contextual impacts on intervention effectiveness. This is a substantial gap in our understandings of pre-existing practice. It must be urgently addressed as understanding what works, for whom, in what respects, to what extent, and in what contexts is vitally important as adolescents in care are an extremely heterogeneous group interacting with a wide variety of complex services [28, 103]. How context influence mechanisms and outcomes is therefore vital.

The current review adds to this by improving our understanding of how, why, what and whom should be involved in the delivery of Adolescent-Focused Low-Intensity LSW to produce potentially therapeutic outcomes. Our realist review, the first of its kind in this area, created a programme theory for Adolescent-Focused Low-Intensity LSW and used this to produce initial guidance for how current practice should be improved. Our review is unique in describing considerations that support adolescents in care to experience ownership over how, when, why, and in what contexts their everyday lived experiences are narrated, recorded, and reflected upon during their time living in social care.

A flexible and person-centred approach is key, both in generating feelings of investment and empowerment but also in implementing Adolescent-Focused Low-Intensity LSW. An important task for caring adults is identifying and empathising with adolescents, particularly in relation to their desired level of involvement within Adolescent-Focused Low-Intensity LSW, which may fluctuate over time and across contexts. This review illustrates that Adolescent-Focused Low-Intensity LSW is likely have core components (elements that cannot be changed) and is likely to have an 'adaptable periphery' (elements that can be changed) capable of adjusting to contextual factors [117]. More research is need to identify these components to allow learning to be transferable between contexts.

## Implications for practice

Adolescent-Focused Low-Intensity LSW is not a magical solution to improving the mental health outcomes for adolescents in care. However, considering National Institute for Health and Care Excellence states LSW should be offered to all those living in care [7], that it is endorsed by legislation underpinning its usage [118-121] and widely valued by those living and working within social care [10, 18-22, 27, 34, 81], we hope our initial practice guidance (summarised in Supplementary File 3) can begin to improve existing practices. In implementing this guidance, there is a need to consider where, how, and in what format Adolescent-Focused Low-Intensity LSW products are stored, who has ownership of this content, and how it can be transferred when those living in care move within and beyond the care system. Our Care-experienced Content Expert Group and Content Expert Group both



For peer review only - http://bmjopen.bmj.com/site/about/guidelines.xhtml

repeatedly highlighted the potential of digital platforms/devices. They felt that a digital platform had the potential to be effectively used within Adolescent-Focused Low-Intensity LSW to record and collate everyday life experiences. This is something that members of the team have written about before [35, 45, 46, 122]. However, existing research was sparse in this area and research to understand the value of digital platforms to support LSW, such as CaringLife, is ongoing currently.

### Implications for research

Given the complexity of Adolescent-Focused Low-Intensity LSW it is perhaps unsurprising that many studies reported variation in interventions and their implementation. However, there were also issues within many studies where the description of both were either absent or underdeveloped. This can be remedied by researchers using tools such as the TIDierR or StaRI checklists to enable authors to efficiently outline interventions and report their implementation in detail [123, 124]. Furthermore, to develop CMOCs or some aspects of CMOCs which were only supported by limited evidence, we drew on expert opinion. These CMOCs were discussed with our expert groups who felt they were important to include. Gaps in these areas, (e.g., use of digital media, peer-to-peer support, engaging with adolescents in care with Special Educational Needs, and involving significant individuals (including birth relatives), effectiveness and implementation of training and supervision) also represent important future research directions.

Further research is required to strengthen our understanding of how Adolescent-Focused Low-Intensity LSW delivery is impacted by individual circumstances experienced by adolescents living within different placement contexts (e.g., foster, residential, kinship), as well as what therapeutic outcomes are important for adolescents, caring adults, and social care more broadly before any future evaluations may be undertaken. Future primary research is therefore needed: (i) to further consolidate Adolescent-Focused Low-Intensity LSW core components (elements that cannot be changed) and its adaptable periphery (elements that can be changed) to enable transferability, accessibility, and implementation, (ii) to identify important and relevant outcomes for adolescents in care and those who support them (iii) use this knowledge to improve the eight initial guidelines offered by this study concisely summarised in Supplementary File 3 and create actionable recommendations to improve the quality and consistency of Adolescent-Focused Low-Intensity LSW; (iv) use this knowledge to inform the design of a comprehensive, appropriately powered evaluation, including economic and process elements of Adolescent-Focused Low-Intensity LSW which would begin to address the current scant evidence-base. When considering Adolescent-Focused Low-Intensity LSW as an intervention, the evaluation of outcomes may be challenging. As a result, evaluations measuring the impact of Adolescent-Focused Low-Intensity LSW may need to focus on intermediate outcomes, for example relationship with caring adult and longer-term outcomes, such as self-esteem, identity coherence and mental health outcomes more broadly over an extended follow up time.

## Conclusion

To our knowledge, this is the first realist review exploring how, why, to what extent and for whom Adolescent-Focused Low-Intensity LSW may work (or not) for adolescents in care. Our programme theory and initial guidelines provide important ways to improve the nature of good practice when delivering Adolescent-Focused Low-Intensity LSW to adolescents in care and produce benefits for them, their carers and health/social-care professionals.

However, to make this approach accessible to all adolescents in care a programme of research is needed to clarify how contexts impact on outcomes to enable greater transferability and more successful implementation. Additionally, to improve the current low quality evidence



For peer review only - http://bmjopen.bmj.com/site/about/guidelines.xhtml

base, a fully powered evaluation with embedded economic and process evaluations and long-term follow-up is required to generate knowledge regarding the therapeutic potential of this highly valued yet inconsistently implemented mental health intervention. Importantly, if not done properly Adolescent-Focused Low-Intensity LSW , like other interventions could do harm [125]. Hence, any future evaluation is not just about optimising delivery but also minimising harm. Given that research consistently demonstrates that adolescents in care are amongst the most vulnerable in society, this difficult but not impossible challenge needs urgent action.

## Contributorship statement

SH: Conceptualization, Methodology, Writing- Original draft preparation, Writing - Review & Editing, Supervision, Funding acquisition, Project administration. EM: Original draft preparation, Conceptualization, Methodology, Funding acquisition, Writing - Review & Editing, Project administration, Visualization, Project administration, Investigation. CD: Funding acquisition, Conceptualization, Formal analysis, data curation, Original draft preparation, writing - Review & Editing, Visualization, Investigation. RH: Funding acquisition, Conceptualization, Writing - Review & Editing. EN: Funding acquisition, Conceptualization, Writing - Review & Editing. RB: Methodology, Conceptualization, Writing - Review & Editing, Resources. KW: Funding acquisition, Conceptualization, Writing - Review & Editing, Resources. JW: Funding acquisition, Conceptualization, Writing - Review & Editing. GW: Funding acquisition, Conceptualization, Methodology, Original draft preparation, Writing - Review & Editing, Supervision.

## Competing interests

SH offers consultancy services in social media and Digital Life Story Work via www.digitallifestorywork.co.uk. KW is the CEO of The Fostering Network. The remaining authors declare that they have no known competing financial interests or personal relationships that could have appeared to influence the work reported in this paper.

## Funding

This project is funded by the National Institute for Health Research (NIHR) Research for Patient Benefit Programme (Grant Reference Number NIHR 201963).

## Data sharing statement

All data relevant to the study are included in the article or uploaded as supplementary information.

## Ethical approval

Ethical approval was not required as this is secondary research.



For peer review only - http://bmjopen.bmj.com/site/about/guidelines.xhtml

# References


1. Gov.UK. *Children looked after in England including adoptions.* 2022; Available from: https://explore-education-statistics.service.gov.uk/find-statistics/children-looked-after-in-england-including-adoptions/2022.
2. Gov.Scot. *Children's Social Work Statistics, Scotland 2020-21*. 2022; Available from: https://www.gov.scot/publications/childrens-social-work-statistics-scotland-2020-21/pages/4/.
3. Gov.Wales. *Children looked after by local authorities: April 2021 to March 2022.* . 2022; Available from: https://gov.wales/children-looked-after-local-authorities-april-2021-march-2022-html#section-109870.
4. NI.Gov. *Looked after children, adoption and fostering statistics for Northern Ireland.* . 2022; Available from: https://www.health-ni.gov.uk/publications/childrens-social-care-statistics-northern-ireland-202021.
5. Meltzer, H., Gatward, R., Corbin, T., Goodman, R., Ford, T., *The Mental Health of Young People Looked After by Local Authorities in England* 2003, London: TSO.
6. Tarren-Sweeney, M., *The mental health of children in out-of-home care.* Current Opinion in Psychiatry, 2008. **21**(4): p. 345-349.
7. NICE. *Looked-after children and young people*. NICE guideline [NG205] 2021 [cited 2023 17.01.2023]; Available from: https://www.nice.org.uk/guidance/ng205/chapter/recommendations#life-story-work-for-identity-and-wellbeing.
8. Taylor, S., et al., *Evaluation of Creative Life Story Work*. 2022. p. 112.
9. Hammond, S.P., et al., *Improving the mental health and mental health support available to adolescents with social care-experience via low-intensity life story work: a realist review protocol.* BMJ Open, 2022. **12**(3): p. e058424.
10. Ryan, T. and R. Walker, *Life Story Work*. 2016, London: CoramBAAF.
11. Herwig, K., *What Does Current Literature Tell Us About the Mental Health of Looked After Children?* BJPsych Open, 2022. **8**(S1): p. S54-S54.
12. Kontomichalos-Eyre, S.A., A.J. Lake, and J.A. McGillivray, *Life story work for children and youth in out of home care: A systematic review and synthesis of qualitative studies.* Children and Youth Services Review, 2023. **144**: p. 106697.
13. Prince, D.M., et al., *Reciprocal Effects of Positive Future Expectations, Threats to Safety, and Risk Behavior Across Adolescence.* Journal of Clinical Child & Adolescent Psychology, 2019. **48**(1): p. 54-67.
14. NICE. *Common mental health problems: identification and pathways to care: Clinical guideline [CG123]* 2011; Available from: https://www.nice.org.uk/guidance/cg123.
15. Kazdin, A.E.a.R., S.M., *Novel Models for Delivering Mental Health Services and Reducing the Burdens of Mental Illness.* Clinical Psychological Science, 2013. **1**(2): p. 170-191.
16. Hammond, S.P., J. Young, and C. Duddy, *Life story work for children and young people with care experience: A scoping review.* Developmental Child Welfare, 2020. **2**(4): p. 293-315.
17. Askelund, A.S., S. Goodyer, IM. van Harmelen, AL., *Positive memory specificity is associated with reduced vulnerability to depression.* Nat Hum Behav, 2019. **3**(3): p. 265-273.
18. Baynes, P., *Untold Stories: A Discussion of Life Story Work.* Adoption & Fostering, 2008. **32**(2): p. 43-49.
19. Cook-Cottone, C. and M. Beck, *A Model for Life-Story Work: Facilitating the Construction of Personal Narrative for Foster Children.* Child and Adolescent Mental Health, 2007. **12**(4): p. 193-195.
20. Willis, R. and S. Holland, *Life Story Work: Reflections on the Experience by Looked after Young People.* Adoption & Fostering, 2009. **33**(4): p. 44-52.


For peer review only

21. Watson, D., S. Latter, and R. Bellew, *Adopters' views on their children's life story books.* Adoption & Fostering, 2015. **39**(2): p. 119-134.
22. Watson, D.L., S. Latter, and R. Bellew, *Adopted children and young people's views on their life storybooks: The role of narrative in the formation of identities.* Children and Youth Services Review, 2015. **58**: p. 90-98.
23. Staines, J.a.J.S., *"I wish someone would explain why I am in care": The impact of children and young people's lack of understanding of why they are in out-of-home care on their well-being and felt security. .* Child & Family Social Work, 2020.
24. Neil, E., *Making sense of adoption: Integration and differentiation from the perspective of adopted children in middle childhood.* Children and Youth Services Review, 2012. **34**(2): p. 409-416.
25. Selwyn, J. and S. Riley, *Children and Young People's Views om Being in Care*. 2015, London:UK.
26. Gallagher, B. and A. Green, *In, out and after care: Young adults' views on their lives, as children, in a therapeutic residential establishment.* Children and Youth Services Review, 2012. **34**(2): p. 437-450.
27. Hooley, K., L. Stokes, and H. Combes, *Life story work with looked after and adopted children: how professional training and experience determine perceptions of its value.* Adoption & Fostering, 2016. **40**(3): p. 219-233.
28. Luke, N., et al., *What works in preventing and treating poor mental health in looked after children?* 2014, Rees Centre: London, UK.
29. Pfeifer, J.H. and E.T. Berkman, *The Development of Self and Identity in Adolescence: Neural Evidence and Implications for a Value-Based Choice Perspective on Motivated Behavior.* Child Development Perspectives, 2018. **12**(3): p. 158-164.
30. Duddy, C.W., G., *Grand rounds in methodology: when are realist reviews useful, and what does a 'good' realist review look like?* BMJ Quality & Safety . 2023. **32**: p. 173-180.
31. Pawson, R.T., N., *Realist evaluation.* 2004.
32. Pawson, R., Greenhalgh, T., Harvey, G, Walshe, K., *Realist review--a new method of systematic review designed for complex policy interventions.* Journal Health Services Research Policy, 2005. **10**(1): p. 21-34.
33. Wong, G., Greenhalgh, T., Westhorp, G., & Pawson, R., *Development of methodological guidance, publication standards and training materials for realist and meta-narrative reviews: the RAMESES (Realist And Meta-narrative Evidence Syntheses - Evolving Standards) project. .* Health Services Delivery Research, 2014. **2**(30).
34. Buchanan, A., *The experience of Life Story Work: Reflections of Young People Leaving Care*. 2014, Cardiff University: Cardiff, UK. p. 181.
35. Hammond, S.P., *Exploring a role for digital technologies in life story work with adolescents in residential care a discourse analysis* in *School of Social Work and Psychology*. 2012, University of East Anglia: Norwich.
36. HCC. *Direct Work and Life Story Work with Children and Young People. Hertfordshire County Council*. 2022 [cited 2022 12.08.22]; Available from: https://hertschildcare.proceduresonline.com/p_direct_life_story.html.
37. Holody, R., & Mäher, S., *Using lifebooks with children in family foster care a here and now process model.* Child Welfare,, 1996. **75**(4): p. 321-335.
38. Shotton, G., *'Remember when...': exploring the experiences of looked after children and their carers in engaging in collaborative reminiscence.* Adoption & Fostering, 2013. **37**(4): p. 352-367.
39. Watson, D., R. Hahn, and J. Staines, *Storying special objects: Material culture, narrative identity and life story work for children in care.* Qualitative Social Work: Research and Practice, 2020. **19**(4): p. 701-718.



For peer review only - http://bmjopen.bmj.com/site/about/guidelines.xhtml

40. Watson, D., C. Meineck, and B. Lancaster, *Adopted children's co-production and use of 'trove' (a digitally enhanced memory box) to better understand their care histories through precious objects.* Clinical child psychology and psychiatry, 2018. **23**(4): p. 614-628.
41. Atwool, N., *Life story work: Optional extra or fundamental entitlement?* Child Care in Practice, 2017. **23**(1): p. 64-76.
42. Hoyle, V., Shepherd, E., Lomas, E., & Flinn, A. , *Recordkeeping and the life-long memory and identity needs of care-experienced children and young people.* Child & Family Social Work, 2020. **25**(4): p. 935-945.
43. Together for Children Sunderland, *Foster Carers Handbook April 2019*. 2019, Sunderland City Council,.
44. Sunderland, T.f.C. *Foster Carers Handbook*. 2021; Available from: https://www.togetherforchildren.org.uk/media/25536/Foster-Carer-Handbook/pdf/Foster_Carer_Handbook.pdf?m=637983250338700000.
45. Hammond, S. and N. Cooper, *Digital Life Story Work: Using technology to help young people make sense of their experience.* 1 ed. 2013, London: British Association of Adoption and Fostering.
46. Hammond, S.P., *Digital Life Story Work in Action*, in *Life story work Why, what, how and when*, R. Walker and T. Ryan, Editors. 2016, CoramBAAF Adoption and Fostering Academy.: UK. p. 106-119.
47. Hammond, S.P., Cooper, N. J., & Jordan, P., *Mental health, identity and informal education opportunities for adolescents with experience of living in state care: a role for digital storytelling.* Cambridge Journal of Education, 2021. **51**(6): p. 713-732.
48. Peake, A., *Life Story Work. A resource for foster carers, residential social workers, adoptive parents, and kinship carers, to support this work*. 2009, Oxfordshire Foster Carers/Adopters.
49. Shotton, G., *"Remember when...": exploring the experiences of looked after children and their carers in engaging in collaborative reminiscence*. 2012, University of Newcastle upon Tyne.
50. Gustavsson, N., & MacEachron, A., *Creating Foster Care Youth Biographies: A Role for the Internet.* Journal of Technology in Human Services, 2008. **26**(1): p. 45-55.
51. Lucas, J., et al., *Therapeutic Life Story Work Barwon Pilot Program: Interim Report 2020*. 2020, School of Health and Social Development, Deakin University: Geelong, Australia.
52. Ferrier, M., *Life story work in the context of attachment-led care planning.* Scottish Journal of Residential Child Care, 2011. **10**(1).
53. Hooley, K.C., *Identifying Perspectives on Life Story Work with Looked-after and Adopted Children.*, in *Clincial Psychology*. 2015, Staffordshire University: Staffordshire.
54. Watts, R., *Relationships, reviews and recording: Developing practice for children in care.* Practice: Social Work in Action, 2021. **33**(3): p. 207-222.
55. Hills, L., *Ensuring children's life stories are at the centre of direct work.* , J. Wilkinson, Editor. 2022, Research in practice.
56. Council, B., *Life Story Work. 5 Minute Facts.*
57. Neil, E., & Beek, B., *Resepecting Children's Relationships and Identities in Adoption* in *The Routledge Handbook of Adoption*. 2020, Routledge: UK. p. 77-89.
58. Brookfield, H., Brown, S. D., & Reavey, P., *Vicarious and post-memory practices in adopting families: The re-production of the past through photography and narrative.* Journal of Community & Applied Social Psychology, 2008. **18**(5): p. 474-491.
59. Watson, D.L., E. Staples, and K. Riches, *'We need to understand what's going on because it's our life': Using sandboxing to understand children and young people's everyday conversations about care.* Children & Society, 2020. **35**(5): p. 663-679.
60. Braiden, S., *Evaluation of a short training programme for foster carers.* Scottish Journal of Residential Child Care, 2016. **15**(2).
61. Malik, S., *Application of attachment theory for training foster parents: A model program*, in *The California School of Professional Psychology*. 2005, San Francisco Bay Campus: US.




For peer review only - http://bmjopen.bmj.com/site/about/guidelines.xhtml

62. Hamilton, E., *Life story approaches and relationships within residential child care: A practice reflection.* Relational Child and Youth Care Practice, 2020. **33**(1): p. 1-11.
63. Eldridge, J., *Confiding in Others: A Qualitative Study Exploring the Experiences of Young people who have been in the Care System*. 2018, University of Surrey Guildford: UK.
64. Aventin, A., Houston, S., & Macdonald, G., *Utilising a computer game as a therapeutic intervention for youth in residential care: Some preliminary findings on use and acceptability.* Children and Youth Services Review, 2014. **47**: p. 362-369.
65. Gutsche, K., *Perceptions of social workers regarding life story work with children in child and youth care centres*. 2013, North-West University.
66. Haight, W., Black, J., & Sheridan, K., *A Mental Health Intervention for Rural, Foster Children from Methamphetamine-involved Families: Experimental Assessment with Qualitative Elaboration.* Child Youth Serv Rev, 2010. **32**(10): p. 1146-1147.
67. Agencies, T.C.o.V.A., *CVAA submission to the Review's Call for Ideas*. 2021.
68. Finlay, J., *Meaningful Natural Mentoring Relationship Characteristics and informal therapeutic life spcae interactions in youth care*, in *Salomons Institute of Applied Psychology*. 2022, Canterbury Christ Church University.
69. Shotton, G., *Telling different stories The experience of foster/adoptive carers in carrying out collaborative memory work with children.* Adoption & Fostering, 2010. **34**(4): p. 61-68.
70. Hughes, J.I., *Life-story work: a journey into a child's world.* Seen and Heard, 2013. **23**(1): p. 23-30.
71. BASW England, *Recording in Children's Social Work. 10 Top Tips.* 2020.
72. Wood, M., & Selwyn, J., *Looked after children and young people's views on what matters to their subjective well-being.* Adoption & Fostering, 2017. **41**(1): p. 20-34.
73. Furnivall, J. and E. Grant, *Trauma sensitive practice with children in care*, in *Insights: evidence summaries to support social services in Scotland*. 2014, Iriss.
74. Davies, M., & Hodges, J., *Relationship renaissance: the use of attachment-based narrative and metaphor in life story work* Adoption & Fostering, 2017. **41**(2): p. 131-141.
75. Hanna, M.D., *Preparing school age children for adoption: perspectives of successful adoptive parents and caseworkers.* Adoption Quarterly, 2007. **10**(2): p. 1-32.
76. Ward, A., *Opportunity led work maximising the possibilities for therapeutic communication in everyday interactions.* Therapeutic Communities, 2002. **23**(2): p. 111-124.
77. Connor, T., Sclare, I., Dunbar, D., & Elliffe, J., *Making a life story book.* Adoption & Fostering, 1985. **9**(2): p. 32-46.
78. Känkänen, P. and M. Bardy, *Life stories and arts in child welfare: enriching communication.* Nordic Social Work Research, 2014. **4**(1): p. 37-51.
79. Fitzhardinge, H., *Adoption, Resilience and the Importance of Stories: The Making of a Film about Teenage Adoptees* Adoption & Fostering, 2008. **32**(1): p. 58-68.
80. Steenbakkers, A., van der Steen, S., & Grietens, H., *'To talk or not to talk?': Foster youth's experiences of sharing stories about their past and being in foster care.* Children and Youth Services Review, 2016. **71**: p. 2-9.
81. NSPCC. *Life Story Work*. Available from: https://learning.nspcc.org.uk/services-children-families/life-story-work/.
82. Furnivall, J., & Grant, E. *Trauma sensitive practice with children in care (Insights: evidence summaries to support social services in Scotland*. 2014; Available from: https://www.iriss.org.uk/sites/default/files/iriss-insight-27.pdf.
83. Pakrosnis, R., & Cˇepukiene, V. (2011). Outcomes of Solution-Focused Brief Therapy for Adolescents in Foster Care and Health Care Settings, *Solution-Focused Brief Therapy: A Handbook of Evidence-Based Practice*, T.S.T. C. Franklin, E. E. McCollum, & W. J. Gingerich (Eds.), Editor. 2011: Oxford Scholarship Online.



For peer review only - http://bmjopen.bmj.com/site/about/guidelines.xhtml

84. Happer, H., McCreadie, J., & Aldgate, J. *Celebrating Success: What Helps Looked After Children Succeed*. 2006; Available from: https://www.wecanandmustdobetter.org/files/8314/2781/0566/Celebrating_success.pdf.
85. Aust, P.H., *Using the Life Story Book in Treatment of Children in Placement.* Child Welfare, 1981. **60**(8): p. 535-560.
86. Teodorczuk, K., Guse, T., & du Plessis, G. A., *The effect of positive psychology interventions on hope and well-being of adolescents living in a child and youth care centre.* British Journal of Guidance & Counselling, 2018. **47**(2): p. 234-245.
87. Sanders, R. *ESSS Outline: Care experienced children and young people's mental health*. 2020; Available from: https://doi.org/10.31583/esss.20201012.
88. Devenney, K., *Pathway planning with unaccompanied young people leaving care: Biographical narratives of past, present, and future.* Child & Family Social Work, 2017. **22**(3): p. 1313-1321.
89. Bazalgette, L., T. Rahilly, and G. Trevelyan, *Achieving emotional wellbeing for looked after children*. 2015, NSPCC.
90. Beste, H.M., & Richardson, R. G., *Developing a Life Story Book Program for Foster Children.* Child Welfare, 1981. **60**(8): p. 529-534.
91. ARCBOX. *Digital Life Story ARCBOX ARC Adoption North East*. 2022; Available from: https://www.arcadoptionne.org.uk/life-story.
92. Monson, K., Moeller-Saxone, K., Humphreys, C., Harvey, C., & Herrman, H., *Promoting mental health in out of home care in Australia.* Health Promot Int, 2020. **35**(5): p. 1026-1036.
93. Watson, D., R. Hahn, and J. Staines, *Storying special objects: Material culture, narrative identity and life story work for children in care.* Qualitative Social Work, 2019. **19**(4): p. 701-718.
94. Observatory, N.F.J. *Modernising post-adoption contact: findings from a recent consultation*. 2021; Available from: https://www.nuffieldfjo.org.uk/wp-content/uploads/2021/08/nfjo_report_adoption_connections_20210913v2.pdf.
95. Atwell, A., *Working with children with a disability*, in *Life Story Work why, what, how and when*, R.W.T. Ryan, Editor. 2016, CoramBAAF Adoption and Fostering Academy. : UK. p. 97-102.
96. Walker, R. and T. Ryan, *Why do life story work?*, in *Life story work Why, what, how and when* R. Ryan and R. Walker, Editors. 2016, CoramBAAF Adoption and Fostering Academy: UK. p. 4-17.
97. Gray, S., et al., *Trove: A Digitally Enhanced Memory Box for Looked after and Adopted Children*, in *Proceedings of the 18th ACM International Conference on Interaction Design and Children*. 2019. p. 458-463.
98. Shepherd, E. *Good practice in record-keeping in children's social care*. 2022; Available from: https://www.researchinpractice.org.uk/children/news-views/2022/february/good-practice-in-record-keeping-in-children-s-social-care/.
99. Nicholls, E., *Model answer.* Community Care, 2003. **1479**: p. 32-34.
100. Humphreys, C., & Kertesz, M., *Making Records Meaningful: Creating an Identity Resource for Young People in Care.* Australian Social Work, 2014. **68**(4): p. 497-514.
101. Eenshuistra, A., Harder, A. T., & Knorth, E. J., *One size does not fit all: A systematic review of training outcomes on residential youth care professionals' skills.* Children and Youth Services Review, 2019. **103**: p. 135-147.
102. Steinberg, L., Monahan, K. C. , *Age differences in resistance to peer influence.* Developmental Psychology, 2007. **43**(6): p. 1531-1543.
103. Evans, R., et al., *Interventions targeting the mental health and wellbeing of care-experienced children and young people in higher-income countries: Evidence map and systematic review.* Systematic Reviews, 2023. **12**(1): p. 111.



For peer review only - http://bmjopen.bmj.com/site/about/guidelines.xhtml

104. O'Higgins, A., J. Sebba, and F. Gardner, *What are the factors associated with educational achievement for children in kinship or foster care: A systematic review.* Children and Youth Services Review, 2017. **79**: p. 198-220.
105. Travagin, G., D. Margola, and T.A. Revenson, *How effective are expressive writing interventions for adolescents? A meta-analytic review.* Clin Psychol Rev, 2015. **36**: p. 42-55.
106. Alpert, E., et al., *A systematic review of literature examining mediators and mechanisms of change in empirically supported treatments for posttraumatic stress disorder.* Clinical Psychology Review, 2023. **103**: p. 102300.
107. McAdams, D.P. and K.C. McLean, *Narrative Identity.* Current Directions in Psychological Science, 2013. **22**(3): p. 233-238.
108. Seligman, M.E.P., *Flourish: A visionary new understanding of happiness and well-being.* Flourish: A visionary new understanding of happiness and well-being. 2011, New York, NY, US: Free Press. xii, 349-xii, 349.
109. Boddy, J., *Understanding permanence for looked after children: a review of research for the Care Inquiry*. 2013, University of Sussex.
110. McKeown, J., A. Clarke, and J. Repper, *Life story work in health and social care: systematic literature review.* Journal of Advanced Nursing, 2006. **55**: p. 237-247.
111. CEBM. *Oxford Centre for Evidence-based Medicine - Levels of Evidence (March 2009)*. 2009 [cited 2023 11.01.23]; Available from: https://www.cebm.net/2009/06/oxford-centre-evidence-based-medicine-levels-evidence-march-2009/.
112. Evans, D., *Hierarchy of evidence: a framework for ranking evidence evaluating healthcare interventions.* Journal of Clinical Nursing, 2003. **12**(1): p. 77-84.
113. Education, D.o.H.D.f. *Transforming children and young people's mental health provision: Green paper*. 2017 [cited 2023 11.01.23]; Available from: https://www.gov.uk/government/consultations/transforming-children-and-young-peoples-mental-health-provision-a-green-paper.
114. Committee, H.o.C.E., *Mental health and well-being of looked-after children*. 2016, House of Commons: London, UK.
115. NICE. *Child abuse and neglect: NICE guideline [NG76]*. 2017 [cited 2023 11.01.23]; Available from: https://www.nice.org.uk/guidance/ng76.
116. Pawson, R. and A. Manzano-Santaella, *A realist diagnostic workshop.* Evaluation, 2012. **18**(2): p. 176-191.
117. Saluja, S.S., A., Mukhopadhyay, S. Lin, Y. Raykar, N. Keshavjee, S. Samad, L. Meara, J. G. , *Using the Consolidated Framework for Implementation Research to implement and evaluate national surgical planning.* BMJ Glob Health, 2017. **2**(2): p. e000269.
118. *Adoption and Children Act 2002*.
119. *Children Act 1989*.
120. Education, D.f., *Guide to the Children's Homes Regulations including the quailty standards*. 2015, D.f. Education: London, UK.
121. Regulations, T.F.S.E., *The Fostering Services (England) Regulations 2011*. 2011.
122. Hammond, S.P. and N. Cooper, *Digital life story work: Using technology to help young people make sense of their experiences*. 2013, London: British Association of Adoption and Fostering.
123. Hoffmann, T.C., Glasziou, P.P., Boutron, I., Milne, R., Perera, R., Moher, D., Altman, D.G., Barbour, V., Macdonald, H., Johnston, M., Lamb, S.E., Dixon-Woods, M., McCulloch P., Wyatt, J.C., Chan, A-W., Michie, S., *Better reporting of interventions: template for intervention description and replication (TIDieR) checklist and guide.* BMJ, 2014. **348**: p. G1687.
124. Pinnock, H., Barwick, M., Carpenter, C.R., Eldridge, S., Grandes, G., Griffiths, C.J., Rycroft-Malone, J., Meissner, P., Murray, E., Patel A., Sheikh, A., Taylor, S.J.C., *Standards for Reporting Implementation Studies (StaRI) Statement.* BMJ, 2017. **356**: p. i6795.
125. Lilienfield, S.O., *Psychological Treatments That Cause Harm.* Perspectives on Psychological Science, 2007. **2**(1): p. 53-70.



For peer review only - http://bmjopen.bmj.com/site/about/guidelines.xhtml

For peer review only



For peer review only - http://bmjopen.bmj.com/site/about/guidelines.xhtml

Figure Legend:

Figure 1: PRISMA flow diagram providing summary of searching and selection processes

Figure Legend:

Figure 2: Initial programme theory for Adolescent-Focused Low-Intensity Life Story Work

Figure Legend:

Figure 3: Consolidated programme theory for Adolescent-Focused Low-Intensity Life Story Work

For peer review only



For peer review only - http://bmjopen.bmj.com/site/about/guidelines.xhtml

# Table 1: Summary of methods

| Step | Aim | Approach |
|---|---|---|
| Step 1: Develop an initial programme theory | To identify existing relevant theories that provide explanations of why and how Adolescent-Focused Low-Intensity Life Story Work (AF-LI-LSW) approaches work (or are thought to work), in what contexts they work, to what extent and for whom. | Exploratory searches (CD)<br><br>Project team knowledge and consultation with stakeholders (All) |
| Step 2: Evidence search | To perform a comprehensive literature search to identify data to develop and refine the initial programme theory . | Electronic database searches across multiple databases (see supplementary file 1)<br><br>Targeted Google searches to retrieve grey literature produced by local authorities (CD)<br><br>Citation tracking (EM)<br><br>Project team knowledge (All)<br><br>Email alert to capture new literature (CD) |
| Step 3: Document selection, data organisation and extraction | To select eligible documents that could include relevant data to contribute to the development and refinement of the programme theory. To describe selected documents and to extract and code relevant data. The full set of extracted data are available on request. | Screen title and abstract and then full text against the inclusion and exclusion criteria (See Table 2: Inclusion and exclusion criteria (EM & SH)<br><br>Included documents selected based on the rigour and relevance of documents.<br><br>10% random sample of documents screened in duplicate with discrepancies resolved through group discussion (EM & SH)<br><br>Key characteristics of selected documents were extracted into Excel (EM)<br><br>Full texts uploaded to NVivo and relevant data to the research question and review focus were coded (EM) |



For peer review only - http://bmjopen.bmj.com/site/about/guidelines.xhtml

| | | |
|---|---|---|
| Step 4: Evidence synthesis | To apply a realist logic of analysis to extracted data to develop Context, Mechanism, Outcome Configurations (CMOCs) about how AF-LI-LSW improves the mental health support available to adolescents looked-after by social care. CMOCs are central to realist analysis, offering an explanation of the relationships between the conditions an intervention is delivered, the mechanisms by which the intervention works and the outcomes that are produced when delivered within those particular contexts. | The coded data was closely inspected, and interpretations were made about whether evidence was functioning as a context, mechanism, or outcome and which CMOC the evidence belonged to (EM & SH)<br><br>Cross document comparisons were made to build CMOCs, as documents did not always contain evidence that supported the development of CMOCs as evidence for each element of the configuration could not always be found in one document (EM & SH)<br><br>As the CMOCs were created and refined, judgements were made on how they related to each other in consultation with the project team and where relevant stakeholders (All) |
| Step 5: Development of consolidated programme theory and drawing conclusions | To develop a consolidated programme theory containing the CMOCs | The programme theory was refined iteratively through the development of CMOCs and consultation with stakeholders and project team discussions (All)<br><br>Judgements were made on the plausibility and coherence of the developing programme theory (All)<br><br>The consolidated programme theory was written and shared with stakeholders and final amendments were made based on their consultation (All) |



For peer review only - http://bmjopen.bmj.com/site/about/guidelines.xhtml

# Table 2: Eligibility criteria

| *Inclusion criteria* | *Exclusion criteria* |
|---|---|
| Population: Young people who are under the care of a local authority, young people who are 'looked after' or care experienced or adopted young people, or their parents/carers. | Research focused solely on parenting style, communicative openness in foster or adoptive families, contact with birth family members |
| Intervention: LSW, including all activities involving recording, exploring, eliciting accounts of a care experienced person's life or personal history, to have an impact on their understanding of themselves and their identify | |
| And/or | |
| Low-intensity interventions that aim to address a mental health or wellbeing need | |
| Document type/study design: any | |
| Other: English language only | |

For peer review only

Improving the mental health and mental health support available to adolescents in out-of-home care via Adolescent-Focused Low-Intensity Life Story Work: A realist review

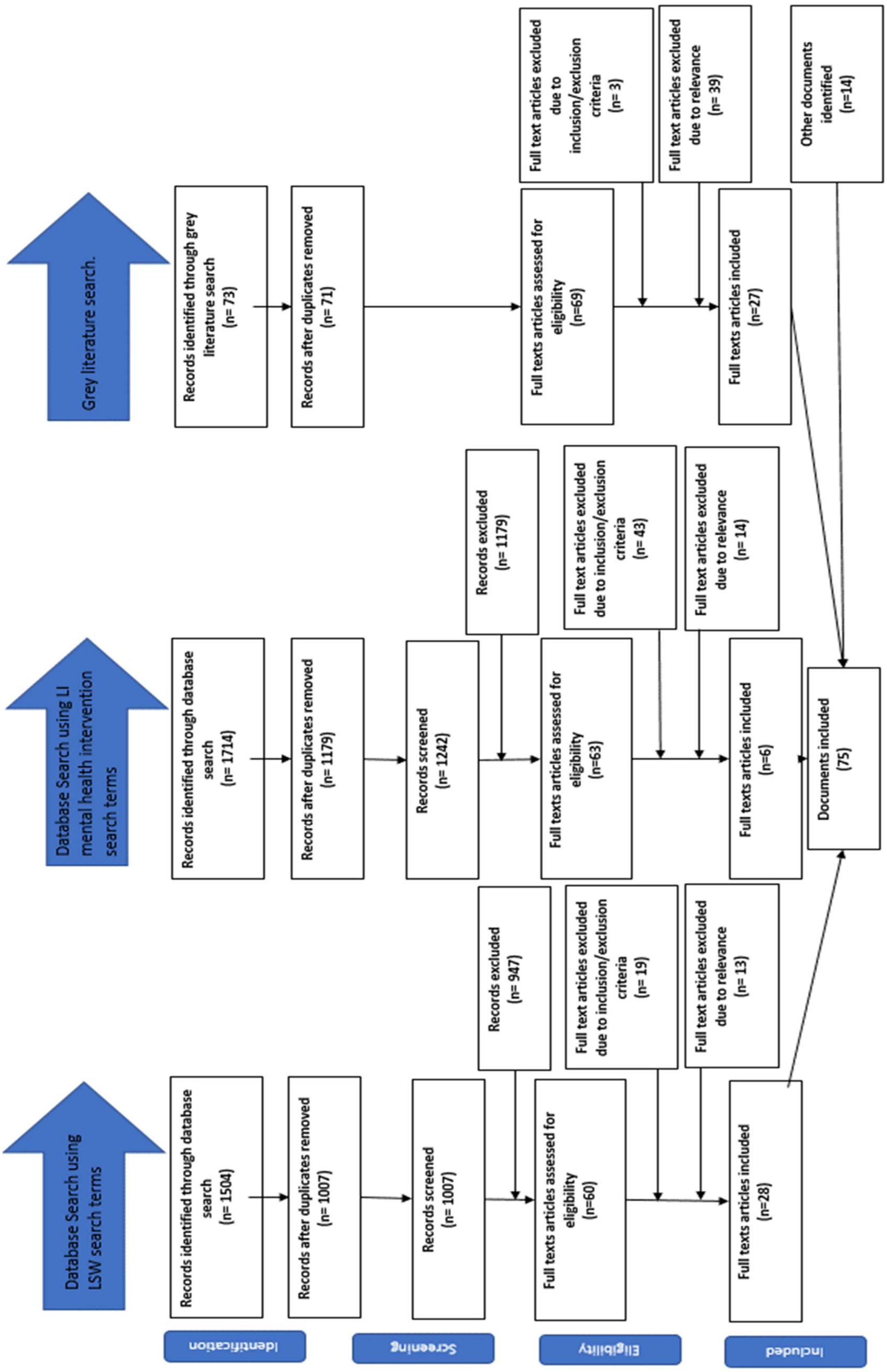

Improving the mental health and mental health support available to adolescents in out-of-home care adolescents looked-after by social care via Adolescent-Focused Low-Intensity Life Story Work: A realist review

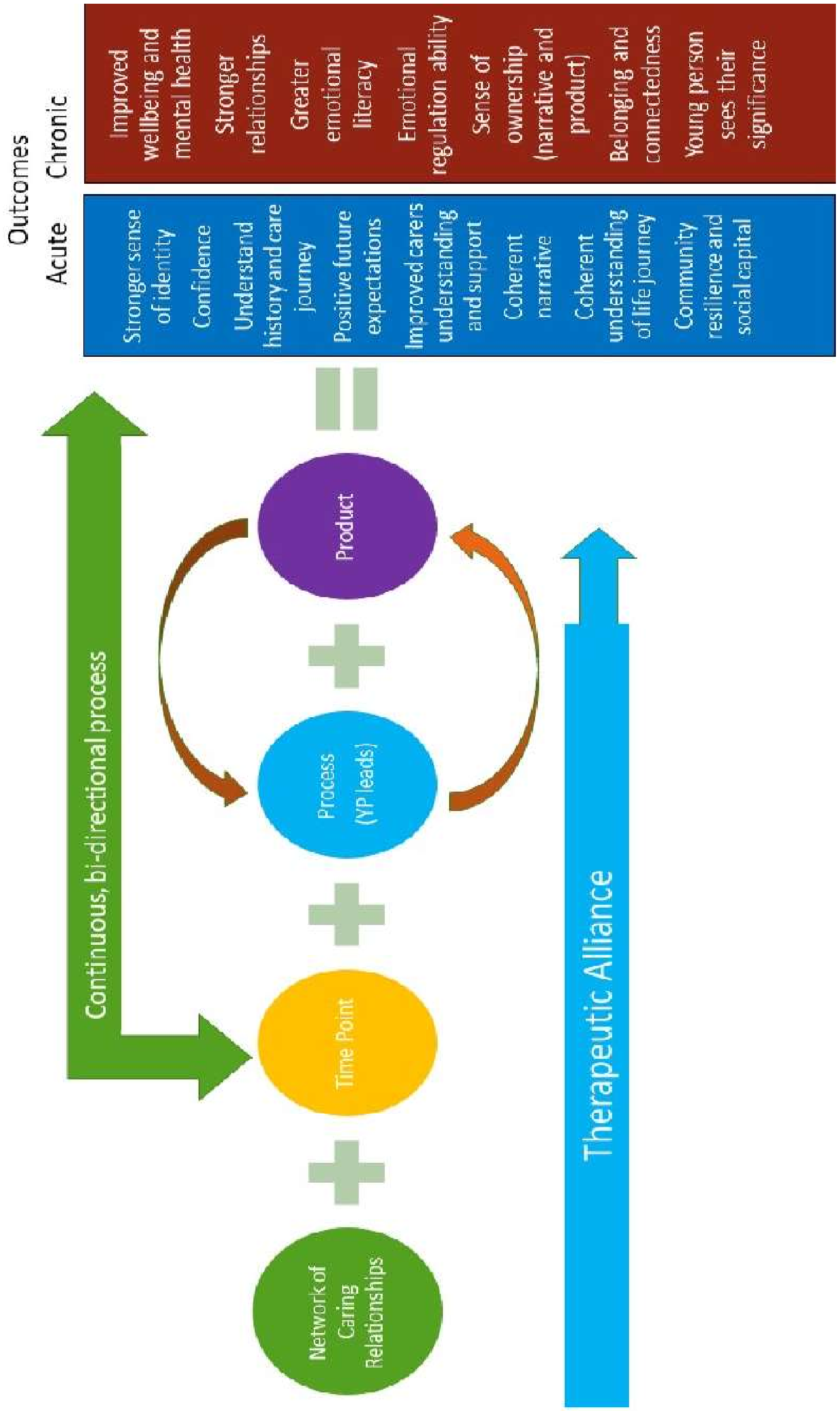

Improving the mental health and mental health support available to adolescents in out-of-home care via Adolescent-Focused Low-Intensity Life Story Work: A realist review

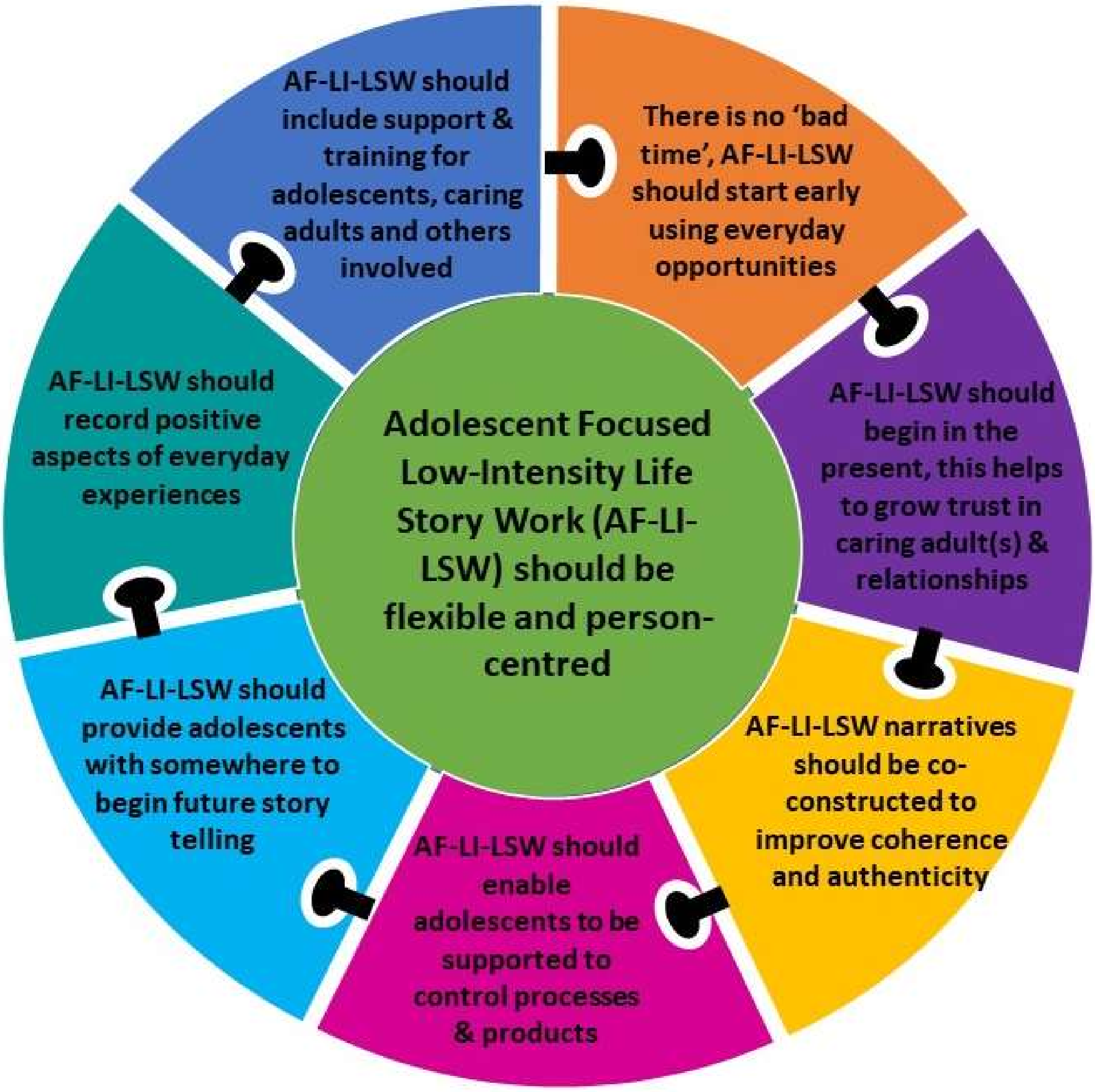

Improving the mental health and mental health support available to adolescents in out-of-home care via Adolescent-Focused Low-Intensity Life Story Work: A realist review

# Supplementary File 1

## Search #1 Life Story Work

### MEDLINE

**Medline (Ovid MEDLINE® Epub Ahead of Print, In-Process & Other Non-Indexed Citations, Ovid MEDLINE® Daily and Ovid MEDLINE®) 1946 to present**

Search run on 1 December 2021

| | Searches | Results | Type |
|---|---|---|---|
| **1** | ((life story or life history) adj2 (work or book* or resource* or tool*)).ti,ab,kw. | 107 | |
| **2** | (personal history or biograph* or autobiograph*).ti,ab,kw. | 30152 | |
| **3** | 1 or 2 | 30262 | |
| **4** | (teen* or youth* or adolescen* or juvenile* or young* or child* or girl? or boy?).ti,ab,kw. | 2435378 | |
| **5** | Adolescent/ | 2143136 | |
| **6** | 4 or 5 | 3811906 | |
| **7** | looked-after.ti,ab,kw. | 569 | |
| **8** | in care.ti,ab,kw. | 18400 | |
| **9** | ((foster* or social or public or state or local authority or residential or institutional or permanent or kinship or relative or substitute or out-of-home or shelter or surrogate) adj1 care*).ti,ab,kw. | 19263 | |
| **10** | (care leaver* or care-experience* or leaving care or care transition*).ti,ab,kw. | 6579 | |
| **11** | child welfare.ti,ab,kw. | 5173 | |
| **12** | (adopted or adoption or adoptive or adoptee*).ti,ab,kw. | 197909 | |
| **13** | Adoption/ | 4869 | |
| **14** | Foster Home Care/ | 3758 | |
| **15** | or/7-14 | 246890 | |
| **16** | 3 and 6 and 15 | 87 | |



For peer review only - http://bmjopen.bmj.com/site/about/guidelines.xhtml

Improving the mental health and mental health support available to adolescents in out-of-home care via Adolescent-Focused Low-Intensity Life Story Work: A realist review

Embase

**Ovid Embase 1974 to Present**

| | Searches | Results | Type |
|---|---|---|---|
| **1** | ((life story or life history) adj2 (work or book* or resource* or tool*)).ti,ab,kw. | 148 | |
| **2** | (personal history or biograph* or autobiograph*).ti,ab,kw. | 25170 | |
| **3** | 1 or 2 | 25307 | |
| **4** | (teen* or youth* or adolescen* or juvenile* or young* or child* or girl? or boy?).ti,ab,kw. | 3050210 | |
| **5** | adolescent/ or exp institutionalized adolescent/ | 1629539 | |
| **6** | 4 or 5 | 3924528 | |
| **7** | looked-after.ti,ab,kw. | 1002 | |
| **8** | in care.ti,ab,kw. | 26487 | |
| **9** | ((foster* or social or public or state or local authority or residential or institutional or permanent or kinship or relative or substitute or out-of-home or shelter or surrogate) adj1 care*).ti,ab,kw. | 23880 | |
| **10** | (care leaver* or care-experience* or leaving care or care transition*).ti,ab,kw. | 8952 | |
| **11** | child welfare.ti,ab,kw. | 3648 | |
| **12** | (adopted or adoption or adoptive or adoptee*).ti,ab,kw. | 257330 | |
| **13** | adoption/ | 18302 | |
| **14** | foster care/ or foster child/ | 5008 | |
| **15** | or/7-14 | 320109 | |
| **16** | 3 and 6 and 15 | 116 | |

Search run on 1 December 2021


For peer review only - http://bmjopen.bmj.com/site/about/guidelines.xhtml

Improving the mental health and mental health support available to adolescents in out-of-home care via Adolescent-Focused Low-Intensity Life Story Work: A realist review

PsycINFO

**Ovid PsycINFO 1806 to Present**

Search run on 1 December 2021

| | Searches | Results | Type |
|---|---|---|---|
| **1** | ((life story or life history) adj2 (work or book* or resource* or tool*)).ti,ab. | 126 | |
| **2** | (personal history or biograph* or autobiograph*).ti,ab. | 27009 | |
| **3** | life review/ or autobiographical memory/ | 5007 | |
| **4** | or/1-3 | 28393 | |
| **5** | (teen* or youth* or adolescen* or juvenile* or young* or child* or girl? or boy?).ti,ab. | 1104726 | |
| **6** | looked-after.ti,ab. | 656 | |
| **7** | in care.ti,ab. | 7188 | |
| **8** | ((foster* or social or public or state or local authority or residential or institutional or permanent or kinship or relative or substitute or out-of-home or shelter or surrogate) adj1 care*).ti,ab. | 19519 | |
| **9** | (care leaver* or care-experience* or leaving care or care transition*).ti,ab. | 2950 | |
| **10** | child welfare.ti,ab. | 7784 | |
| **11** | (adopted or adoption or adoptive or adoptee*).ti,ab. | 66430 | |
| **12** | adoption (child)/ or adopted children/ or adoptive parents/ | 5319 | |
| **13** | foster care/ or foster children/ or foster parents/ | 7544 | |
| **14** | or/6-13 | 99727 | |
| **15** | 4 and 5 and 14 | 203 | |

For peer review only

Improving the mental health and mental health support available to adolescents in out-of-home care via Adolescent-Focused Low-Intensity Life Story Work: A realist review

Proquest Sociology Collection

**ASSIA (Applied Social Sciences Index & Abstracts) (1987 to present); Sociological Abstracts (1952 to present); Sociology Database (1985 to present)**

Search run on 1 December 2021

| | Searches | Results | Type |
|---|---|---|---|
| **1** | noft(("life story" OR "life history") N/2 (work OR book* OR resource* OR tool*)) | 223 | |
| **2** | noft(("personal history" OR biograph* OR autobiograph*)) | 30,652 | |
| **3** | 1 or 2 | 30,836 | |
| **4** | noft((teen* OR youth* OR adolescen* OR juvenile* OR young* OR child* OR boy* OR girl*)) | 776,851 | |
| **5** | noft("looked after" OR "looked-after") | 1,477 | |
| **6** | noft("in care") | 8,932 | |
| **7** | noft((foster* OR social OR public OR state OR "local authority" OR residential OR institutional OR permanent OR kinship OR relative OR substitute OR "out of home" OR out-of-home OR shelter OR surrogate) N/1 care*) | 49,937 | |
| **8** | noft("care leaver" OR "care experience*" OR care-experience* OR "leaving care" OR "care transition*") | 3,219 | |
| **9** | noft("child welfare") | 27,228 | |
| **10** | noft(adopted OR adoption OR adoptive OR adoptee*) | 35,937 | |
| **11** | 5 or 6 or 7 or 8 or 9 or 10 | 110774 | |
| **12** | 3 and 4 and 12 | 372 | |
| | | | |
| **Full** | (noft(("life story" OR "life history") NEAR/2 (work OR book* OR resource* OR tool*)) OR noft(("personal history" OR biograph* OR autobiograph*))) AND (noft("looked after" OR "looked-after") OR noft("in care") OR noft((foster* OR social OR public OR state OR "local authority" OR residential OR institutional OR permanent OR kinship OR relative OR substitute OR "out of home" OR out-of-home OR shelter OR surrogate) NEAR/1 care*) OR noft("care leaver" OR "care experience*" OR care-experience* OR "leaving care" OR "care transition*") OR noft("child welfare") OR noft(adopted OR adoption OR adoptive OR adoptee*)) AND noft((teen* OR youth* OR adolescen* OR juvenile* OR young* OR child* OR boy* OR girl*)) | | |



For peer review only - http://bmjopen.bmj.com/site/about/guidelines.xhtml

Improving the mental health and mental health support available to adolescents in out-of-home care via Adolescent-Focused Low-Intensity Life Story Work: A realist review

CINAHL

**Ebscohost CINAHL (Cumulative Index to Nursing and Allied Health Literature) (Start date unknown)**

Search run 1 December 2021

| | Searches | Results | Type |
|---|---|---|---|
| **S1** | TI ("life story" OR "life history") N2 (work OR book* OR resource* OR tool*) OR AB ("life story" OR "life history") N2 (work OR book* OR resource* OR tool*) | 135 | |
| **S2** | TI ("personal history" OR biograph* OR autobiograph*) OR AB ("personal history" OR biograph* OR autobiograph*) | 6148 | |
| **S3** | (MH "Life History Review") OR (MH "Autobiographical Memory") | 1592 | |
| **S4** | S1 OR S2 OR S3 | 7675 | |
| **S5** | TI teen* OR youth* OR adolescen* OR juvenile* OR young* OR child* OR girl* OR boy* OR AB teen* OR youth* OR adolescen* OR juvenile* OR young* OR child* OR girl* OR boy* | 1475230 | |
| **S6** | (MH "Adolescence") | 564984 | |
| **S7** | S5 OR S6 | 1475230 | |
| **S8** | TI "looked after" OR looked-after OR AB "looked after" OR looked-after | 718 | |
| **S9** | TI "in care" OR AB "in care" | 929827 | |
| **S10** | TI (foster* OR social OR public OR state OR "local authority" OR residential OR institutional OR permanent OR kinship OR relative OR substitute OR "out of home" OR out-of-home OR shelter OR surrogate) N1 care* OR AB (foster* OR social OR public OR state OR "local authority" OR residential OR institutional OR permanent OR kinship OR relative OR substitute OR "out of home" OR out-of-home OR shelter OR surrogate) N1 care* | 31046 | |
| **S11** | TI ("care leaver" OR "care experience*" OR care-experience* OR "leaving care" OR "care transition*") OR AB ("care leaver" OR "care experience*" OR care-experience* OR "leaving care" OR "care transition*") | 5417 | |
| **S12** | TI "child welfare" OR AB "child welfare" | 4202 | |
| **S13** | TI adopted or adoption or adoptive or adoptee* OR AB adopted or adoption or adoptive or adoptee* | 57750 | |
| **S14** | (MH "Adoption") OR (MH "Child, Adopted") OR (MH "Adoptive Parents") | 4085 | |
| **S15** | (MH "Foster Home Care") OR (MH "Foster Parents") OR (MH "Child, Foster") | 6372 | |
| **S16** | S8 OR S9 OR S10 OR S11 OR S12 OR S13 OR S14 OR S15 | 9 75840 | |
| **S17** | S4 AND S7 AND S16 | 359 | |



For peer review only - http://bmjopen.bmj.com/site/about/guidelines.xhtml

Improving the mental health and mental health support available to adolescents in out-of-home care via Adolescent-Focused Low-Intensity Life Story Work: A realist review

CDAS

**Ebscohost CDAS (Child Development and Adolescent Studies) (Start date unknown)**

Search run 1 December 2021

| | Searches | Results | Type |
|---|---|---|---|
| **S1** | TI ("life story" OR "life history") N2 (work OR book* OR resource* OR tool*) OR AB ("life story" OR "life history") N2 (work OR book* OR resource* OR tool*) | 29 | |
| **S2** | TI ("personal history" OR biograph* OR autobiograph*) OR AB ("personal history" OR biograph* OR autobiograph*) | 1442 | |
| **S3** | S1 OR S2 | 1471 | |
| **S4** | TI "looked after" OR looked-after OR AB "looked after" OR looked-after | 696 | |
| **S5** | TI "in care" OR AB "in care" | 32026 | |
| **S6** | TI (foster* OR social OR public OR state OR "local authority" OR residential OR institutional OR permanent OR kinship OR relative OR substitute OR "out of home" OR out-of-home OR shelter OR surrogate) N1 care* OR AB (foster* OR social OR public OR state OR "local authority" OR residential OR institutional OR permanent OR kinship OR relative OR substitute OR "out of home" OR out-of-home OR shelter OR surrogate) N1 care* | 7379 | |
| **S7** | TI ("care leaver" OR "care experience*" OR care-experience* OR "leaving care" OR "care transition*") OR AB ("care leaver" OR "care experience*" OR care-experience* OR "leaving care" OR "care transition*") | 651 | |
| **S8** | TI "child welfare" OR AB "child welfare" | 5715 | |
| **S9** | TI adopted or adoption or adoptive or adoptee* OR AB adopted or adoption or adoptive or adoptee* | 6798 | |
| **S10** | S4 OR S5 OR S6 OR S7 OR S8 OR S9 | 41463 | |
| **S11** | S3 AND S10 | 86 | |
| | | | |
| | *NB Search string describing adolescents removed due to population focus in this database* | | |



For peer review only - http://bmjopen.bmj.com/site/about/guidelines.xhtml

Improving the mental health and mental health support available to adolescents in out-of-home care via Adolescent-Focused Low-Intensity Life Story Work: A realist review

Web of Science

**Clarivate Web of Science Core Collection (SCI-EXPANDED and SSCI indexes) 1900 to present**

Search run 1 December 2021

| | Searches | Results | Type |
|---|---|---|---|
| **1** | TS=((("life story" OR "life history") NEAR/2 (work OR book* OR resource* OR tool*))) | 449 | |
| **2** | TS=((("personal history" OR biograph* OR autobiograph*))) | 41850 | |
| **3** | 1 OR 2 | 41850 | |
| **4** | TS=(((teen* OR youth* OR adolescen* OR juvenile* OR young* OR child* OR boy* OR girl*))) | 3066217 | |
| **5** | TS=(("looked after" OR "looked-after")) | 768 | |
| **6** | TS=(("in care")) | 16889 | |
| **7** | TS=((foster* OR social OR public OR state OR "local authority" OR residential OR institutional OR permanent OR kinship OR relative OR substitute OR "out of home" OR out-of-home OR shelter OR surrogate) NEAR/1 care*) | 43040 | |
| **8** | TS=("care leaver" OR "care experience*" OR care-experience* OR "leaving care" OR "care transition*") | 6615 | |
| **9** | TS=("child welfare") | 8348 | |
| **10** | TS=(adopted OR adoption OR adoptive OR adoptee*) | 655351 | |
| **11** | 5 OR 6 OR 7 OR 8 OR 9 OR 10 | 721923 | |
| **12** | 3 AND 4 AND 11 | 233 | |



For peer review only - http://bmjopen.bmj.com/site/about/guidelines.xhtml

Improving the mental health and mental health support available to adolescents in out-of-home care via Adolescent-Focused Low-Intensity Life Story Work: A realist review

## Social Care Online

**SCIE Social Care Online 1980 to present**

Search run 3 February 2022

| | Searches | Results | Type |
|---|---|---|---|
| **1** | "life story" OR "life history" | | |
| **2** | teen OR youth OR adolescent OR juvenile OR young OR child OR girl OR boy | | |
| **3** | "looked after" OR "foster care" OR "social care" OR "public care" OR "state care" OR "local authority care" OR "residential care" OR "institutional care" OR "permanent care" OR "kinship care" OR "relative care" OR "substitute care" OR "out of home care" OR "shelter care" OR adopted OR adoption OR adoptive OR adoptee | | |
| **4** | (1 AND 2 AND 3) | 58 | |
| | *NB Search strategies were adapted for simpler search interface; word variations included automatically* | | |



For peer review only - http://bmjopen.bmj.com/site/about/guidelines.xhtml

Improving the mental health and mental health support available to adolescents in out-of-home care via Adolescent-Focused Low-Intensity Life Story Work: A realist review

## Search #2 Low intensity mental health interventions

### MEDLINE

**Medline (Ovid MEDLINE® Epub Ahead of Print, In-Process & Other Non-Indexed Citations, Ovid MEDLINE® Daily and Ovid MEDLINE®) 1946 to present**

Search run on 2 February 2022

| | Searches | Results | Type |
|---|---|---|---|
| **1** | (teen* or youth* or adolescen* or juvenile*).ti,ab,kw. | 486326 | |
| **2** | Adolescent/ | 2155772 | |
| **3** | 1 or 2 | 2326981 | |
| **4** | looked-after.ti,ab,kw. | 585 | |
| **5** | ((foster* or social or public or state or local authority or residential or institutional or permanent or kinship or relative or substitute or out-of-home or shelter or surrogate) adj1 care*).ti,ab,kw. | 19561 | |
| **6** | (care leaver* or care-experience* or leaving care or care transition*).ti,ab,kw. | 6715 | |
| **7** | child welfare.ti,ab,kw. | 5217 | |
| **8** | Adoption/ | 4875 | |
| **9** | Foster Home Care/ | 3770 | |
| **10** | or/4-9 | 37119 | |
| **11** | ((adopted or adoption or adoptive or adoptee* or in-care or "in care") adj3 (teen* or youth* or adolescen* or juvenile* or young* or child* or girl? or boy?)).ti,ab,kw. | 3708 | |
| **12** | 10 or 11 | 38990 | |
| **13** | ((low-intensity or minimal or brief or online or internet or tele* or mobile* or e-health or m-health or virtual) adj2 (intervention* or program* or service* or package* or training* or therap* or treatment*)).ti,ab,kw. | 48784 | |
| **14** | Therapy, Computer-Assisted/ | 6949 | |
| **15** | Computer-Assisted Instruction/ | 12318 | |
| **16** | Telemedicine/ | 32176 | |
| **17** | or/13-16 | 92385 | |
| **18** | (lay-therapy or lay-therapist* or lay-worker* or lay-person* or lay-people).ti,ab,kw. | 2143 | |
| **19** | (para-professional* or non-specialist* or non-clinician* or health worker* or support worker*).ti,ab,kw. | 24797 | |
| **20** | ((unqualified or unregistered or volunt*) adj2 (therap* or worker* or coach* or facilitator* or practitioner*)).ti,ab,kw. | 1337 | |
| **21** | Community Health Workers/ | 6092 | |
| **22** | or/18-21 | 30823 | |
| **23** | (self-help or self-manage*).ti,ab,kw. | 31158 | |
| **24** | Self Care/ | 35033 | |
| **25** | or/23-24 | 57185 | |
| **26** | ((group* or peer*) adj2 (intervention* or program* or service* or package* or training* or therap* or treatment)).ti,ab,kw. | 228457 | |
| **27** | Self-Help Groups/ | 9435 | |



For peer review only - http://bmjopen.bmj.com/site/about/guidelines.xhtml

Improving the mental health and mental health support available to adolescents in out-of-home care via Adolescent-Focused Low-Intensity Life Story Work: A realist review

| | | |
|---|---|---|
| **28** | or/26-27 | 236909 |
| **29** | 17 or 22 or 25 or 28 | 398896 |
| **30** | (mental health or wellbeing or well-being).ti,ab,kw. | 280239 |
| **31** | mental health/ or exp mental disorders/ | 1382592 |
| **32** | Quality of Life/ | 232462 |
| **33** | or/30-32 | 1732433 |
| **34** | 3 and 12 and 29 and 33 | 160 |
| **35** | limit 34 to english language | 151 |

For peer review only

Improving the mental health and mental health support available to adolescents in out-of-home care via Adolescent-Focused Low-Intensity Life Story Work: A realist review

Embase

**Ovid Embase 1974 to Present**

Search run on 2 February 2022

| | Searches | Results | Type |
|---|---|---|---|
| **1** | (teen* or youth* or adolescen* or juvenile*).ti,ab,kw. | 601244 | |
| **2** | adolescent/ or institutionalized adolescent/ | 1644483 | |
| **3** | 1 or 2 | 1868684 | |
| **4** | looked-after.ti,ab,kw. | 1012 | |
| **5** | ((foster* or social or public or state or local authority or residential or institutional or permanent or kinship or relative or substitute or out-of-home or shelter or surrogate) adj1 care*).ti,ab,kw. | 24293 | |
| **6** | (care leaver* or care-experience* or leaving care or care transition*).ti,ab,kw. | 9146 | |
| **7** | child welfare.ti,ab,kw. | 3695 | |
| **8** | adoption/ | 18546 | |
| **9** | foster care/ or foster child/ | 5037 | |
| **10** | or/4-9 | 56946 | |
| **11** | ((adopted or adoption or adoptive or adoptee* or in-care or "in care") adj3 (teen* or youth* or adolescen* or juvenile* or young* or child* or girl? or boy?)).ti,ab,kw. | 4485 | |
| **12** | 10 or 11 | 59432 | |
| **13** | ((low-intensity or minimal or brief or online or internet or tele* or mobile* or e-health or m-health or virtual) adj2 (intervention* or program* or service* or package* or training* or therap* or treatment*)).ti,ab,kw. | 65233 | |
| **14** | computer assisted therapy/ | 4801 | |
| **15** | Computer-Assisted Instruction/ | 81963 | |
| **16** | telemedicine/ or exp teleconsultation/ or exp telemonitoring/ or exp telepsychiatry/ or exp telepsychology/ or exp teletherapy/ or exp video consultation/ | 51669 | |
| **17** | or/13-16 | 193129 | |
| **18** | (lay-therapy or lay-therapist* or lay-worker* or lay-person* or lay-people).ti,ab,kw. | 2836 | |
| **19** | (para-professional* or non-specialist* or non-clinician* or health worker* or support worker*).ti,ab,kw. | 29728 | |
| **20** | ((unqualified or unregistered or volunt*) adj2 (therap* or worker* or coach* or facilitator* or practitioner*)).ti,ab,kw. | 1615 | |
| **21** | voluntary worker/ or exp volunteer/ | 64329 | |
| **22** | lay health worker/ or mental health care personnel/ or health auxiliary/ | 11904 | |
| **23** | or/18-22 | 103668 | |
| **24** | (self-help or self-manage*).ti,ab,kw. | 43320 | |
| **25** | self care/ or self help/ | 80302 | |
| **26** | or/24-25 | 94122 | |



For peer review only - http://bmjopen.bmj.com/site/about/guidelines.xhtml

Improving the mental health and mental health support available to adolescents in out-of-home care via Adolescent-Focused Low-Intensity Life Story Work: A realist review

| | | |
|---|---|---|
| **27** | ((group* or peer*) adj2 (intervention* or program* or service* or package* or training* or therap* or treatment)).ti,ab,kw. | 333041 |
| **28** | group therapy/ | 19771 |
| **29** | or/27-28 | 343422 |
| **30** | 17 or 23 or 26 or 29 | 706680 |
| **31** | (mental health or wellbeing or well-being).ti,ab,kw. | 349720 |
| **32** | exp mental health/ or exp mental disease/ | 2482186 |
| **33** | quality of life/ or exp wellbeing/ | 614976 |
| **34** | or/31-33 | 3026040 |
| **35** | 3 and 12 and 30 and 34 | 283 |
| **36** | limit 35 to english language | 274 |

For peer review only



For peer review only - http://bmjopen.bmj.com/site/about/guidelines.xhtml

Improving the mental health and mental health support available to adolescents in out-of-home care via Adolescent-Focused Low-Intensity Life Story Work: A realist review

PsycINFO

**Ovid PsycINFO 1806 to Present**

Search run on 2 February 2022

| | Searches | Results | Type |
|---|---|---|---|
| **1** | (teen* or youth* or adolescen* or juvenile*).ti,ab. | 344737 | |
| **2** | looked-after.ti,ab. | 662 | |
| **3** | ((foster* or social or public or state or local authority or residential or institutional or permanent or kinship or relative or substitute or out-of-home or shelter or surrogate) adj1 care*).ti,ab. | 19672 | |
| **4** | (care leaver* or care-experience* or leaving care or care transition*).ti,ab. | 2980 | |
| **5** | child welfare.ti,ab. | 7836 | |
| **6** | adoption (child)/ or adopted children/ or adoptive parents/ | 5337 | |
| **7** | foster care/ or foster children/ or foster parents/ | 7582 | |
| **8** | or/2-7 | 34369 | |
| **9** | ((adopted or adoption or adoptive or adoptee* or in-care or "in care") adj3 (teen* or youth* or adolescen* or juvenile* or young* or child* or girl? or boy?)).ti,ab. | 5753 | |
| **10** | 8 or 9 | 36284 | |
| **11** | ((low-intensity or minimal or brief or online or internet or tele* or mobile* or e-health or m-health or virtual) adj2 (intervention* or program* or service* or package* or training* or therap* or treatment*)).ti,ab. | 28828 | |
| **12** | exp computer assisted therapy/ | 11642 | |
| **13** | exp computer assisted instruction/ | 21848 | |
| **14** | or/11-13 | 57608 | |
| **15** | (lay-therapy or lay-therapist* or lay-worker* or lay-person* or lay-people).ti,ab. | 1793 | |
| **16** | (para-professional* or non-specialist* or non-clinician* or health worker* or support worker*).ti,ab. | 7045 | |
| **17** | ((unqualified or unregistered or volunt*) adj2 (therap* or worker* or coach* or facilitator* or practitioner*)).ti,ab. | 746 | |
| **18** | volunteers/ | 5507 | |
| **19** | or/15-18 | 14762 | |
| **20** | (self-help or self-manage*).ti,ab. | 19273 | |
| **21** | self-care/ | 3097 | |
| **22** | self-help techniques/ | 4398 | |
| **23** | or/20-22 | 23360 | |
| **24** | ((group* or peer*) adj2 (intervention* or program* or service* or package* or training* or therap* or treatment)).ti,ab. | 75638 | |
| **25** | group psychotherapy/ | 20529 | |



For peer review only - http://bmjopen.bmj.com/site/about/guidelines.xhtml

Improving the mental health and mental health support available to adolescents in out-of-home care via Adolescent-Focused Low-Intensity Life Story Work: A realist review

| | | |
|---|---|---|
| **26** | support groups/ | 4449 |
| **27** | or/24-26 | 86800 |
| **28** | 14 or 19 or 23 or 27 | 173409 |
| **29** | (mental health or wellbeing or well-being).ti,ab. | 289304 |
| **30** | exp mental health/ | 77446 |
| **31** | exp mental disorders/ | 919736 |
| **32** | well being/ or exp spiritual well being/ | 51717 |
| **33** | quality of life/ | 44133 |
| **34** | or/29-33 | 1164452 |
| **35** | 1 and 10 and 28 and 34 | 137 |
| **36** | limit 35 to english language | 130 |

For peer review only


For peer review only - http://bmjopen.bmj.com/site/about/guidelines.xhtml

Improving the mental health and mental health support available to adolescents in out-of-home care via Adolescent-Focused Low-Intensity Life Story Work: A realist review

## Proquest Sociology Collection

**ASSIA (Applied Social Sciences Index & Abstracts) (1987 to present); Sociological Abstracts (1952 to present); Sociology Database (1985 to present)**

Search run on 2 February 2022

| | Searches | Results | Type |
|---|---|---|---|
| **Full String** | (noft((teen* OR youth* OR adolescen* OR juvenile*)) AND (noft("looked after" OR "looked-after") OR noft((foster* OR social OR public OR state OR "local authority" OR residential OR institutional OR permanent OR kinship OR relative OR substitute OR "out of home" OR out-of-home OR shelter OR surrogate) N/1 care*) OR noft("care leaver" OR "care experience*" OR care-experience* OR "leaving care" OR "care transition*") OR noft("child welfare") OR noft(((adopted or adoption or adoptive or adoptee* or in-care or "in care") N/3 (teen* or youth* or adolescen* or juvenile* or young* or child* or girl? or boy?)))) AND (noft((low-intensity or minimal or brief or online or internet or tele* or mobile* or e-health or m-health or virtual) N/2 (intervention* or program* or service* or package* or training* or therap* or treatment*)) OR noft((lay-therapy or lay-therapist* or lay-worker* or lay-person* or lay-people)) OR noft((para-professional* or non-specialist* or non-clinician* or ("health worker" OR "health workers") or ("support worker" OR "support workers"))) OR noft((unqualified OR unregistered OR volunt*) NEAR/2 (therap* OR worker* OR coach* OR facilitator* OR practitioner*)) OR noft((self-help OR self-manage*)) OR noft((group* or peer*) N/2 (intervention* or program* or service* or package* or training* or therap* or treatment)))) AND noft("mental health" or wellbeing or well-being) | 249 | |
| | (Filter: English language) | | |

Improving the mental health and mental health support available to adolescents in out-of-home care via Adolescent-Focused Low-Intensity Life Story Work: A realist review

CINAHL

**Ebscohost CINAHL (Cumulative Index to Nursing and Allied Health Literature) (Start date unknown)**

Search run 2 February 2022

| | Searches | Results | Type |
|---|---|---|---|
| **S32** | S25 AND S30 | 471 | |
| **S31** | S25 AND S30 | 477 | |
| **S30** | S26 OR S27 OR S28 OR S29 | 835,613 | |
| **S29** | (MH "Quality of Life") | 127,552 | |
| **S28** | (MH "Mental Disorders+") | 611,172 | |
| **S27** | (MH "Mental Health") | 46,077 | |
| **S26** | TI ( "mental health" OR wellbeing OR well-being ) OR AB ( "mental health" or wellbeing or well-being ) | 182,035 | |
| **S25** | S12 AND S24 | 1,451 | |
| **S24** | S13 OR S14 OR S15 OR S16 OR S17 OR S18 OR S19 OR S20 OR S21 OR S22 OR S23 | 216,784 | |
| **S23** | (MH "Support Groups") | 11,056 | |
| **S22** | TI ( ((group* or peer*) N2 (intervention* or program* or service* or package* or training* or therap* or treatment)) ) OR AB ( ((group* or peer*) N2 (intervention* or program* or service* or package* or training* or therap* or treatment)) ) | 99,368 | |
| **S21** | (MH "Self Care") | 42,907 | |
| **S20** | TI ( "self help" OR self-help OR "self manage*" OR self-manage* ) OR AB ( "self help" OR self-help OR "self manage*" OR self-manage* ) | 20,838 | |
| **S19** | (MH "Community Health Workers") | 4,008 | |
| **S18** | TI ( ((unqualified or unregistered or volunt*) N2 (therap* or worker* or coach* or facilitator* or practitioner*)) ) OR AB ( ((unqualified or unregistered or volunt*) N2 (therap* or worker* or coach* or facilitator* or practitioner*)) ) | 962 | |
| **S17** | TI ( ("lay therapy" or lay-therapy or "lay therapist*" or lay-therapist* or "lay worker*" or lay-worker* or "lay person*" or lay-person* or "lay people" or lay-people) ) AND AB ( ("lay therapy" or lay-therapy or "lay therapist*" or lay-therapist* or "lay worker*" or lay-worker* or "lay person*" or lay-person* or "lay people" or lay-people) ) | 82 | |
| **S16** | (MH "Telemedicine") OR (MH "Remote Consultation") OR (MH "Telepsychiatry") | 17,115 | |
| **S15** | (MH "Computer Assisted Instruction") | 8,174 | |



For peer review only - http://bmjopen.bmj.com/site/about/guidelines.xhtml

Improving the mental health and mental health support available to adolescents in out-of-home care via Adolescent-Focused Low-Intensity Life Story Work: A realist review

| | | |
|---|---|---|
| **S14** | (MH "Therapy, Computer Assisted") | 5,457 |
| **S13** | TI ( ((low-intensity or "low intensity" or minimal or brief or online or internet or tele* or mobile* or e-health or m-health or virtual) N2 (intervention* or program* or service* or package* or training* or therap* or treatment*)) ) OR AB ( ((low-intensity or "ow intensity" or minimal or brief or online or internet or tele* or mobile* or e-health or m-health or virtual) N2 (intervention* or program* or service* or package* or training* or therap* or treatment*)) ) | 34,674 |
| **S12** | S10 AND S11 | 19,442 |
| **S11** | S3 OR S4 OR S5 OR S6 OR S7 OR S8 OR S9 | 77,416 |
| **S10** | S1 OR S2 | 626,344 |
| **S9** | (MH "Foster Home Care") OR (MH "Foster Parents") OR (MH "Child, Foster") | 6,425 |
| **S8** | (MH "Adoption") OR (MH "Child, Adopted") OR (MH "Adoptive Parents") | 4,102 |
| **S7** | TI ( ((adopted OR adoption OR adoptive OR adoptee* OR "in-care" OR "in care") N2 (teen* OR youth* OR adolescen* OR juvenile* or young* OR child*)) ) OR AB ( ((adopted OR adoption OR adoptive OR adoptee* OR "in-care" OR "in care") N2 (teen* OR youth* OR adolescen* OR juvenile* OR young* OR child*)) ) | 37,133 |
| **S6** | TI "child welfare" OR AB "child welfare" | 4,278 |
| **S5** | TI ("care leaver" OR "care experience*" OR care-experience* OR "leaving care" OR "care transition*") OR AB ("care leaver" OR "care experience*" OR care-experience* OR "leaving care" OR "care transition*") | 5,533 |
| **S4** | TI (foster* OR social OR public OR state OR "local authority" OR residential OR institutional OR permanent OR kinship OR relative OR substitute OR "out of home" OR out-of-home OR shelter OR surrogate) N1 care* OR AB (foster* OR social OR public OR state OR "local authority" OR residential OR institutional OR permanent OR kinship OR relative OR substitute OR "out of home" OR out-of-home OR shelter OR surrogate) N1 care* | 31,528 |
| **S3** | TI "looked after" OR looked-after OR AB "looked after" OR looked-after | 723 |
| **S2** | (MH ""Adolescence"") | 569,286 |
| **S1** | TI ( teen* OR youth* OR adolescen* OR juvenile* ) OR AB ( teen* OR youth* OR adolescen* OR juvenile* ) | 209,502 |



For peer review only - http://bmjopen.bmj.com/site/about/guidelines.xhtml

Improving the mental health and mental health support available to adolescents in out-of-home care via Adolescent-Focused Low-Intensity Life Story Work: A realist review

For peer review only



For peer review only - http://bmjopen.bmj.com/site/about/guidelines.xhtml

Improving the mental health and mental health support available to adolescents in out-of-home care via Adolescent-Focused Low-Intensity Life Story Work: A realist review

CDAS

**Ebscohost CDAS (Child Development and Adolescent Studies) (Start date unknown)**

Search run 3 February 2022

| | Searches | Results | Type |
|---|---|---|---|
| **S1** | TI ( teen* OR youth* OR adolescen* OR juvenile* ) OR AB ( teen* OR youth* OR adolescen* OR juvenile* ) | 99,382 | |
| **S2** | TI "looked after" OR looked-after OR AB "looked after" OR looked-after | 698 | |
| **S3** | TI (foster* OR social OR public OR state OR "local authority" OR residential OR institutional OR permanent OR kinship OR relative OR substitute OR "out of home" OR out-of-home OR shelter OR surrogate) N1 care* OR AB (foster* OR social OR public OR state OR "local authority" OR residential OR institutional OR permanent OR kinship OR relative OR substitute OR "out of home" OR out-of-home OR shelter OR surrogate) N1 care* | 7434 | |
| **S4** | TI ("care leaver" OR "care experience*" OR care-experience* OR "leaving care" OR "care transition*") OR AB ("care leaver" OR "care experience*" OR care-experience* OR "leaving care" OR "care transition*") | 656 | |
| **S5** | TI "child welfare" OR AB "child welfare" | 5752 | |
| **S6** | TI ( ((adopted OR adoption OR adoptive OR adoptee* OR "in-care" OR "in care") N2 (teen* OR youth* OR adolescen* OR juvenile* or young* OR child*)) ) OR AB ( ((adopted OR adoption OR adoptive OR adoptee* OR "in-care" OR "in care") N2 (teen* OR youth* OR adolescen* OR juvenile* OR young* OR child*)) ) | 14908 | |
| **S7** | TI ( ((low-intensity or "low intensity" or minimal or brief or online or internet or tele* or mobile* or e-health or m-health or virtual) N2 (intervention* or program* or service* or package* or training* or therap* or treatment*)) ) OR AB ( ((low-intensity or "ow intensity" or minimal or brief or online or internet or tele* or mobile* or e-health or m-health or virtual) N2 (intervention* or program* or service* or package* or training* or therap* or treatment*)) ) | 2481 | |
| **S8** | TI ( ("lay therapy" or lay-therapy or "lay therapist*" or lay-therapist* or "lay worker*" or lay-worker* or "lay person*" or lay-person* or "lay people" or lay-people) ) AND AB ( ("lay therapy" or lay-therapy or "lay therapist*" or lay-therapist* or "lay worker*" or lay-worker* or "lay | 5 | |



For peer review only - http://bmjopen.bmj.com/site/about/guidelines.xhtml

Improving the mental health and mental health support available to adolescents in out-of-home care via Adolescent-Focused Low-Intensity Life Story Work: A realist review

| | | |
|---|---|---|
| | person*" or lay-person* or "lay people" or lay-people) ) | |
| **S9** | TI ( ((unqualified or unregistered or volunt*) N2 (therap* or worker* or coach* or facilitator* or practitioner*)) ) OR AB ( ((unqualified or unregistered or volunt*) N2 (therap* or worker* or coach* or facilitator* or practitioner*)) ) | 78 |
| **S10** | TI ( "self help" OR self-help OR "self manage*" OR self-manage* ) OR AB ( "self help" OR self-help OR "self manage*" OR self-manage* ) | 804 |
| **S11** | TI ( ((group* or peer*) N2 (intervention* or program* or service* or package* or training* or therap* or treatment)) ) OR AB ( ((group* or peer*) N2 (intervention* or program* or service* or package* or training* or therap* or treatment)) ) | 6798 |
| **S12** | TI ( "mental health" OR wellbeing OR well-being ) OR AB ( "mental health" or wellbeing or well-being ) | 24106 |
| **S13** | S2 OR S3 OR S4 OR S5 OR S6 | 22614 |
| **S14** | S7 OR S8 OR S9 OR S10 OR S11 | 9795 |
| **S15** | S1 AND S12 AND S13 AND S14 | 61 |


For peer review only - http://bmjopen.bmj.com/site/about/guidelines.xhtml

Improving the mental health and mental health support available to adolescents in out-of-home care via Adolescent-Focused Low-Intensity Life Story Work: A realist review

## Web of Science

**Clarivate Web of Science Core Collection (SCI-EXPANDED and SSCI indexes) 1900 to present**

Search run 2 February 2022

| | Searches | Results | Type |
|---|---|---|---|
| 1 | TS=(((teen* OR youth* OR adolescen* OR juvenile*))) | 757523 | |
| 2 | TS=(("looked after" OR "looked-after")) | 776 | |
| 3 | TS=((foster* OR social OR public OR state OR "local authority" OR residential OR institutional OR permanent OR kinship OR relative OR substitute OR "out of home" OR out-of-home OR shelter OR surrogate) NEAR/1 care*) | 43729 | |
| 4 | TS=("care leaver" OR "care experience*" OR care-experience* OR "leaving care" OR "care transition*") | 6760 | |
| 5 | TS=("child welfare") | 8425 | |
| 6 | TS=(((adopted or adoption or adoptive or adoptee* or "in-care" or "in care") NEAR/3 (teen* or youth* or adolescen* or juvenile* or young* or child* or girl? or boy?))) | 7120 | |
| 7 | or/2-6 | 61902 | |
| 8 | TS=(((low-intensity or minimal or brief or online or internet or tele* or mobile* or e-health or m-health or virtual) NEAR/2 (intervention* or program* or service* or package* or training* or therap* or treatment*))) | 90429 | |
| 9 | TS=((lay-therapy or lay-therapist* or lay-worker* or lay-person* or lay-people)) | 2561 | |
| 10 | TS=((para-professional* or non-specialist* or non-clinician* or health worker* or support worker*)) | 120315 | |
| 11 | TS=(((unqualified or unregistered or volunt*) NEAR/2 (therap* or worker* or coach* or facilitator* or practitioner*))) | 1938 | |
| 12 | TS=(self-help or self-manage*) | 41426 | |
| 13 | TS=((group* or peer*) NEAR/2 (intervention* or program* or service* or package* or training* or therap* or treatment)) | 263661 | |
| 14 | #13 OR #12 OR #11 OR #10 OR #8 OR #9 | 499546 | |
| 15 | TS=((mental health or wellbeing or well-being)) | 409139 | |
| 16 | #15 and #14 and #7 and #1 | | |
| 17 | limit to english language | | |


For peer review only - http://bmjopen.bmj.com/site/about/guidelines.xhtml

Improving the mental health and mental health support available to adolescents in out-of-home care via Adolescent-Focused Low-Intensity Life Story Work: A realist review

## Social Care Online

**SCIE Social Care Online 1980 to present**

Search run 3 February 2022

| | Searches | Results | Type |
|---|---|---|---|
| **1** | teen or youth or adolescent or juvenile | | |
| **2** | looked after OR "foster care" OR "social care" OR "public care" OR "state care" OR "local authority care" OR "residential care" OR "institutional care" OR "permanent care" OR "kinship care" OR "relative care" OR "substitute care" OR "out of home care" OR "shelter care" OR adopted OR adoption OR adoptive OR adoptee | | |
| **3** | *low intensity OR minimal OR brief OR online OR digital OR internet OR tele OR mobile OR e-health OR m-health OR virtual* | | |
| **4** | mental health OR wellbeing OR "well being" | | |
| **5** | 1 and 2 and 3 and 4 | 29 | |
| | | | |
| **1** | teen or youth or adolescent or juvenile | | |
| **2** | looked after OR "foster care" OR "social care" OR "public care" OR "state care" OR "local authority care" OR "residential care" OR "institutional care" OR "permanent care" OR "kinship care" OR "relative care" OR "substitute care" OR "out of home care" OR "shelter care" OR adopted OR adoption OR adoptive OR adoptee | | |
| **3** | *"lay therapy" OR "lay therapist" OR "lay worker" OR "lay person" OR "lay people" OR "paraprofessional" OR "non-specialist" OR "non-clinician" OR "health worker" OR coach OR facilitator OR practitioner OR unqualified OR unregistered OR volunteer* | | |
| **4** | mental health OR wellbeing OR "well being" | | |
| **5** | 1 and 2 and 3 and 4 | 7 | |
| | | | |
| **1** | teen or youth or adolescent or juvenile | | |
| **2** | looked after OR "foster care" OR "social care" OR "public care" OR "state care" OR "local authority care" OR "residential care" OR "institutional care" OR "permanent care" OR "kinship care" OR "relative care" OR "substitute care" OR "out of home care" OR "shelter care" OR adopted OR adoption OR adoptive OR adoptee | | |
| **3** | *"self care" OR "self help" OR "self management" OR group OR peer* | | |
| **4** | mental health OR wellbeing OR "well being" | | |
| **5** | 1 and 2 and 3 and 4 | 21 | |
| | | | |
| | NB *search strategies adapted for simpler search interface and run in three separate queries* | | |



For peer review only - http://bmjopen.bmj.com/site/about/guidelines.xhtml

Improving the mental health and mental health support available to adolescents in out-of-home care via Adolescent-Focused Low-Intensity Life Story Work: A realist review

For peer review only



For peer review only - http://bmjopen.bmj.com/site/about/guidelines.xhtml

Improving the mental health and mental health support available to adolescents in out-of-home care via Adolescent-Focused Low-Intensity Life Story Work: A realist review

## Supplementary File 2: Detailed summary of the included documents

| Authors | Year | Country | Title | Publication type/ Study Design/ Methods | Sample/ Setting | Objectives |
|---|---|---|---|---|---|---|
| ARCBOX [93] | | United Kingdom (UK) | Digital Life Story | Webpage | Adoption | To describe a digital platform for adopted young people to collect memories throughout their life from the past to the present day. |
| Atwell [97] | 2016 | UK | Working with children with a disability. | Book Chapter | Across all settings | To discuss Life Story Work (LSW) with children and young people with special educational needs or disabilities (SEND). |
| Atwool [43] | 2017 | New Zealand | Life Story Work Optional Extra or Fundamental Entitlement. | Journal, Commentary/ practice guidance | Across all settings | To discuss LSW practice in New Zealand and the reasons why LSW is fundamental to young people with care experience. |
| Aust [87] | 1981 | United States of America (USA) | Using the Life Story Book in Treatment of Children in Placement | Journal, Case Study | Foster Placement | To describe the use of life story books in foster placements using a case study example. |
| Aventin, Houston, & Macdonald [66] | 2014 | UK | Utilising a computer game as a therapeutic intervention for youth in residential care Some preliminary findings | Journal, Semi-structured interviews | Residential placement (N = 16 consisting of 8 young person and | To explore the accessibility and therapeutic impact of a computer-based intervention delivered to young people in residential placements by their key worker. |

Improving the mental health and mental health support available to adolescents in out-of-home care via Adolescent-Focused Low-Intensity Life Story Work: A realist review

| | | | | | | |
|---|---|---|---|---|---|---|
| | | | on use and acceptability. | | worker pairs) | |
| BASW [73] | 2020 | UK | Recording in children's social work | Commentary/ practice guidance | Across all settings | To discuss recording in social work providing ten recommendations for practice. |
| Baynes [18] | 2008 | UK | Untold stories A discussion of life story work. | Journal, Commentary/ practice guidance | Across all settings | To discuss LSW practice by social care professionals and the issues surrounding practice. |
| Bazalgette, Rahilly &Trevelyan [91] | 2015 | UK | Achieving-emotional-wellbeing-for-looked-after children a whole system approach | Report, Commentary/ practice guidance | Across all settings | To discuss recommendations based on research for a whole system approach to improving the emotional wellbeing of young people with care experience by delivering preventative as opposed to crisis driven interventions, including LSW. |
| Beste & Richardson [92] | 1981 | USA | Developing a Life Story Book Program for Foster Children | Journal, Commentary/ practice guidance | Across all settings | To describe a training programme for foster carers to support young people to create a Life Story Book. |
| Bolton Council [58] | 2022 | UK | Life Story Work 5 minute facts | Webpage, Commentary/ practice guidance | Foster placement | To provide guidance for carrying out LSW within foster placements. |
| Braiden [62] | 2016 | UK | Evaluation of a short training programme for foster carers | Journal, Case study | Foster placement | To discuss a training package for foster carers that provides skills around art and creative therapy they can engage in with the young person in their care. |
| Brookfield, Brown, & Reavey [60] | 2008 | UK | Vicarious and Post-memory Practices in Adopting Families: The Re-production of the Past through | Journal, Focus Groups | Adoption (n=12 adoptive parents) | To explore adoptive parents experience of supporting adoptive children with life story work. |

Improving the mental health and mental health support available to adolescents in out-of-home care via Adolescent-Focused Low-Intensity Life Story Work: A realist review

| | | | | | | |
|---|---|---|---|---|---|---|
| | | | Photography and Narrative | | | |
| Buchanan [36] | 2014 | UK | The experience of life story work reflections of young people leaving care. | Thesis, semi-structured interviews | Care leavers | To explore care leavers experiences of LSW, highlighting the importance of taking a person-centred approach, trusted relationships and involving birth relatives for successful LSW. |
| Connor, Sclare, Dunbar and Elliffe [79] | 1985 | UK | Making a life story book | Journal, Case Study | Residential Placement | To discuss making a life story book with a young person in a residential placement, highlights topics of planning, relationship building and access to supervision. |
| Cook-Cottone & Beck [19] | 2007 | USA | A Model for Life-Story Work Facilitating the Construction of Personal Narrative for Foster Children | Journal, Commentary/ practice guidance | Foster placement | To discuss life story work and theoretical frameworks around LSW. Introduces a model for life story work for children in foster care. |
| Davies & Hodges [76] | 2017 | UK | Relationship renaissance the use of attachment-based narrative and metaphor in life story work | Journal, Case Study | Foster placement (n=3) | To describe an attachment based narrative approach to LSW presenting two case studies of the approach in practice. |
| Devenney [90] | 2017 | UK | Pathway planning with unaccompanied young people leaving care Biographical narratives of past, present, and future | Journal, Interviews | unaccompanied care leavers (n=18) Social work professionals (n=12) | To explore how unaccompanied young people create biographical narratives of their past, present and future. |

Improving the mental health and mental health support available to adolescents in out-of-home care via Adolescent-Focused Low-Intensity Life Story Work: A realist review

| | | | | | | |
|---|---|---|---|---|---|---|
| Eenshuistra, Harder, & Knorth [103] | 2019 | Netherlands | One size does not fit all A systematic review of training outcomes on residential youth care professionals' skills | Journal, Systematic Review | Residential placement | To review training programmes for residential placement workers. |
| Eldridge [65] | 2018 | UK | Confiding in Others: A Qualitative Study Exploring the Experiences of Young people who have been in the Care System | Thesis, semi-structured interviews | Across all settings | To explore factors associated with confiding relationships and what elements are influential on an individual confiding in others. |
| Ferrier [54] | 2011 | UK | Life story work in the context of attachment led care planning | Journal, Commentary/ practice guidance | Residential placement | To discuss a model of LSW that uses an attachment-based theoretical framework for carrying out LSW in residential placements. |
| Finlay [70] | 2022 | UK | Meaningful natural mentoring relationship characteristics and informal therapeutic life space interactions for youth in care | Thesis, systematic review and interviews | Therapeutic care workers (n=8) | To explore factors that contribute to 'natural mentoring relationships' and trusting relationships with young people with care experience by looking at the perspectives of workers within a residential setting in UK. |
| Fitzhardinge [81] | 2008 | Australia | Adoption, resilience and the importance of stories The making of a film about teenage adoptees. | Journal, Commentary/ practice guidance | Adoption (n=6) | To discuss a group project where adopted young people work as a group to make a film about being adopted. |

Improving the mental health and mental health support available to adolescents in out-of-home care via Adolescent-Focused Low-Intensity Life Story Work: A realist review

| | | | | | | |
|---|---|---|---|---|---|---|
| Fostering Sunderland [46] | 2021 | UK | Foster Carer Handbook | Webpage, Practice guidance | Foster Placement | To provide guidance to foster carers. |
| Furnivall & Grant [75] | 2014 | UK | Trauma sensitive practice with children in care | Report | | To discuss the importance of social care practitioners being trauma informed to improve their practice when working with young people with care experience. |
| Gallagher & Green [26] | 2012 | UK | In, out and after care Young adults' views on their lives, as children, in a therapeutic residential establishment. | Journal, Semi-structured interviews | Residential placement (n=16) | To explore young people with care experience views of their experience in residential care including their experience of LSW. |
| Gray, Hahn, Cater, Watson, Meineck, & Metcalfe [99] | 2019 | UK | Trove A Digitally Enhanced Memory Box for Looked after and adopted children | Conference paper, report | Across all settings | To discuss Trove, a digital memory box used with in LSW, it's development, intended purpose and uses. |
| Gustavsson & MacEachron [52] | 2008 | USA | Creating Foster Care Youth Biographies A Role for the Internet. | Journal, Commentary/ practice guidance | Across all settings | To describe a digital LSW approach where digital records are made for young people with care experience. |
| Gutsche [67] | 2013 | South Africa | Perceptions of social workers regarding life story work with children in child youth centres. | Thesis, semi-structured interviews and focus groups | Residential placement Social workers (n=6) | To explore social workers views in South Africa of LSW with young people living in South Africa's equivalent of residential placements. |

Improving the mental health and mental health support available to adolescents in out-of-home care via Adolescent-Focused Low-Intensity Life Story Work: A realist review

| | | | | | | |
|---|---|---|---|---|---|---|
| Haight, Black, & Sheridan [68] | 2010 | USA | A Mental Health Intervention for Rural, Foster Children from Methamphetamine-involved Families Experimental Assessment with Qualitative Elaboration | Journal, Randomised | Foster placement (n=23) | To evaluate the efficacy of a LSW intervention delivered by individuals from the community to young people living in foster placements. |
| Hamilton [64] | 2020 | UK | Life story approaches and relationships within residential child care A practice reflection. E. Hamilton | Journal, Case Study | Residential placement | To explore the importance of everyday interactions to support life stories for young people within residential care using a case study as an example. |
| Hammond [37] | 2012 | UK | Exploring a role for digital technologies in life story work with adolescents in residential care a discourse analysis | Thesis, ethnographic observations, interviews and focus groups | Residential placement (10 young people, 35 carer) | To explore the use of novel digital technologies as tools to facilitate LSW with adolescents in residential placements. |
| Hammond & Cooper [47] | 2013 | UK | Digital Life Story Work using technology to help young people make sense of their experience | Book | Across all settings | To discuss digital LSW approaches that can be used in practice with young people with care experience. |
| Hammond [48] | 2016 | UK | Digital Life Story Work in Action. | Book chapter | Across all settings | To discuss digital LSW approaches that can be used in practice with young people with care experience. |

Improving the mental health and mental health support available to adolescents in out-of-home care via Adolescent-Focused Low-Intensity Life Story Work: A realist review

| | | | | | | |
|---|---|---|---|---|---|---|
| Hammond, Cooper & Jordan [49] | 2021 | UK | Mental health, identity and informal education opportunities for adolescents with experience of living in state care: a role for digital storytelling, | Journal, Commentary/ practice guidance | Residential placement (10 young people, 35 carer) | To discuss digital storytelling, how both formal and informal educators can play a role in supporting young people with care experiences use of digital technologies within LSW. |
| Hanna [77] | 2007 | USA | Preparing School Age Children for Adoption | Journal, Interviews | Adoption - social care professional s (n=26) and adoptive parents (n=55) | To explore how older children are prepared for adoption, an included topic discussed is life story work/books. |
| Happer, McCreadie, & Aldgate [86] | 2006 | UK | Celebrating Success: What Helps Looked After Children Succeed | Book | Across all settings | To explore young people with care experiences views on their successes and the factors that promoted success for them while being looked after by the local authority. |
| Hertfordshire Children's Specialist and Safeguarding Services [38] | 2022 | UK | Direct Work and Life Story Work with Children and young people | Webpage, Commentary/ practice guidance | Adoptive and foster placements | To provide guidance on how LSW should be carried out in practice making a distinction between adoptive and foster placements and how these different contexts impact LSW. |
| Hills [57] | 2022 | UK | Ensuring life stories are at the centre of direct work | Podcast | Across all settings | To discuss a model of practice that ensures LSW is woven into all interactions with young people in contact with social care services. |
| Holody & Mäher [39] | 1996 | USA | Using lifebooks with children in family foster care a here | Journal, Commentary/ practice guidance | Foster placement | To discuss the 'here and now' model for supporting young people in foster placements with life story books using case examples. |

Improving the mental health and mental health support available to adolescents in out-of-home care via Adolescent-Focused Low-Intensity Life Story Work: A realist review

| | | | | | | |
|---|---|---|---|---|---|---|
| | | | and now process model | | | |
| Hooley [55] | 2015 | UK | Identifying perspectives on life story work with looked after and adopted children | Thesis, Q-methodology | Clinical psychologists and therapists, social work professionals, foster carers, adoptive parents, care leavers (N= 29) | To explore how different aspects of LSW that are important to different groups of people using Q-Methodology. |
| Hooley, Stokes & Combes [27] | 2016 | UK | Life story work with looked after and adopted children how professional training and experience determine perceptions of its value | Journal, mixed methods | Clinical psychologists and therapists, social work professionals, foster carers, adoptive parents, care leavers (N= 29) | To investigate which aspects of LSW are important to different groups of people using Q-Methodology. |
| Hoyle, Shepherd, Lomas, & Flinn [44] | 2020 | UK | Recordkeeping and the life long memory and identity needs of care experienced children and young people | Journal, Interviews | N=21 care leavers | To explores record keeping practices in social care settings. |

Improving the mental health and mental health support available to adolescents in out-of-home care via Adolescent-Focused Low-Intensity Life Story Work: A realist review

| | | | | | | |
|---|---|---|---|---|---|---|
| Hughes [72] | 2013 | UK | Life-story work a journey into a child's world Seen and Heard | Commentary/ practice guidance | Across all settings | To discuss different LSW models using case examples to describe their uses |
| Humphreys, & Kertesz [102] | 2014 | Australia | Making Records Meaningful Creating an Identity Resource for young people in care | Journal, Survey and focus groups | Survey n=37 young people with care experience by foster care and residential placement. Focus group n=5 social care workers, n=2 young people current care experience, n=4 care leavers. | To explore record keeping practices in Australia to support identity from key stakeholders. |
| Känkänen & Bardy [80] | 2014 | Norway | Life stories and arts in child welfare enriching communication | Commentary/ practice guidance | Residential placement | To discuss the authors experiences of using participatory arts within LSW in residential placements. |
| Lucas, Matthews, L. Brady, Breguet, & Parson [53] | 2020 | Australia | Therapeutic-Life-Story-Work-Barwon-Pilot-Evaluation | Interim report | Across all settings | To evaluate the implementation of a therapeutic LSW model in Australia. |

Improving the mental health and mental health support available to adolescents in out-of-home care via Adolescent-Focused Low-Intensity Life Story Work: A realist review

| | | | | | | |
|---|---|---|---|---|---|---|
| Malik [63] | 2005 | USA | Application of attachment theory for training foster parents a model program | Thesis, semi-structured interviews | Foster carers | To discuss the development of a training programme for foster carers. |
| Monson [94] | 2020 | Australia | Promoting mental health in out of home care in Australia | Journal, focus group and interviews | N=14 young people with care experience | To explore the experience of young people with care experience in Australia on what promotes positive mental health. |
| Neil & Beek [59] | 2020 | UK | Respecting Children's Relationships and Identities in Adoption. | Book Chapter | Adoption | To discuss how adopted young people can be supported to maintain significant relationships while outlining key principles for supporting transitions for adoptive families, drawing on the "Secure Base Model". |
| National Institute for Health and Care Excellence (NICE) [7] | 2021 | UK | Looked after children and young people NICE guideline 2021 | Quality standard | Across all settings | To provide the NICE guidelines for LSW with young people with care experience, including recommendations for how LSW should be delivered and what should be included. |
| Nicholls [101] | 2003 | UK | Model answer - Community Care | Commentary/ practice guidance | Across all settings | To discuss a model of LSW that focuses on a collaborative approach to securing memories for young people with care experience. |
| National Society for the Prevention of Cruelty to Children (NSPCC) [83] | 2022 | UK | Life Story Work _ NSPCC Learning 2022 | Commentary/ practice guidance | Across all settings | To discuss the NSPCC's strengths-based model of LSW that is delivered to young people with care experience. |
| Nuffield Family | 2021 | UK | Modernising post adoption contact | Report | Adoption n=80 | To discuss findings of a consultation with organisations, birth families, adoptive families, |

Improving the mental health and mental health support available to adolescents in out-of-home care via Adolescent-Focused Low-Intensity Life Story Work: A realist review

| | | | | | | |
|---|---|---|---|---|---|---|
| Justice Observatory [96] | | | findings from recent consultation | | | young people, regional and voluntary adoption agencies on post-adoption contact with birth relatives |
| Pakrosnis, & Cˇepukiene [85] | 2011 | Lithuani a | Outcomes of Solution-Focused Brief Therapy for Adolescents in Foster Care and Health Care Settings. | Book chapter, Matched pairs design | Foster and health care placement N=139 | To evaluate the efficacy of solution focused brief therapy for adolescents in Foster Care and Health Care Settings. |
| Peake [50] | 2009 | UK | Life story work A resource for foster carers, residential social workers, adoptive parents, and kinship carers, to support this work. | Booklet | Foster care | To provide guidance for foster carers and social care professionals to carry out LSW with young people with care experience. |
| Sanders [89] | 2020 | UK | Care experienced children and young people's mental health | Literature review | Across all settings | To review the factors that impact the mental health of young people with care experience. |
| Shepard [100] | 2022 | UK | Good practice in record-keeping in children's social care | Commentary/ practice guidance | Across all settings | To discuss record keeping practices in children's social care including the development of a digital tool for care experienced young people to keep a diary to preserve their memories. |
| Shotton [51] | 2012 | UK | "Remember when…" Exploring the experiences of looked after children and their carers in engaging in | Thesis, Semi-structured interviews | Foster placement (N=5 carers, N=4 young people) | To explore the experience of foster carers and looked after children using the memory store approach. |

Improving the mental health and mental health support available to adolescents in out-of-home care via Adolescent-Focused Low-Intensity Life Story Work: A realist review

| | | | | | | |
|---|---|---|---|---|---|---|
| | | | collaborative reminiscence | | | |
| Shotton [40] | 2013 | UK | Remember when...' exploring the experiences of looked after children and their carers in engaging in collaborative reminiscence | Journal, Semi-structured interviews | Foster placement (N=5 carers, N=4 young people) | To explore carers and young people's experiences of using the memory store approach to LSW using semi-structured interviews and a board game approach using Interpretative Phenomenological Analysis. |
| Shotton [71] | 2010 | UK | Telling different stories The experience of foster adoptive carers in carrying out collaborative memory work with children. | Journal, Semi-structured interviews | Adoptive parents and foster carers (n=5) | To explore adoptive and foster carers experience using the memory story approach to LSW. |
| Steenbakkers, van der Steen, & Grietens [82] | 2016 | Netherlands | 'To talk or not to talk?': Foster youth's experiences of sharing stories about their past and being in foster care | Journal, Interviews | Foster placement (n=13) | To explore young people's experiences of foster care in Netherlands. |
| Teodorczuk, Guse, & du Plessis [88] | 2018 | South Africa | The effect of positive psychology interventions on hope and well-being of adolescents living in a child and youth care centre. | Journal, Matched pairs design | Adolescents (n=29) | To investigate the efficacy of a positive psychology intervention on hope and wellbeing of young people with care experience in South Africa. |

Improving the mental health and mental health support available to adolescents in out-of-home care via Adolescent-Focused Low-Intensity Life Story Work: A realist review

| | | | | | | |
|---|---|---|---|---|---|---|
| Together for Children Sunderland [45] | 2019 | UK | Foster Carer Handbook 2019 together for children Sunderland | Commentary/ practice guidance | Foster care | To give guidance to foster carers. |
| Walker, & Ryan [97] | 2016 | UK | Working with black and minority ethnic children | Book chapter | Across all settings | To discuss LSW with black and minority ethnic children. |
| Walker, & Ryan [98] | 2016 | UK | Why do life story work? | Book chapter | Across all settings | To discuss why LSW is carried out with young people with care experience. |
| Ward [78] | 2002 | UK | Opportunity led work maximising the possibilities for therapeutic communication in everyday interactions. | Commentary/ practice guidance | Across all settings | To discuss the concept of "opportunity led working', a framework to view everyday interactions as having therapeutic potential. |
| Watson, Hahn, & Staines [41] | 2020 | UK | Storying special objects Material culture, narrative identity and life story work for children in care. | Journal, Semi-structured interviews | Social care professionals (n=9) | To explore social care professional's perspectives of the importance of material objects in LSW to support young people with care experience identity construction. |
| Watson, Latter, & Bellew [22] | 2015 | UK | Adopted children and young people's views on their life storybooks The role of narrative in the formation of identities. | Journal, Semi-structured interviews | Adopted young people (n=20) | To explore adopted children and young people's views on their life story books. |

Improving the mental health and mental health support available to adolescents in out-of-home care via Adolescent-Focused Low-Intensity Life Story Work: A realist review

| | | | | | | |
|---|---|---|---|---|---|---|
| Watson, Meineck & Lancaster [42] | 2018 | UK | Adopted children's co-production and use of 'trove' (a digitally enhanced memory box) to better understand their care histories through precious objects. | Journal, Semi-structured interviews | Adopted young people (n=10) | To explore adopted young people's experience of trialling the prototype Trove, a digital approach to facilitating LSW. |
| Watson, Staples, & Riches [61] | 2021 | UK | 'We need to understand what's going on because it's our life' using sandboxing to understand children and young people's everyday conversations about care | Journal, Semi-structured interviews | Care experienced children and young people N = 11 (5 adopted, 6 care leavers). | To explore children and young people's hopes and fears for their everyday conversations with carers about their care experience. |
| Watson, Latter & Bellew [21] | 2015 | UK | Adopters' views on their children's life story books. | Journal, Focus groups and interviews | Adopters (n=40) | To explore adopters' views on their children's life story books. |
| Watts [56] | 2021 | UK | Relationships, reviews and recording Developing practice for children in care. | Journal, Focus Groups and interviews | Social care professional s (n=42), foster carers (n=10) and young people with care | To explore stakeholders views on reviews and recording practices within social care including The Me and My World model introduced to Brighton and Hove City Council. |

For peer review only

Improving the mental health and mental health support available to adolescents in out-of-home care via Adolescent-Focused Low-Intensity Life Story Work: A realist review

| | | | | | | |
|---|---|---|---|---|---|---|
| | | | | | experience (n=4). | |
| Willis, & Holland [20] | 2009 | UK | Life story work Reflections on the experience by looked after young people | Journal, Semi-structured interviews | Young people with care experience (n=12) | To explore young people with care experiences views on LSW. |
| Wood [104] | 2019 | UK | West Sussex Children's improvement board. Performance report against the practice improvement plan improving quality of life story work. | Report | Across all settings | To report findings from an audit of West Sussex children's services LSW practice. |
| Wood, & Selwyn [74] | 2017 | UK | Looked after children and young people's views on what matters to their subjective well-being. | Journal, Focus groups | Young people with care experience (n=140) | To explore and identify the factors that children and young people identify as being indicative of subjective wellbeing. |

For peer review only

Improving the mental health and mental health support available to adolescents in out-of-home care via Adolescent-Focused Low-Intensity Life Story Work: A realist review

# Supplementary File 3: Initial practice guidelines

| Initial practice guidance recommendation |
|---|
| **1. Adolescent-Focused Low-Intensity Life Story Work should be flexible and person-centred** to ensure how, why, when, by whom and to what extent Adolescent-Focused Low-Intensity LSW is delivered, fits the needs of the individual adolescent and their circumstances. |
| **2. There is no 'bad time' to start Adolescent-Focused Low-Intensity Life Story Work so this should start early and make use of everyday opportunities.** Everyday opportunities should be used to engage the adolescent in preserving and reflecting on their life experiences. |
| **3. Adolescent-Focused Low-Intensity Life Story Work should begin in the present day as this grows trust in caring adult(s) and relationships.** Sharing everyday life experiences helps to establish trusting relationships with caring adult(s). |
| **4. Adolescent-Focused Low-Intensity Life Story Work should involve co-construction of narratives because this improves coherence and authenticity.** Adolescents should be supported to construct a narrative that makes sense and feels authentic to them. |
| **5. Adolescents should be supported to control how their lives are recorded and preserved in Adolescent-Focused Low-Intensity Life Story Work whenever and wherever possible.** Adolescents should control the processes involved so they feel ownership and power over their own story and how and what is captured. |
| **5.1 Adolescents should be supported to control the processes for recording and preserving** |
| **5.2 Adolescents should be supported to control Adolescent-Focused Low-Intensity Life Story Work products (e.g., artefacts)** |
| **6. Adolescent-Focused Low-Intensity Life Story Work should provide adolescents with somewhere to begin future telling,** supporting adolescents' communication about their lived experiences by providing storytelling prompts, helping adolescents through transitions. |
| **7. Adolescent-Focused Low-Intensity Life Story Work should record positive aspects of everyday experiences**, there should be **a** focus on the positives in adolescents lives to promote the development of positive identity and positive future expectations. |
| **8 Adolescent-Focused Low-Intensity Life Story Work should include support for adolescents, caring adults and others involved in supporting adolescents** with life story work should be adequately prepared and/or trained and supported themselves. |


For peer review only - http://bmjopen.bmj.com/site/about/guidelines.xhtml

Improving the mental health and mental health support available to adolescents in out-of-home care via Adolescent-Focused Low-Intensity Life Story Work: A realist review

## Supplementary File 4: Detailed summary of the CMOCs developed and underpinning data

| CMOC Label | CMOC | Supporting evidence | Example extracts |
|---|---|---|---|
| **CMOC1** | When low-intensity LSW is flexible and person-centred (C) better practice is achieved (O) because adaptions have been made relating to who, when, where, in what circumstances and how low intensity LSW is delivered to the adolescent (M). | (Buchanan, 2014) (Hammond, 2012) (HCC, 2022) (Holody & Mäher, 1996) (Hooley et al., 2016) (NICE, 2021) (Shotton, 2013) (Watson, Hahn, et al., 2020) (Watson et al., 2018) (Willis & Holland, 2009) | 'Take a flexible approach to life story work, and tailor it to the developmental age and needs of the looked-after child or young person' (NICE, 2021)<br><br>'The content of the LSW reported as being most helpful incorporated photos, drawings, speech bubbles, sentimental objects and opportunities for the young people to comment on their feelings and to be creative in the content. This type of child-led approach offered choice with respect to both content and format' (Buchanan, 2014)<br><br>'The findings suggest that it may not be important what form the work takes, as long as it is adapted to suit the child's interests and needs.' (Willis & Holland, 2009) |
| **CMOC2** | When everyday life experiences are preserved (C) it improves young people's ability to construct a coherent identity (O) because they have autobiographical memory cues available to re-visit (M). | (Atwool, 2017) (Buchanan, 2014) (Cook-Cottone & Beck, 2007) (Hammond, 2016) (Hammond et al., 2021) (Shotton, 2013) (Watson, Hahn, et al., 2020) (Peake, 2009) (Shotton, 2012) (Willis & Holland, 2009) (Gustavsson & MacEachron, 2008) (Hooley, 2015) | 'A tangible and visual record of the child's time in care maintained from the outset through the use of memory boxes and life story books is essential to ensure access to a coherent narrative.' (Watson, Hahn, et al., 2020) |



For peer review only - http://bmjopen.bmj.com/site/about/guidelines.xhtml

Improving the mental health and mental health support available to adolescents in out-of-home care via Adolescent-Focused Low-Intensity Life Story Work: A realist review

| | | | |
|---|---|---|---|
| | | | 'Entries assist in helping the youth preserve memories. These entries can consist of a description of a birthday party, or a school event in which the youth played a role such as a play or sporting event. These stories may someday help the youth establish a stronger sense of self based on positive memories of their past.' (Gustavsson & MacEachron, 2008) |
| **CMOC3** | When significant events are preserved through recording and made available to an adolescent when they have contact with social care (C) this helps an adolescent construct a coherent understanding of their experiences (O) because memories are available to revisit and reflect upon (M). | (Hills, 2022) (Watson et al., 2015b) (Hoyle et al., 2020) (Together for Children Sunderland, 2019) (Sunderland, 2021) (Hammond, 2012) (Hammond & Cooper, 2013) | 'Life Story Work is very important to the child and will help them in childhood and in later life gain some understanding of their identity and background and help them understand and recall their life in care. As the child's carer it is essential that you keep items for the child that can later be used for life story work, such as mementos of special occasions, photographs (including school photos) special clothes or toys.' (Together for Children Sunderland, 2019) |
| **CMOC4** | When everyday life experiences are preserved with narrative accounts (C) the ability to construct a coherent identity improves (O) because of the availability of contextual information (M). | (Watson et al., 2015a) (Atwool, 2017; Lucas et al., 2020) | 'For many of the children the absence of a "story" was a source of criticism about their own book and we were regularly told about books that contained photographs but with little account of who was in the photos or how the photos contributed to the child's story.' (Watson et al., 2015a)<br><br>'The children appeared overall to value their books even though they could see the flaws |



For peer review only - http://bmjopen.bmj.com/site/about/guidelines.xhtml

Improving the mental health and mental health support available to adolescents in out-of-home care via Adolescent-Focused Low-Intensity Life Story Work: A realist review

| | | | |
|---|---|---|---|
| | | | and were critical of these. A lack of narrative and the failure to capture multiple perspectives was a common theme.' (Atwool, 2017) |
| **CMOC5** | If adolescents are not ready to engage in low-intensity LSW, caring adults in the adolescent's support network should begin to collect and preserve everyday life experiences (C) this ensures adolescents have access to information about their care journey (O) because information is not lost or forgotten (M). | (HCC, 2022) (Ferrier, 2011) (Willis & Holland, 2009) (Cook-Cottone & Beck, 2007) (Atwool, 2017) (Hooley et al., 2016) (Watts, 2021) (Peake, 2009) (NICE, 2021) (Shotton, 2012) (Shotton, 2013) | 'The gaps in records that several young people had, including from some foster placements, reinforce the importance of gathering photographs and other mementos at each stage of a child's life, even if they do not want them immediately.' (Willis & Holland, 2009)<br><br>Start life story work as soon as possible after the looked-after child or young person enters care, to support care placement and emotional stability, rather than as an intervention to deliver once placements are stable.' (NICE, 2021)<br><br>'Some children may not feel motivated to engage with the approach, though even if they show little or no interest the carer should continue to collect memories of the child's time with them so that memories of that placement are safeguarded… Knowing that those memories have been safeguarded communicates to the child that their time is important and that they matter.' (Shotton, 2012) |



For peer review only - http://bmjopen.bmj.com/site/about/guidelines.xhtml

Improving the mental health and mental health support available to adolescents in out-of-home care via Adolescent-Focused Low-Intensity Life Story Work: A realist review

| | | | |
|---|---|---|---|
| **CMOC6** | When all caring adults within the adolescent's support network are actively involved in preserving memories and artefacts of everyday life experiences (C) it reduces the risk of there being gaps in the adolescent's autobiographical memory (O) because information is not lost (M). | (HCC, 2022) (Hills, 2022) (Atwool, 2017) (Bolton, 2022) (Watson, Hahn, et al., 2020) (Gustavsson & MacEachron, 2008) (Hammond, 2012) (Hammond et al., 2021) (Neil & Beek, 2020) (Brookfield et al., 2008) | 'Birth families, all workers involved in the child's journey should take responsibility and have a role in collecting memorabilia of all significant events/achievements for the child. This information should be recorded for the child, and any memorabilia given to the child (or held in safekeeping by the carers according to the child's age and understanding).' (HCC, 2022)<br><br>'All caregivers (including those offering short-term care) need to be equipped to support children in keeping an ongoing record of their time in care through the use of memory boxes, life story books and digital records.' (Atwool, 2017) |
| **CMOC7** | When adolescents do not have access to information about their everyday life experiences and care journey (C) they can become frustrated and angry (O) because they feel a lack of control over information about their life and their experiences (M). | (Hoyle et al., 2020) (Buchanan, 2014) (Watson, Staples, et al., 2020) | 'Their wanting information and clarity about their life was often exacerbated by their judgement that others knew more about their lives and families than they did. This was a source of frustration and anger for many.' (Buchanan, 2014)<br><br>'The common belief that others knew more than they did was often associated with a general feeling that the information others held was deliberately kept from them. A sense of information being kept secret, hidden or confused was evident, which understandably led to many feeling frustrated, |



For peer review only - http://bmjopen.bmj.com/site/about/guidelines.xhtml

Improving the mental health and mental health support available to adolescents in out-of-home care via Adolescent-Focused Low-Intensity Life Story Work: A realist review

| | | | |
|---|---|---|---|
| | | | angry and mistrustful of others' (Buchanan, 2014) |
| **CMOC8** | When caring adults and adolescents share, preserve, and reflect on everyday life experiences (C) connection and rapport between them increases (O) because they spend quality one to one time together (M). | (Hammond et al., 2021) (Braiden, 2016) (Shotton, 2012) (Holody & Mäher, 1996) (Hooley, 2015) (NICE, 2021) (Shotton, 2012) (Malik, 2005) (Buchanan, 2014) (Watson, Staples, et al., 2020) (Hamilton, 2020) (Hooley et al., 2016) (Eldridge, 2018) (Shotton, 2013) (Aventin et al., 2014) | 'All the carers talked positively about how the approach had been good for helping them to spend quality time with the child. The carers felt closer to the child through using it, and thought that the child felt closer to them as well.' (Shotton, 2012)<br><br>'Life story work has the potential for building relationships (for example, by sharing joint activities).' (NICE, 2021)<br><br>'While it may be time consuming, it can also be very rewarding such that in the process of creating this narrative and recording these memories together, a special relationship develops whereby both the child and foster parent learn more about the foster child.' (Malik, 2005) |
| **CMOC9** | When caring adults and adolescents share, preserve and reflect on everyday life experiences (C) adolescent's self-worth increases (O) because the caring adult shows they want to spend time with them, and their experiences are valuable (M). | (Malik, 2005) (Shotton, 2012) (Shotton, 2013) (Watts, 2021) (Peake, 2009) (Hooley, 2015) | 'By taking the time to complete this project, the caregiver conveys to the child that he or she is important and worth the time to get to know.' (Malik, 2005)<br><br>'Evidence within the store of children's particular preferences helps them to see that their opinions matter and are worth recording and talking about, thus contributing to their |



For peer review only - http://bmjopen.bmj.com/site/about/guidelines.xhtml

Improving the mental health and mental health support available to adolescents in out-of-home care via Adolescent-Focused Low-Intensity Life Story Work: A realist review

| | | | |
|---|---|---|---|
| | | | sense of identity and self-worth' (Shotton, 2013) |
| **CMOC10** | When caring adults and adolescents share, preserve, and reflect on everyday life experiences (C) it helps to establish a safe relationship for the exploration of thoughts and feelings (O) because they establish a point of connection, rapport, and trust (M). | (Aventin et al., 2014) (Gutsche, 2013) (Hammond et al., 2021) (Haight et al., 2010) (Watson, Staples, et al., 2020) (Shotton, 2012) (Finlay, 2022) (Hamilton, 2020) (Holody & Mäher, 1996) (Shotton, 2013) (Hooley, 2015) (Hamilton, 2020) (Willis & Holland, 2009) (Buchanan, 2014) | 'The participants claimed that often the children preferred drawing or doing arts and crafts to talking, for example, and this would sometimes prompt them to talk more as they built their confidence in their therapeutic relationship with the social worker.' (Gutsche, 2013)<br><br>'As in conventional LSW with younger children with care-experience, the trusted adult relationship context and engagement with reflective activities created opportunities for a therapeutic alliance between adolescent participants and the first author. In this space, adolescent participants were able to express and begin to reflect on difficult events in their lives' (Hammond et al., 2021) |
| **CMOC11** | When adolescents' everyday life experiences are recorded (C) they can share it with others in the future (O) because prompts from events have been saved (M). | (Shotton, 2012) (Shotton, 2013) (Watson, Hahn, et al., 2020) (Hammond, 2012) (Atwool, 2017) (Hanna, 2007) | 'In looking through the store both carers and children were able to flesh out each memory with details of when it occurred or who else was there. It was clear that the pictorial information was very powerful in taking the participants back to the time and place when it occurred.' (Shotton, 2013)<br><br>'Importantly, children's 'stuff' can be used to help the child and new family to understand their journey' (Watson, Hahn, et al., 2020) |



For peer review only - http://bmjopen.bmj.com/site/about/guidelines.xhtml

Improving the mental health and mental health support available to adolescents in out-of-home care via Adolescent-Focused Low-Intensity Life Story Work: A realist review

| | | | |
|---|---|---|---|
| **CMOC12** | Sharing, preserving, and reflecting on everyday life experiences with a caring adult (C) increases an adolescent's sense of connectedness and understanding of themselves and others (O) because it provides opportunities to talk and be listened to (M) | (Shotton, 2010) (Watts, 2021) (Eldridge, 2018) (Shotton, 2012) (Watson, Staples, et al., 2020) (Hamilton, 2020) (Shotton, 2013) (Känkänen & Bardy, 2014) (Malik, 2005) (Hughes, 2013) (Gutsche, 2013) (BASW, 2020) | It often opened up lines of communication, sometimes leading to conversations, where other positive memories were triggered or where the child felt able to talk about sensitive issues.' (Shotton, 2010)<br><br>'Where adopted CYP were able to discuss their life stories with their parents in nonthreatening, everyday ways, feelings of relief and connection with parents were expressed.' (Watson, Staples, et al., 2020) |
| **CMOC13** | When caring adults are prepared to share personal information with the adolescent they are caring for (C) the adolescent is more willing to confide with their caring adult (O) because trust is established (M). | (Eldridge, 2018) (Watson, Staples, et al., 2020) | 'Furthermore, in the current study, limited reciprocal confiding and self-disclosure appeared to help equalise power dynamics and facilitate genuine confiding across personal and professional relationships.' (Eldridge, 2018) |
| **CMOC14** | When adolescents have consistent and repeated positive engagements with caring adults who support them to preserve and reflect on everyday life experiences (C) then they may start to build trust in others (O) because it challenges their previously held negative relational beliefs (M). | (Finlay, 2022) (Eldridge, 2018) (Wood & Selwyn, 2017) (Atwool, 2017) (Hamilton, 2020) (Hooley et al., 2016) (Shotton, 2012) (Furnivall & Grant, 2014) | 'Reciprocity, understanding, acceptance and perseverance all appeared to be important for the emergence of sufficient trust to confide. For some young people, trust developed in one relationship then appeared to generalise into other relationships' (Eldridge, 2018)<br><br>'Such work also enables the child to share their story, has the potential to increase self-esteem and, by building a sense of trust with the social worker and caregivers, may facilitate the development of secure |



For peer review only - http://bmjopen.bmj.com/site/about/guidelines.xhtml

Improving the mental health and mental health support available to adolescents in out-of-home care via Adolescent-Focused Low-Intensity Life Story Work: A realist review

| | | | |
|---|---|---|---|
| | | | attachment in new situations.' (Atwool, 2017) |
| **CMOC15** | When a caring adult provides consistent support to an adolescent to discuss their thoughts and feelings in response to everyday life experiences (C) the adolescent comes to understand their experiences and themselves better, including their emotional and behavioural responses to experiences (O) because they develop reflective and reminiscence skills through shared story telling (M). | (Ward, 2002) (Hamilton, 2020) (Hooley et al., 2016) (Hooley, 2015) (Fitzhardinge, 2008) (Steenbakkers et al., 2016) (Watson et al., 2018) (Haight et al., 2010) (Holody & Mäher, 1996) (Watson et al., 2015b) (Malik, 2005) (Shotton, 2013) (Ferrier, 2011) | 'Reflective function can be encouraged by bringing into consciousness a range of possible interpretations or ways of experiencing similar stories. It has been argued that both reflective function and neural integration develop only in a context of something akin to a secure attachment relationship.' (Fitzhardinge, 2008)<br><br>"This life book focuses on the here and now and makes use of the relationship between the worker and the child. While not specifically therapeutic in its goals, it seeks to help the child make connections between his or her present experiences, emotional needs, and past events." (Holody & Mäher, 1996)<br><br>'In this model, the key aims of the LSW are to integrate a child's internal and external experiences by collaboratively constructing a narrative.' (Hooley et al., 2016) |
| **CMOC16** | When a caring adult provides consistent support to an adolescent to discuss their thoughts and feelings in response to everyday life experiences (C) they experience better mental | (NSPCC, 2022) (Hooley et al., 2016) (Willis & Holland, 2009) (Känkänen & Bardy, 2014) (Steenbakkers et al., 2016) (Hills, 2022) (Furnivall & Grant, 2014) (Aventin et al., 2014) (Holody & Mäher, 1996) (Eldridge, 2018) | 'A universal viewpoint of exploring and managing emotions emerged, clearly suggesting the importance of helping children to identify, express and regulate emotions during their involvement' (Hooley et al., 2016)<br><br>'respondents indicated that good life story |



For peer review only - http://bmjopen.bmj.com/site/about/guidelines.xhtml

Improving the mental health and mental health support available to adolescents in out-of-home care via Adolescent-Focused Low-Intensity Life Story Work: A realist review

| | | | |
|---|---|---|---|
| | health (O) because they learn emotion regulation skills (M). | | work should involve helping a child to express and manage emotions that arise during the work.' (Hooley et al., 2016) |
| **CMOC17** | When caring adults consistently support an adolescent to preserve and reflect on everyday life experiences (C) it strengthens the relationship between them (O) because the caring adult becomes better attuned to understanding the adolescent and their support needs (M). | (Davies & Hodges, 2017) (Malik, 2005) (Shotton, 2012) (Holody & Mäher, 1996) (Atwool, 2017) (Hooley, 2015) (Hanna, 2007) | 'But by this stage, the carer was more attuned to Katie's particular experiences and was able to demonstrate the necessary acceptance, understanding, commitment and unconditional love for Katie that soothed and ameliorated her anxieties'. (Davies & Hodges, 2017)<br><br>'it gives the worker information critical to understanding and linking the child's present functioning and coping patterns with previous events in the child's life.' (Holody & Mäher, 1996)<br><br>'The here-and-now approach allowed Helen's mother to accept her child's thoughts and feelings as the girl's own perception and understanding of her life.' (Holody & Mäher, 1996) |
| **CMOC18** | When caring adults view everyday interactions with an adolescent as providing reflective opportunities (C) their relationship is strengthened (O) because adolescents are more comfortable | (Ward, 2002) (Finlay, 2022) (Hammond, 2016) (Connor et al., 1985) (Hamilton, 2020) (Aventin et al., 2014) (Känkänen & Bardy, 2014) | 'The keyworker also had to find time for spontaneous 'out of hours' sessions, such as helping Ben write short stories about his temper tantrums after they happened.' (Connor et al., 1985)<br><br>'You can use your very being to embrace the |



For peer review only - http://bmjopen.bmj.com/site/about/guidelines.xhtml

Improving the mental health and mental health support available to adolescents in out-of-home care via Adolescent-Focused Low-Intensity Life Story Work: A realist review

| | | | |
|---|---|---|---|
| | talking and reflecting on everyday events (M). | | relationships and create key opportunities for exploring each life story. 'Don't wait for the perfect moment, take the moment and make it perfect' (Anonymous).' (Hamilton, 2020) |
| **CMOC19** | When low-intensity LSW takes place in emotionally meaningful places (C) richer narratives can be constructed (O) because of the availability of contextual cues (M). | (Hills, 2022) (Haight et al., 2010) (Hammond, 2012) (Hamilton, 2020) (Watson, Staples, et al., 2020) | 'Providing an intervention to children within a context familiar and emotionally meaningful to them has a variety of other benefits as well. When the clinician travels to see them individually and on their own turf, children can feel valued and cared for, and relationship building may be enhanced. In addition, the community context is rich in cues for children to spontaneously initiate personal narratives.' (Haight et al., 2010) |
| **CMOC20** | When everyday life experiences are preserved using temporal anchor points and reflected on at regular intervals (C) it aids the ease of storytelling (O) because it is easier to follow (M). | (Hammond et al., 2021) (Hammond, 2012) (Gustavsson & MacEachron, 2008) (NICE, 2021) | 'My regular weekly visits also provided young people with a frame of reference from which to begin to construct and reflect upon recent events in a temporal fashion.' (Hammond, 2012)<br><br>'Schedule regular, dedicated times for life story work to help the looked-after child or young person make sense of their journey through the care system and beyond, their significant relationships and their identity.' (NICE, 2021) |



For peer review only - http://bmjopen.bmj.com/site/about/guidelines.xhtml

Improving the mental health and mental health support available to adolescents in out-of-home care via Adolescent-Focused Low-Intensity Life Story Work: A realist review

| | | | |
|---|---|---|---|
| **CMOC21** | When everyday life experiences are preserved and reflected on at regular intervals (C) adolescents become more comfortable engaging in the process (O) because the approach becomes familiar and predictable (M). | (Holody & Mäher, 1996) (Hamilton, 2020) (Shotton, 2012) | 'The here-and-now lifebook is more user-friendly than the traditional lifebook, not only for the child but for the worker as well. If it is used in every contact between worker and child, it is transformed from something extra into the very forum for interaction.'(Holody & Mäher, 1996) |
| **CMOC22** | When a caring adult regularly demonstrates how to respond to challenging situations in constructive ways (C) the adolescent learns how to respond to challenging situations (O) because of role modelling (M). | (Furnivall & Grant, 2014) (Hammond & Cooper, 2013) (Hammond, 2016) (Finlay, 2022) (Steenbakkers et al., 2016) | Developing the capacity to self-regulate and become accountable requires safe, positive relationships with adults who can scaffold children's' learning (Furnivall & Grant, 2014)<br><br>'This person also needs to be a role model, particularly when handling the inherent frustrations of working with digital media in innovative ways and helping to mediate the risks that digital media may bring (discussed later in this chapter).' (Hammond, 2016)<br><br>'In addition, negative experiences with (previous) attachment figures make sharing distressing memories more difficult for adolescents in family foster care, for example when these attachment figures insufficiently regulated the emotional aspects of distressing events for them, or insufficiently scaffolded them in constructing these memories' (Steenbakkers et al., 2016) |



For peer review only - http://bmjopen.bmj.com/site/about/guidelines.xhtml

Improving the mental health and mental health support available to adolescents in out-of-home care via Adolescent-Focused Low-Intensity Life Story Work: A realist review

| | | | |
|---|---|---|---|
| **CMOC23** | When a caring adult consistently and sensitively helps an adolescent curate, develop, and reflect upon narratives about their lived experiences (C) the narratives become authentic accounts that resonate with the adolescent's experience (O) because they were actively involved in the construction of accounts (M) | (Atwool, 2017) (Baynes, 2008) (Watson, Hahn, et al., 2020) (Watson, Staples, et al., 2020) | 'This will help to ensure that the written story reflects the child's experiences, helping to create a coherent narrative of events, feelings and memories. Without this, we risk creating a life story book that has little meaning to the child,' (Baynes, 2008) |
| **CMOC24** | When adolescents are not actively involved in the construction of narratives about their lived experience (C) they can struggle to accept the narratives as their own story (O) because they do not resonate with the young persons lived experience (M). | (Baynes, 2008) (Watson, Hahn, et al., 2020) (Gallagher & Green, 2012) (Atwool, 2017) | 'Children may feel that their life story book lacks a coherent narrative or presents a narrative with which they disagree (Watson et al., 2015a). This can lead to dissonance between their identity and the identity presented in the book, leading children to believe that including multiple perspectives would be beneficial (Watson et al., 2015a).' (Watson, Hahn, et al., 2020)<br><br>'All proponents of life story work emphasise the importance of working with the child and allowing them to give voice to their subjective experience. Kagan (2014) warns of the dangers of practitioners writing life stories for children, arguing that their validity is likely to be questioned because they do not fully capture the child's experience or perspective and traumatic events may be glossed over.' |



For peer review only - http://bmjopen.bmj.com/site/about/guidelines.xhtml

Improving the mental health and mental health support available to adolescents in out-of-home care via Adolescent-Focused Low-Intensity Life Story Work: A realist review

| | | | |
|---|---|---|---|
| | | | (Atwool, 2017) |
| **CMOC25** | When caring adults do not consistently and sensitively help adolescents curate, develop, and reflect upon narratives about their lived experiences (C) identity development can be negatively impacted (O) because the adolescent constructs unnecessarily negative narrative accounts (M). | (Watson, Hahn, et al., 2020) (Shotton, 2012) (Atwool, 2017) (Watson, Staples, et al., 2020) | 'Granting children control over narratives aids identity formation but left unchallenged their perspective of events may also become one-sided and inaccurate' (Watson, Hahn, et al., 2020) |
| **CMOC26** | When a caring adult supports an adolescent to construct narrative accounts of their experience using alternative information or interpretations (C), less negative narratives may result (O), because they have more perspectives to draw on (M) | (Fitzhardinge, 2008) (Shotton, 2012) (Davies & Hodges, 2017) (Pakrosnis & Cˇepukiene, 2011) (Hooley et al., 2016) (Baynes, 2008) (Shotton, 2013) (Gallagher and Green, 2012) (Shotton, 2010) (Willis and Holland, 2009) | 'Narratives were enriched by bringing into consciousness some alternative interpretations and accommodating in a deeper way the perspectives of different people within the story.' (Fitzhardinge, 2008)<br><br>'From a narrative perspective it can help the child to thicken positive counter-narratives impacting on the dominant stories the child has and is able to tell about themselves, particularly thickening stories around their strengths, worth and belonging' (Shotton, 2012)<br><br>Participants also highlighted the importance of finding out what the events mean to the child and offering alternative narratives:' (Hooley et al., 2016) |
| **CMOC27** | When a caring adult consistently and sensitively helps an | (Connor et al., 1985) (Hammond & Cooper, 2013) (Finlay, 2022) | The therapeutic process involves creating opportunities to open up conversational |



For peer review only - http://bmjopen.bmj.com/site/about/guidelines.xhtml

Improving the mental health and mental health support available to adolescents in out-of-home care via Adolescent-Focused Low-Intensity Life Story Work: A realist review

| | | | |
|---|---|---|---|
| | adolescent curate, develop, and reflect upon narratives about their lived experiences (C) positive narratives including liberating narratives can be constructed (O) because caring adults can help adolescents to re-script negative narrative accounts (M). | (Hammond, 2016) (Hammond, 2012) (Fitzhardinge, 2008) (Gutsche, 2013) (Happer et al., 2006) (Hooley, 2015) (Känkänen & Bardy, 2014) (Cook-Cottone & Beck, 2007) (Watson et al., 2015b) (Holody & Mäher, 1996) (Shotton, 2013) (Hooley et al., 2016) | spaces. These spaces provide young people with opportunities to share accounts of their own lives, in their own words. These accounts are listened to by a consistently available adult who can sensitively support the young person's reflections on the stories they choose to share, and potentially, provide re-scripting or re-editing advice.' (Hammond, 2016).<br><br>'Importantly, these reflections must eradicate self-blame and encourage young people to look forward positively towards their futures. This phase is about encouraging young people to recognise their own resilience, effectively turning 'if only this hadn't happened' into 'despite all these things that have happened to me'. (Hammond & Cooper, 2013) |
| **CMOC28** | When significant individuals provide alternative positively framed narratives to the adolescent (C) it helps to increase their self-esteem and ability to develop positive future expectations (O) because they become increasingly aware of their positive characteristics and abilities (M). | (Shotton, 2012) (Aust, 1981) (Hooley et al., 2016) (Teodorczuk et al., 2018) (Eldridge, 2018) (Happer et al., 2006) (Sanders, 2020) (Devenney, 2017) | 'From a narrative perspective it can help the child to thicken positive counter-narratives impacting on the dominant stories the child has and is able to tell about themselves, particularly thickening stories around their strengths, worth and belonging.' (Shotton, 2012)<br><br>'The co-construction of stories around their achievements and certificates contributes to |



For peer review only - http://bmjopen.bmj.com/site/about/guidelines.xhtml

Improving the mental health and mental health support available to adolescents in out-of-home care via Adolescent-Focused Low-Intensity Life Story Work: A realist review

| | | | |
|---|---|---|---|
| | | | their perception of themselves as someone who can achieve. As they reflect with their carer and look together at their writing and spelling they are able to co-construct stories about their progress over time and see themselves as someone who can make progress, who can grow and change in positive ways.' (Shotton, 2012) |
| **CMOC29** | When significant individuals are given the opportunity to offer their narrative account of adolescent's life experiences (C) adolescents become aware of alternative interpretations of life events (O) because they have access to different narrative accounts (M). | (Bazalgette, 2015) (HCC, 2022) (Hills, 2022) (Beste & Richardson, 1981) (Baynes, 2008) (Peake, 2009) (Ferrier, 2011) (BASW, 2020) (ARCBOX, 2022) (Fitzhardinge, 2008) (Watson, Hahn, et al., 2020) (Hoyle et al., 2020) (Watson et al., 2015a) (Watts, 2021) | 'Fourth, Life Story Books that include contributions from more than one source, such as foster parents, social worker, and parents, will allow children to have a broader understanding of their life events. It will also increase the child's awareness that different people see the same situation differently.' (Beste & Richardson, 1981)<br><br>'Children were consistently clear that the narrative presented in their book should be of their biography, representing multiple actor viewpoints including those of their birth family.' (Watson et al., 2015a)<br><br>'In addition, a life story book that contains many voices is likely to be seen by the child as having more credibility in the future'' (Baynes, 2008) |
| **CMOC30** | When significant individuals including birth relatives are | (Cook-Cottone & Beck, 2007) (Shotton, 2012) (Buchanan, 2014) | ''The life-story work can also be used as a way to structure visits with biological parents |



For peer review only - http://bmjopen.bmj.com/site/about/guidelines.xhtml

Improving the mental health and mental health support available to adolescents in out-of-home care via Adolescent-Focused Low-Intensity Life Story Work: A realist review

| | | | |
|---|---|---|---|
| | positively involved in Low intensity LSW (C) stronger relationships can be established between those involved (O) because of the interactions facilitated through the activity (M). | (Watson, Staples, et al., 2020) (Hooley, 2015) (Shotton, 2012) | and siblings. The visit time can be used to collect artifacts (take photos or share old photos) and record related information (e.g. write letters, create a memory page for photos, tell stories about the photos).' (Cook-Cottone & Beck, 2007)<br><br>'facilitating interaction with other family members, bringing the family closer together, helping other family members feel involved in the care of the child' (Shotton, 2012) |
| **CMOC31** | When significant individuals including birth relatives are involved in low intensity LSW (C) identity development can be supported (O) because significant individuals can share information about the adolescent's family heritage (M). | (Monson et al., 2020) (Neil & Beek, 2020) (Buchanan, 2014) (Hoyle et al., 2020) (Sanders, 2020) | 'For young people, carers and workers could also be gatekeepers for connectedness with cultural heritage and identity. One young person described what he believed was a turning point in a relationship with a carer, when the carer took him to a restaurant serving the cuisine of the young person's country of origin' (Monson et al., 2020)<br><br>'Staying in touch with parents, siblings and other important people helps children develop a sense of identity and belonging and promotes healthy and stable relationships,'(Sanders, 2020) |
| **CMOC32** | When adolescents exert a choice over how low intensity LSW everyday life experiences are preserved and reflected on (C) they experience ownership over the process and resulting | (Hooley, 2015) (Watson et al., 2018) (Buchanan, 2014) (Hammond & Cooper, 2013) (Watson, Hahn, et al., 2020) (Holody & Mäher, 1996) (NICE, 2021) (HCC, 2022) (Hammond, 2012) (Peake, 2009) | 'Children can have ownership of their story work via choosing which objects to story/not story and by dictating the pace at which the work progresses:<br>I think it's a great concept and would be a good idea for children to have ownership of |



For peer review only - http://bmjopen.bmj.com/site/about/guidelines.xhtml

Improving the mental health and mental health support available to adolescents in out-of-home care via Adolescent-Focused Low-Intensity Life Story Work: A realist review

| | | | |
|---|---|---|---|
| | products (O) because they feel valued their perspectives and opinions are valued. | (Gutsche, 2013) (Buchanan, 2014) (Lucas et al., 2020) (Baynes, 2008) | their stories and memories. (FSW, Ben)' (Watson, Hahn, et al., 2020)<br><br>'A feeling of ownership appeared to be fostered through the use of a person-centred and flexible approach to LSW.' (Buchanan, 2014) |
| **CMOC33** | When adolescents are given agency to choose the mediums (digital, paints, puppets, physical/digital 'visits' music etc) used to collect and conserve everyday memories (C) it is engaging to them (O) because they have a sense of control, ownership and power over the process, (M). | (Watson, Hahn, et al., 2020) (Hammond, 2012) (Hammond, 2016) (Watson et al., 2015a) (Aventin et al., 2014) (Hills, 2022) (Buchanan, 2014) (Holody & Mäher, 1996) (Hammond & Cooper, 2013) (Nuffield Family Justice Observatory, 2021) (Baynes, 2008) (Hooley et al., 2016) (Hammond et al., 2021) (Lucas et al., 2020) (Peake, 2009) (Willis & Holland, 2009) (Känkänen & Bardy, 2014) (Gutsche, 2013) (Hammond & Cooper, 2013) | 'trove aims to support children to keep their own record of their life, through interactive child-driven technology, to give them some control of their life story.' (Watson, Hahn, et al., 2020)<br><br>'…how we can support that so it feels like their story rather than produced in a format that somebody has given them. We want to make sure that for young people, their stories are owned by them, and that means having them in a format that they would choose.' (Hills, 2022) |
| **CMOC34** | When caring adults are aware of the adolescent's special educational needs and disabilities (SEND) (C) the adolescent can engage with preserving and reflecting on their experiences (O) because appropriate adaptations | (Hamilton, 2020) (Hammond, 2012) (HCC, 2022) (Atwell, 2016) (NICE, 2021) | 'Where the child has some level of learning disability, careful thought will need to be given to the implications this will have on undertaking life story work, considering in particular the most effective methods of communication.' (HCC, 2022) |



For peer review only - http://bmjopen.bmj.com/site/about/guidelines.xhtml

Improving the mental health and mental health support available to adolescents in out-of-home care via Adolescent-Focused Low-Intensity Life Story Work: A realist review

| | | | |
|---|---|---|---|
| | can be made to make the activity accessible (M). | | 'For children with a disability who perhaps have difficulty in communicating by speech, or who may lack the use of one or more of the senses, it is possible to develop life story work that does not depend only on visual methods but involves stimulation of other senses.' (Atwell, 2016)<br><br>'Take a flexible approach to life story work, and tailor it to the developmental age and needs of the looked-after child or young person.' (NICE, 2021) |
| **CMOC35** | When caring adults from the adolescent's support network have training materials available to them (C) adolescents with SEND can engage with preserving and reflecting on their lived experiences (O) because the caring adults in their support network are equipped with the knowledge and skill to make appropriate adaptations (M). | (Atwell, 2016) | 'In deciding how to tackle life story work with disabled children, it is also important to consider who would be best able to do this work. Since being able to understand and communicate with the child is paramount, this should be undertaken by the person who communicates best with the child, rather than assume it is always done by the child's social worker.' (Atwell, 2016) |
| **CMOC36** | When the creation of a product is prioritised over a meaningful process for securing everyday memories (C) the value of narratives contained will be | (Känkänen & Bardy, 2014) (Hammond & Cooper, 2013) (Hooley et al., 2016) (Baynes, 2008) (Hammond, 2012) | 'Baynes (2008) continues by suggesting that life story work has been pushed to the periphery of practice within a more quantitatively driven target-focused profession. She fears that this may lead some |



For peer review only - http://bmjopen.bmj.com/site/about/guidelines.xhtml

Improving the mental health and mental health support available to adolescents in out-of-home care via Adolescent-Focused Low-Intensity Life Story Work: A realist review

| | | | |
|---|---|---|---|
| | reduced (O) because the product does not represent adolescents lived experience of everyday events (M). | | to conceptualise life story work as a product, which could detract from the potentially therapeutic nature of the underlying process.' (Hammond, 2012)<br><br>'They also note that although the process is of primary importance, the material record was also significant.' (Atwool, 2017) |
| **CMOC37** | When the process of curating, developing, and reflecting upon narratives about lived experiences is prioritised over the recording (C) a bank of future reflective starting points is lost (O) because everyday memories and artefacts are not secured (M). | (Aust, 1981) (Walker & Ryan, 2016a) (Hoyle et al., 2020) (Hooley, 2015) (Watson et al., 2015b) (Hills, 2022) (Watts, 2021) (Buchanan, 2014) (Känkänen & Bardy, 2014) (Atwool, 2017) (Willis & Holland, 2009) (Beste & Richardson, 1981) (Aust, 1981) (Hammond, 2016) | 'We never regard the work as finished as life goes on, but some record of the process is important as it provides a reference point, particularly as it can be updated until adulthood.' (Walker & Ryan, 2016a)<br><br>'Material items and the records produced during the work were important to the young people. Many described treasuring them, returning to them often and planning to continue to add to them.' (Buchanan, 2014)<br><br>'Concrete artefacts – such as poems, photographs, paintings, cartoons and so on – cannot be ignored, which also means that unlike an unrecorded spoken narrative, they persist after the initial act of telling and we can return to them again and again.' (Känkänen & Bardy, 2014) |
| **CMOC38** | When adolescents feel empowered to have editorial | (Baynes, 2008) (Watson, Hahn, et al., 2020) (Watson et al., 2018) | 'Children can have ownership of their story work via choosing which objects to story/not |



For peer review only - http://bmjopen.bmj.com/site/about/guidelines.xhtml

Improving the mental health and mental health support available to adolescents in out-of-home care via Adolescent-Focused Low-Intensity Life Story Work: A realist review

| | | | |
|---|---|---|---|
| | control over memory product(s) (C) they feel more positive about the product(s) and experience greater ownership of their story/narrative (O) because they have control over how, and which, events are recorded or not (M). | (Hoyle et al., 2020; Watson et al., 2015a) (Buchanan, 2014) (Gallagher & Green, 2012) (Gray et al., 2019) | story and by dictating the pace at which the work progresses' (Watson, Hahn, et al., 2020)<br><br>'In some cases children were dismissive about their book, as the focus did not seem to be on them specifically. For a few children, photos included were stark reminders of differential treatment of siblings by birth parents and contributed to negative feelings.' (Watson et al., 2015a)<br><br>'have stuck a piece of paper over a section about me behaving inappropriately around Rhian and John, my adoptive family that broke down. The thing in the life story book spoilt my memory of Rhian and John's …. It seemed like it was written for somebody younger than me. (Caitlyn, 19)' (Gallagher & Green, 2012) |
| **CMOC39** | When the medium used for collecting the product is easily editable (e.g., digitally) (C) it helps the contents of low intensity LSW hold meaning over time (O) because it can be updated and edited when needed (M) | (Beste & Richardson, 1981) (Hammond, 2012) (Hammond & Cooper, 2013) (Watson et al., 2015a) (Watson, Latter and Below, 2015) (Atwool, 2017) (Hammond & Cooper, 2013) (Hooley et al., 2016) (Nuffield Family Justice Observatory, 2021) | 'Digital tools offer the ability to help young people to express thoughts and feelings which can be continually updated, edited and reflected upon. Using interactive computer-based mediums in this way grants the user flexibility to make changes frequently and easily.' (Hammond & Cooper, 2013)<br><br>'Concerns about the scrapbook style focused on the inability to remove information or to update as the child grew older. ' (Watson, |



For peer review only - http://bmjopen.bmj.com/site/about/guidelines.xhtml

Improving the mental health and mental health support available to adolescents in out-of-home care via Adolescent-Focused Low-Intensity Life Story Work: A realist review

| | | | |
|---|---|---|---|
| | | | Latter and Below, 2015) |
| **CMOC40** | When digital artefacts/products are backed-up and have access controls (e.g., encrypted and password protected) (C) it helps preserve and keep the contents safe (O) because it is secured and protected from unauthorised access (M). | (Hammond & Cooper, 2013) (Shepard, 2022) (Ferrier, 2011) (Watson et al., 2015a) (Atwool, 2017) (Willis & Holland, 2009) (Gustavsson & MacEachron, 2008) (Beste & Richardson, 1981) (Hammond & Cooper, 2013) (Hammond, 2016) (Ferrier, 2011) (Gray et al., 2019) | 'Security mechanisms were posited from both groups, including remotely programmable passcodes or a scannable key.' (Gray et al., 2019)<br><br>'Provision of back-up storage in case records are lost or destroyed is also needed.' (Atwool, 2017)<br><br>'Digital life story work products can be copied easily and, with permission, back-up copies can be stored with young people's files for posterity.' (Hammond, 2016) |
| **CMOC41** | When a medium for capturing a product enables narratives to be stored in different formats (e.g. voices, videos, pictures, objects) (C), they provide a richer starting point for future retelling (O), because they connect adolescents to their memories through the engaging and tangible nature of the artefacts (M). | (Hammond & Cooper, 2013) (Peake, 2009) (Hills, 2022) (Watson et al., 2015a) (Gray et al., 2019) (Watson et al., 2018) (Shotton, 2012) (Shotton, 2013) (Hammond, 2016) (Buchanan, 2014) (Atwell, 2016) (Hoyle et al., 2020) (Watson, Hahn, et al., 2020) | 'The background sounds to everyday life often go unnoticed. Sounds from the environment can help to connect to memories and hearing a specific sound can prompt a memory.' (Hammond & Cooper, 2013)<br><br>'For looked after and adopted children, physical objects are often the only remaining link to their past; a portal to stories of birth families, former homes, and significant people.' (Gray et al., 2019)<br><br>'In looking through the store both carers and children were able to flesh out each memory with details of when it occurred or who else |



For peer review only - http://bmjopen.bmj.com/site/about/guidelines.xhtml

Improving the mental health and mental health support available to adolescents in out-of-home care via Adolescent-Focused Low-Intensity Life Story Work: A realist review

| | | | |
|---|---|---|---|
| | | | was there. It was clear that the pictorial information was very powerful in taking the participants back to the time and place when it occurred.' (Shotton, 2013) |
| **CMOC42** | When adolescent's communication needs are considered when creating low-intensity LSW products (C) it ensures that memories are accessible for the adolescent to engage with (O) because they can understand the contents (M). | (Watson, Latter & Bellew, 2015) (Hammond, 2012) (Atwell, 2016) | "A lack of appropriate methods of communication in life story work may result in engagement opportunities with adolescents becoming lost and/or overlooked." (Hammond, 2012)<br><br>"…the care system and social work practice itself needs to converse using culturally appropriate methods of communication… ' (Hammond, 2012)<br><br>'For children with a disability who perhaps have difficulty in communicating by speech, or who may lack the use of one or more of the senses, it is possible to develop life story work that does not depend only on visual methods but involves stimulation of other senses.' (Atwell, 2016) |
| **CMOC43** | When adolescents use LI-LSW products as storytelling prompts to share stories with chosen caring adults (C) it helps to strengthen relationships between adolescents | (Buchanan, 2014) (Shotton, 2012) (Hammond, 2012) (Hamilton, 2020) | 'Sharing a moment, an experience together helps to create social connections and helps us understand one another and our thinking.' (Hamilton, 2020) |



For peer review only - http://bmjopen.bmj.com/site/about/guidelines.xhtml

Improving the mental health and mental health support available to adolescents in out-of-home care via Adolescent-Focused Low-Intensity Life Story Work: A realist review

| | | | |
|---|---|---|---|
| | and caring adults (O) because caring adults understand adolescents and their experiences (M) | | |
| **CMOC44** | When current everyday life experiences are preserved and reflected on (C) positive self-narratives can become more accessible for the adolescent to make sense of their experiences, construct their identity and develop positive future expectations (O) because the focus is not on past negative experiences (M) | (Shotton, 2013) (Shotton, 2012) (Nicholls, 2003) (Teodorczuk et al., 2018) (Ferrier, 2011) (Haight et al., 2010) (Watson, Staples, et al., 2020) (Steenbakkers et al., 2016) (NSPCC, 2022) (Hammond et al., 2021) (Teodorczuk et al., 2018) | 'Why Life Story Work is Failing…It has the potential to deny the promotion of a child's identity by focusing on his or her experience as a looked-after child.' (Nicholls, 2003)<br><br>'From a narrative perspective it can help the child to thicken positive counter-narratives impacting positively on the dominant stories the child has and is able to tell about themselves, particularly thickening stories around their strengths, worth and belonging.' (Shotton, 2012)<br><br>'A key part of Life Story Work is celebrating the child or young person's achievements. We recognise the resilience they've shown by overcoming adversity and help them to see themselves in a positive light.' (NSPCC, 2022) |
| **CMOC45** | When everyday life experiences are reflected on between an adolescent and caring adult (C) difficulties and challenges can be positively re-framed (O) because | (Walker & Ryan, 2016a) (Holody & Mäher, 1996) (Buchanan, 2014) (Teodorczuk et al., 2018) (Hooley et al., 2016) | 'Life story books are thought to sometimes silence the difficulties experienced by adoptees by not allowing them enough space for exploring troublesome feelings and fantasies.' (Walker & Ryan, 2016a) |



For peer review only - http://bmjopen.bmj.com/site/about/guidelines.xhtml

Improving the mental health and mental health support available to adolescents in out-of-home care via Adolescent-Focused Low-Intensity Life Story Work: A realist review

| | | | |
|---|---|---|---|
| | opportunities to reflect on positive interpretations are taken (M). | | 'The statements relating to this all emphasised 'feelings to be shown, managed and normalised'. Participants in all groups agreed with the suggestion that work should not be stopped if difficult feelings came up and that upsetting or traumatic experiences should be explored. They indicated that a balance needed to be achieved that included happy as well as difficult memories.' (Hooley et al., 2016) |
| **CMOC46** | When low-intensity LSW containing a bank of positive memories and personal achievements is re-visited by an adolescent needing emotional support (C) their wellbeing may improve (O), because they find the contents comforting (M) | (Watts, 2021) (Shotton, 2010) (Buchanan, 2014) (Cook-Cottone & Beck, 2007; Gallagher & Green, 2012) (Shotton, 2012) (Shotton, 2013) (Humphreys & Kertesz, 2014) (Willis & Holland, 2009) (Watts, 2021) (Holody & Mäher, 1996) | 'The value of returning to the book was described by the other participants. Some chose to simply look through it to see 'familiar faces' or to remind them of coming through difficult times in the past when faced with new transitions. (Buchanan, 2014)<br><br>'As many of the memories were of happy/positive times, the carers felt that reflecting on the store often had a positive effect on mood for both themselves and the children.' (Shotton, 2012)<br><br>'The carers perceived that using the memory store approach often had a calming effect on the children and that it was also an activity that they seemed to find comforting. (Shotton, 2012) |



For peer review only - http://bmjopen.bmj.com/site/about/guidelines.xhtml

Improving the mental health and mental health support available to adolescents in out-of-home care via Adolescent-Focused Low-Intensity Life Story Work: A realist review

| | | | |
|---|---|---|---|
| **CMOC47** | When caring adults have an awareness of the adolescent's cultural background (C) it promotes appropriate preservation and reflection of everyday life experiences (O) because caring adults have an increased awareness of the adolescent's individuality and cultural heritage (M). | (Monson et al., 2020) (HCC, 2022) (Walker & Ryan, 2016b) (NICE, 2021) | 'It is important to a child that the worker doing life story work has a good grasp of the child's world, both the inner world and external realities. This means the worker must familiarise him or herself with aspects of family life of the ethnic community of the child in the context of this society. This involves getting information from a variety of sources, for example, people from a culture similar to that of the child, and of course the internet.' (Walker & Ryan, 2016b)<br><br>'Ensure that life story work for looked-after children and young people captures and embraces ethnicity, cultural and religious identity, as well as other personal aspects of identity, for example, sexual identity or disabilities.' (NICE, 2021) |
| **CMOC48** | When institutions consistently communicate and take measures to support the importance of collecting everyday life experiences for adolescents (C) significant individuals are more likely to prioritise this activity (O) because they know what is expected of them (M). | (Finlay, 2022) (Wood, 2019) (Atwool, 2017) (Holody & Mäher, 1996) (Shotton, 2013) (Watts, 2021) (Hooley et al., 2016) (Connor et al., 1985) (Brookfield et al., 2008) | 'Recognition of children's entitlement to a coherent narrative needs to be embedded in practice at the micro level. This can only occur if appropriate supports are in place at the organisational level, facilitated by macro-level priority being accorded to provision of resources needed to ensure the best possible outcomes for children and young people in care.' (Atwool, 2017)<br><br>''Policy needs to be developed which specifies that training for foster carers in |



For peer review only - http://bmjopen.bmj.com/site/about/guidelines.xhtml

Improving the mental health and mental health support available to adolescents in out-of-home care via Adolescent-Focused Low-Intensity Life Story Work: A realist review

| | | | |
|---|---|---|---|
| | | | using the approach, or similar, should be part of their induction to fostering. This would raise their awareness and help them feel equipped to use the approach from the start of a child's placement with them.' (Shotton, 2013) |
| **CMOC49** | When caring adults are provided with relevant ongoing training that highlights the importance of preserving and reflecting on everyday life experiences (C), they are more likely to carry these out (O) because they have the confidence and skills to do so (M). | (Finlay, 2022) (Braiden, 2016) (Walker & Ryan, 2016a) (Eenshuistra et al., 2019) (Hills, 2022) (Haight et al., 2010) (Wood, 2019) (Watson, Staples, et al., 2020) (NICE, 2021) (Shotton, 2012) (Buchanan, 2014) (Holody & Mäher, 1996) (Shotton, 2013) (Atwool, 2017) (Sanders, 2020) | 'To embark on this journey with the child, above all you need time, sensitivity, empathy for the child and a commitment to the work. An ability to listen to the child and understand them is paramount; the skills and particular techniques can be learnt' (Walker & Ryan, 2016a)<br><br>'Ensure that the experience and skillset of the practitioner or carer delivering life story work for looked-after children and young people is sufficient to deliver good quality work, particularly in complex situations.' (NICE, 2021) |
| **CMOC50** | When peers with similar lived experiences introduce low-intensity LSW to an adolescent (C) their engagement with low-intensity LSW increases (O) because they find advice from peers more credible and authentic (M). | (Holody & Mäher, 1996) (Hughes, 2013; Lucas et al., 2020) (Fitzhardinge, 2008) | 'James was able to show his lifebook to another foster child who was resistant to beginning this work.' (Holody & Mäher, 1996)<br><br>'The use of published story books which describe similar scenarios of children who are in similar circumstances - such as where the child in the story is also blaming themselves about their parents' separation - can be very helpful.' (Hughes, 2013) |



For peer review only - http://bmjopen.bmj.com/site/about/guidelines.xhtml

Improving the mental health and mental health support available to adolescents in out-of-home care via Adolescent-Focused Low-Intensity Life Story Work: A realist review

| | | | |
|---|---|---|---|
| **CMOC51** | Caring adults who are supporting low-intensity LSW should have access to regular supervision (C) this helps the caring adult feel prepared and confident to support low-intensity LSW and improves their practice (O) because they have support and guidance available to reinforce training and a self-reflective space to process emotions (M). | (Walker & Ryan, 2016a) (Braiden, 2016) (Hooley et al., 2016) (Eenshuistra et al., 2019) (Haight et al., 2010) (Furnivall & Grant, 2014) (Buchanan, 2014) (Connor et al., 1985) (Watson, Hahn, et al., 2020) (Holody & Mäher, 1996) (Peake, 2009) | ‘Unless training is reinforced by regular supervision and consultancy and embedded within a trauma sensitive organisational system, it will be unable to change children’s experience significantly.’ (Furnivall & Grant, 2014)<br><br>‘Clear areas for support and consultation that could be provided by clinical psychologists and social care professionals have been highlighted together with areas for future research.’ (Hooley et al., 2016)<br><br>‘Any worker undertaking life story work requires not only a reasonable knowledge of child development but also access to other workers in this field who can be called upon to give advice and support in the way that we used the clinical psychologist.’ (Connor et al., 1985) |



For peer review only - http://bmjopen.bmj.com/site/about/guidelines.xhtml

Improving the mental health and mental health support available to adolescents in out-of-home care via Adolescent-Focused Low-Intensity Life Story Work: A realist review

References

ARCBOX. (2022). *Digital Life Story ARCBOX* ARC Adoption North East. https://www.arcadoptionne.org.uk/life-story

Atwell, A. (2016). Working with children with a disability. In R. Walker & T. Ryan (Eds.), *Life Story Work why, what, how and when* (pp. 97-102). CoramBAAF Adoption and Fostering Academy.

Atwool, N. (2017). Life story work: Optional extra or fundamental entitlement? *Child Care in Practice*, *23*(1), 64-76. https://www.tandfonline.com/doi/full/10.1080/13575279.2015.1126228

Aust, P. H. (1981). Using the Life Story Book in Treatment of Children in Placement. *Child Welfare*, *60*(8), 535-536,553-560. https://www.jstor.org/stable/45393827

Aventin, A., Houston, S., & Macdonald, G. (2014). Utilising a computer game as a therapeutic intervention for youth in residential care: Some preliminary findings on use and acceptability. *Children and Youth Services Review*, *47*, 362-369. http://ovidsp.ovid.com/ovidweb.cgi?T=JS&PAGE=reference&D=psyc11&NEWS=N&AN=2014-55927-021

BASW. (2020). *Recording in childrens social work*. https://www.basw.co.uk/system/files/resources/basw_recording_in_childrens_social_work_aug_2020.pdf

Baynes, P. (2008). Untold stories: A discussion of life story work. *Adoption & Fostering*, *32*(2), 43-49. https://doi.org/10.1177%2F030857590803200206

Bazalgette, T. R., G. Trevelyan. (2015). Achieving emotional wellbeing for looked after children a whole system approach. .

Beste, H. M., & Richardson, R. G. (1981). Developing a Life Story Book Program for Foster Children. *Child Welfare*, *60*(8), 529-534. https://www.jstor.org/stable/45393826

Bolton, C. (2022). *Life Story Work 5 minute facts* https://www.bolton.gov.uk/downloads/file/2299/life-story-work

Braiden, S. (2016). Evaluation of a short training programme for foster carers. *Scottish Journal of Residential Child Care*, *15*(2). https://www.celcis.org/application/files/2016/2308/5288/004._2016_Vol_15_2_Braiden_Evaluation_for_foster_carers.pdf

Brookfield, H., Brown, S. D., & Reavey, P. (2008). Vicarious and post-memory practices in adopting families: The re-production of the past through photography and narrative. *Journal of Community & Applied Social Psychology*, *18*(5), 474-491. https://onlinelibrary.wiley.com/doi/abs/10.1002/casp.960

Buchanan, A. (2014). *The experience of life story work: reflections of young people leaving care* Cardiff University]. EThOS. https://orca.cardiff.ac.uk/64531/

Connor, T., Sclare, I., Dunbar, D., & Elliffe, J. (1985). Making a life story book. *Adoption & Fostering*, *9*(2), 32-46. https://dx.doi.org/10.1177/030857598500900210

Cook-Cottone, & Beck. (2007). A Model for Life-Story Work: Facilitating the Construction of Personal Narrative for Foster Children. *Child Adolesc Ment Health*, *12*(4), 193-195. https://doi.org/10.1111/j.1475-3588.2007.00446.x



For peer review only - http://bmjopen.bmj.com/site/about/guidelines.xhtml

Improving the mental health and mental health support available to adolescents in out-of-home care via Adolescent-Focused Low-Intensity Life Story Work: A realist review


Davies, M., & Hodges, J. (2017). Relationship renaissance: the use of attachment-based narrative and metaphor in life story work. *Adoption & Fostering*, *41*(2), 131-141. https://doi.org/10.1177%2F0308575917702831

Devenney, K. (2017). Pathway planning with unaccompanied young people leaving care: Biographical narratives of past, present, and future. *Child & Family Social Work*, *22*(3), 1313-1321. https://onlinelibrary.wiley.com/doi/full/10.1111/cfs.12348

Eenshuistra, A., Harder, A. T., & Knorth, E. J. (2019). One size does not fit all: A systematic review of training outcomes on residential youth care professionals' skills. *Children and Youth Services Review*, *103*, 135-147. https://doi.org/10.1016/j.childyouth.2019.05.010

Eldridge, J. (2018). *Confiding in Others: A Qualitative Study Exploring the Experiences of Young people who have been in the Care System* University of Surrey Guildford].

Ferrier, M. (2011). Life story work in the context of attachment-led care planning. *Scottish Journal of Residential Child Care*, *10*(1). https://www.celcis.org/application/files/4516/2316/8859/2011_Vol_10_1_Ferrier_Life_story_work.pdf

Finlay, J. (2022). *Meaningful Natural Mentoring Relationship Characteristics and informal therapeutic life spcae interactions in youth care* Canterbury Christ Church University].

Fitzhardinge, H. (2008). Adoption, Resilience and the Importance of Stories: The Making of a Film about Teenage Adoptees. *Adoption & Fostering*, *32*(1), 58-68. https://doi.org/10.1177/030857590803200108

Furnivall, J., & Grant, E. (2014). *Trauma sensitive practice with children in care* (Insights: evidence summaries to support social services in Scotland, Issue. https://www.iriss.org.uk/sites/default/files/iriss-insight-27.pdf

Gallagher, B., & Green, A. (2012). In, out and after care: Young adults' views on their lives, as children, in a therapeutic residential establishment. *Children and Youth Services Review*, *34*(2), 437-450. https://dx.doi.org/10.1016/j.childyouth.2011.11.014

Gray, S., Hahn, R., Cater, K., Watson, D., Meineck, C., & Metcalfe, T. (2019). *Trove: A Digitally Enhanced Memory Box for Looked after and Adopted Children* Proceedings of the 18th ACM International Conference on Interaction Design and Children, https://www.researchgate.net/publication/333628573_Trove_A_Digitally_Enhanced_Memory_Box_for_Looked_after_and_Adopted_Children/link/5d31cbc4299bf1995b382ab3/download

Gustavsson, N., & MacEachron, A. (2008). Creating Foster Care Youth Biographies: A Role for the Internet. *Journal of Technology in Human Services*, *26*(1), 45-55. https://www.tandfonline.com/doi/abs/10.1300/J017v26n01_03

Gutsche, K. H. (2013). *Perceptions of social workers regarding life story work with children in child youth centres* North-West University].

Haight, W., Black, J., & Sheridan, K. (2010). A Mental Health Intervention for Rural, Foster Children from Methamphetamine-involved Families: Experimental Assessment with Qualitative Elaboration. *Child Youth Serv Rev*, *32*(10), 1146-1457. https://doi.org/10.1016/j.childyouth.2010.06.024

Hamilton, E. (2020). Life story approaches and relationships within residential child care: A practice reflection. *Relational Child and Youth Care Practice*, *33*(1),


For peer review only

Improving the mental health and mental health support available to adolescents in out-of-home care via Adolescent-Focused Low-Intensity Life Story Work: A realist review

87. https://www.celcis.org/application/files/8316/2307/7669/2019_Vol_18_No__2_Hamilton_E_A_practice_reflection.pdf

Hammond, S. (2012). *Exploring a role for digital technology in life story work with adolescents in residential care: a discourse analysis* University of East Anglia]. EThOS. https://ueaeprints.uea.ac.uk/id/eprint/39133/

Hammond, S. (2016). Digital Life Story Work in Action. In R. Walker & T. Ryan (Eds.), *Life story work Why, what, how and when* (pp. pp.106-119). CoramBAAF Adoption and Fostering Academy.

Hammond, S., & Cooper, N. (2013). *Digital Life Story Work: Using technology to help young people make sense of their experience.* (1 ed.). British Association of Adoption and Fostering.

Hammond, S. P., Cooper, N. J., & Jordan, P. (2021). Mental health, identity and informal education opportunities for adolescents with experience of living in state care: a role for digital storytelling. *Cambridge Journal of Education*, *51*(6), 713-732. https://doi.org/10.1080/0305764x.2021.1919057

Hanna, M. D. (2007). Preparing school age children for adoption: perspectives of successful adoptive parents and caseworkers. *Adoption Quarterly*, *10*(2), 1-32. https://www.tandfonline.com/doi/abs/10.1300/J145v10n02_01

Happer, H., McCreadie, J., & Aldgate, J. (2006). *Celebrating Success: What Helps Looked After Children Succeed*. Social Work Inspection Agency.

HCC. (2022). *Direct Work and Life Story Work with Children and Young People*. Hertfordshire County Council. Retrieved 12.08.22 from https://hertschildcare.proceduresonline.com/p_direct_life_story.html

Hills, L. (2022). In *Ensuring children's life stories are at the centre of direct work.*

Holody, R., & Mäher, S. (1996). Using lifebooks with children in family foster care a here and now process model. *Child Welfare*, *75*(4), 321-335.

Hooley, K., Stokes, L., & Combes, H. (2016). Life story work with looked after and adopted children: how professional training and experience determine perceptions of its value. *Adoption & Fostering*, *40*(3), 219-233. https://doi.org/10.1177%2F0308575916661129

Hooley, K. C. (2015). *Identifying perspectives on life story work with looked-after and adopted children* Staffordshire University]. EThOS. http://eprints.staffs.ac.uk/2244/

Hoyle, V., Shepherd, E., Lomas, E., & Flinn, A. (2020). Recordkeeping and the life-long memory and identity needs of care-experienced children and young people. *Child & Family Social Work*, *25*(4), 935-945. https://doi.org/https://doi.org/10.1111/cfs.12778

Hughes, J. I. (2013). Life-story work: a journey into a child's world. *Seen and Heard*, *23*(1), 23-30.

Humphreys, C., & Kertesz, M. (2014). Making Records Meaningful: Creating an Identity Resource for Young People in Care. *Australian Social Work*, *68*(4), 497-514. https://doi.org/10.1080/0312407x.2014.960434

Känkänen, P., & Bardy, M. (2014). Life stories and arts in child welfare: enriching communication. *Nordic Social Work Research*, *4*(1), 37-51. https://www.tandfonline.com/doi/full/10.1080/2156857X.2013.781536

Lucas, J., Matthews, L., Brady, K., Breguet, R., & Parson, J. (2020). *Therapeutic Life Story Work Barwon Pilot Program: Interim Report 2020* [Research report].



For peer review only - http://bmjopen.bmj.com/site/about/guidelines.xhtml

Improving the mental health and mental health support available to adolescents in out-of-home care via Adolescent-Focused Low-Intensity Life Story Work: A realist review


Malik, S. (2005). *Application of attachment theory for training foster parents: A model program* https://www.proquest.com/dissertations-theses/application-attachment-theory-training-foster/docview/305340219/

Monson, K., Moeller-Saxone, K., Humphreys, C., Harvey, C., & Herrman, H. (2020). Promoting mental health in out of home care in Australia. *Health Promot Int*, *35*(5), 1026-1036. https://doi.org/10.1093/heapro/daz090

Neil, E., & Beek, B. (2020). Resepecting Children's Relationships and Identities in Adoption. In *The Routledge Handbook of Adoption* (1st ed., pp. 77-89). Routledge.

NICE. (2021). *Looked after children and young people NICE guideline*. www.nice.org.uk/guidance/ng205

Nicholls, E. (2003). Model answer. *Community Care*(1479), 32-34. https://www.communitycare.co.uk/2003/07/03/model-answer/

NSPCC. (2022). *Life Story Work NSPCC Learning*. https://learning.nspcc.org.uk/services-children-families/life-story-work#heading-top

Nuffield Family Justice Observatory. (2021). *Modernising post-adoption contact: findings from a recent consultation*. https://www.nuffieldfjo.org.uk/wp-content/uploads/2021/08/nfjo_report_adoption_connections_20210913v2.pdf

Pakrosnis, R., & Cˇepukiene, V. (2011). Outcomes of Solution-Focused Brief Therapy for Adolescents in Foster Care and Health Care Settings. In C. Franklin, T. S. Trepper, E. E. McCollum, & W. J. Gingerich (Eds.), *Solution-Focused Brief Therapy: A Handbook of Evidence-Based Practice*. Oxford Scholarship Online.

Peake, A. (2009). Life Story Work. A resource for foster carers, residential social workers, adoptive parents, and kinship carers, to support this work. . In.

Sanders, R. (2020). *ESSS Outline: Care experienced children and young people's mental health*. https://doi.org/10.31583/esss.20201012

Shepard, E. (2022). *Good practice in record-keeping in children's social care*. Research in Practice. https://www.researchinpractice.org.uk/children/news-views/2022/february/good-practice-in-record-keeping-in-children-s-social-care/

Shotton, G. (2010). Telling different stories The experience of foster/adoptive carers in carrying out collaborative memory work with children. *Adoption & Fostering*, *34*(4), 61-68. https://doi.org/10.1177%2F030857591003400407

Shotton, G. (2012). *"Remember when...": exploring the experiences of looked after children and their carers in engaging in collaborative reminiscence* University of Newcastle upon Tyne]. EThOS. http://theses.ncl.ac.uk/jspui/handle/10443/1685

Shotton, G. (2013). 'Remember when...': exploring the experiences of looked after children and their carers in engaging in collaborative reminiscence. *Adoption & Fostering*, *37*(4), 352-367. https://doi.org/10.1177%2F0308575913508721

Steenbakkers, A., van der Steen, S., & Grietens, H. (2016). 'To talk or not to talk?': Foster youth's experiences of sharing stories about their past and being in foster care. *Children and Youth Services Review*, *71*, 2-9. https://doi.org/10.1016/j.childyouth.2016.10.008

Sunderland, F. (2021). Foster Carers Handbook. In.

Teodorczuk, K., Guse, T., & du Plessis, G. A. (2018). The effect of positive psychology interventions on hope and well-being of adolescents living in a




For peer review only - http://bmjopen.bmj.com/site/about/guidelines.xhtml

child and youth care centre. *British Journal of Guidance & Counselling*, *47*(2), 234-245. https://doi.org/10.1080/03069885.2018.1504880

Together for Children Sunderland. (2019). *Foster Carers Handbook April 2019*. https://www.togetherforchildren.org.uk/sites/default/files/2019-12/Foster%20Carer%20HandbookFinalDraft16052019%20-%20Updated%20April%202019.pdf

Walker, R., & Ryan, T. (2016a). Why do life story work? In R. Walker & T. Ryan (Eds.), *Life story work Why, what, how and when* (pp. 4-17). CoramBAAF Adoption and Fostering Academy.

Walker, R., & Ryan, T. (2016b). Working with black and minority ethnic children. In R. Walker & T. Ryan (Eds.), *Life story work Why, what, how and when* (pp. 77-84). CoramBAAF Adoption and Fostering Academy.

Ward, A. (2002). Opportunity led work maximising the possibilities for therapeutic communication in everyday interactions. *Therapeutic Communities*, *23*(2), 111-124.

Watson, Hahn, & Staines. (2020). Storying special objects: Material culture, narrative identity and life story work for children in care. *Qualitative Social Work: Research and Practice*, *19*(4), 701-718. https://doi.org/10.1177%2F1473325019850616

Watson, Latter, & Bellew. (2015a). Adopted children and young people's views on their life storybooks: The role of narrative in the formation of identities. *Children and Youth Services Review*, *58*, 90-98. https://dx.doi.org/10.1016/j.childyouth.2015.09.010

Watson, Latter, & Bellew. (2015b). Adopters' views on their children's life story books. *Adoption & Fostering*, *39*(2), 119. https://doi.org/10.1177%2F0308575915588723

Watson, Staples, & Riches. (2020). 'We need to understand what's going on because it's our life': Using sandboxing to understand children and young people's everyday conversations about care. *Children & Society*, *35*(5), 663-679. https://doi.org/10.1111/chso.12432

Watson, D., Meineck, C., & Lancaster, B. (2018). Adopted children's co-production and use of 'trove' (a digitally enhanced memory box) to better understand their care histories through precious objects. *Clinical child psychology and psychiatry*, *23*(4), 614-628. https://dx.doi.org/10.1177/1359104518776359

Watts, R. (2021). Relationships, reviews and recording: Developing practice for children in care. *Practice: Social Work in Action*, *33*(3), 207-222. https://www.tandfonline.com/doi/full/10.1080/09503153.2021.1889488

Willis, R., & Holland, S. (2009). Life story work: Reflections on the experience by looked after young people. *Adoption & Fostering*, *33*(4), 44-52. https://doi.org/10.1177%2F030857590903300406

Wood, J. (2019). West Sussex Children's imporvement board. Performance Report Against the Practice Improvement Plan Improving Quality of Life Story Work.

Wood, M., & Selwyn, J. (2017). Looked after children and young people's views on what matters to their subjective well-being. *Adoption & Fostering*, *41*(1), 20-34. https://doi.org/10.1177/0308575916686034



For peer review only - http://bmjopen.bmj.com/site/about/guidelines.xhtml
